# Modelling of clinical TQT studies outcomes from preclinical cardiovascular safety pharmacology studies using the one-step QTc model

**DOI:** 10.3389/fphar.2025.1619547

**Published:** 2025-10-10

**Authors:** Pascal Champéroux, Raafat Fares, Derek Leishman

**Affiliations:** ^1^ ERBC France, Chemin de Montifault, Baugy, France; ^2^ Eli Lilly and Company, Indianapolis, IN, United States

**Keywords:** electrocardiogram, QT interval correction, safety pharmacology, hERG blockers, QT prolongation

## Abstract

**Introduction:**

Retrospective translational analyses have shown a high rate of false negatives between clinical thorough QT studies (TQT) and preclinical cardiovascular safety pharmacology studies. The aim of this work was to model the results of clinical TQT studies from cardiovascular safety pharmacology studies conducted on a large set of reference drugs in beagle dogs by implanted telemetry.

**Methods:**

All preclinical studies were based on a standard four-animal crossover design comparing the vehicle to the reference drug. The model used was based on the one-step QTc correction model. This model was adapted in order to apply the same statistical method in preclinical studies as in clinical trials.

**Results:**

The sensitivity of the model made it possible to detect QTc prolongation of at least 5 ms with all reference hERG blockers known to cause QT prolongation in humans. Modelling of moxifloxacin (10 mg/kg, po) effects was in agreement with data published from clinical TQT studies with moxifloxacin 400 mg showing a QTc prolongation greater than 5 ms between 1 and 4 hours post-dose. This study shows a noticeable reduction in the risk of false negatives with several references drugs associated with a phenomenon of concealed QTc prolongation. Moreover, several reference drugs did change the slope of the QT/HR slope justifying the principles of the one-step QTc model.

**Conclusion:**

Modelling results of TQT studies with the one-step QTc model for applying the same statistical approach as in the clinic can improve the translational value of preclinical cardiovascular safety pharmacology studies and reduce the risk of false negatives.

## 1 Introduction

All new drug candidates based on small size chemical entities must be assessed for their effects on ventricular repolarization. Indeed, any prolongation of the duration of ventricular repolarization could increase the risk of triggering ventricular arrhythmias known as Torsades de Pointes. These arrhythmias can be fatal, as they can degenerate into ventricular fibrillation, leading to sudden cardiac death. Regulatory guidelines have been published for assessing the risk of ventricular repolarization prolongation in preclinical studies and clinical trials. These are the ICH S7B guideline (ICH S7B, 2005) for preclinical studies and the ICH E14 guideline (ICH Topic E14 The Clinical Evaluation of QT/QTc Interval Prolongation and Proarrhythmic Potential for Non-Antiarrhythmic Drugs, 2005) for clinical trials. The QT interval is used to estimate the duration of ventricular repolarization from the electrocardiogram, both in preclinical studies and in clinical trials. The QT interval must be corrected by heart rate because of its inverse dependence on heart rate. The reference model for the *in vivo* preclinical phase is based on electrocardiogram (ECG) recordings by telemetry in large animals in a standalone study following a cross-over design with placebo *versus* three dose levels. The number of animals included in such studies is low to minimize the number of animals used. The cross-over plan based on a Latin square (4 × 4) involving four animals is the plan recommended under ICH S7B good practice principles (Rossman et al., 2023) and in accordance with the 3Rs rule for animal welfare. In the clinic, effects of new drug candidates are assessed in thorough QT (TQT) studies. These studies systematically include a positive reference drug known to prolong the QT interval. Moxifloxacin 400 mg is the most frequently used positive control. The number of subjects in the clinical trial must be calculated in such a way as to detect a QTc (corrected QT) interval prolongation of more than 5 ms with moxifloxacin 400 mg. To achieve this level of sensitivity, TQT clinical studies are often carried out on several dozen subjects. The statistical methods are different, as are the methods for correcting the QT interval by heart rate. Unfortunately, retrospective translational analyses have shown a high rate of false negatives between clinical TQT (Thorough QT studies) and preclinical cardiovascular safety pharmacology studies (Park et al., 2018). The aim of this work was to assess whether applying the statistical methods used in the clinical TQT studies to preclinical cardiovascular safety pharmacology studies could reduce the risk of false negatives in clinical studies. The one-step QTc model (Chiang et al., 2006) has been used and adapted to compensate for the fact that the numbers of subjects involved are very different in clinical and preclinical studies. This work also made it possible to test the one-step QT interval correction model, the principle assumptions of which are different from that of other individual correction methods used in preclinical research.

## 2 Methods

### 2.1 Animals

All animal experiments were subjected to ethical review (Ethics Committee n ° CEEA-111) according to European directive 2010/63/UE on animal welfare.

### 2.2 Telemetry studies

In accordance with the 3Rs encouraging the reduction in the number of animals used for experimental research, only telemetry studies already recorded in the internal ERBC database from 2008 were re-analysed to apply the one-step QTc model. Results of 51 studies in total with reference drugs were analyzed in this work. Twenty-four-hour telemetric ECG recordings were collected in beagle dogs. All telemetry studies were carried out on adult (3 males and three females per group for former studies or six males per group for recent studies due to logistic reasons linked to group housing) beagle dogs (10–15 kg, 8–24 months, CEDS, Mezilles, France). In the present work, the first four animals were systematically selected on the basis of their identification number in each group and included in the statistical analysis. In fact, a sample of four animals is most common in pharmacological studies on cardiovascular safety based on telemetry. Animals were fitted with radio telemetry transmitters (TL11M2D70PCT, L11 or M11 models for ECG, Data Sciences International, Saint Paul, MN, USA). Dogs were premedicated with acetylpromazine (0.05 mg/kg, s. c.) and buprenorphine (0.01 mg/kg, s. c.). Anaesthesia was induced by thiopental (15–20 mg/kg, i. v.) and then maintained with isoflurane 0.5%–1.5% in oxygen. After left thoracotomy, one electrode was sutured directly to the left ventricular epicardium near the apex while the second electrode was sutured to the pericardium above the right atrium to approximate a limb Lead II ECG. Analgesic treatment with buprenorphine/meloxicam was continued for a minimum of 2 days to alleviate any post-operative pain. A minimum period of 3 weeks was allowed for recovery from the surgery. Animals were housed in pens in groups of two to four animals with environmental enrichment. Environmental parameters were recorded continuously and maintained within a fixed range, room temperature at 15 °C–21 °C at 45%–65% relative humidity. The artificial day/night cycle was 12 h light and 12 h darkness with light on at 07:30 a.m. Drinking water was provided *ad libitum*. Solid diet (300 g/animal) was given daily in the morning. All dosing with drugs was performed between 2:00 and 3:30 p.m. ECGs were recorded continuously for a minimum of 2 h before dosing up to 24 h post dose. Animals serve as their own control according to cross-over design with a washout period of 48–72 h at minimum between dosing sessions. ECG signal was recorded at a sampling rate of 500 Hz using ART™ acquisition software (release 4.33, Data Sciences International, St Paul, MN, USA) or Ponemah™ (release 6.33, Data Sciences International, St Paul, MN, USA).

### 2.3 Beat-to-beat analysis

Beat-to-beat RR interval and QT interval were calculated from the epicardial ECG signal using a software developed in RPL (RS/1 programming language, RS/1 release 6.3, Applied Materials). Beat-to-beat heart rate (HR) was derived from beat-to-beat RR values: HR = 60000/RR. All calculations were performed using fully automated computer procedures, validated according to GLP (Good Laboratory Practices), from beat-to-beat analysis up to statistics.

### 2.4 Conventional QT corrections

The two main conventional methods for individual correction of the QT interval are based on calculation of the slope β of the QT/RR or QT/HR relationship. A Log (RR)/Log (QT) transformation was applied to linearize the QT/RR relationship (LogQTRR model), as this relationship shows a curvature in dogs for low RR intervals (Champeroux et al., 2010). Conversely, the QT/HR relationship is almost linear in dogs and requires no transformation (LinQTHR model). The slope β_QT/RR_ is calculated individually by linear regression of the LogQT/LogRR relationships determined from all the average QT/RR pairs calculated each minute during the circadian cycle in the vehicle control period for the LogQTRR model. Similarly, the slope β_QT/HR_ is calculated individually by linear regression of the QT/HR relationships determined from all the average QT/HR pairs calculated each minute during the circadian cycle in the vehicle control period for the LinQTHR model.

QTc values are then calculated every minute as follows:

LogQTRR model: QTc = 10^(LogQT−βQT/RR.(LogRR-logRRref))^ where RRref = 750 ms in dogs.

LinQTHR model: QTc = QT-β_QT/HR_. (HR-HRref) where HRref = 80 bpm.

Finally, average QTc values are calculated every hour.

### 2.5 The one-step QTc model

**FIGURE 1:**
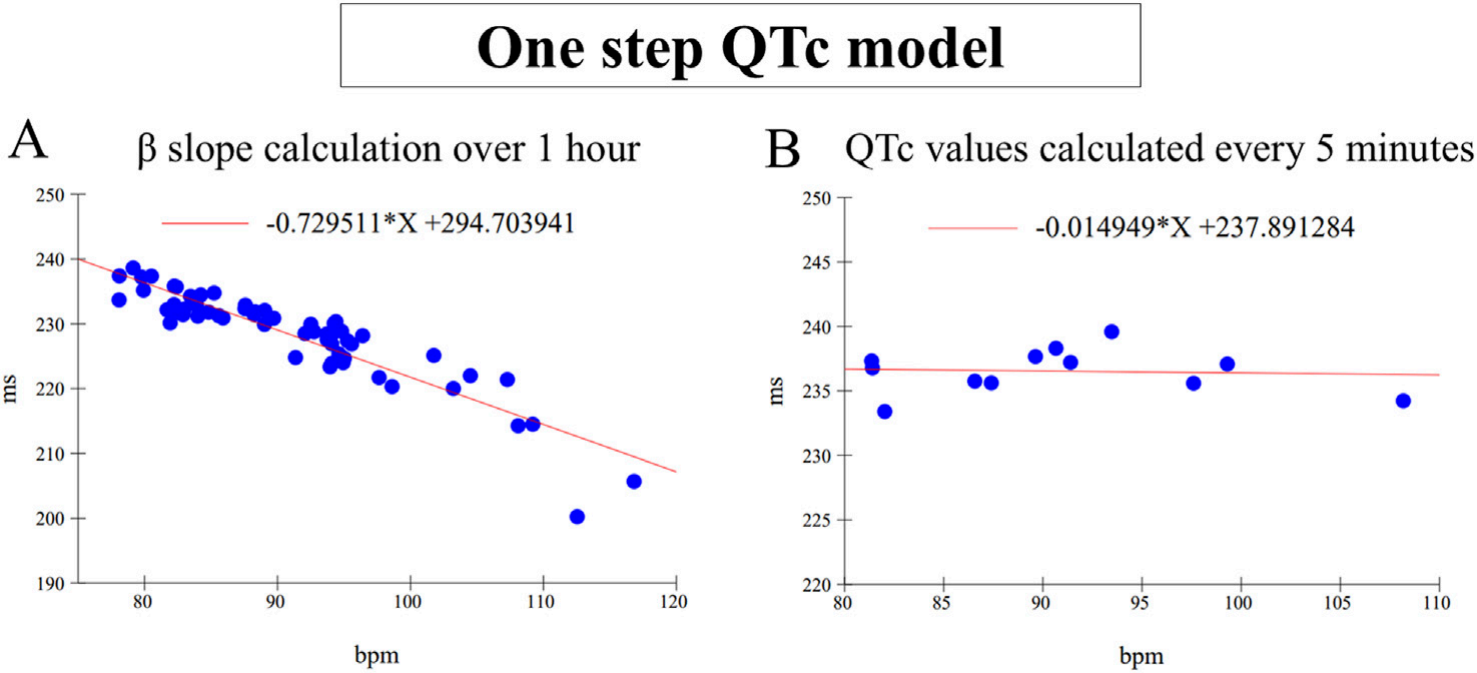
**(A)** Example of β slope calculation by linear regression carried out on 60 mean QT/RR pairs calculated every minute for 1 h. **(B)** Example of QTc values calculated every 5 min with the same β value calculated in **(A)** After correction, the slope is almost null. Note the variability of QTc values which is mainly dependent on the influence of the autonomic nervous system.

The one-step QTc model is an individual correction QT method which does not require a treatment free period for the slope calculation as other QT correction method (Leishman et al., 2023). The correction slope β of the QT/HR relationship was directly calculated individually every hour from all QT/HR pairs themselves calculated every minute (Figure 1A). Then, QTc values were calculated from mean QT and HR values calculated over 5 min sequences using the following formula: QTc = QT-β. (HR-HRref) where HRref = 80 bpm (Figure 1B). This value is a rounded average of the heart rate calculated over the circadian cycle in beagle dogs. It corresponds to a RRref value of 750 ms which is classically used in standard methods of individual QT correction based on a slope calculation from the QT/RR relationship (Holzgrefe et al., 2014). By this way, 12 QTc values are determined per hour and per animal. All QTc values were pooled for each hour to model the number of subjects incorporated in clinical trials, e.g.,48 QTc values with four animals. Single ΔQTc values were calculated from the time matched difference between QTc values calculated for each successive 5 min period in control *versus* treated sessions carried out in the same animal. The double ΔΔQTc values were calculated in the same way, with prior subtraction of the individual mean baseline calculated over the 1-h period preceding administration. Mean ΔQTc and ΔΔQTc values ± one-sided 95% confidence interval were finally calculated every hour over 24 h after dosing.

### 2.6 Statistical procedures

The lower bound of the one-sided 95% confidence interval was compared with the 5 ms threshold used in clinical studies for the positive control. Graphs are marked with *, meaning that the lower bound is above this threshold with statistical significance P ≥ 0.05. The term LSD (Least Significant Difference) which is commonly used in preclinical studies corresponds to the one-sided 95% confidence interval of mean ΔQTc and ΔQTc. Statistics were performed using RS/1 release 6.3, Applied Materials. Operators for animal experiments and data analysts were not blinded. This work does not contain any subjective observation or analyses that would justify blinding.

### 2.7 Drugs

Most of drugs used in this work are known for their hERG blocking properties. Some other are drugs known for their interaction with the autonomic nervous system. Doses were selected from preliminary trials to determine the minimum effective dose causing a QTc prolongation or to test a high dose for drugs with no effect on ventricular repolarization. All chemicals’ were purchased from Abcam Biochemicals (Abcam Biochemicals, Cambridge, United Kingdom), Carbosynth (Carbosynth Ltd., Berkshire, United Kingdom) and Sigma-Aldrich (Saint Quentin, France). Supplementary Material available online includes the full list of selected drugs, suppliers, vehicles, volumes and routes of administration.

## 3 Results

**FIGURE 2:**
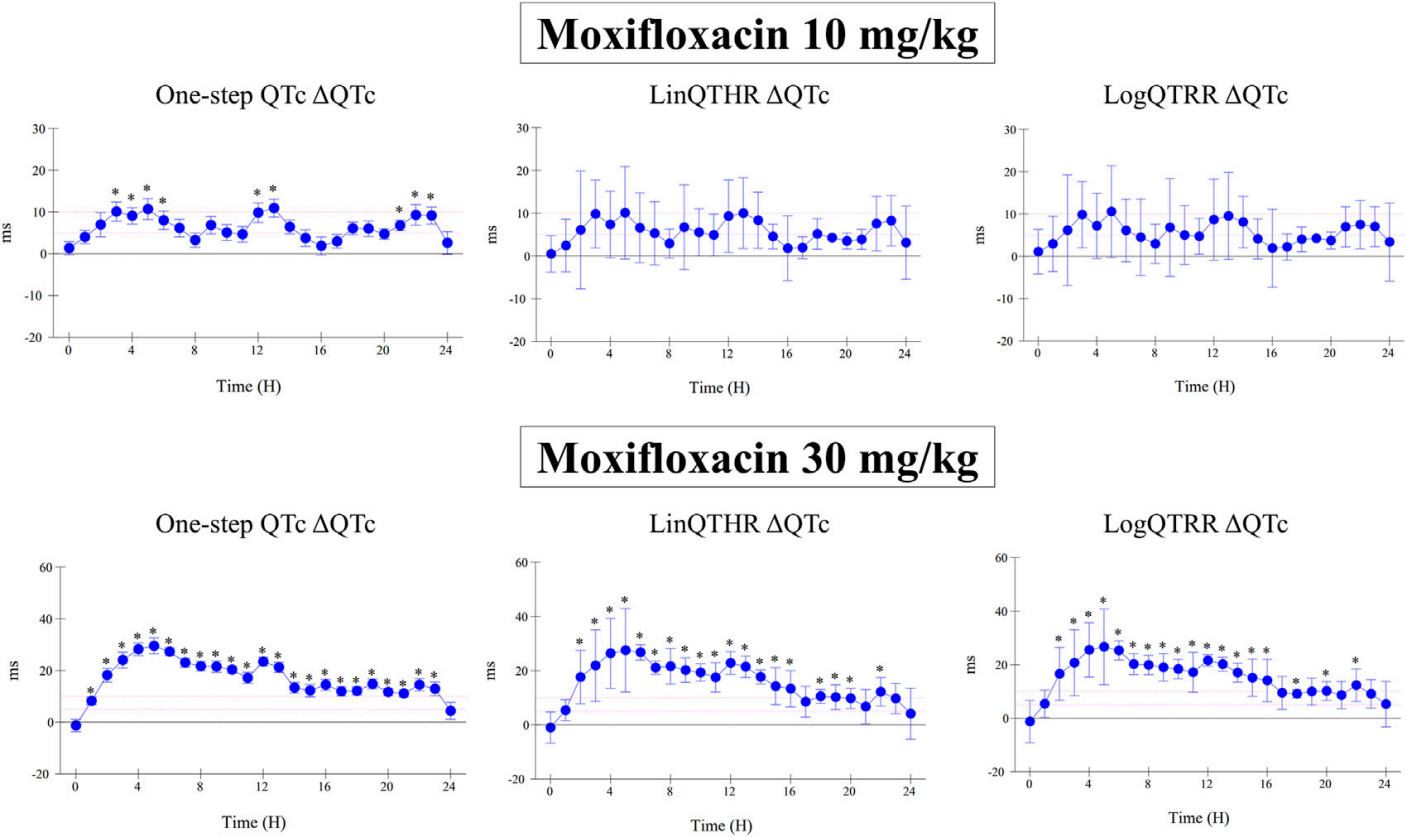
Effect of moxifloxacin at doses of 10 mg/kg and 30 mg/kg orally on QTc interval calculated with one-step QTc, LinQTHR and LogQTRR models. ΔQTc: time matched control *versus* moxifloxacin sessions. Mean difference ± one sided 95% confidence interval. *: lower bound of one sided 95% confidence interval LB ≥ 5 ms.

**FIGURE 3:**
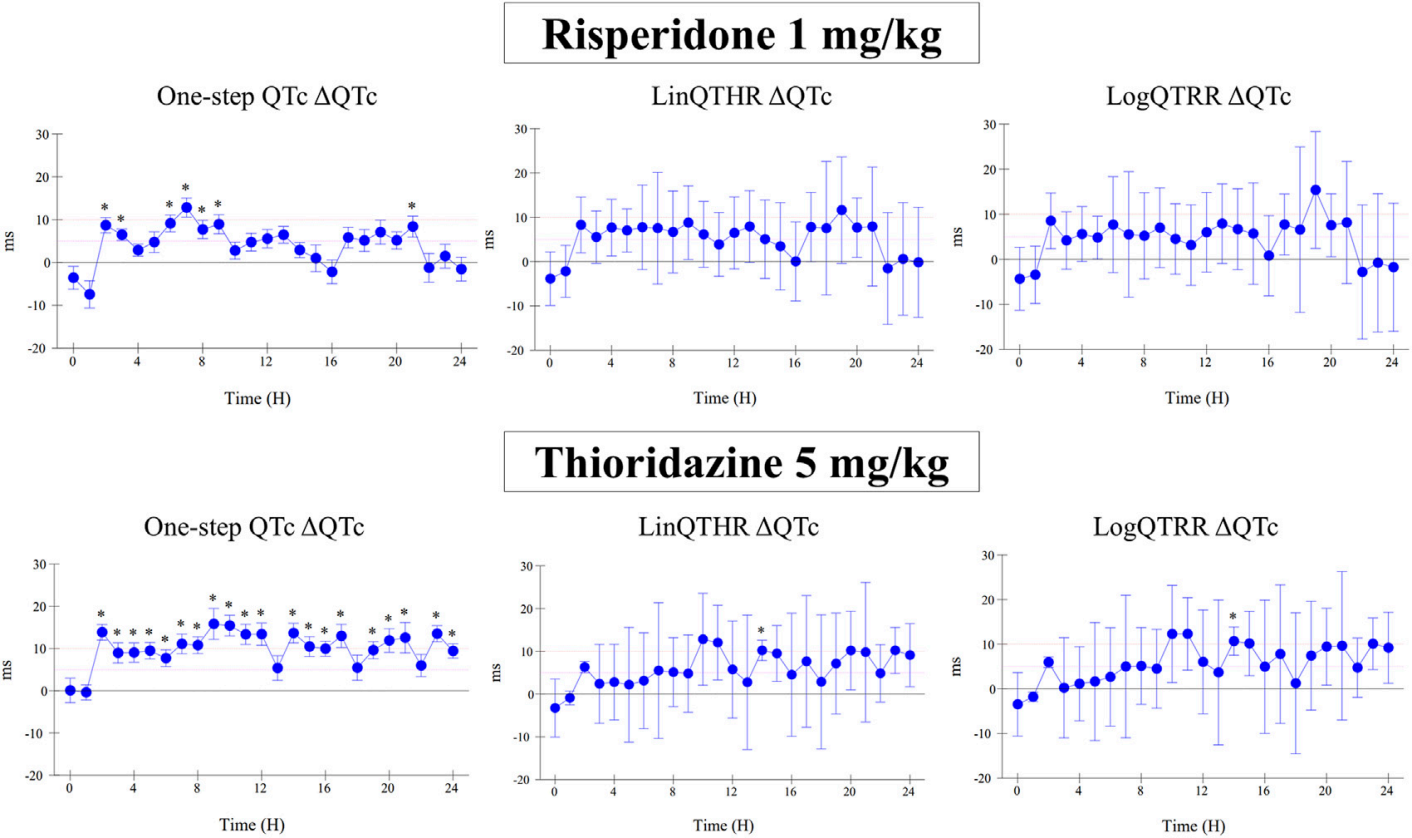
Effect of risperidone at a dose of 1 mg/kg (i.v.) and thioridazine at a dose of 5 mg/kg (po) on QTc interval calculated with one-step QTc, LinQTHR and LogQTRR models. ΔQTc: time matched control *versus* drug sessions. Mean difference ± one sided 95% confidence interval. *: lower bound of one sided 95% confidence interval LB ≥ 5 ms.

**FIGURE 4:**
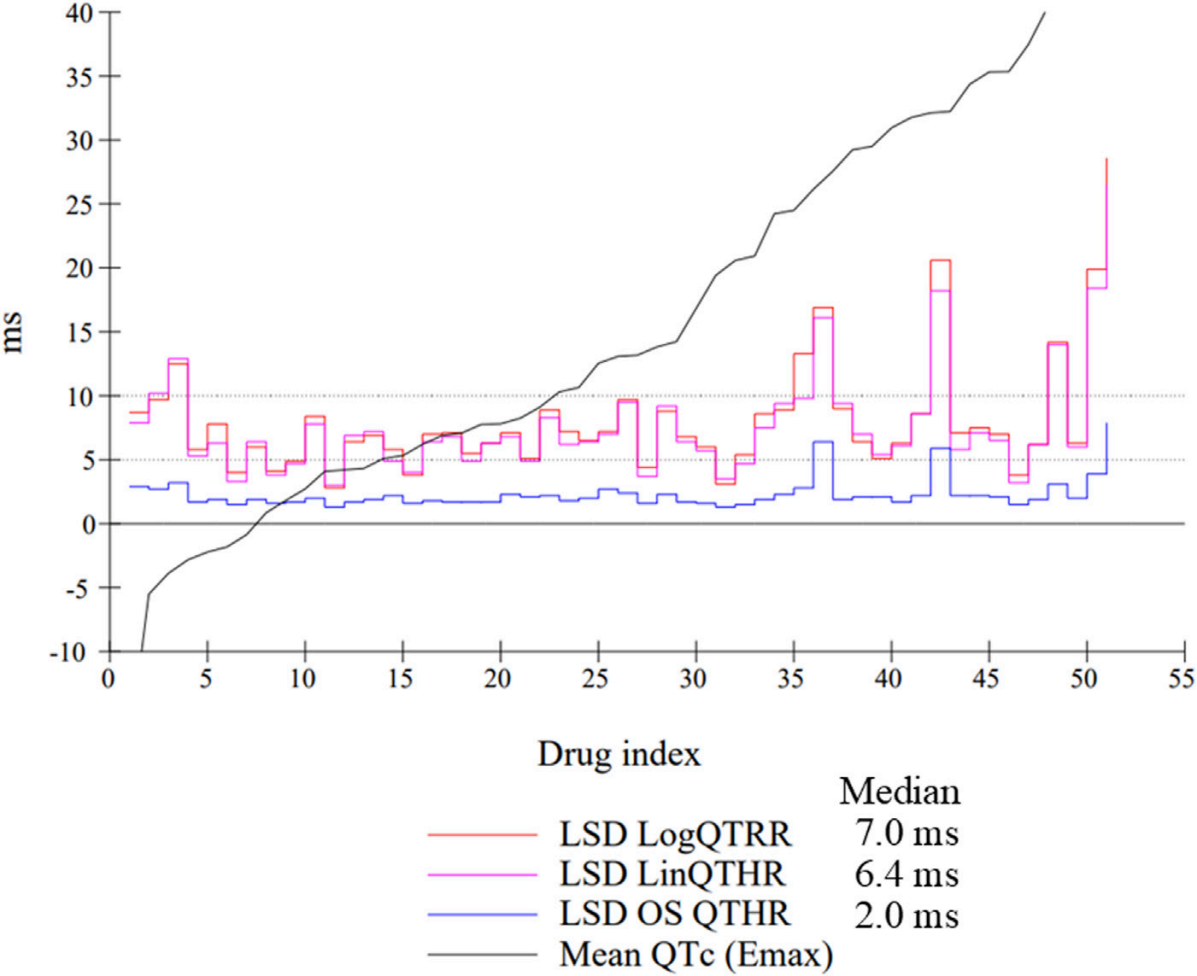
Individual LSD values from all studies (n = 51) using the LogQTRR and LinQTHR models and the one-step (OS) QTc model (ΔQTc). Median: median LSD value calculated of all studies. Drugs were ranked in ascending order on the basis of maximum effect on QTc.

**FIGURE 5:**
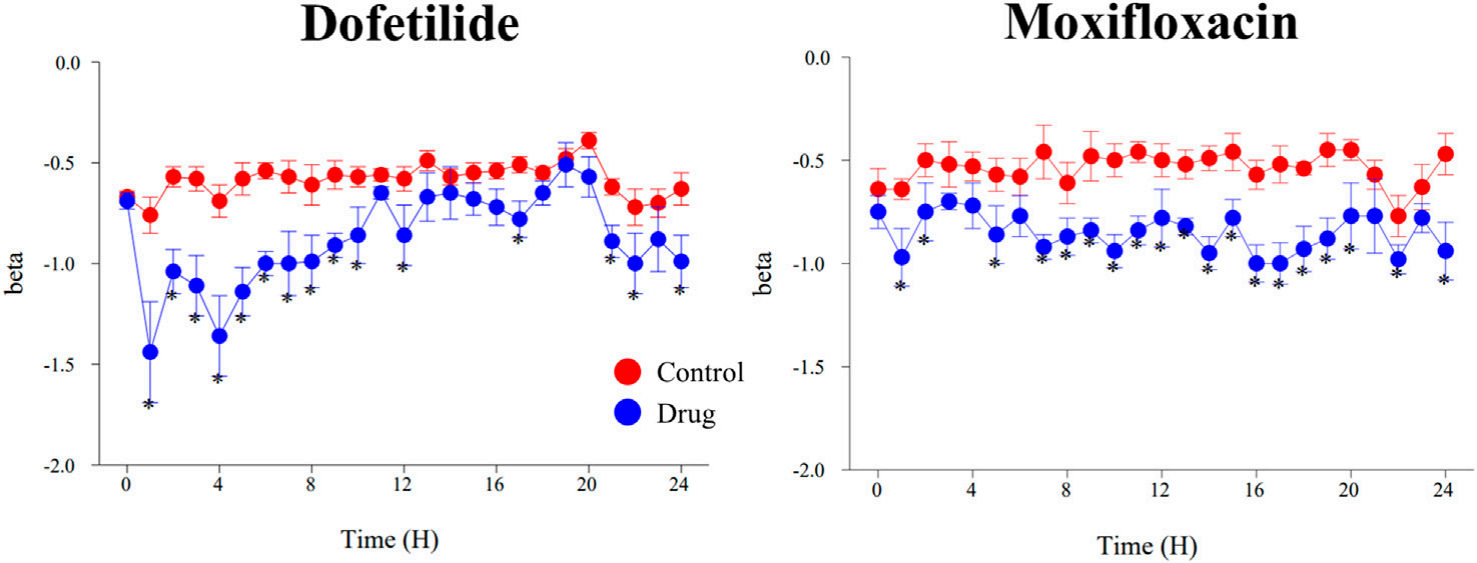
Effects of dofetilide (1 mg/kg, po) and moxifloxacin (90 mg/kg, po) on β-slope calculated with the one-step QTc model. Mean values ±SEM, *: P ≤ 0.05 when compared to control session.

The assessment of drug-induced QTc prolongation using the one-step QTc model presented in Tables 1, 2 was based on the single ΔQTc. Results obtained with ΔΔQTc are available in Supplementary Material. Table 1 shows the results obtained from studies that did not show QTc prolongation at the 5 ms threshold with the one-step QTc model. This list includes molecules that have no hERG blocking properties, such as atenolol, isoprenaline, milrinone and phenylephrine. All other molecules are known to have hERG channel blocking properties. Among these hERG blockers, there are all the molecules in the database known not to cause QT prolongation in humans: ciprofloxacin (Kang et al., 2001), ebastine (Ko et al., 1997), nicardipine (Champeroux et al., 2011), phenytoin (Champeroux et al., 2011), prazosin (Thomas et al., 2004), ranolazine (Rajamani et al., 2008) and verapamil (Kramer et al., 2013). The only hERG blocker known to cause QTc prolongation was cisapride at a low dose of 2 mg/kg (po). This dose level was also found devoid of effects on QTc interval in other studies (Toyoshima et al., 2005). Alternatively, conventional methods of QT correction also did not show any QTc prolongation except with isoprenaline when using the LinQTHR model. Table 2 shows the list of studies that showed QTc prolongation at the 5 ms threshold with the one-step QTc model. With the exception of terfenadine, which showed a delayed effect, the peak effects were observed during the first 6 hours post dosing. Except clonidine, all reference drugs showing QTc prolongation at the 5 ms threshold with the one-step QTc model are hERG blocking drugs (Redfern et al., 2003; Kramer et al., 2013): astemizole, cisapride, chlorpromazine, dofetilide, droperidol, haloperidol, ibutilide, morphine, moxifloxacin, procainamide, quinidine, risperidone, sertindole, sotalol, terfenadine, thioridazine. In parallel, conventional QTc prolongation models failed to demonstrate QTc prolongation at the 5 ms threshold in 13 out of 35 studies (38%). Among them, moxifloxacin 10 mg/kg orally failed to reach the 5 ms threshold with the conventional LogQTRR and LinQTHR models whereas it did with the one-step QTc model (Figure 2). In most cases like moxifloxacin where dose responses were available, drug induced QTc prolongation reached the 5 ms threshold with the conventional LogQTRR and LinQTHR models at higher doses only. Comparison of the results obtained with the one step QTc model shows almost no difference in terms of sensitivity with respect to the 5 ms threshold between the single ΔQTc and double ΔΔQTc modes. The most noticeable difference was the case of procainamide which reached the threshold of 5 ms with ΔQTc (Table 2) but not with ΔΔQTc. The choice of presenting only ΔQTC results reflects a preference for this model. This avoids attributing too much weight to baseline values. Interestingly, drugs exhibiting a phenomenon of concealed QTc prolongation with conventional models were revealed with the one-step QTc model and reached directly the 5 ms threshold: chlorpromazine, risperidone, sertindole and thioridazine (Table 2; Figure 3, risperidone and thioridazine). LSD median values from all studies (n = 51) which correspond to one-sided 95% confidence interval were 7 ms with the LogQTRR model, 6.4 ms with the LinQTHR model and 2 ms with the one-step QTc model (Figure 4). Peaks observed with all models for drug n° 36, 42 and 51 correspond to studies of effects of haloperidol (1, 3 and 10 mg/kg). Lack of sensitivity in these three studies were attributed to interference with marked neurobehavioral effects. Given that β-slope is calculated individually each hour separately in control and treatment sessions using the one-step QTc model, the effects of drugs on β-slope can be compared to control. Almost all hERG blocking drugs known as causing torsades de pointes in human induced changes in β-slope. The effects were more or less pronounced depending on the drug, and frequently observed around the peak of the QTc effects. The most marked effects were decreases in β-slope with dofetilide and moxifloxacin at a high dose (Figure 5).

**TABLE 1:** List of studies with reference drugs that did not show QTc prolongation at the 5 ms threshold with the one-step QTc model (OS linQTHR: single ΔQTc) compared with conventional individual QT correction models, LogQTRR and LinQTRR. Drugs were ranked in ascending order on the basis of maximum effect on QTc. In brackets: one sided 95% lower bound and upper bound. In square brackets: time of peak effect (in hour) over the 6 hours post dosing. Green: lower bound of one sided 95% confidence interval LB < 5 ms. Red: lower bound of one sided 95% confidence interval LB ≥ 5 ms. HR: Maximum change in heart rate in bpm.

Treatment	HR	LogQTRR	LinQTHR	OS LinQTHR
Nicardipine 30 mg/kg po	73.5	(‐24.6;‐9.6) [4]	(0;10.1) [1]	(-20.7;-14.8) [6]
Milrinone 3 mg/kg iv	20.4	(-7.3;6.7) [6]	(-6.8;4.8) [6]	(-7.3;-3.7) [6]
Milrinone 1 mg/kg iv	9.3	(-9.5;4.2) [6]	(-10.3;3.8) [6]	(-6.2;-1.6) [6]
Phenytoin 100 mg/kg po	0.5	(-4.2;3.8) [1]	(-4;3.3) [1]	(-4.3;-1.4) [2]
Verapamil 30 mg/kg po	10.6	(-15.5;0.6) [1]	(-2.7;-0.9) [1]	(-4.9;0.5) [2]
Verapamil 10 mg/kg po	5.3	(-3.1;-1) [6]	(-3.2;-1) [6]	(-2.9;-0.7) [6]
Prazosin 10 mg/kg po	17.5	(-1.8;5.9) [6]	(-3.5;3.9) [6]	(-2.4;0.7) [1]
Ranolazine 50 mg/kg po	4.0	(-2.1;4.7) [5]	(-1.5;3.2) [1]	(-0.9;2.6) [5]
Verapamil 3 mg/kg po	-1.4	(-3.4;6.8) [3]	(-3.4;9) [3]	(0.3;3.3) [3]
Ebastine 30 mg/kg po	0.3	(0.5;7.4) [1]	(0.2;9.3) [1]	(1.3;4.2) [1]
Cisapride 2 mg/kg po	0.5	(1.1;4.3) [5]	(1;3) [5]	(2.2;5.9) [3]
Phenylephrine 1 mg/kg po	-10.2	(-3.7;8.2) [3]	(-3;9.1) [3]	(2.6;5.9) [4]
Atenolol 1 mg/kg iv	-12.5	(-5.9;7) [3]	(-6.1;7.6) [3]	(1.6;7) [6]
Nicardipine 3 mg/kg po	0.9	(4.1;11.2) [2]	(2.8;7.2) [6]	(3.9;6.3) [6]
Isoprenaline 1 mg/kg po	26.9	(4.1;12.5) [1]	(7.2;17.3) [2]	(4.2;6.4) [2]
Ciprofloxacin 100 mg/kg po	0.6	(2.3;8) [3]	(1.8;8.1) [3]	(4.9;7.5) [2]

**TABLE 2:** List of studies with reference drugs showing QTc prolongation at the 5 ms threshold with the one-step QTc model (OS linQTHR: single ΔQTc) compared with conventional individual QT correction models, LogQTRR and LinQTRR. Drugs were ranked in ascending order on the basis of maximum effect on QTc. In brackets: one sided 95% lower bound and upper bound. In square brackets: time of peak effect (in hour) over the 6 hours post dosing. Green: lower bound of one sided 95% confidence interval LB < 5 ms. Red: lower bound of one sided 95% confidence interval LB ≥ 5 ms. HR: Maximum change in heart rate in bpm.

Treatment	HR	LogQTRR	LinQTHR	OS LinQTHR
Procainamide 10 mg/kg iv	-3.9	(0.5;14.4) [1]	(1.3;13.8) [1]	(5.5;8.3) [1]
Quinidine 3 mg/kg po	-7.9	(3.3;10) [4]	(4;8.5) [4]	(5.7;8.5) [4]
Risperidone 1 mg/kg iv + atenolol	0.2	(2.6;14.3) [4]	(2.5;13.8) [3]	(6.4;9.1) [4]
Thioridazine 1.5 mg/kg po	5.2	(2.3;15.4) [4]	(1.9;15) [4]	(5.6;10.1) [3]
Chlorpromazine 1 mg/kg iv	11.1	(2.8;11) [2]	(3;12.4) [2]	(6.6;9.9) [1]
Risperidone 1 mg/kg iv	19.5	(2.4;14.7) [2]	(2.2;11.9) [5]	(7.2;11.1) [6]
Quinidine 10 mg/kg po	-10.9	(2.1;17.6) [6]	(4.2;16.6) [6]	(8.9;11.7) [6]
Moxifloxacin 10 mg/kg po	-2.7	(2;17.6) [3]	(1.9;17.7) [3]	(8.1;13.2) [5]
Thioridazine 1.5 mg/kg po + atenolol	-6.2	(6.3;15.3) [4]	(7.7;15) [4]	(10.6;14.5) [3]
Sertindole 1 mg/kg iv	16.8	(3.7;17.4) [1]	(2.3;17.9) [1]	(10.8;15.4) [1]
Sotalol 3 mg/kg po	-8.8	(8.7;12.1) [2]	(8.8;13.9) [2]	(11.5;14.9) [3]
Thioridazine 5 mg/kg po	14.7	(4.8;7.1) [2]	(5;7.6) [2]	(12;15.7) [2]
Procainamide 30 mg/kg iv	10.2	(12.4;20.3) [1]	(12.8;20.5) [1]	(12.9;15.6) [1]
Clonidine 0.1 mg/kg iv	-25.1	(4;6.7) [4]	(5.5;16.7) [4]	(14.9;18.8) [3]
Cisapride 6 mg/kg po	9.9	(6;10.6) [6]	(4.9;16.2) [2]	(14.5;24.3) [3]
Ibutilide 1 mg/kg iv	-3.7	(10;20.6) [4]	(10.9;26.8) [2]	(18.5;22.6) [2]
Quinidine 30 mg/kg po	8.5	(11;29.8) [4]	(10.5;28.6) [4]	(19.5;22.3) [4]
Terfenadine 30 mg/kg po	3.0	(17.2;27.2) [20]	(15.6;29) [20]	(22.1;26.4) [20]
Thioridazine 20 mg/kg po	21.9	(11.5;27.9) [2]	(14.1;24.8) [2]	(20.5;28.5) [1]
Haloperidol 1 mg/kg po	1.6	(0;10.8) [2]	(1.9;10.1) [2]	(19.8;32.5) [5]
Sotalol 10 mg/kg po	-12.9	(12.1;29.2) [5]	(14.1;32.3) [5]	(24.5;30.7) [3]
Morphine 2 mg/kg sc	-32.1	(13.9;28.2) [2]	(21.7;31.8) [2]	(27.3;31.2) [2]
Moxifloxacin 30 mg/kg po	-7.8	(21.7;28.9) [6]	(23.9;29.6) [6]	(26.4;32.6) [5]
Droperidol 3 mg/kg iv	2.8	(23.4;31.6) [1]	(22;29.7) [1]	(28.7;33.2) [1]
Thioridazine 20 mg/kg po + atenolol	1.7	(22.8;34) [2]	(23;30.9) [2]	(28.8;34.7) [2]
Haloperidol 10 mg/kg po	6.5	(2.7;20) [1]	(1.8;18.9) [1]	(26.1;38.1) [2]
Pimozide 1 mg/kg iv	16.2	(10.1;31.2) [2]	(11.8;26.5) [2]	(30.6;33.9) [2]
Terfenadine 100 mg/kg po	-3.5	(16.6;36.9) [23]	(16;34.7) [23]	(32.1;36.6) [23]
Sotalol 30 mg/kg po	-4.2	(30.3;31.4) [6]	(30.3;43.7) [5]	(33.9;36.7) [5]
Astemizole 1 mg/kg iv	5.8	(27.5;38.3) [2]	(24.6;34.6) [2]	(32.7;38) [1]
Sertindole 1 mg/kg iv + atenolol	-4.5	(23.7;37.2) [1]	(22.2;35.4) [1]	(35.5;39.5) [1]
Moxifloxacin 90 mg/kg po	-8.9	(23.4;52.7) [5]	(22.8;52.3) [5]	(37.2;43.7) [6]
Dofetilide 0.1 mg/kg po	4.0	(34.4;39.1) [5]	(33;38.5) [5]	(40.4;43.9) [2]
Dofetilide 1 mg/kg po	1.4	(42.3;70.4) [3]	(40.3;67.1) [3]	(65.6;68.2) [4]
Haloperidol 3 mg/kg po	28.4	(19.1;28.3) [5]	(16.7;30.2) [5]	(68;128.4) [5]

## 5 Discussion

This work demonstrates that is possible to adopt the same criteria for assessing the risk of QTc prolongation in standalone preclinical safety pharmacology studies as in the clinical thorough QT studies (TQT). In a retrospective analysis carried out from the HESI/FDA database on 150 drugs (Park et al., 2018), 43 tested positive in clinical TQT studies. Of these 43 TQT-positive drugs, 28 showed no evidence of QT prolongation or hERG channel blocking properties in preclinical studies in the concentration range up to 30 times free Cmax showing QT prolongation in TQT studies (Valentin et al., 2022). The proportion of false-negative results in preclinical studies is large (65%), and called into question the strategy proposed in the ICH S7B guideline (Bass et al., 2015). Such discrepancies in terms of free Cmax at therapeutic concentration *versus* IC50 values for hERG channel inhibition have long been known (Redfern et al., 2003), since the authors initially proposed an hERG IC50/free Cmax safety margin >30-fold. Even larger safety margins of up to 50-fold or 100-fold have been proposed on the basis of retrospective studies (Leishman et al., 2024). Conversely, such discrepancies in *vivo* effects on the QT interval between preclinical and clinical studies were not expected, and are in contradiction with analyses of the scientific literature showing good qualitative concordance (>90%) between humans and animals for drugs that prolong the QT interval in humans (Vargas et al., 2015). A more detailed analysis of the reasons for the high level of false negatives reported for the HESI/FDA database revealed problems of missing or incomplete data in a number of cases, but these problems are far from explaining most of the discrepancies. “Good practices” have been defined in the form of questions and answers (ICH E14/S7B Q&A, 2022) to complement the ICH S7B guideline, in order to ensure better quality of the studies and data produced, while standardizing the methods used. Individual methods of QT correction are now clearly recommended over traditional QT correction formulas such as those of Bazett or Fridericia. The notion of statistical sensitivity is also highlighted. It is interesting to note that this document deals with the same statistical topics for clinical and preclinical studies, but in different ways. Two points are worth highlighting. First, the interpretation criteria are precise in the clinical section: the proarrhythmic risk is considered low if the upper bound of the confidence interval of the effect on the QTc interval is less than 10 ms. If the upper bound is greater than 10 ms, the hypothesis of a prolongation of the QTc interval cannot be excluded. Conversely, there are no precise criteria for characterizing a drug induced QTc prolongation in preclinical studies. Secondly, a positive control is systematically used in TQT studies to assess the assay sensitivity. Here again, the criteria are very precise in the sense that positive control must show a prolongation of the QTc interval >5 ms i.e., lower bound of one sided 95% confidence interval >5 ms. On the preclinical side, there are no strong recommendations related to common requirement or practice. No positive control is systematically included to assess the statistical sensitivity of the studies. Assessment of assay sensitivity should be based on the notion of minimum detectable difference (MDD) (Duquesne et al., 2020). The MDD, like the LSD (Least Significant Difference), provides a statistical evaluation of the minimum effect compared to zero that could be detected under the experimental conditions of the studies, but they do not constitute proof that the studies were capable of detecting QTc prolongation by a hERG channel blocker drug as in clinical trials with the positive control. Moxifloxacin 400 mg is the most commonly used positive control in clinical TQT studies. Several studies have shown that an oral dose of 10 mg/kg in dogs (Chen et al., 2005; Chui et al., 2021) results in equivalent exposure in humans (Yan et al., 2010), with a Cmax of around 3,000 ng/mL in both species. The present study shows that modelling moxifloxacin effects at 10 mg/kg orally with the one-step QTc model provides extremely close ΔQTc values as found in humans with moxifloxacin 400 mg, i.e., above 5 ms and close to 10 ms in average. At the same time, conventional QTc correction methods have not demonstrated in this study their ability to detect the effects of moxifloxacin at a dose of 10 mg/kg with a threshold greater than 5 ms, as in TQT studies. These results confirm that the use of moxifloxacin as a positive control at a dose that achieves the same exposure and QTc prolongation as in humans is not appropriate for assessing the sensitivity of experimental conditions in preclinical studies with conventional QT correction methods. On the other hand, the results obtained with this variant of the one-step QTc model demonstrate that this approach would be possible. This would clearly be the best way to improve the translation of results from preclinical to clinical studies. Beyond this question of positive control, the results of this work overall show an excellent ability of the one-step QTc model to detect small-amplitude effect prolongations slightly greater than 5 ms with the same threshold criterion as the clinic, in contrast to conventional individual correction methods. The model also directly reveals QTc prolongations that were initially reported as concealed QT prolongations, as they were only revealed in the presence of a beta blocking agent such as atenolol with conventional QT correction methods (Champeroux et al., 2010; Champéroux et al., 2022). It is also interesting to note that drugs such as ranolazine, verapamil and ciprofloxacin fall below the 5 ms threshold in line with their low proarrhythmic risk classification. The thresholds chosen in TQT studies, i.e. 5 ms and 10 ms for the lower and upper bounds respectively, are challenging. Many mechanisms other than hERG channel blockade can induce QTc prolongation in humans like decrease in body temperature, hyperkaliemia, changes in glycemia or in autonomic control (Valentin et al., 2022). Increase in parasympathetic activity can also induce mild QTc prolongation that could be appear as positive in thorough QT studies as suggested by results obtained with clonidine. It is therefore important to have a predictive approach to the risk of QTc interval prolongation in humans in the preclinical phase, in order to anticipate or even avoid the need to carry out TQT studies, considered by some sponsors to be costly (Fermini et al., 2016). Alternatively, *in vitro* approaches on isolated cardiomyocytes or *in silico* approaches based on multichannel patch-clamp profiles are real advances in proarrhythmic risk assessment (Yim, 2018). However, the CIPA (Comprehensive *In vitro* Proarrhythmia Assay) approach cannot directly simulate the results of TQT studies, as could preclinical *in vivo* studies using the same interpretation criteria as the clinical study. In theory, this statistical model could be fully applicable to humans, provided that electrocardiogram recordings are made using the Holter method to obtain continuous recordings over 24 h. Indeed, the One-step QTc model enables direct calculation of correction slopes independently of treatments. This study also shows that the calculation of slopes during treatment is necessary to correctly correct QT values. Several torsadogenic drugs such as moxifloxacin have shown significant effects on the slope of QT/HR relationships. These changes in QT/HR or QT/RR relationship are consistent with the concept of abnormal restitution of QT-TQ relationships and impaired QT interval hysteresis which increases vulnerability to arrhythmias described for certain hERG channel blocking drugs (Fossa, 2008).

The main limitation of *in vivo* preclinical studies is the number of animals included in the design of stand-alone safety pharmacology studies. This number is low, commonly 4, for several reasons. The first, rather obvious reason is that conducting this type of study on fewer than four animals would not ensure a level of statistical sensitivity deemed satisfactory by most safety pharmacologists. The second is that the recommended design for this type of study is a Latin square cross-over (Rossman et al., 2023). As the studies involve four groups (vehicle and three dose levels), the number of four makes for a perfect balanced Latin square. The third reason relates to the cost of the studies, both financial and ethical. Four animals is indeed a good compromise shared by many researchers in the field. The basis of the model used in this study is to make the best possible use of the data recorded on this small number of animals to mimic the large number of subjects involved in clinical trials. The choice of calculating a QTc value from all the beats recorded over successive 5-min sequences has two advantages. The first is to smooth out the phenomenon of QT interval hysteresis reflecting delayed adaptation of the QT interval in response to changes in heart rate. This is the principle of the probabilistic method proposed by Holzegrefe, which involves calculating a mean value from a minimum number of 250 beats in the dog, i.e., for 5 min approximately (Holzgrefe et al., 2007). The second advantage is to calculate 12 successive average values per hour, mimicking the number of subjects in a TQT-type clinical trial (4 animals x 12 values = 48 values/hour). These 12 values are clearly not independent, which is just as clearly a breach of the principle of statistical rules. Nevertheless, they display a certain level of variability whose component is mainly related to the activity of the autonomic nervous system, which in fact reflects the animal’s level of activity and excitement which is unlikely to be constant over an hour. This source of variability is used to mimic the inter-subject variability in the model. In clinical trials, the number of subjects is often close to 40 to 60, depending on studies (Yan et al., 2010).

In conclusion, this statistical model makes it possible to take this phenomenon into account in the process of QT correction. In addition, it could ultimately enable empirical correction formulas such as the Fridericia or Bazett formula to be abandoned or at least challenged. This work carried out on a large set of hERG blockers makes it possible to imagine designing pilot clinical studies on a small number of subjects to apply the model along the same lines as those described in this study, before launching a full TQT study. This transposition of the present model of QT correction to humans could lead to a better translation between preclinical and clinical results when assessing the risk of ventricular repolarization prolongation and reduce the risk of false negatives.

## Data Availability

The original contributions presented in the study are publicly available. This data can be found here: https://figshare.com/articles/journal_contribution/One_step_QTc/29900846?file=57159332.

## Abbreviations

BP, Blood pressure; ECG, electrocardiogram; HR, heart rate; QTc, corrected QT interval; TQT, thorough QT.
