# Supplementary material for: Modelling of clinical TQT studies outcomes from preclinical cardiovascular safety pharmacology studies using the one-step QTc model
Source: Front Pharmacol. 2025 Oct 10;16:1619547. doi: 10.3389/fphar.2025.1619547 (PMC12549647; doi:10.3389/fphar.2025.1619547)
Supplement: Supplementary file 1 [file DataSheet1.pdf]

One step QTc vs conventional QTc correction in $\triangle$QTc and $\triangle\triangle$QTc mode

Pascal Champeroux

April 18, 2025

AIM

This document presents graphs comparing the modeling of QTc changes using the one-step QTc model to conventional methods of individual QT correction. The comparison was carried out on a large number of drugs (51 studies), most of them hERG blocking drugs (90%).

The aim was to apply the same statistical method and criteria in these preclinical studies as in thorough QT studies (TQT).

This document also reports hourly changes in the β slope of the QT/HR relationship which is used in the one-step QTc model.

STATISTICS

The lower bound of the one-sided 95% confidence interval was compared with the 5 ms threshold used in clinical studies for the positive control. Graphs are marked with $\star$, meaning that the lower bound is above this threshold with statistical significance P$\geq$0.05. The term LSD (Least Significant Difference) which is commonly used in preclinical studies corresponds directly to the one-sided 95% confidence interval of mean single Δ dQTc and double $\Delta\Delta$ ddQTc.

TITLE PAGE 1

CONTENTS 2

Table 1: Drug information 8

Comparative summary tables 9

Table 2: OS QTc vs conventional QTc Maximum UB over the first 6 hours - dQTc n=4 . . . 10

Table 3: OS QTc vs conventional QTc Maximum UB over the first 6 hours - ddQTc n=4 . . 11

Astemizole 1 mg/kg iv 12

Figure 1 Astemizole 1 mg/kg iv 13

Figure 2: Astemizole 1 mg/kg iv - Effect on β slope (one step QTc model) 14

Atenolol 1 mg/kg iv 15

Figure 3 Atenolol 1 mg/kg iv 16

Figure 4: Atenolol 1 mg/kg iv - Effect on β slope (one step QTc model) 17

Ciprofloxacin 100 mg/kg po 18

Figure 5 Ciprofloxacin 100 mg/kg po 19

Figure 6: Ciprofloxacin 100 mg/kg po - Effect on β slope (one step QTc model) 20

Cisapride 2 mg/kg po 21

Figure 7 Cisapride 2 mg/kg po 22

Figure 8: Cisapride 2 mg/kg po - Effect on β slope (one step QTc model) 23

Cisapride 6 mg/kg po 24

Figure 9 Cisapride 6 mg/kg po 25

Figure 10: Cisapride 6 mg/kg po - Effect on β slope (one step QTc model) 26

Clonidine 0.1 mg/kg iv 27

Figure 11 Clonidine 0.1 mg/kg iv 28

Figure 12: Clonidine 0.1 mg/kg iv - Effect on β slope (one step QTc model) 29

Chlorpromazine 1 mg/kg iv 30

Figure 13 Chlorpromazine 1 mg/kg iv 31

Figure 14: Chlorpromazine 1 mg/kg iv - Effect on β slope (one step QTc model) 32

Dofetilide 0.1 mg/kg po 33

Figure 15 Dofetilide 0.1 mg/kg po 34

Figure 16: Dofetilide 0.1 mg/kg po - Effect on β slope (one step QTc model) 35

Dofetilide 1 mg/kg po **36**
Figure 17 Dofetilide 1 mg/kg po . . . 37
Figure 18: Dofetilide 1 mg/kg po - Effect on β slope (one step QTc model) . . . 38

Droperidol 3 mg/kg iv **39**
Figure 19 Droperidol 3 mg/kg iv . . . 40
Figure 20: Droperidol 3 mg/kg iv - Effect on β slope (one step QTc model) . . . 41

Ebastine 30 mg/kg po **42**
Figure 21 Ebastine 30 mg/kg po . . . 43
Figure 22: Ebastine 30 mg/kg po - Effect on β slope (one step QTc model) . . . 44

Haloperidol 1 mg/kg po **45**
Figure 23 Haloperidol 1 mg/kg po . . . 46
Figure 24: Haloperidol 1 mg/kg po - Effect on β slope (one step QTc model) . . . 47

Haloperidol 3 mg/kg po **48**
Figure 25 Haloperidol 3 mg/kg po . . . 49
Figure 26: Haloperidol 3 mg/kg po - Effect on β slope (one step QTc model) . . . 50

Haloperidol 10 mg/kg po **51**
Figure 27 Haloperidol 10 mg/kg po . . . 52
Figure 28: Haloperidol 10 mg/kg po - Effect on β slope (one step QTc model) . . . 53

Ibutilide 1 mg/kg iv **54**
Figure 29 Ibutilide 1 mg/kg iv . . . 55
Figure 30: Ibutilide 1 mg/kg iv - Effect on β slope (one step QTc model) . . . 56

Isoprenaline 1 mg/kg po **57**
Figure 31 Isoprenaline 1 mg/kg po . . . 58
Figure 32: Isoprenaline 1 mg/kg po - Effect on β slope (one step QTc model) . . . 59

Milrinone 1 mg/kg iv **60**
Figure 33 Milrinone 1 mg/kg iv . . . 61
Figure 34: Milrinone 1 mg/kg iv - Effect on β slope (one step QTc model) . . . 62

Milrinone 3 mg/kg iv **63**
Figure 35 Milrinone 3 mg/kg iv . . . 64
Figure 36: Milrinone 3 mg/kg iv - Effect on β slope (one step QTc model) . . . 65

Morphine 2 mg/kg sc 66
Figure 37 Morphine 2 mg/kg sc . . . 67
Figure 38: Morphine 2 mg/kg sc - Effect on β slope (one step QTc model) . . . 68

Moxifloxacin 10 mg/kg po 69
Figure 39 Moxifloxacin 10 mg/kg po . . . 70
Figure 40: Moxifloxacin 10 mg/kg po - Effect on β slope (one step QTc model) . . . 71

Moxifloxacin 30 mg/kg po 72
Figure 41 Moxifloxacin 30 mg/kg po . . . 73
Figure 42: Moxifloxacin 30 mg/kg po - Effect on β slope (one step QTc model) . . . 74

Moxifloxacin 90 mg/kg po 75
Figure 43 Moxifloxacin 90 mg/kg po . . . 76
Figure 44: Moxifloxacin 90 mg/kg po - Effect on β slope (one step QTc model) . . . 77

Nicardipine 3 mg/kg po 78
Figure 45 Nicardipine 3 mg/kg po . . . 79
Figure 46: Nicardipine 3 mg/kg po - Effect on β slope (one step QTc model) . . . 80

Nicardipine 30 mg/kg po 81
Figure 47 Nicardipine 30 mg/kg po . . . 82
Figure 48: Nicardipine 30 mg/kg po - Effect on β slope (one step QTc model) . . . 83

Phenylephrine 1 mg/kg po 84
Figure 49 Phenylephrine 1 mg/kg po . . . 85
Figure 50: Phenylephrine 1 mg/kg po - Effect on β slope (one step QTc model) . . . 86

Phenytoin 100 mg/kg po 87
Figure 51 Phenytoin 100 mg/kg po . . . 88
Figure 52: Phenytoin 100 mg/kg po - Effect on β slope (one step QTc model) . . . 89

Pimozide 1 mg/kg iv 90
Figure 53 Pimozide 1 mg/kg iv . . . 91
Figure 54: Pimozide 1 mg/kg iv - Effect on β slope (one step QTc model) . . . 92

Prazosin 10 mg/kg po 93
Figure 55 Prazosin 10 mg/kg po . . . 94
Figure 56: Prazosin 10 mg/kg po - Effect on β slope (one step QTc model) . . . 95

Procainamide 10 mg/kg iv 96
Figure 57 Procainamide 10 mg/kg iv . . . 97
Figure 58: Procainamide 10 mg/kg iv - Effect on β slope (one step QTc model) . . . 98

Procainamide 30 mg/kg iv 99
Figure 59 Procainamide 30 mg/kg iv . . . 100
Figure 60: Procainamide 30 mg/kg iv - Effect on β slope (one step QTc model) . . . 101

Quinidine 3 mg/kg po 102
Figure 61 Quinidine 3 mg/kg po . . . 103
Figure 62: Quinidine 3 mg/kg po - Effect on β slope (one step QTc model) . . . 104

Quinidine 10 mg/kg po 105
Figure 63 Quinidine 10 mg/kg po . . . 106
Figure 64: Quinidine 10 mg/kg po - Effect on β slope (one step QTc model) . . . 107

Quinidine 30 mg/kg po 108
Figure 65 Quinidine 30 mg/kg po . . . 109
Figure 66: Quinidine 30 mg/kg po - Effect on β slope (one step QTc model) . . . 110

Ranolazine 50 mg/kg po 111
Figure 67 Ranolazine 50 mg/kg po . . . 112
Figure 68: Ranolazine 50 mg/kg po - Effect on β slope (one step QTc model) . . . 113

Risperidone 1 mg/kg iv 114
Figure 69 Risperidone 1 mg/kg iv . . . 115
Figure 70: Risperidone 1 mg/kg iv - Effect on β slope (one step QTc model) . . . 116

Risperidone 1 mg/kg iv + atenolol 117
Figure 71 Risperidone 1 mg/kg iv + atenolol . . . 118
Figure 72: Risperidone 1 mg/kg iv + atenolol - Effect on β slope (one step QTc model) . . . 119

Sotalol 3 mg/kg po 120
Figure 73 Sotalol 3 mg/kg po . . . 121
Figure 74: Sotalol 3 mg/kg po - Effect on β slope (one step QTc model) . . . 122

Sotalol 10 mg/kg po 123
Figure 75 Sotalol 10 mg/kg po . . . 124
Figure 76: Sotalol 10 mg/kg po - Effect on β slope (one step QTc model) . . . 125

Sotalol 30 mg/kg po 126
Figure 77 Sotalol 30 mg/kg po . . . 127
Figure 78: Sotalol 30 mg/kg po - Effect on β slope (one step QTc model) . . . 128

Sertindole 1 mg/kg iv 129
Figure 79 Sertindole 1 mg/kg iv . . . 130
Figure 80: Sertindole 1 mg/kg iv - Effect on β slope (one step QTc model) . . . 131

Sertindole 1 mg/kg iv + atenolol 132
Figure 81 Sertindole 1 mg/kg iv + atenolol . . . 133
Figure 82: Sertindole 1 mg/kg iv + atenolol - Effect on β slope (one step QTc model) . . . 134

Terfenadine 30 mg/kg po 135
Figure 83 Terfenadine 30 mg/kg po . . . 136
Figure 84: Terfenadine 30 mg/kg po - Effect on β slope (one step QTc model) . . . 137

Terfenadine 100 mg/kg po 138
Figure 85 Terfenadine 100 mg/kg po . . . 139
Figure 86: Terfenadine 100 mg/kg po - Effect on β slope (one step QTc model) . . . 140

Thioridazine 1.5 mg/kg po 141
Figure 87 Thioridazine 1.5 mg/kg po . . . 142
Figure 88: Thioridazine 1.5 mg/kg po - Effect on β slope (one step QTc model) . . . 143

Thioridazine 1.5 mg/kg po + atenolol 144
Figure 89 Thioridazine 1.5 mg/kg po + atenolol . . . 145
Figure 90: Thioridazine 1.5 mg/kg po + atenolol - Effect on β slope (one step QTc model) . 146

Thioridazine 5 mg/kg po 147
Figure 91 Thioridazine 5 mg/kg po . . . 148
Figure 92: Thioridazine 5 mg/kg po - Effect on β slope (one step QTc model) . . . 149

Thioridazine 20 mg/kg po 150
Figure 93 Thioridazine 20 mg/kg po . . . 151
Figure 94: Thioridazine 20 mg/kg po - Effect on β slope (one step QTc model) . . . 152

Thioridazine 20 mg/kg po + atenolol 153
Figure 95 Thioridazine 20 mg/kg po + atenolol . . . 154
Figure 96: Thioridazine 20 mg/kg po + atenolol - Effect on β slope (one step QTc model) . . 155

Verapamil 3 mg/kg po **156**

Figure 97 Verapamil 3 mg/kg po 157

Figure 98: Verapamil 3 mg/kg po - Effect on β slope (one step QTc model) 158

Verapamil 10 mg/kg po **159**

Figure 99 Verapamil 10 mg/kg po 160

Figure 100: Verapamil 10 mg/kg po - Effect on β slope (one step QTc model) 161

Verapamil 30 mg/kg po **162**

Figure 101 Verapamil 30 mg/kg po 163

Figure 102: Verapamil 30 mg/kg po - Effect on β slope (one step QTc model) 164

Table 1 Drug information

Molecules	Doses	route	Volume of administration	Suplier	Vehicle
Astemizole	1 mg/kg	iv	1 ml/kg	BioTREND Chemikalien GmbH, Koln, Allemagne	0.9% NaCl
Atenolol	1 mg/kg	iv	1 ml/kg	Sigma Aldrich, Saint Quentin Fallavier, France	0.9% NaCl
Chlorpromazine hydrochloride	1 mg/kg	iv	1 ml/kg	Sigma Aldrich, Saint Quentin Fallavier, France	Purified water
Ciprofloxacin	100 mg/kg	po	5 ml/kg	Sigma Aldrich, Saint Quentin Fallavier, France	Methyl cellulose 0.5%
Cisapride	2 6 mg/kg	po	5 ml/kg	Sequoia Research Products, Pangbourne, United Kingdom	0.6% acid acetic in purified water
Clonidine hydrochloride	0.1 mg/kg	iv	1 ml/kg	Sigma Aldrich, Saint Quentin Fallavier, France	0.9% NaCl
Dofetilide	0.1 mg/kg	po	5 ml/kg	Carbosynth Ltd, Berkshire, United Kingdom	Purified water
Dofetilide	1 mg/kg	po	5 ml/kg	Carbosynth Ltd, Berkshire, United Kingdom	Purified water
Droperidol	3 mg/kg	iv	1 ml/kg	Sigma Aldrich, Saint Quentin Fallavier, France	Water for injectable preparation
Haloperidol 1 3 10 mg/kg	1 3 10 mg/kg	po	5 ml/kg	Sigma Aldrich, Saint Quentin Fallavier, France	0.5% Methylcellulose
Ibutilide fumarate	1 mg/kg	iv	1 ml/kg	CliniSciences, Nanterre, France	Water for injectable preparation
Isoproterenol Hydrochloride	1 mg/kg	po	5 ml/kg	Sigma Aldrich, Saint Quentin Fallavier, France	Purified water
Milrinone	1 3 mg/kg	iv	1 ml/kg	CliniSciences, Nanterre, France	0.9% NaCl
Moxifloxacin	10 30 90 mg/kg	po		Izilox, Bayer	non gastro resistant gelatin capsule
Morphine	2 mg/kg	sc	1 ml/kg		0.9% NaCl
Nicardipine hydrochloride	30 mg/kg	po	5 ml/kg	Sigma Aldrich, Saint Quentin Fallavier, France	Purified water
Phenylephrine	1 mg/kg	po	5 ml/kg	Sigma Aldrich, Saint Quentin Fallavier, France	Purified water
Phenytoin	100 mg/kg	po	5 ml/kg	Sigma Aldrich, Saint Quentin Fallavier, France	0.9% NaCl
Pimozide	1 mg/kg	iv	1 ml/kg	Sigma Aldrich, Saint Quentin Fallavier, France	DMSO 5% in water for injectable Preparation
Prazosin hydrochloride	1 mg/kg	po	10 ml/kg	Abcam Biochemicals, Cambridge, United Kingdom	Water for injectable preparation
Procainamide	10 30 mg/kg	iv	1 ml/kg	Sigma Aldrich, Saint Quentin Fallavier, France	0.9% NaCl
Quinidine anhydrous	3 10 30 mg/kg	po		Sigma Aldrich, Saint Quentin Fallavier, France	non gastro resistant gelatin capsule
Ranolazine	50 mg/kg	iv	1 ml/kg	Sigma Aldrich, Saint Quentin Fallavier, France	0.9% NaCl
Risperidone	1 mg/kg	po		Risperdal, Janssen	non gastro resistant gelatin capsule
Sertindole	1 mg/kg	iv	1 ml/kg	Carbosynth Ltd, Berkshire, United Kingdom	Purified water
Sotalol chlorhydrate	3 10 30 mg/kg	po	5 ml/kg	Sotalol TEVA	Purified water
Terfenadine	30 100 mg/kg	po	5 ml/kg	Sigma Aldrich, Saint Quentin Fallavier, France	1% DMSO + 0.5% methylcellulose
Thioridazine hydrochloride	1.5 5 20 mg/kg	po	5 ml/kg	Sigma Aldrich, Saint Quentin Fallavier, France	Purified water
Verapamil hydrochloride	3 10 30 mg/kg	po	5 ml/kg	Sigma Aldrich, Saint Quentin Fallavier, France	Purified water

Comparative summary tables

Table 2 OS QTc vs conventional QTc Maximum UB over the first 6 hours - dQTc n=4

Treatment	HR	LogQTRR	LinQTHR	OS LinQTHR
Nicardipine 30 mg/kg po	73.5	(-24.6;-9.6) [4]	(0;10.1) [1]	(-20.7;-14.8) [6]
Milrinone 3 mg/kg iv	20.4	(-7.3;6.7) [6]	(-6.8;4.8) [6]	(-7.3;-3.7) [6]
Milrinone 1 mg/kg iv	9.3	(-9.5;4.2) [6]	(-10.3;3.8) [6]	(-6.2;-1.6) [6]
Phenytoin 100 mg/kg po	0.5	(-4.2;3.8) [1]	(-4;3.3) [1]	(-4.3;-1.4) [2]
Verapamil 30 mg/kg po	10.6	(-15.5;0.6) [1]	(-2.7;-0.9) [1]	(-4.9;0.5) [2]
Verapamil 10 mg/kg po	5.3	(-3.1;-1) [6]	(-3.2;-1) [6]	(-2.9;-0.7) [6]
Prazosin 10 mg/kg po	17.5	(-1.8;5.9) [6]	(-3.5;3.9) [6]	(-2.4;0.7) [1]
Ranolazine 50 mg/kg po	4.0	(-2.1;4.7) [5]	(-1.5;3.2) [1]	(-0.9;2.6) [5]
Verapamil 3 mg/kg po	-1.4	(-3.4;6.8) [3]	(-3.4;9) [3]	(0.3;3.3) [3]
Ebastine 30 mg/kg po	0.3	(0.5;7.4) [1]	(0.2;9.3) [1]	(1.3;4.2) [1]
Cisapride 2 mg/kg po	0.5	(1.1;4.3) [5]	(1;3) [5]	(2.2;5.9) [3]
Phenylephrine 1 mg/kg po	-10.2	(-3.7;8.2) [3]	(-3;9.1) [3]	(2.6;5.9) [4]
Atenolol 1 mg/kg iv	-12.5	(-5.9;7) [3]	(-6.1;7.6) [3]	(1.6;7) [6]
Nicardipine 3 mg/kg po	0.9	(4.1;11.2) [2]	(2.8;7.2) [6]	(3.9;6.3) [6]
Isoprenaline 1 mg/kg po	26.9	(4.1;12.5) [1]	(7.2;17.3) [2]	(4.2;6.4) [2]
Ciprofloxacin 100 mg/kg po	0.6	(2.3;8) [3]	(1.8;8.1) [3]	(4.9;7.5) [2]
Procainamide 10 mg/kg iv	-3.9	(0.5;14.4) [1]	(1.3;13.8) [1]	(5.5;8.3) [1]
Quinidine 3 mg/kg po	-7.9	(3.3;10) [4]	(4;8.5) [4]	(5.7;8.5) [4]
Risperidone 1 mg/kg iv + atenolol	0.2	(2.6;14.3) [4]	(2.5;13.8) [3]	(6.4;9.1) [4]
Thioridazine 1.5 mg/kg po	5.2	(2.3;15.4) [4]	(1.9;15) [4]	(5.6;10.1) [3]
Chlorpromazine 1 mg/kg iv	11.1	(2.8;11) [2]	(3;12.4) [2]	(6.6;9.9) [1]
Risperidone 1 mg/kg iv	19.5	(2.4;14.7) [2]	(2.2;11.9) [5]	(7.2;11.1) [6]
Quinidine 10 mg/kg po	-10.9	(2.1;17.6) [6]	(4.2;16.6) [6]	(8.9;11.7) [6]
Moxifloxacin 10 mg/kg po	-2.7	(2;17.6) [3]	(1.9;17.7) [3]	(8.1;13.2) [5]
Thioridazine 1.5 mg/kg po + atenolol	-6.2	(6.3;15.3) [4]	(7.7;15) [4]	(10.6;14.5) [3]
Sertindole 1 mg/kg iv	16.8	(3.7;17.4) [1]	(2.3;17.9) [1]	(10.8;15.4) [1]
Sotalol 3 mg/kg po	-8.8	(8.7;12.1) [2]	(8.8;13.9) [2]	(11.5;14.9) [3]
Thioridazine 5 mg/kg po	14.7	(4.8;7.1) [2]	(5;7.6) [2]	(12;15.7) [2]
Procainamide 30 mg/kg iv	10.2	(12.4;20.3) [1]	(12.8;20.5) [1]	(12.9;15.6) [1]
Clonidine 0.1 mg/kg iv	-25.1	(4;6.7) [4]	(5.5;16.7) [4]	(14.9;18.8) [3]
Cisapride 6 mg/kg po	9.9	(6;10.6) [6]	(4.9;16.2) [2]	(14.5;24.3) [3]
Ibutilide 1 mg/kg iv	-3.7	(10;20.6) [4]	(10.9;26.8) [2]	(18.5;22.6) [2]
Quinidine 30 mg/kg po	8.5	(11;29.8) [4]	(10.5;28.6) [4]	(19.5;22.3) [4]
Terfenadine 30 mg/kg po	3.0	(17.2;27.2) [20]	(15.6;29) [20]	(22.1;26.4) [20]
Thioridazine 20 mg/kg po	21.9	(11.5;27.9) [2]	(14.1;24.8) [2]	(20.5;28.5) [1]
Haloperidol 1 mg/kg po	1.6	(0;10.8) [2]	(1.9;10.1) [2]	(19.8;32.5) [5]
Sotalol 10 mg/kg po	-12.9	(12.1;29.2) [5]	(14.1;32.3) [5]	(24.5;30.7) [3]
Morphine 2 mg/kg sc	-32.1	(13.9;28.2) [2]	(21.7;31.8) [2]	(27.3;31.2) [2]
Moxifloxacin 30 mg/kg po	-7.8	(21.7;28.9) [6]	(23.9;29.6) [6]	(26.4;32.6) [5]
Droperidol 3 mg/kg iv	2.8	(23.4;31.6) [1]	(22;29.7) [1]	(28.7;33.2) [1]
Thioridazine 20 mg/kg po + atenolol	1.7	(22.8;34) [2]	(23;30.9) [2]	(28.8;34.7) [2]
Haloperidol 10 mg/kg po	6.5	(2.7;20) [1]	(1.8;18.9) [1]	(26.1;38.1) [2]
Pimozide 1 mg/kg iv	16.2	(10.1;31.2) [2]	(11.8;26.5) [2]	(30.6;33.9) [2]
Terfenadine 100 mg/kg po	-3.5	(16.6;36.9) [23]	(16;34.7) [23]	(32.1;36.6) [23]
Sotalol 30 mg/kg po	-4.2	(30.3;31.4) [6]	(30.3;43.7) [5]	(33.9;36.7) [5]
Astemizole 1 mg/kg iv	5.8	(27.5;38.3) [2]	(24.6;34.6) [2]	(32.7;38) [1]
Sertindole 1 mg/kg iv + atenolol	-4.5	(23.7;37.2) [1]	(22.2;35.4) [1]	(35.5;39.5) [1]
Moxifloxacin 90 mg/kg po	-8.9	(23.4;52.7) [5]	(22.8;52.3) [5]	(37.2;43.7) [6]
Dofetilide 0.1 mg/kg po	4.0	(34.4;39.1) [5]	(33;38.5) [5]	(40.4;43.9) [2]
Dofetilide 1 mg/kg po	1.4	(42.3;70.4) [3]	(40.3;67.1) [3]	(65.6;68.2) [4]
Haloperidol 3 mg/kg po	28.4	(19.1;28.3) [5]	(16.7;30.2) [5]	(68;128.4) [5]

negative
positive

Table 3 OS QTc vs conventional QTc Maximum UB over the first 6 hours - ddQTc n=4

Treatment	HR	LogQTRR	LinQTHR	OS LinQTHR
Nicardipine 30 mg/kg po	73.5	(-21.8;1.1) [6]	(-0.5;17.4) [1]	(-18.7;-13.6) [6]
Milrinone 3 mg/kg iv	20.4	(-5.8;4.7) [6]	(-5;1.7) [6]	(-8.8;-3.6) [6]
Milrinone 1 mg/kg iv	9.3	(-10.8;6.6) [6]	(-10.8;5.2) [6]	(-5.7;0.4) [6]
Atenolol 1 mg/kg iv	-12.5	(-9.6;-2.4) [2]	(-8.7;-2.1) [2]	(-4.3;0.3) [6]
Phenytoin 100 mg/kg po	0.5	(-6.3;2.6) [1]	(-6.2;2.5) [1]	(-2.9;1.2) [2]
Verapamil 10 mg/kg po	5.3	(-5.2;0.8) [6]	(-3.3;-1.9) [1]	(-2.6;1.1) [6]
Ebastine 30 mg/kg po	0.3	(-5.4;-0.3) [1]	(-5.4;1.2) [1]	(-2.4;3.1) [1]
Verapamil 30 mg/kg po	10.6	(-8.9;4.7) [1]	(2.3;3.2) [1]	(0;6.1) [2]
Verapamil 3 mg/kg po	-1.4	(-3.2;10.8) [3]	(-1.2;3.5) [1]	(0.9;5.7) [3]
Ranolazine 50 mg/kg po	4.0	(-0.9;8.2) [5]	(-1.3;8) [5]	(1.5;5.4) [5]
Ciprofloxacin 100 mg/kg po	0.6	(-0.5;10.7) [4]	(0.7;9.9) [4]	(1.4;6.1) [2]
Prazosin 10 mg/kg po	17.5	(2.7;11.1) [6]	(3.3;7) [2]	(1.7;6) [1]
Phenylephrine 1 mg/kg po	-10.2	(-4.2;16) [5]	(-3.2;16.5) [5]	(2.7;6.8) [4]
Nicardipine 3 mg/kg po	0.9	(-0.1;14.5) [2]	(0.7;8.2) [6]	(3.9;7.1) [6]
Isoprenaline 1 mg/kg po	26.9	(4.5;13.3) [1]	(7.1;17.4) [2]	(4;7) [2]
Cisapride 2 mg/kg po	0.5	(2.5;9.7) [6]	(2.5;8.8) [6]	(4.1;7.1) [3]
Procainamide 10 mg/kg iv	-3.9	(3.8;11) [1]	(2.8;11.9) [1]	(4;8.3) [1]
Quinidine 3 mg/kg po	-7.9	(0;15) [3]	(0.1;18.1) [4]	(6.4;11.2) [4]
Chlorpromazine 1 mg/kg iv	11.1	(0.6;16.6) [2]	(1.4;17.3) [2]	(7;11.3) [1]
Moxifloxacin 10 mg/kg po	-2.7	(-0.4;4.1) [1]	(0.3;18.3) [3]	(6.3;12.3) [5]
Quinidine 10 mg/kg po	-10.9	(2.4;15.5) [2]	(1;16.1) [2]	(7;12) [6]
Thioridazine 1.5 mg/kg po	5.2	(4.3;11.7) [3]	(1.6;13.4) [3]	(6.9;12.3) [3]
Risperidone 1 mg/kg iv + atenolol	0.2	(5.3;12.5) [6]	(6.7;10.8) [6]	(10.3;14.8) [4]
Risperidone 1 mg/kg iv	19.5	(7.8;17.9) [2]	(7.8;16.5) [2]	(9.8;15.6) [6]
Procainamide 30 mg/kg iv	10.2	(12.1;19.1) [1]	(11.6;19.2) [1]	(12;15) [1]
Thioridazine 1.5 mg/kg po + atenolol	-6.2	(2.4;18.3) [3]	(1.7;19.6) [3]	(10.6;16.7) [3]
Thioridazine 5 mg/kg po	14.7	(2.6;16.3) [2]	(3;16.2) [2]	(10;17.6) [2]
Sotalol 3 mg/kg po	-8.8	(6.8;20.3) [3]	(8.1;19.1) [3]	(12.5;17) [3]
Sertindole 1 mg/kg iv	16.8	(9.9;18.7) [1]	(8.2;18.3) [1]	(13.9;19.7) [1]
Clonidine 0.1 mg/kg iv	-25.1	(5.2;10.3) [4]	(7.9;18.6) [4]	(15.9;19.8) [3]
Cisapride 6 mg/kg po	9.9	(4;15.9) [2]	(4.6;14.1) [2]	(14.7;21.7) [3]
Ibutilide 1 mg/kg iv	-3.7	(10.9;28.1) [2]	(11.4;27) [2]	(16.8;21.7) [2]
Quinidine 30 mg/kg po	8.5	(12.9;24.3) [4]	(13.2;23.5) [4]	(19.4;23.1) [4]
Thioridazine 20 mg/kg po	21.9	(3.6;22.8) [1]	(6.2;34.6) [2]	(21.1;25.7) [1]
Sotalol 10 mg/kg po	-12.9	(12.2;21.9) [3]	(14;31.3) [5]	(23.5;28.2) [3]
Terfenadine 100 mg/kg po	-3.5	(10.5;32.6) [23]	(10.4;28.3) [23]	(24.4;29.9) [23]
Astemizole 1 mg/kg iv	5.8	(22.8;32.5) [2]	(21.1;31.9) [2]	(25.8;31.5) [1]
Terfenadine 30 mg/kg po	3.0	(17.6;33.5) [20]	(19;32.7) [20]	(27.6;32.9) [20]
Moxifloxacin 30 mg/kg po	-7.8	(22;31.1) [6]	(22.7;32.8) [6]	(27.9;34) [5]
Thioridazine 20 mg/kg po + atenolol	1.7	(18;37.2) [2]	(16.7;30.7) [3]	(28.5;33.9) [2]
Haloperidol 1 mg/kg po	1.6	(5.9;16.1) [4]	(4.9;15.4) [4]	(21.5;45.5) [5]
Morphine 2 mg/kg sc	-32.1	(16.8;31.5) [2]	(20.9;38.3) [2]	(32.5;38.3) [2]
Pimozide 1 mg/kg iv	16.2	(12.8;34.3) [2]	(15.3;20.9) [1]	(34;37.9) [2]
Sertindole 1 mg/kg iv + atenolol	-4.5	(21.7;36.3) [2]	(19.9;34.6) [2]	(34.2;39.6) [1]
Haloperidol 10 mg/kg po	6.5	(11.6;26.1) [3]	(10.9;26.7) [3]	(25.7;49.5) [2]
Droperidol 3 mg/kg iv	2.8	(29.5;35.8) [2]	(26.2;36.1) [1]	(35.4;40.6) [1]
Sotalol 30 mg/kg po	-4.2	(32.6;47.6) [5]	(32.3;46.3) [5]	(35.9;42) [5]
Moxifloxacin 90 mg/kg po	-8.9	(28.1;47.7) [5]	(24;48.3) [5]	(36.2;41.9) [6]
Dofetilide 0.1 mg/kg po	4.0	(37.9;46.4) [2]	(36.5;42.5) [2]	(42.8;46.8) [2]
Dofetilide 1 mg/kg po	1.4	(38.1;63.2) [2]	(35.5;60.7) [2]	(63.3;67.7) [4]
Haloperidol 3 mg/kg po	28.4	(13.3;36.2) [5]	(12.2;20.6) [3]	(62.7;120.9) [5]

negative
positive

Astemizole 1 mg/kg iv

Figure 1 Astemizole 1 mg/kg iv

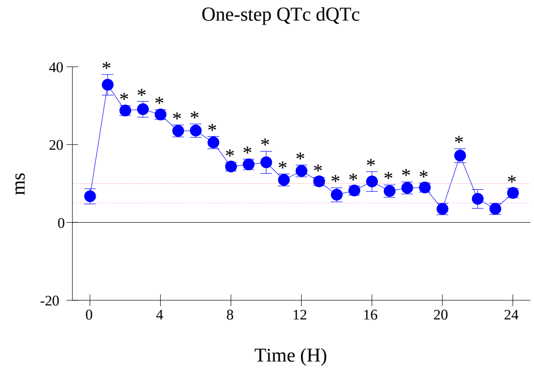

*: P<=0.05: one-sided 95% CI lower bound =>5ms
LSD= 1.5 ms

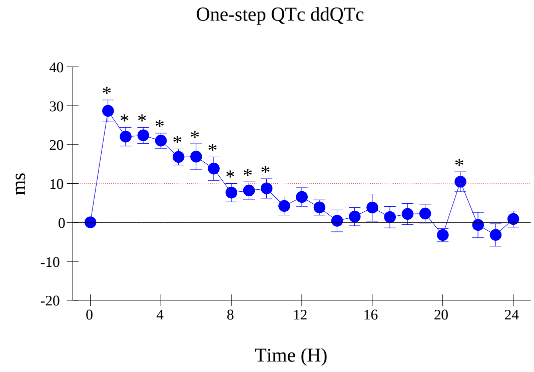

*: P<=0.05: one-sided 95% CI lower bound =>5ms
LSD= 2.4 ms

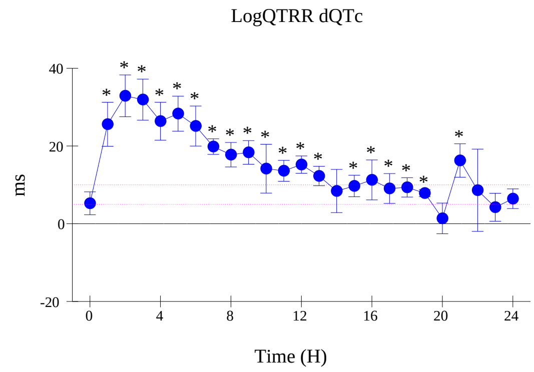

*: P<=0.05: one-sided 95% CI lower bound =>5ms
LSD= 3.8 ms

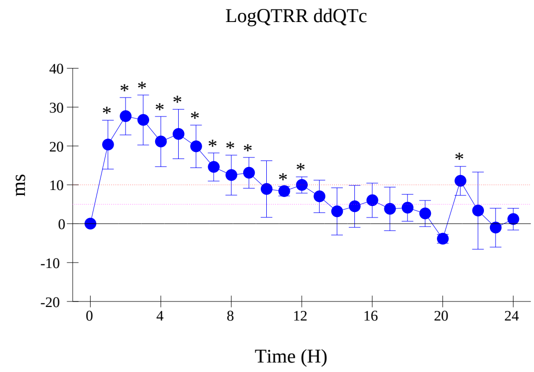

*: P<=0.05: one-sided 95% CI lower bound =>5ms
LSD= 4.8 ms

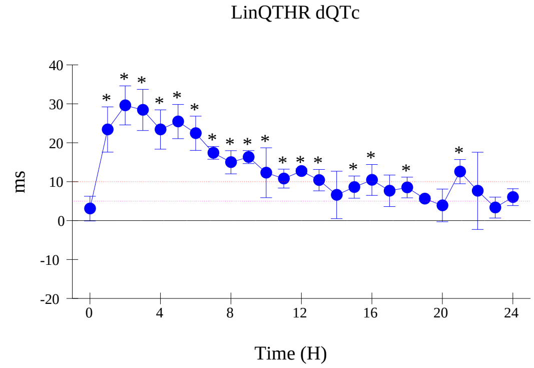

*: P<=0.05: one-sided 95% CI lower bound =>5ms
LSD= 3.2 ms

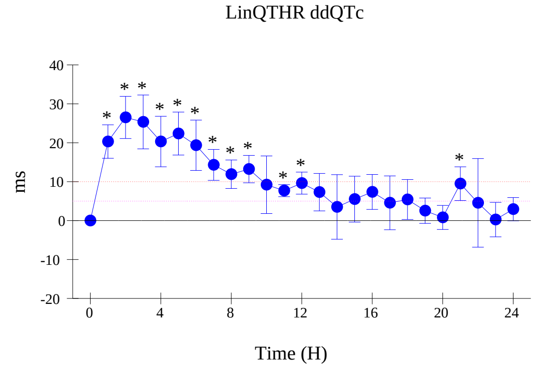

*: P<=0.05: one-sided 95% CI lower bound =>5ms
LSD= 4.5 ms

Figure 2 Astemizole 1 mg/kg iv - Effect on β slope (one step QTc model)

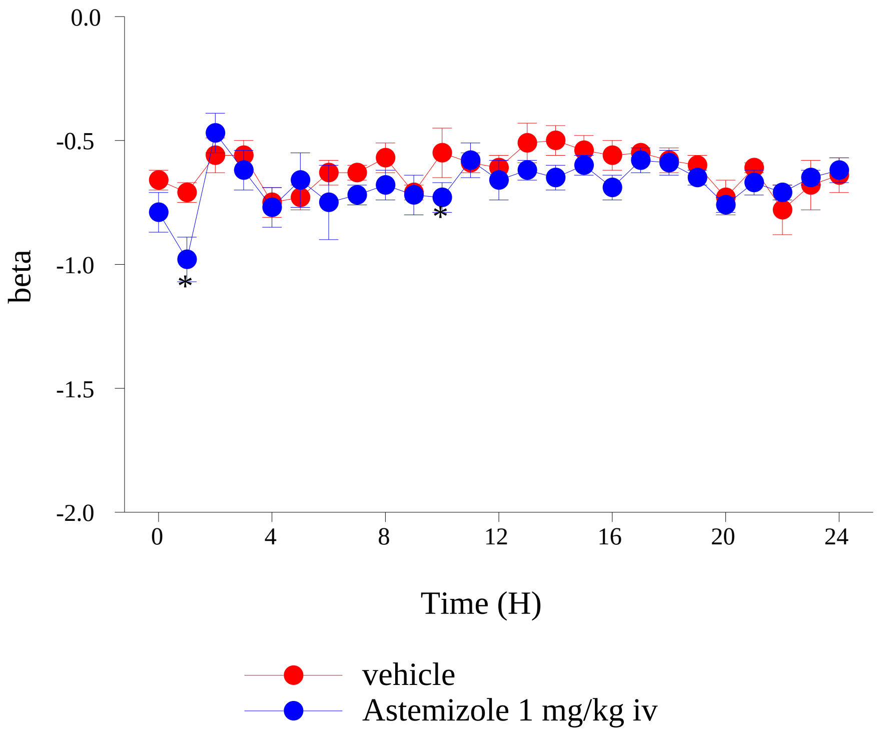

Results expressed in β
Repeated measures analysis of variance (RMANOVA)
Probability for Treatment factor: P=0.079
Probability for Time X Treatment interaction: P=0.602
$\star$: P$\leq$0.05 (LSD)
LSD=0.2 - Least significant difference for α type-1 error=5%
MDD=0.2 - Minimum detectable difference for α type-1 error=5% and β type-2 error=20% (*i.e.*power=80%)
Electronic authentication: created by Pascal Champéroux on 11-FEV-2025 at 14:47:35.052
Study QTOS

Atenolol 1 mg/kg iv

Figure 3 Atenolol 1 mg/kg iv

One-step QTc dQTc

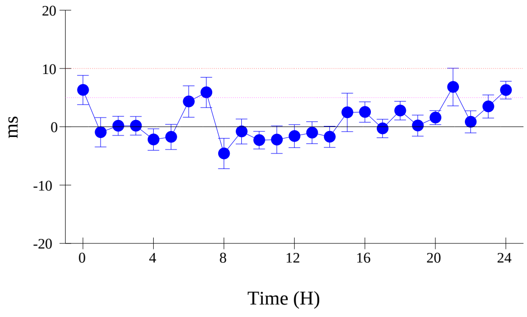

*: P<=0.05: one-sided 95% CI lower bound =>5ms
LSD= 1.9 ms

One-step QTc ddQTc

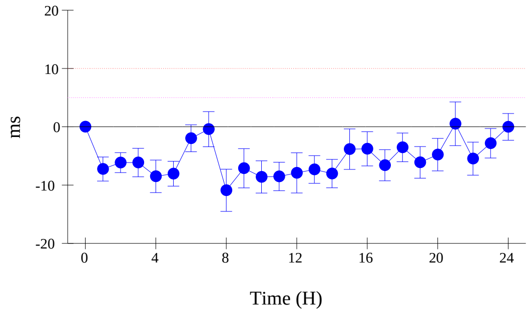

*: P<=0.05: one-sided 95% CI lower bound =>5ms
LSD= 2.7 ms

LogQTRR dQTc

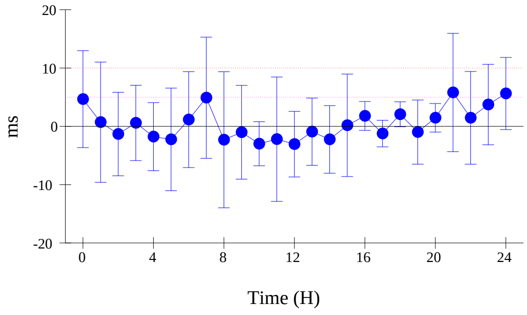

*: P<=0.05: one-sided 95% CI lower bound =>5ms
LSD= 6.9 ms

LogQTRR ddQTc

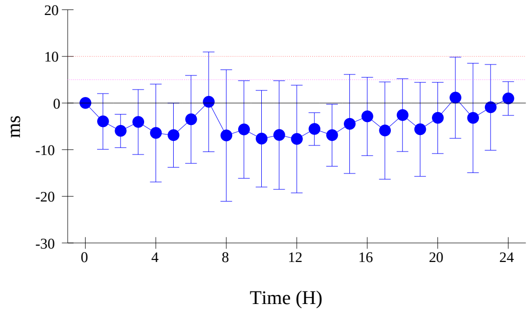

*: P<=0.05: one-sided 95% CI lower bound =>5ms
LSD= 9.2 ms

LinQTHR dQTc

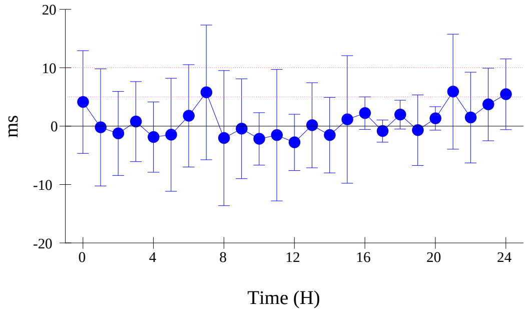

*: P<=0.05: one-sided 95% CI lower bound =>5ms
LSD= 7.2 ms

LinQTHR ddQTc

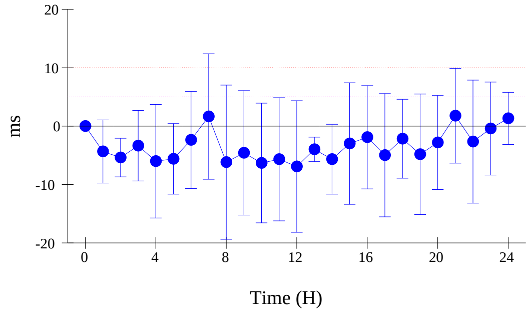

*: P<=0.05: one-sided 95% CI lower bound =>5ms
LSD= 8.3 ms

Figure 4 Atenolol 1 mg/kg iv - Effect on β slope (one step QTc model)

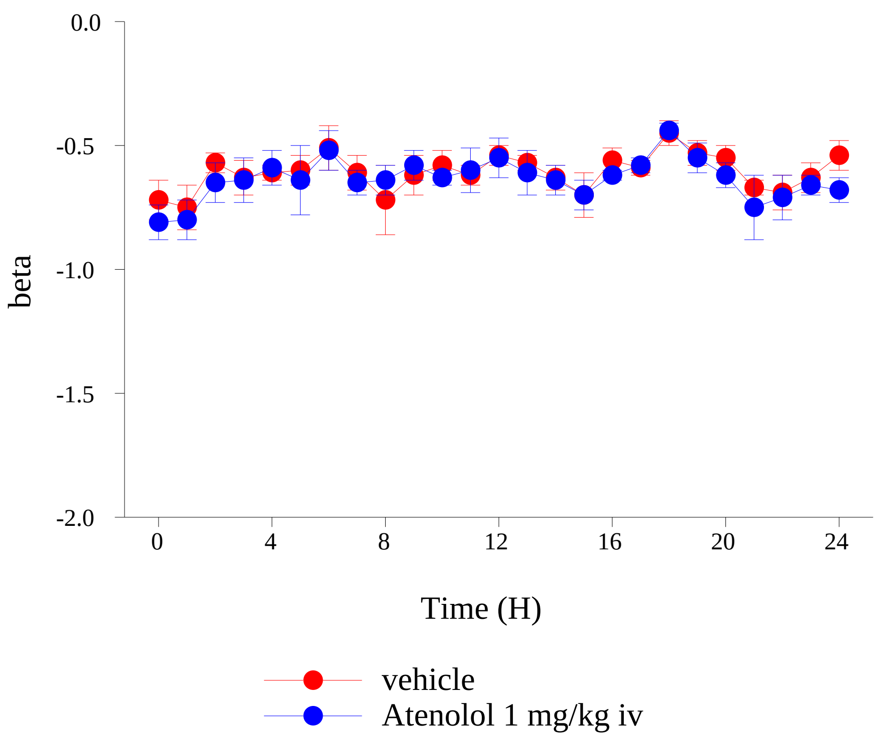

Results expressed in β
Repeated measures analysis of variance (RMANOVA)
Probability for Treatment factor: P=0.281
Probability for Time X Treatment interaction: P=1
$\star$: P$\leq$0.05 (LSD)
LSD=0.2 - Least significant difference for α type-1 error=5%
MDD=0.3 - Minimum detectable difference for α type-1 error=5% and β type-2 error=20% (*i.e.*power=80%)
Electronic authentication: created by Pascal Champéroux on 11-FEV-2025 at 14:47:35.179
Study QTOS

Ciprofloxacin 100 mg/kg po

Figure 5 Ciprofloxacin 100 mg/kg po

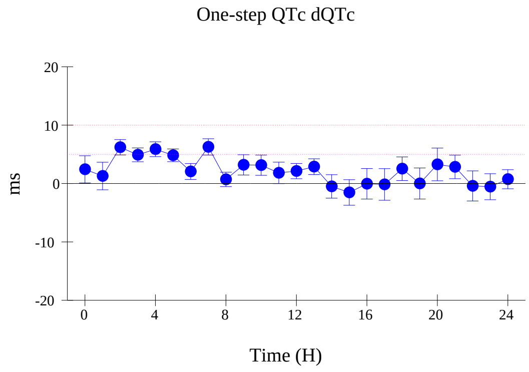

*: P<=0.05: one-sided 95% CI lower bound =>5ms
LSD= 1.8 ms

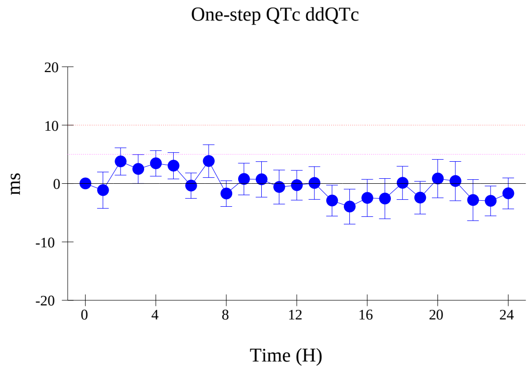

*: P<=0.05: one-sided 95% CI lower bound =>5ms
LSD= 2.8 ms

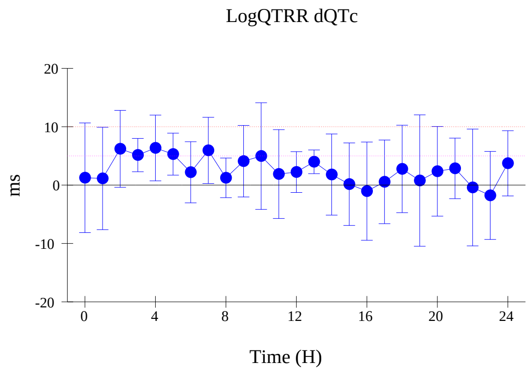

*: P<=0.05: one-sided 95% CI lower bound =>5ms
LSD= 7 ms

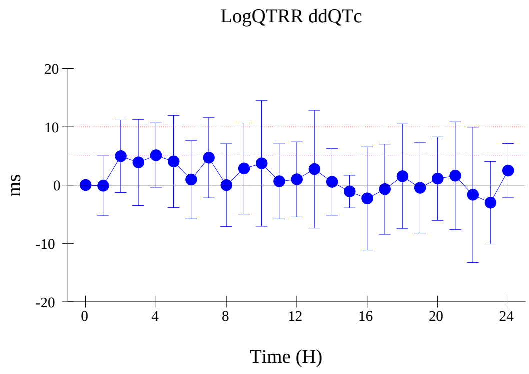

*: P<=0.05: one-sided 95% CI lower bound =>5ms
LSD= 7.1 ms

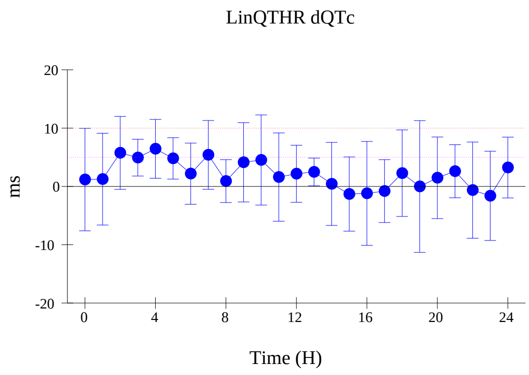

*: P<=0.05: one-sided 95% CI lower bound =>5ms
LSD= 6.4 ms

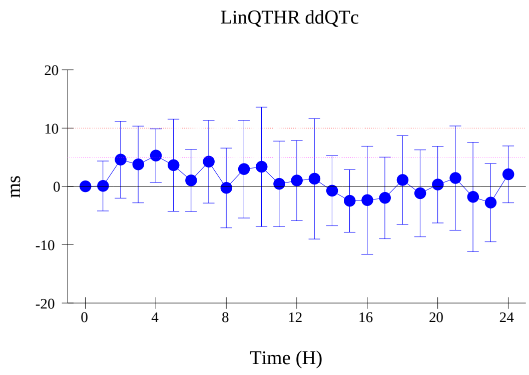

*: P<=0.05: one-sided 95% CI lower bound =>5ms
LSD= 6.9 ms

Figure 6 Ciprofloxacin 100 mg/kg po - Effect on β slope (one step QTc model)

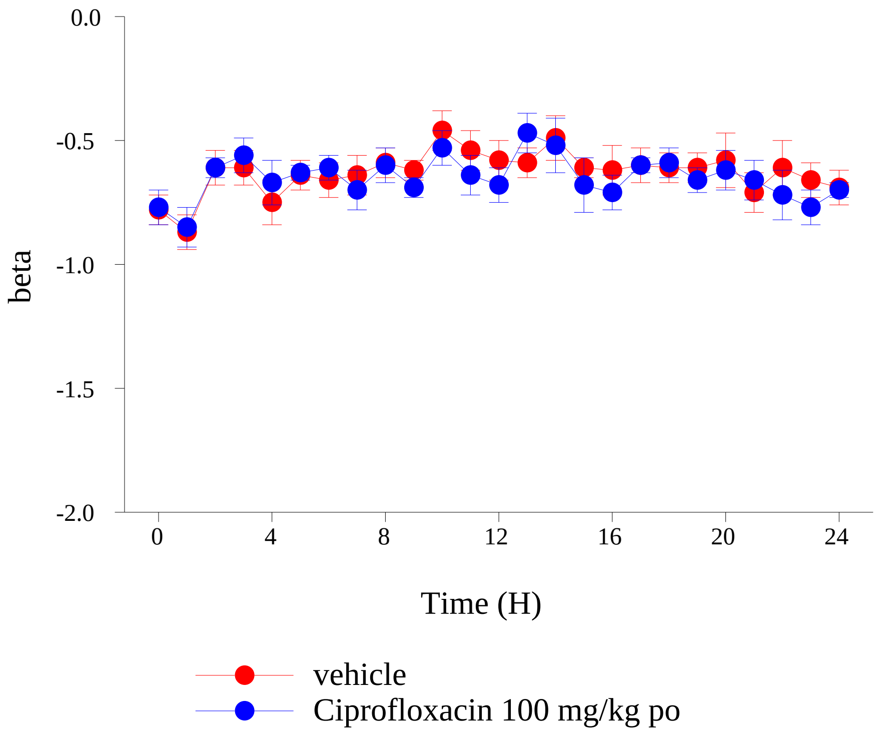

Results expressed in β
Repeated measures analysis of variance (RMANOVA)
Probability for Treatment factor: P=0.785
Probability for Time X Treatment interaction: P=0.87
$\star$: P$\leq$0.05 (LSD)
LSD=0.2 - Least significant difference for α type-1 error=5%
MDD=0.2 - Minimum detectable difference for α type-1 error=5% and β type-2 error=20% (*i.e.*power=80%)
Electronic authentication: created by Pascal Champéroux on 11-FEV-2025 at 14:47:35.291
Study QTOS

Cisapride 2 mg/kg po

Figure 7 Cisapride 2 mg/kg po

One-step QTc dQTc

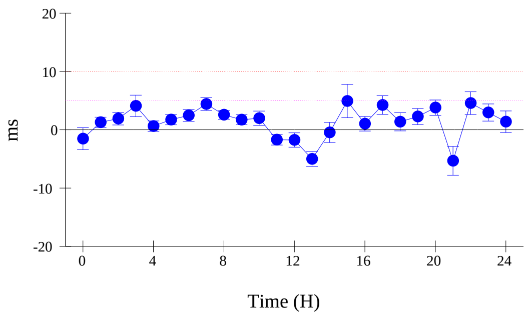

*: P<=0.05: one-sided 95% CI lower bound =>5ms
LSD= 1.3 ms

One-step QTc ddQTc

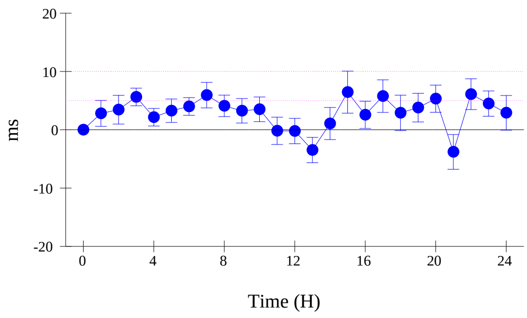

*: P<=0.05: one-sided 95% CI lower bound =>5ms
LSD= 2.2 ms

LogQTRR dQTc

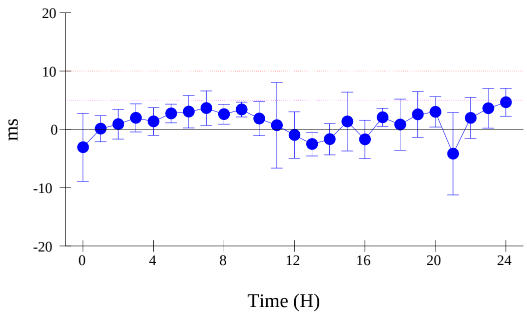

*: P<=0.05: one-sided 95% CI lower bound =>5ms
LSD= 2.8 ms

LogQTRR ddQTc

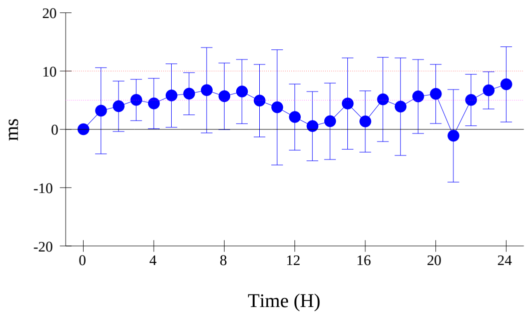

*: P<=0.05: one-sided 95% CI lower bound =>5ms
LSD= 5.7 ms

LinQTHR dQTc

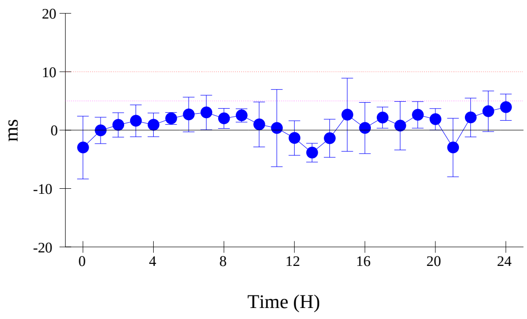

*: P<=0.05: one-sided 95% CI lower bound =>5ms
LSD= 3 ms

LinQTHR ddQTc

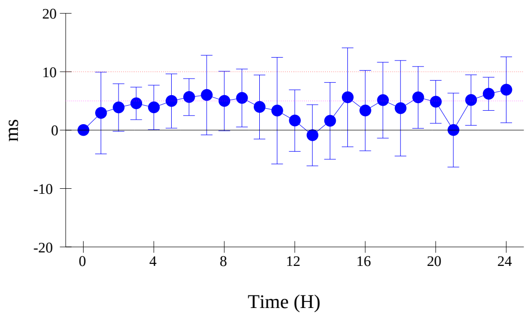

*: P<=0.05: one-sided 95% CI lower bound =>5ms
LSD= 5.3 ms

Figure 8 Cisapride 2 mg/kg po - Effect on β slope (one step QTc model)

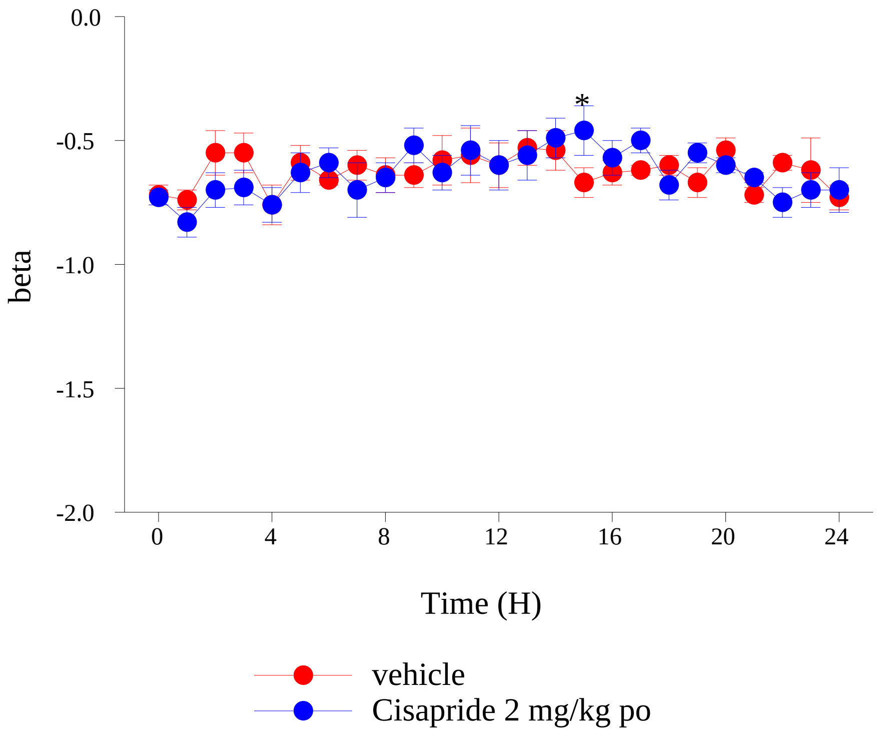

Results expressed in β
Repeated measures analysis of variance (RMANOVA)
Probability for Treatment factor: P=0.943
Probability for Time X Treatment interaction: P=0.161
$\star$: P$\leq$0.05 (LSD)
LSD=0.2 - Least significant difference for α type-1 error=5%
MDD=0.2 - Minimum detectable difference for α type-1 error=5% and β type-2 error=20% (*i.e.*power=80%)
Electronic authentication: created by Pascal Champéroux on 11-FEV-2025 at 14:47:35.402
Study QTOS

Cisapride 6 mg/kg po

Figure 9 Cisapride 6 mg/kg po

One-step QTc dQTc

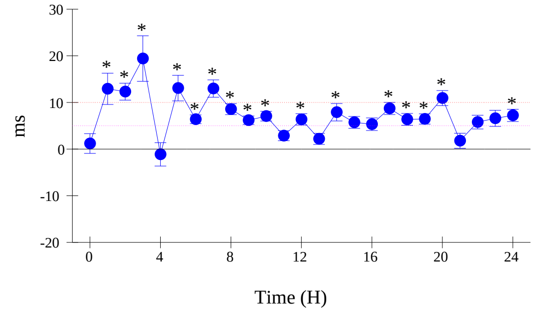

*: P<=0.05: one-sided 95% CI lower bound =>5ms
LSD= 1.3 ms

One-step QTc ddQTc

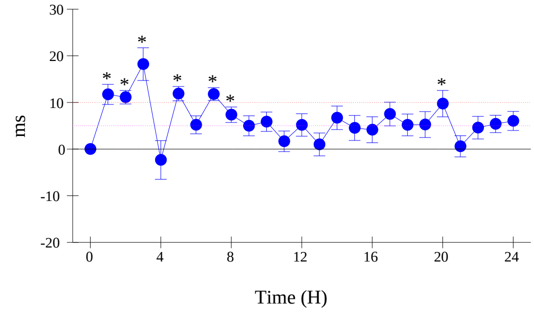

*: P<=0.05: one-sided 95% CI lower bound =>5ms
LSD= 2.3 ms

LogQTRR dQTc

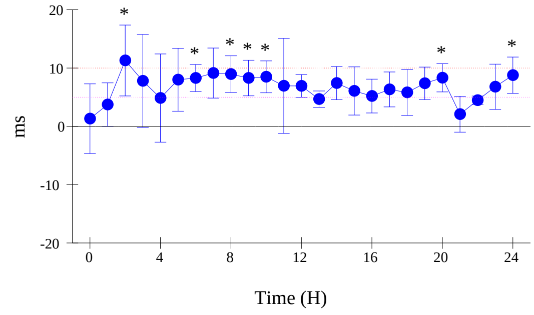

*: P<=0.05: one-sided 95% CI lower bound =>5ms
LSD= 3.1 ms

LogQTRR ddQTc

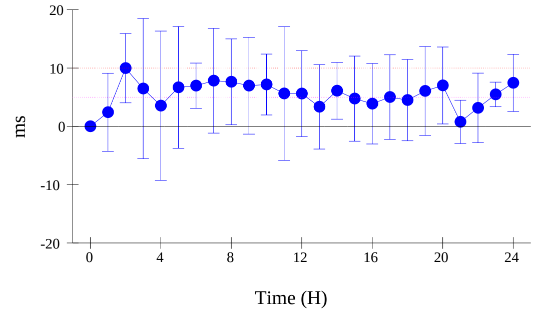

*: P<=0.05: one-sided 95% CI lower bound =>5ms
LSD= 7 ms

LinQTHR dQTc

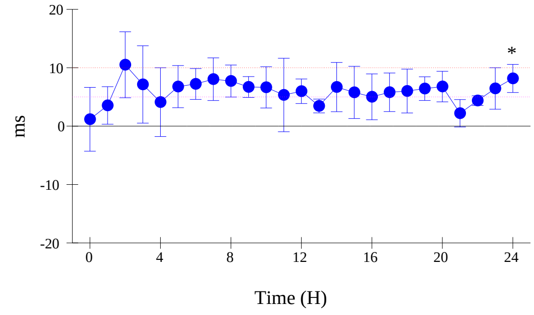

*: P<=0.05: one-sided 95% CI lower bound =>5ms
LSD= 3.5 ms

LinQTHR ddQTc

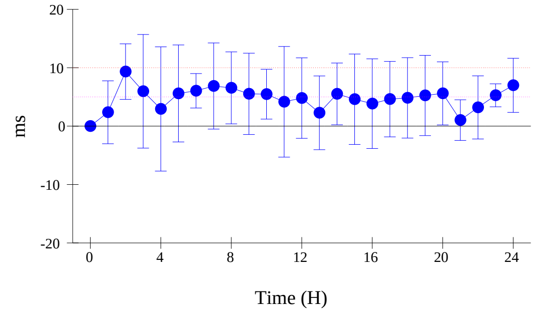

*: P<=0.05: one-sided 95% CI lower bound =>5ms
LSD= 6.3 ms

Figure 10 Cisapride 6 mg/kg po - Effect on β slope (one step QTc model)

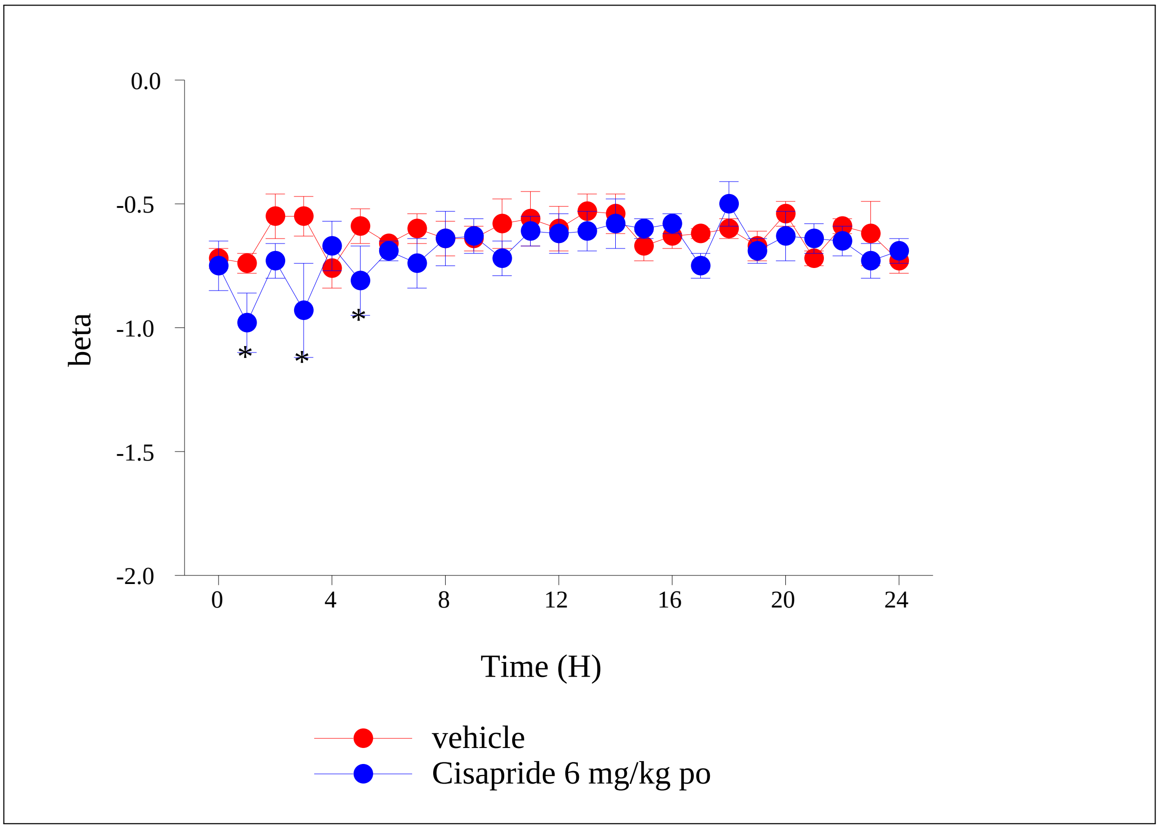

Results expressed in β
Repeated measures analysis of variance (RMANOVA)
Probability for Treatment factor: P=0.365
Probability for Time X Treatment interaction: P=0.095
$\star$: P$\leq$0.05 (LSD)
LSD=0.2 - Least significant difference for α type-1 error=5%
MDD=0.3 - Minimum detectable difference for α type-1 error=5% and β type-2 error=20% (*i.e.*power=80%)
Electronic authentication: created by Pascal Champéroux on 11-FEV-2025 at 14:47:35.529
Study QTOS

Clonidine 0.1 mg/kg iv

Figure 11 Clonidine 0.1 mg/kg iv

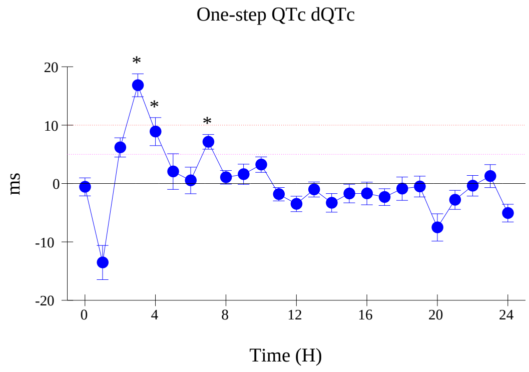

*: P<=0.05: one-sided 95% CI lower bound =>5ms
LSD= 1.6 ms

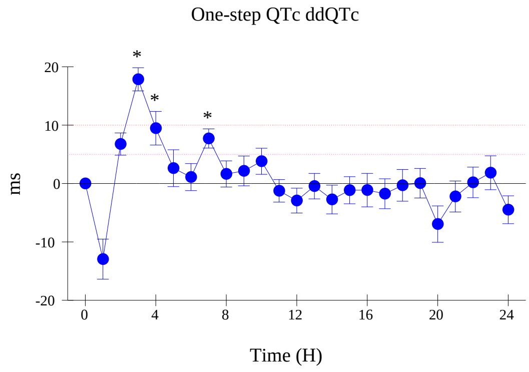

*: P<=0.05: one-sided 95% CI lower bound =>5ms
LSD= 2.5 ms

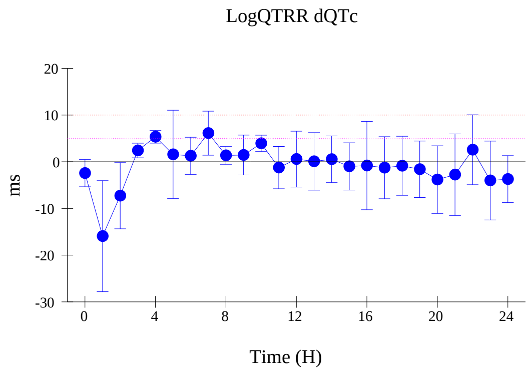

*: P<=0.05: one-sided 95% CI lower bound =>5ms
LSD= 6 ms

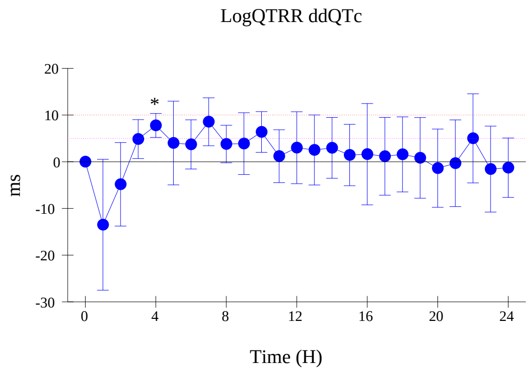

*: P<=0.05: one-sided 95% CI lower bound =>5ms
LSD= 7.5 ms

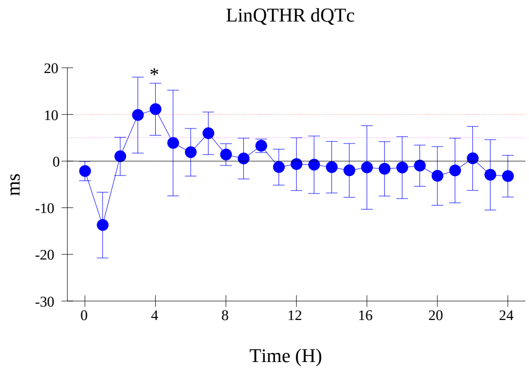

*: P<=0.05: one-sided 95% CI lower bound =>5ms
LSD= 5.7 ms

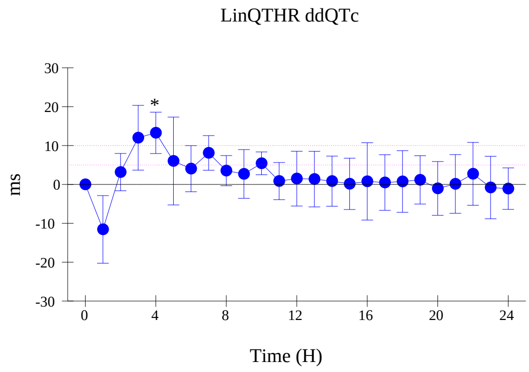

*: P<=0.05: one-sided 95% CI lower bound =>5ms
LSD= 6.6 ms

Figure 12 Clonidine 0.1 mg/kg iv - Effect on β slope (one step QTc model)

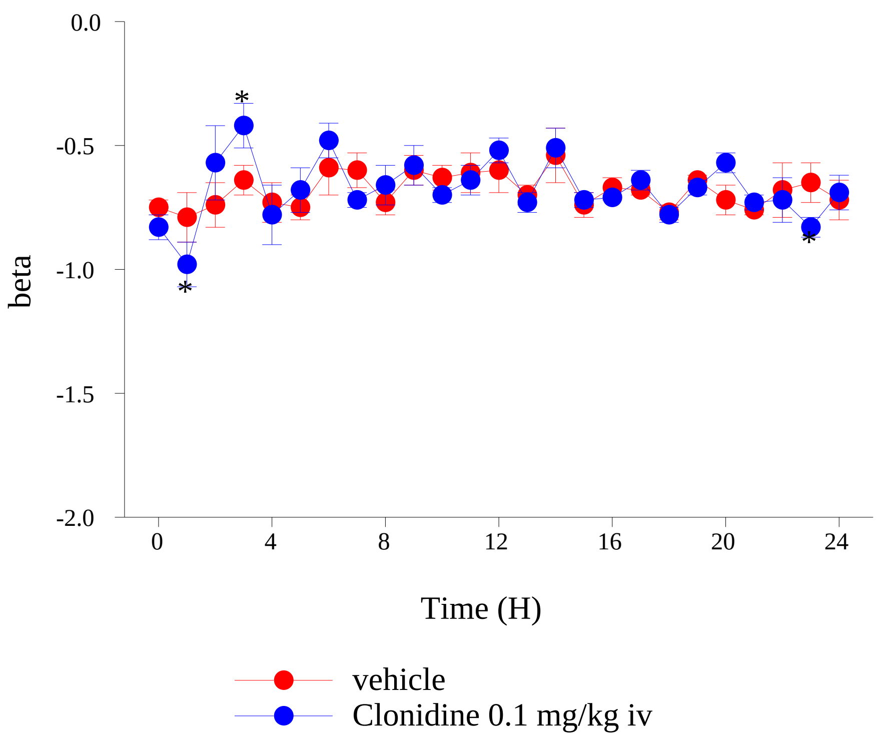

Results expressed in β
Repeated measures analysis of variance (RMANOVA)
Probability for Treatment factor: P=0.883
Probability for Time X Treatment interaction: P=0.215
$\star$: P$\leq$0.05 (LSD)
LSD=0.2 - Least significant difference for α type-1 error=5%
MDD=0.2 - Minimum detectable difference for α type-1 error=5% and β type-2 error=20% (*i.e.*power=80%)
Electronic authentication: created by Pascal Champéroux on 11-FEV-2025 at 14:47:35.896
Study QTOS

Chlorpromazine 1 mg/kg iv

Figure 13 Chlorpromazine 1 mg/kg iv

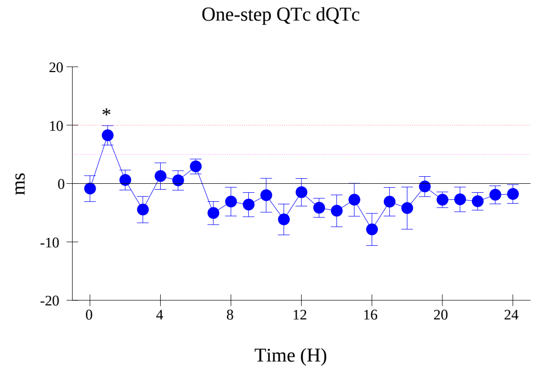

*: P<=0.05: one-sided 95% CI lower bound =>5ms
LSD= 2.1 ms

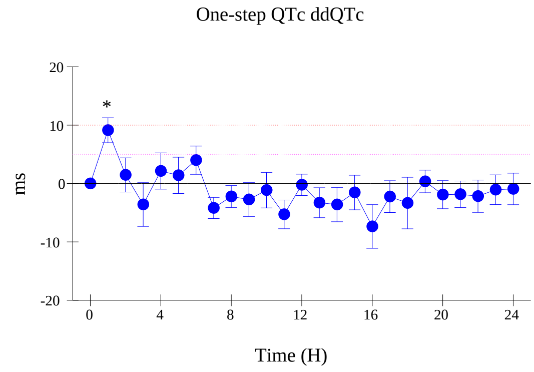

*: P<=0.05: one-sided 95% CI lower bound =>5ms
LSD= 2.7 ms

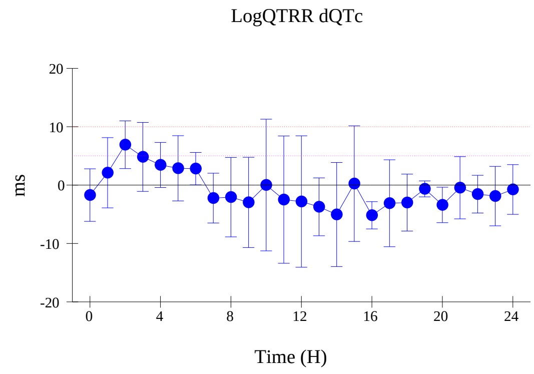

*: P<=0.05: one-sided 95% CI lower bound =>5ms
LSD= 5.1 ms

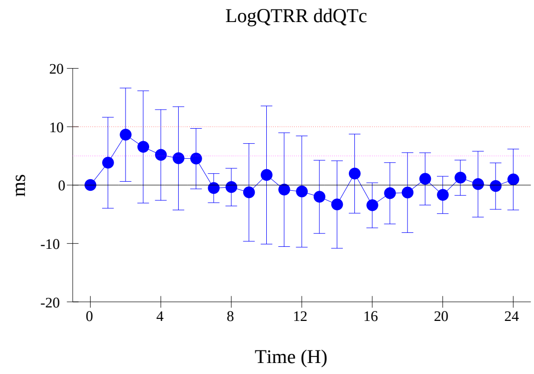

*: P<=0.05: one-sided 95% CI lower bound =>5ms
LSD= 6.3 ms

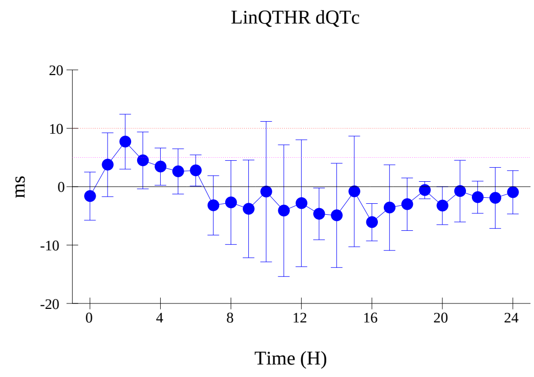

*: P<=0.05: one-sided 95% CI lower bound =>5ms
LSD= 4.9 ms

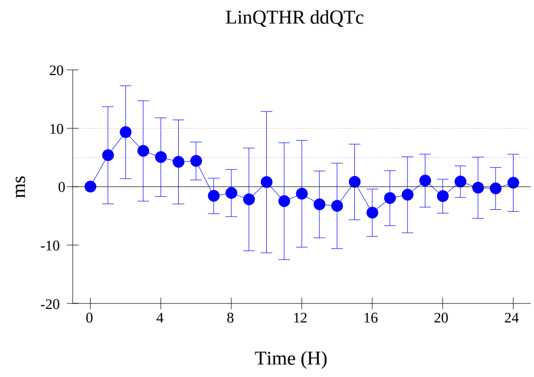

*: P<=0.05: one-sided 95% CI lower bound =>5ms
LSD= 5.7 ms

Figure 14 Chlorpromazine 1 mg/kg iv - Effect on β slope (one step QTc model)

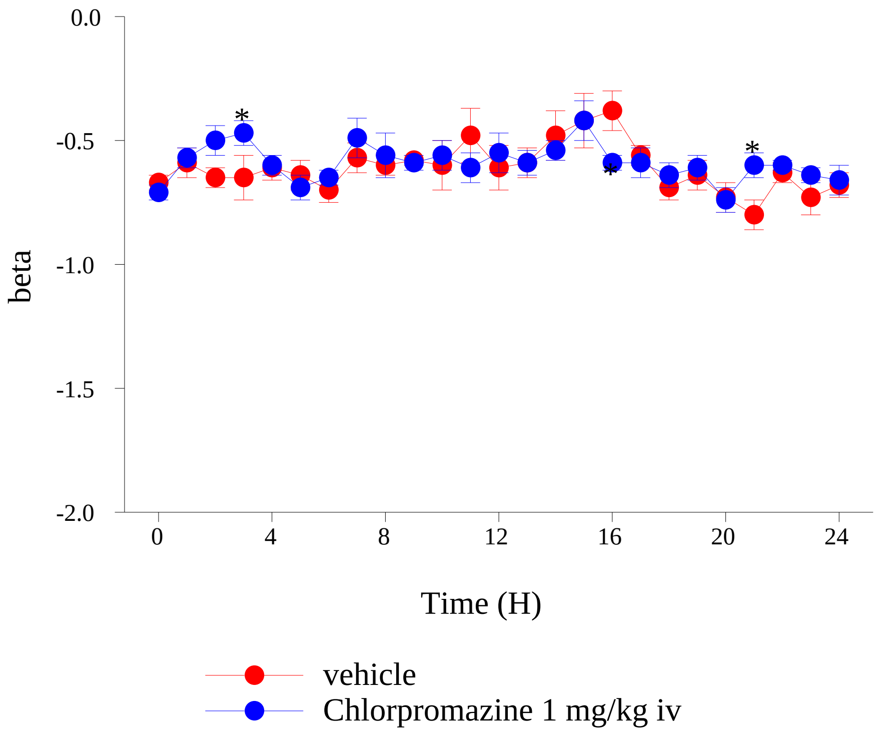

Results expressed in β
Repeated measures analysis of variance (RMANOVA)
Probability for Treatment factor: P=0.643
Probability for Time X Treatment interaction: P=0.217
$\star$: P$\leq$0.05 (LSD)
LSD=0.2 - Least significant difference for α type-1 error=5%
MDD=0.2 - Minimum detectable difference for α type-1 error=5% and β type-2 error=20% (*i.e.*power=80%)
Electronic authentication: created by Pascal Champéroux on 11-FEV-2025 at 14:47:36.376
Study QTOS

Dofetilide 0.1 mg/kg po

Figure 15 Dofetilide 0.1 mg/kg po

One-step QTc dQTc

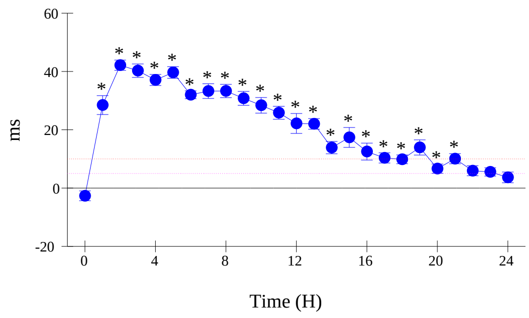

*: P<=0.05: one-sided 95% CI lower bound =>5ms
LSD= 2 ms

One-step QTc ddQTc

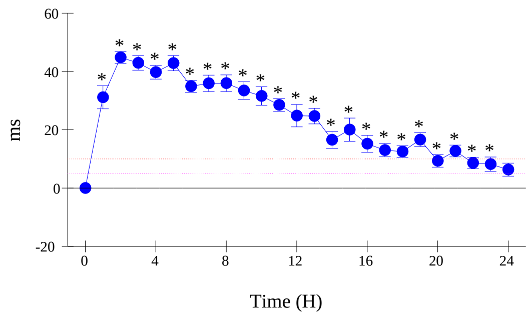

*: P<=0.05: one-sided 95% CI lower bound =>5ms
LSD= 2.5 ms

LogQTRR dQTc

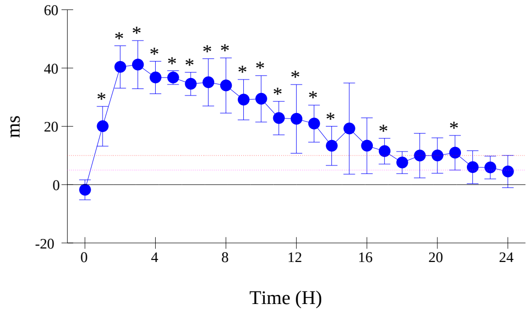

*: P<=0.05: one-sided 95% CI lower bound =>5ms
LSD= 6.3 ms

LogQTRR ddQTc

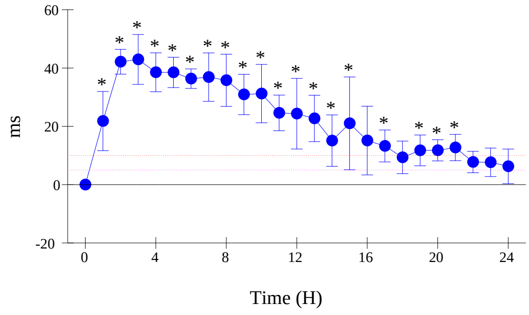

*: P<=0.05: one-sided 95% CI lower bound =>5ms
LSD= 6.1 ms

LinQTHR dQTc

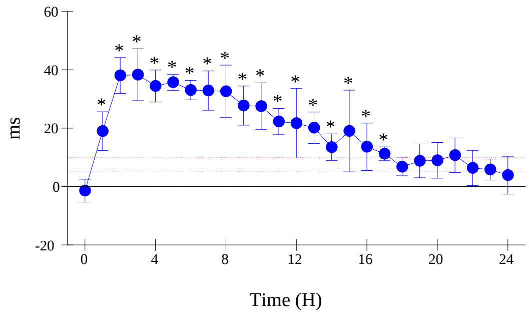

*: P<=0.05: one-sided 95% CI lower bound =>5ms
LSD= 6 ms

LinQTHR ddQTc

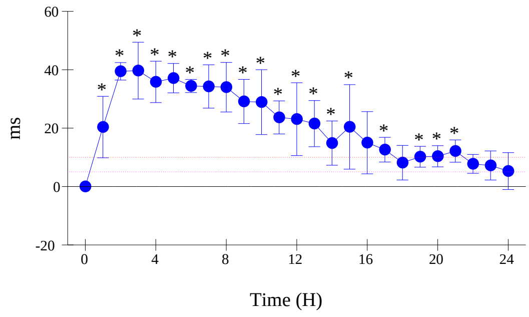

*: P<=0.05: one-sided 95% CI lower bound =>5ms
LSD= 6.3 ms

Figure 16 Dofetilide 0.1 mg/kg po - Effect on β slope (one step QTc model)

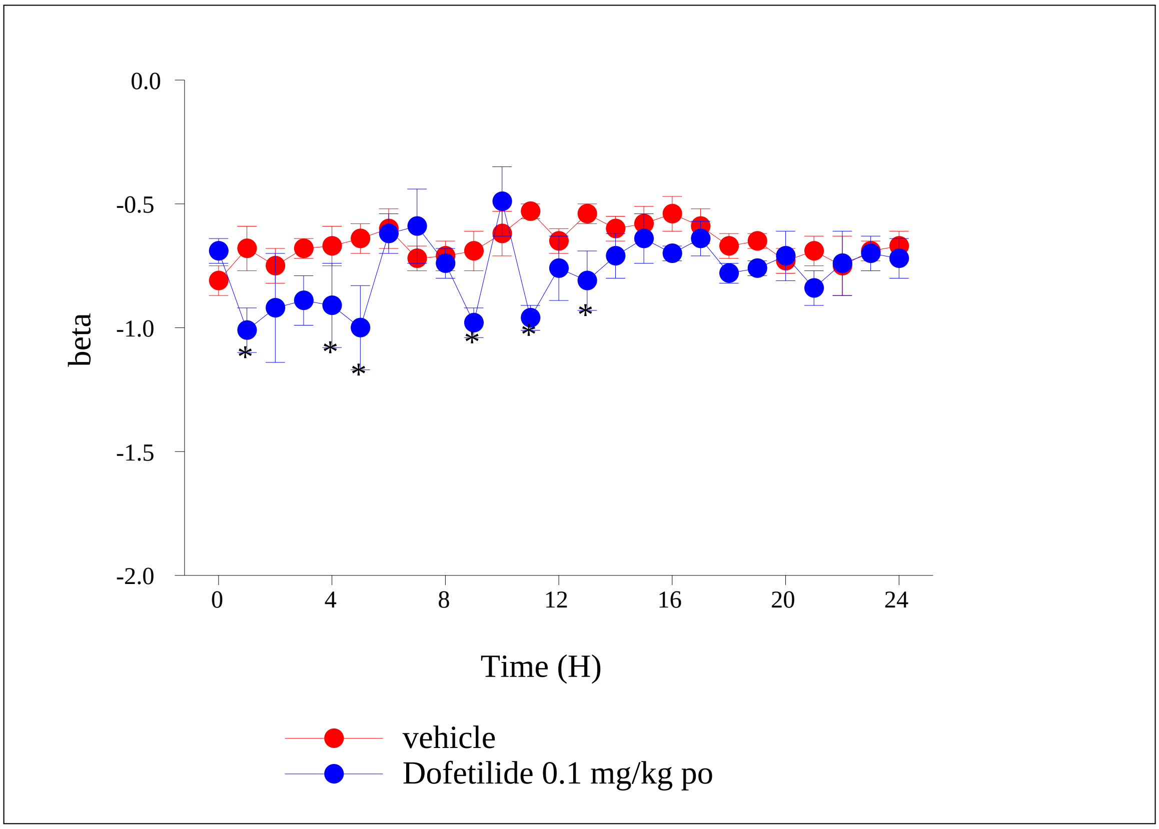

Results expressed in β
Repeated measures analysis of variance (RMANOVA)
Probability for Treatment factor: P=0.013
Probability for Time X Treatment interaction: P=0.053
$\star$: P$\leq$0.05 (LSD)
LSD=0.2 - Least significant difference for α type-1 error=5%
MDD=0.3 - Minimum detectable difference for α type-1 error=5% and β type-2 error=20% (*i.e.*power=80%)
Electronic authentication: created by Pascal Champéroux on 11-FEV-2025 at 14:47:37.303
Study QTOS

Dofetilide 1 mg/kg po

Figure 17 Dofetilide 1 mg/kg po

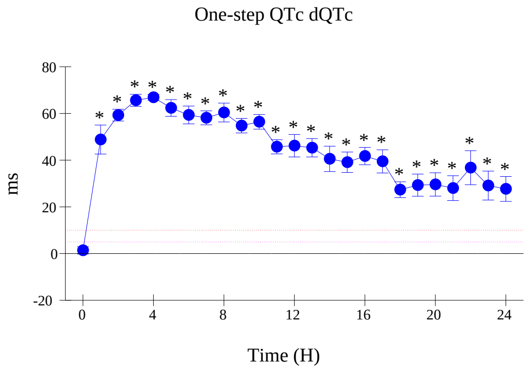

*: P<=0.05: one-sided 95% CI lower bound =>5ms
LSD= 3.9 ms

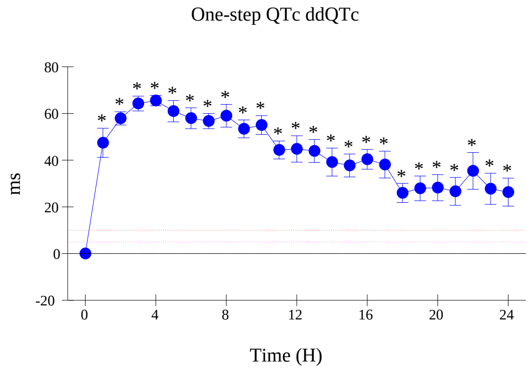

*: P<=0.05: one-sided 95% CI lower bound =>5ms
LSD= 4.9 ms

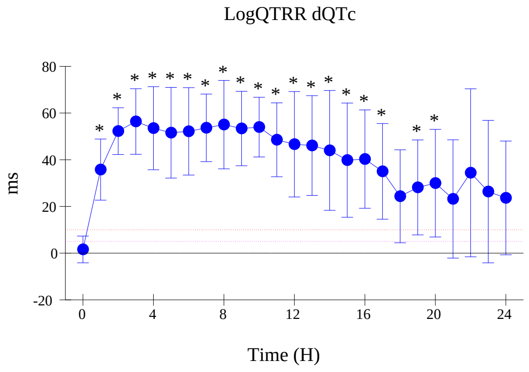

*: P<=0.05: one-sided 95% CI lower bound =>5ms
LSD= 19.9 ms

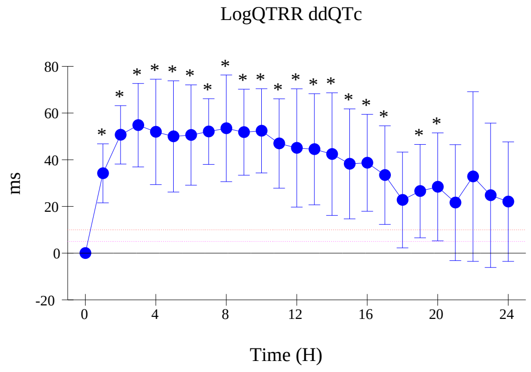

*: P<=0.05: one-sided 95% CI lower bound =>5ms
LSD= 21.5 ms

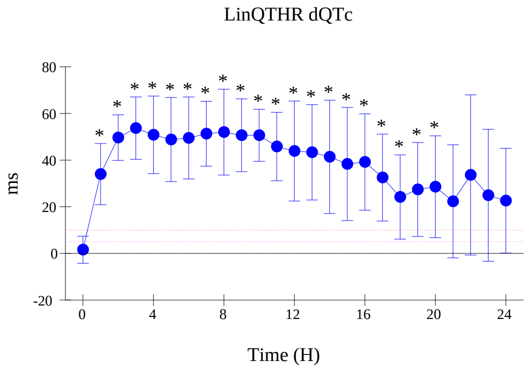

*: P<=0.05: one-sided 95% CI lower bound =>5ms
LSD= 18.4 ms

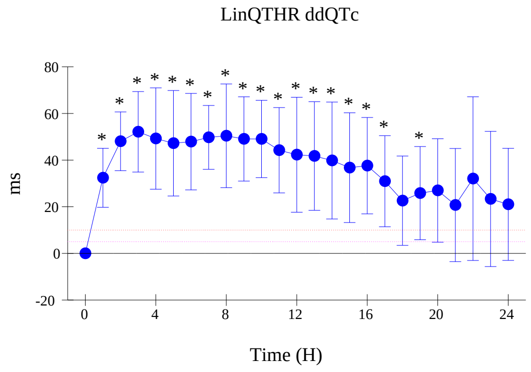

*: P<=0.05: one-sided 95% CI lower bound =>5ms
LSD= 20.7 ms

Figure 18 Dofetilide 1 mg/kg po - Effect on β slope (one step QTc model)

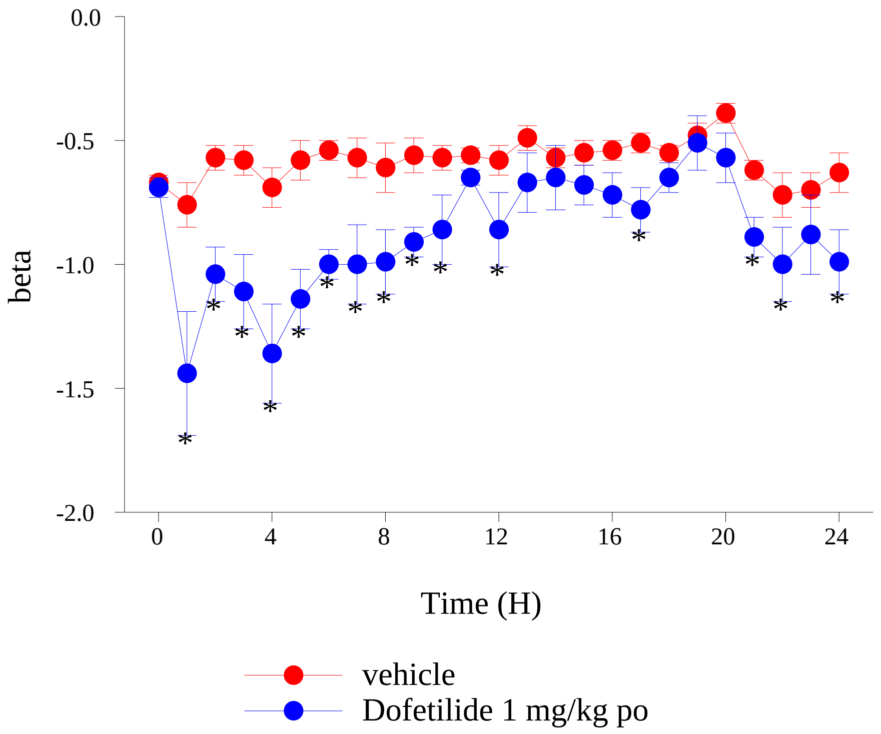

Results expressed in β
Repeated measures analysis of variance (RMANOVA)
Probability for Treatment factor: P=0
Probability for Time X Treatment interaction: P=0.002
$\star$: P$\leq$0.05 (LSD)
LSD=0.3 - Least significant difference for α type-1 error=5%
MDD=0.4 - Minimum detectable difference for α type-1 error=5% and β type-2 error=20% (*i.e.*power=80%)
Electronic authentication: created by Pascal Champéroux on 11-FEV-2025 at 14:47:37.782
Study QTOS

Droperidol 3 mg/kg iv

Figure 19 Droperidol 3 mg/kg iv

One-step QTc dQTc

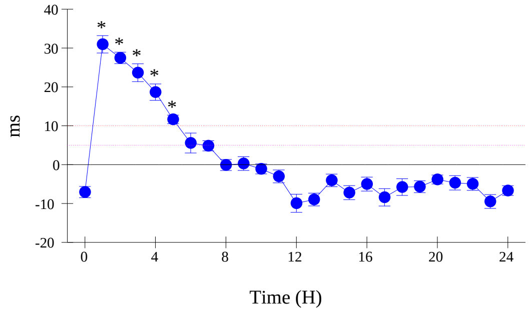

*: P<=0.05: one-sided 95% CI lower bound =>5ms
LSD= 1.7 ms

One-step QTc ddQTc

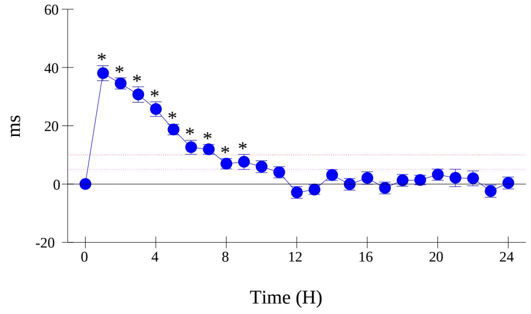

*: P<=0.05: one-sided 95% CI lower bound =>5ms
LSD= 2 ms

LogQTRR dQTc

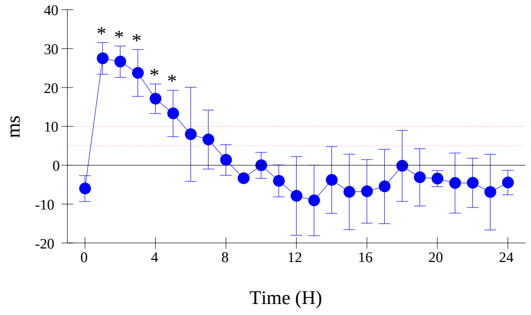

*: P<=0.05: one-sided 95% CI lower bound =>5ms
LSD= 6.3 ms

LogQTRR ddQTc

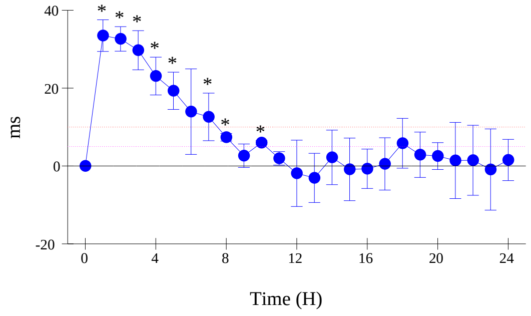

*: P<=0.05: one-sided 95% CI lower bound =>5ms
LSD= 5.3 ms

LinQTHR dQTc

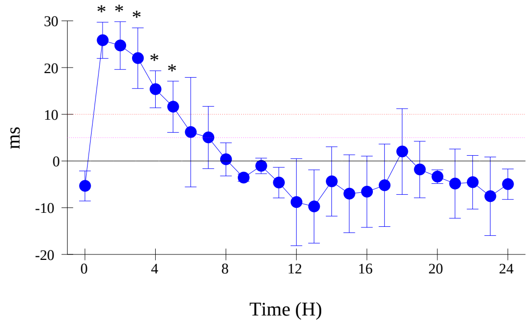

*: P<=0.05: one-sided 95% CI lower bound =>5ms
LSD= 6.1 ms

LinQTHR ddQTc

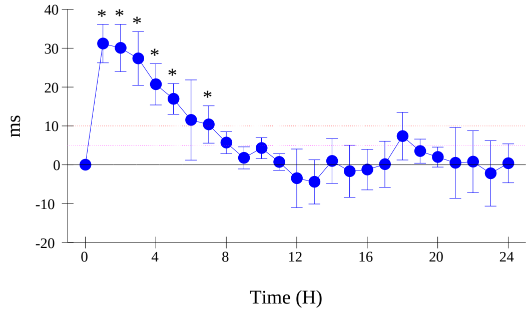

*: P<=0.05: one-sided 95% CI lower bound =>5ms
LSD= 5.3 ms

Figure 20 Droperidol 3 mg/kg iv - Effect on β slope (one step QTc model)

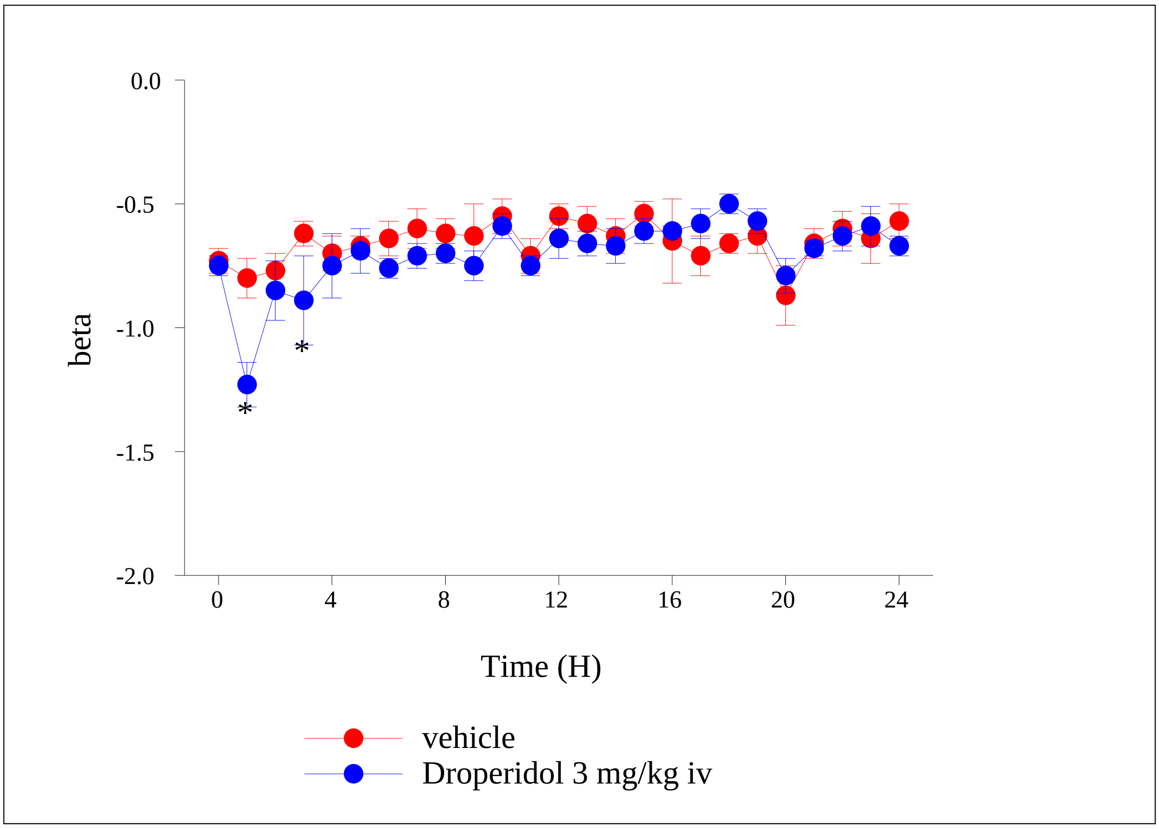

Results expressed in β
Repeated measures analysis of variance (RMANOVA)
Probability for Treatment factor: P=0.386
Probability for Time X Treatment interaction: P=0.067
$\star$: P$\leq$0.05 (LSD)
LSD=0.2 - Least significant difference for α type-1 error=5%
MDD=0.3 - Minimum detectable difference for α type-1 error=5% and β type-2 error=20% (*i.e.*power=80%)
Electronic authentication: created by Pascal Champéroux on 11-FEV-2025 at 14:47:38.244
Study QTOS

Ebastine 30 mg/kg po

Figure 21 Ebastine 30 mg/kg po

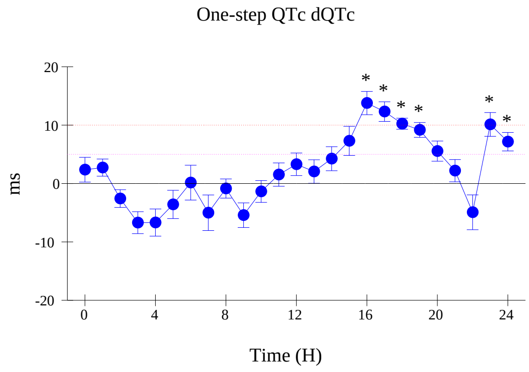

*: P<=0.05: one-sided 95% CI lower bound =>5ms
LSD= 2 ms

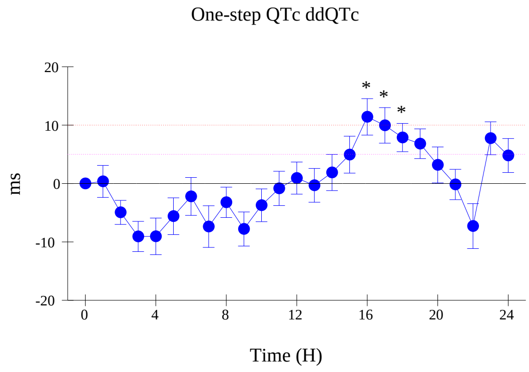

*: P<=0.05: one-sided 95% CI lower bound =>5ms
LSD= 2.9 ms

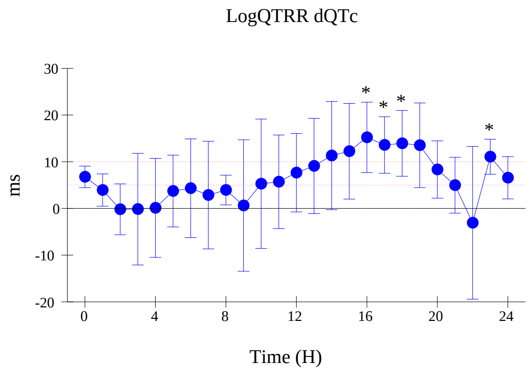

*: P<=0.05: one-sided 95% CI lower bound =>5ms
LSD= 8.4 ms

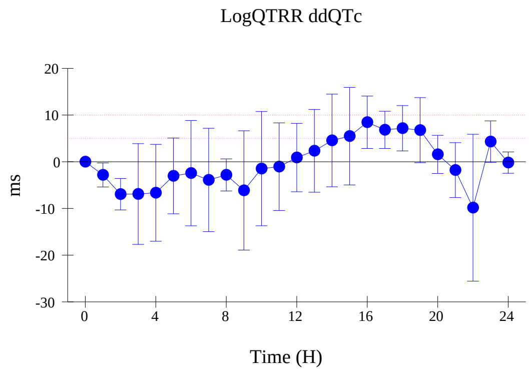

*: P<=0.05: one-sided 95% CI lower bound =>5ms
LSD= 7.3 ms

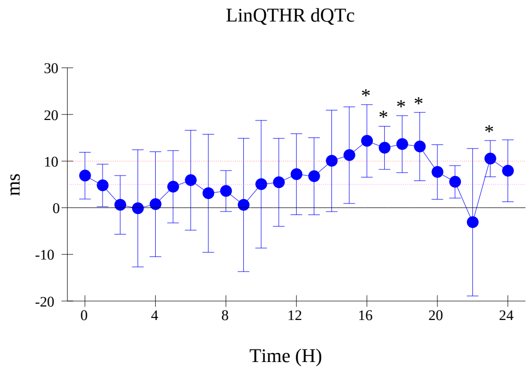

*: P<=0.05: one-sided 95% CI lower bound =>5ms
LSD= 7.8 ms

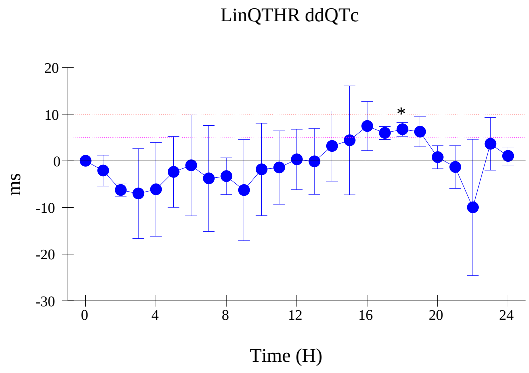

*: P<=0.05: one-sided 95% CI lower bound =>5ms
LSD= 6.5 ms

Figure 22 Ebastine 30 mg/kg po - Effect on β slope (one step QTc model)

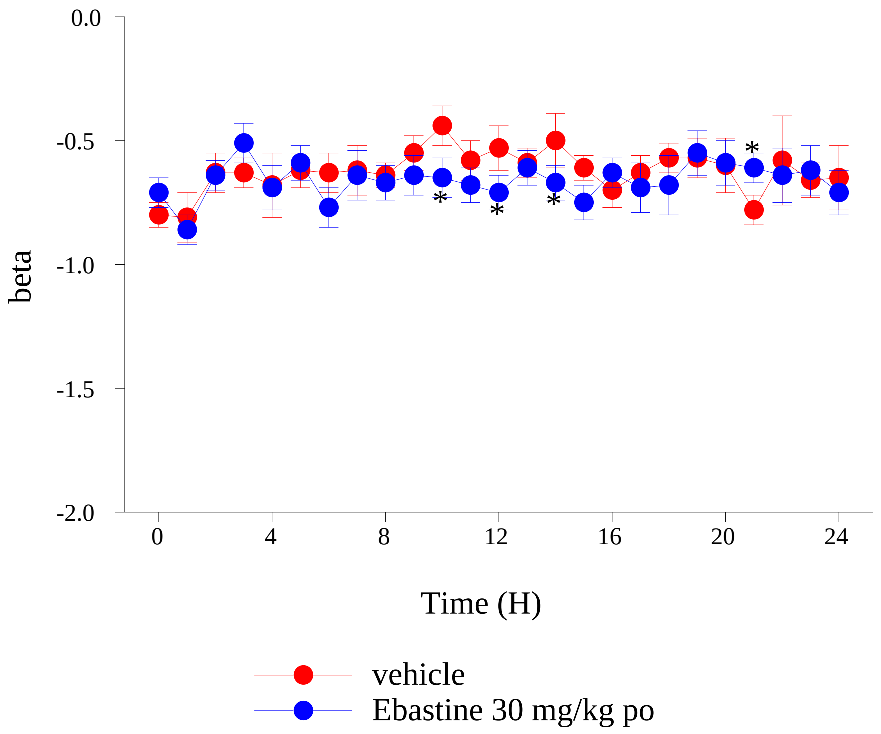

Results expressed in β
Repeated measures analysis of variance (RMANOVA)
Probability for Treatment factor: P=0.691
Probability for Time X Treatment interaction: P=0.222
$\star$: P$\leq$0.05 (LSD)
LSD=0.2 - Least significant difference for α type-1 error=5%
MDD=0.2 - Minimum detectable difference for α type-1 error=5% and β type-2 error=20% (*i.e.*power=80%)
Electronic authentication: created by Pascal Champéroux on 11-FEV-2025 at 14:47:38.689
Study QTOS

Haloperidol 1 mg/kg po

Figure 23 Haloperidol 1 mg/kg po

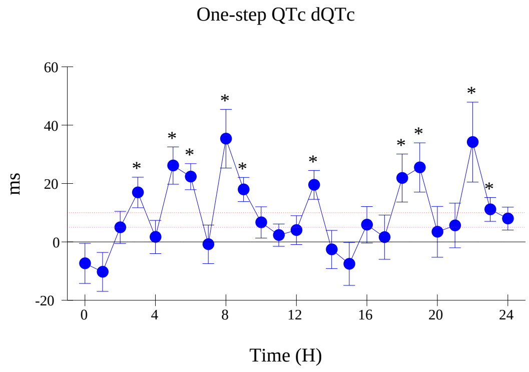

*: P<=0.05: one-sided 95% CI lower bound =>5ms
LSD= 6.4 ms

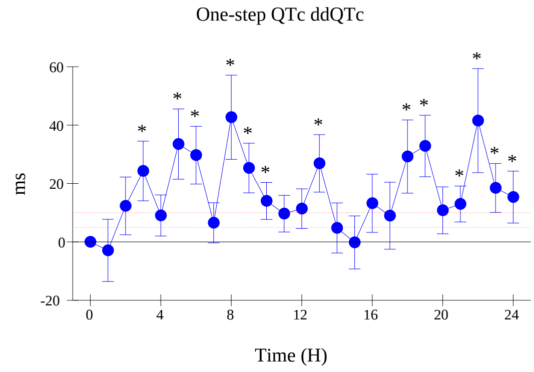

*: P<=0.05: one-sided 95% CI lower bound =>5ms
LSD= 9.1 ms

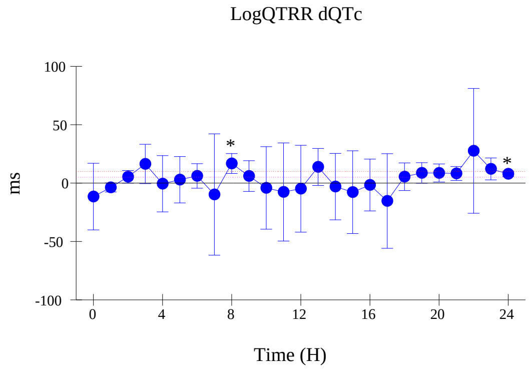

*: P<=0.05: one-sided 95% CI lower bound =>5ms
LSD= 16.9 ms

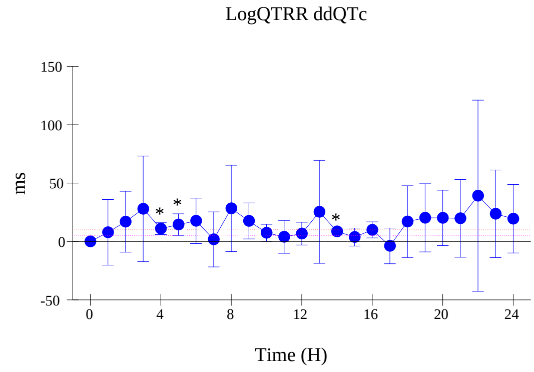

*: P<=0.05: one-sided 95% CI lower bound =>5ms
LSD= 23.6 ms

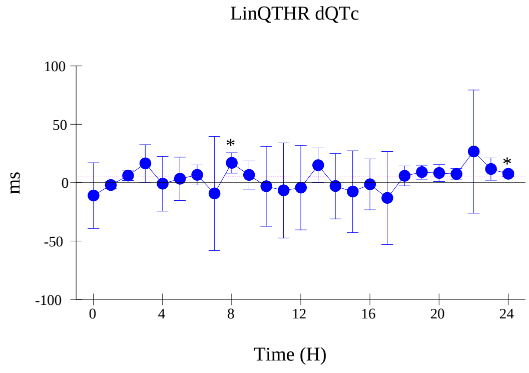

*: P<=0.05: one-sided 95% CI lower bound =>5ms
LSD= 16.1 ms

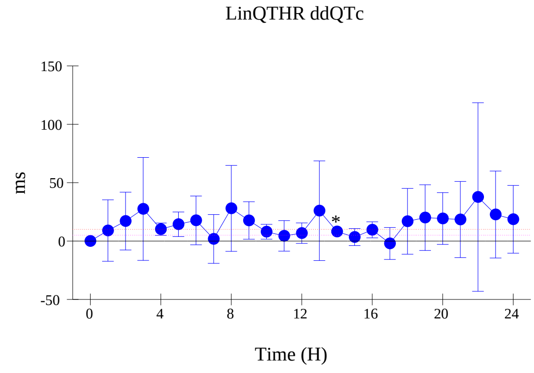

*: P<=0.05: one-sided 95% CI lower bound =>5ms
LSD= 20.9 ms

Figure 24 Haloperidol 1 mg/kg po - Effect on β slope (one step QTc model)

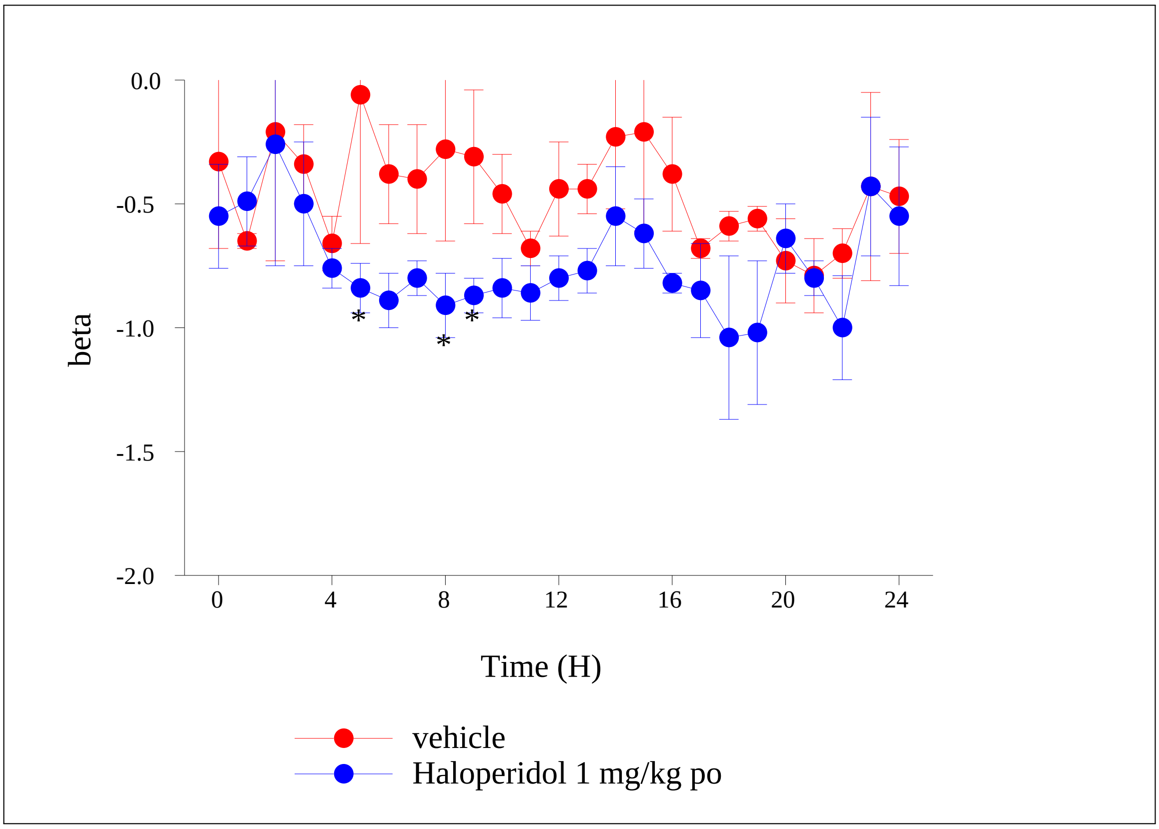

Results expressed in β
Repeated measures analysis of variance (RMANOVA)
Probability for Treatment factor: P=0.16
Probability for Time X Treatment interaction: P=0.861
$\star$: P$\leq$0.05 (LSD)
LSD=0.5 - Least significant difference for α type-1 error=5%
MDD=0.8 - Minimum detectable difference for α type-1 error=5% and β type-2 error=20% (*i.e.*power=80%)
Electronic authentication: created by Pascal Champéroux on 11-FEV-2025 at 14:47:39.150
Study QTOS

Haloperidol 3 mg/kg po

Figure 25 Haloperidol 3 mg/kg po

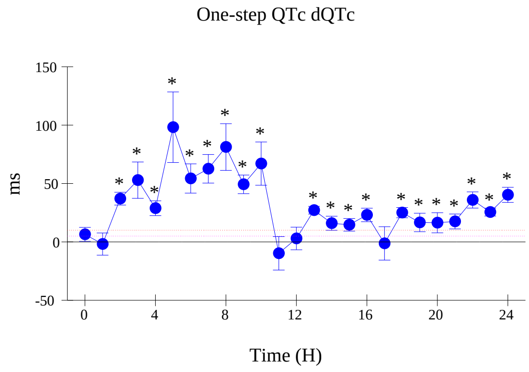

*: P<=0.05: one-sided 95% CI lower bound =>5ms
LSD= 7.9 ms

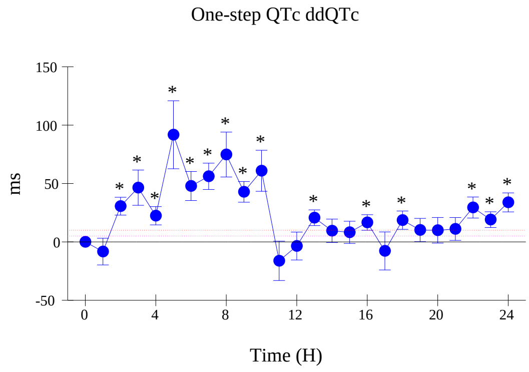

*: P<=0.05: one-sided 95% CI lower bound =>5ms
LSD= 10 ms

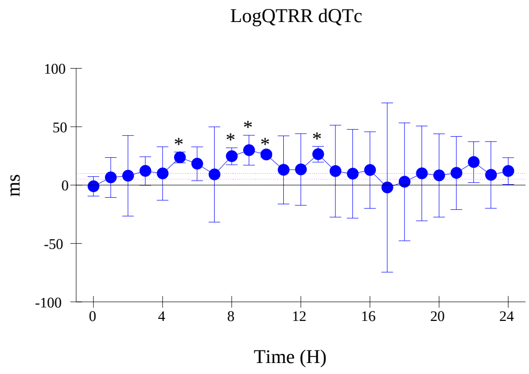

*: P<=0.05: one-sided 95% CI lower bound =>5ms
LSD= 28.6 ms

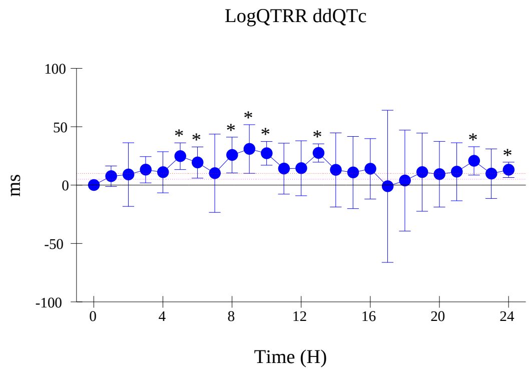

*: P<=0.05: one-sided 95% CI lower bound =>5ms
LSD= 21.2 ms

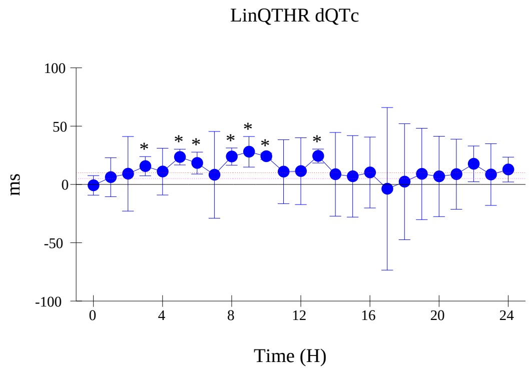

*: P<=0.05: one-sided 95% CI lower bound =>5ms
LSD= 26.5 ms

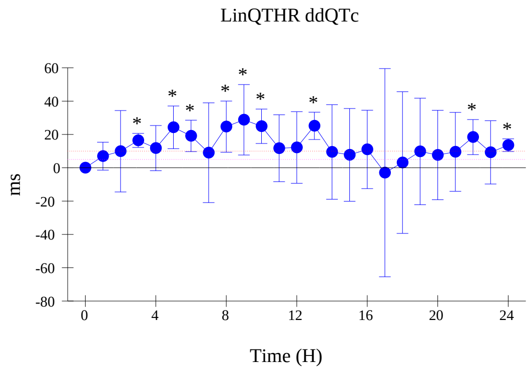

*: P<=0.05: one-sided 95% CI lower bound =>5ms
LSD= 20.1 ms

Figure 26 Haloperidol 3 mg/kg po - Effect on β slope (one step QTc model)

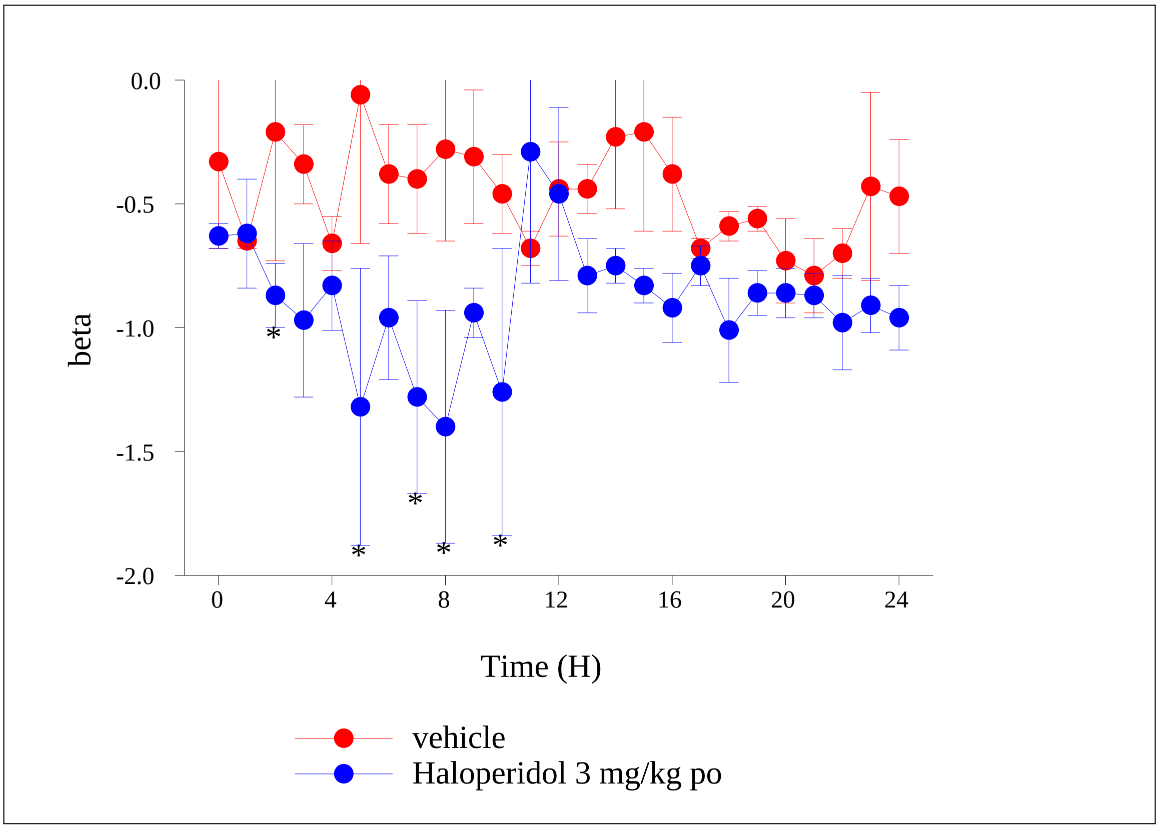

Results expressed in β
Repeated measures analysis of variance (RMANOVA)
Probability for Treatment factor: P=0.056
Probability for Time X Treatment interaction: P=0.211
$\star$: P$\leq$0.05 (LSD)
LSD=0.6 - Least significant difference for α type-1 error=5%
MDD=0.9 - Minimum detectable difference for α type-1 error=5% and β type-2 error=20% (*i.e.*power=80%)
Electronic authentication: created by Pascal Champéroux on 11-FEV-2025 at 14:47:39.387
Study QTOS

Haloperidol 10 mg/kg po

Figure 27 Haloperidol 10 mg/kg po

One-step QTc dQTc

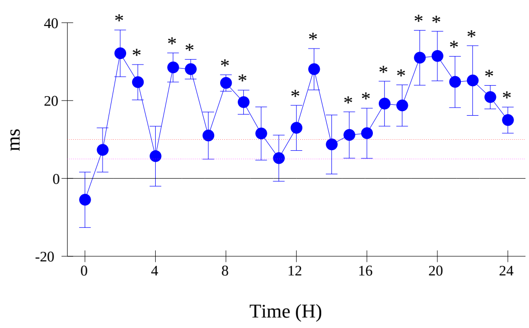

*: P<=0.05: one-sided 95% CI lower bound =>5ms
LSD= 5.9 ms

One-step QTc ddQTc

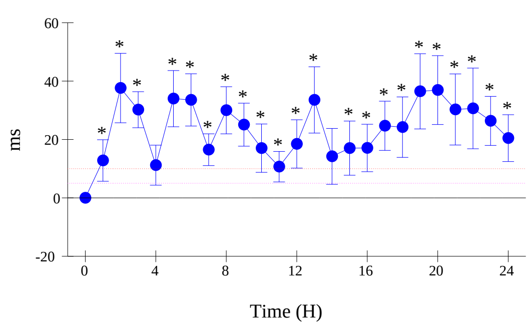

*: P<=0.05: one-sided 95% CI lower bound =>5ms
LSD= 8.4 ms

LogQTRR dQTc

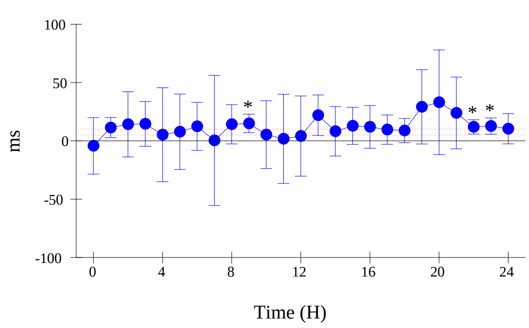

*: P<=0.05: one-sided 95% CI lower bound =>5ms
LSD= 20.6 ms

LogQTRR ddQTc

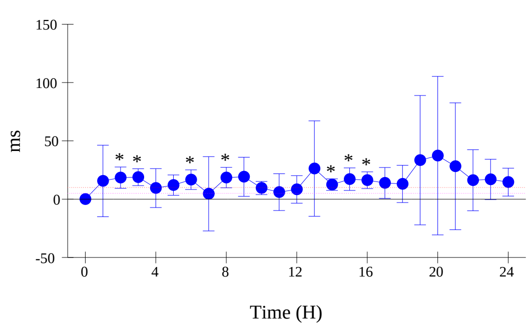

*: P<=0.05: one-sided 95% CI lower bound =>5ms
LSD= 13.2 ms

LinQTHR dQTc

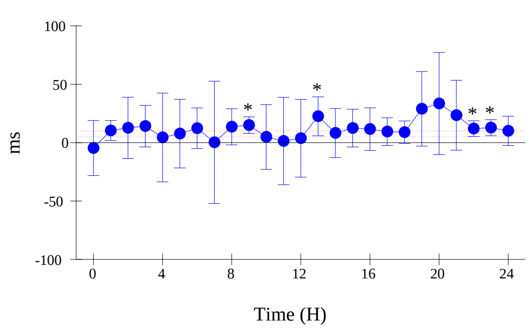

*: P<=0.05: one-sided 95% CI lower bound =>5ms
LSD= 18.2 ms

LinQTHR ddQTc

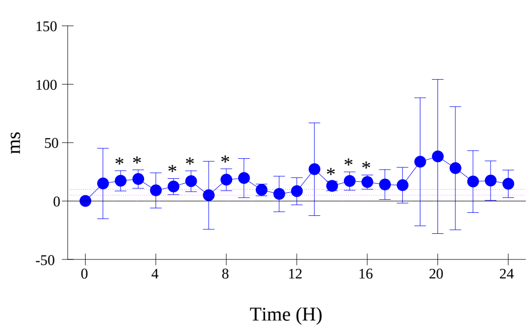

*: P<=0.05: one-sided 95% CI lower bound =>5ms
LSD= 12.8 ms

Figure 28 Haloperidol 10 mg/kg po - Effect on β slope (one step QTc model)

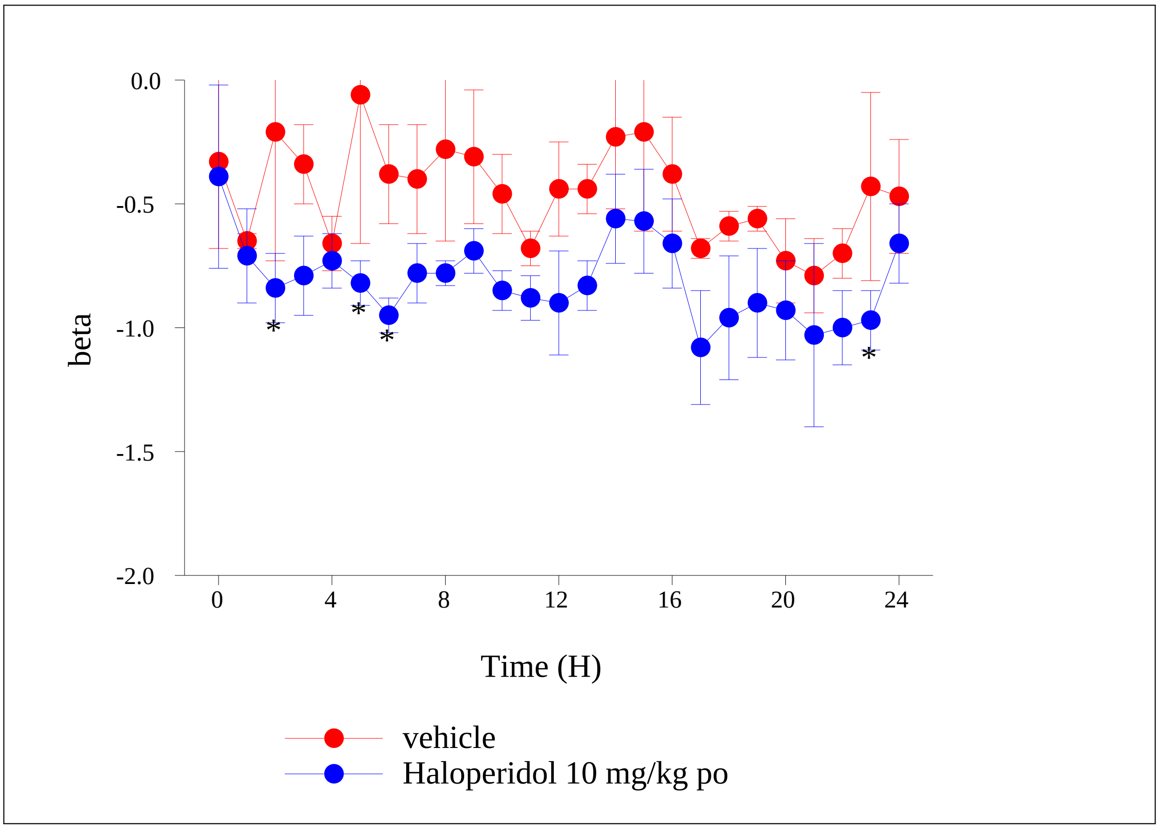

Results expressed in β
Repeated measures analysis of variance (RMANOVA)
Probability for Treatment factor: P=0.085
Probability for Time X Treatment interaction: P=0.993
$\star$: P$\leq$0.05 (LSD)
LSD=0.5 - Least significant difference for α type-1 error=5%
MDD=0.8 - Minimum detectable difference for α type-1 error=5% and β type-2 error=20% (*i.e.*power=80%)
Electronic authentication: created by Pascal Champéroux on 11-FEV-2025 at 14:47:39.829
Study QTOS

Ibutilide 1 mg/kg iv

Figure 29 Ibutilide 1 mg/kg iv

One-step QTc dQTc

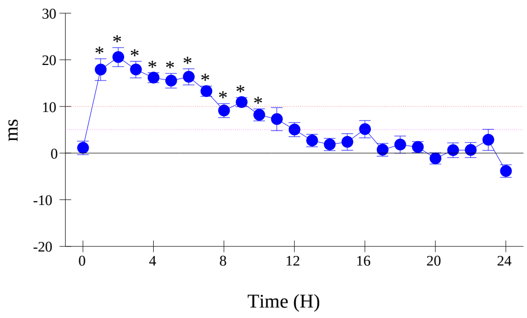

*: P<=0.05: one-sided 95% CI lower bound =>5ms
LSD= 1.5 ms

One-step QTc ddQTc

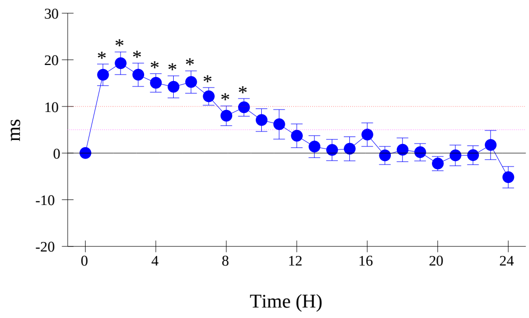

*: P<=0.05: one-sided 95% CI lower bound =>5ms
LSD= 2.3 ms

LogQTRR dQTc

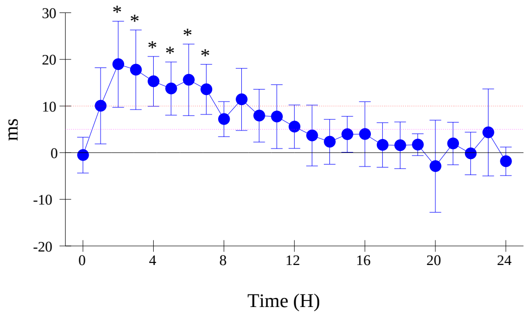

*: P<=0.05: one-sided 95% CI lower bound =>5ms
LSD= 5.4 ms

LogQTRR ddQTc

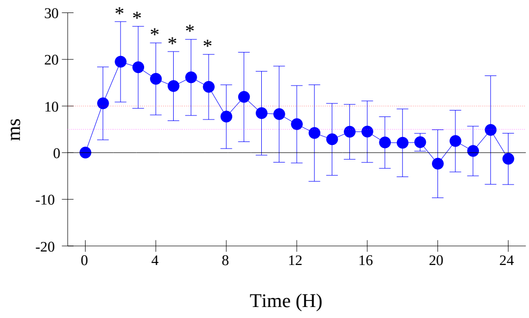

*: P<=0.05: one-sided 95% CI lower bound =>5ms
LSD= 7.4 ms

LinQTHR dQTc

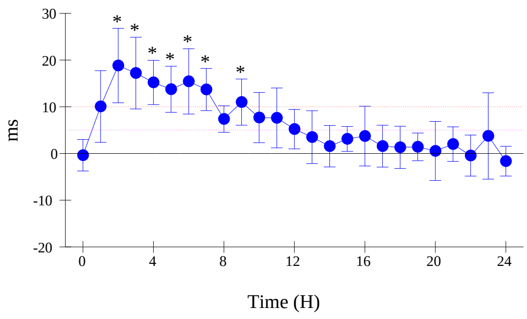

*: P<=0.05: one-sided 95% CI lower bound =>5ms
LSD= 4.7 ms

LinQTHR ddQTc

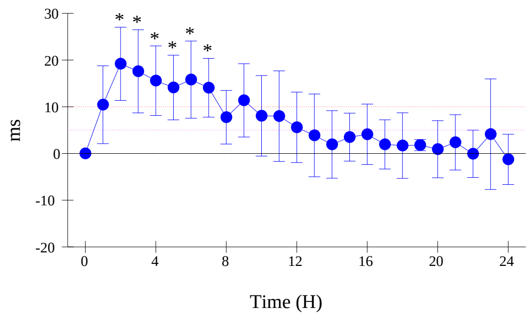

*: P<=0.05: one-sided 95% CI lower bound =>5ms
LSD= 7 ms

Figure 30 Ibutilide 1 mg/kg iv - Effect on β slope (one step QTc model)

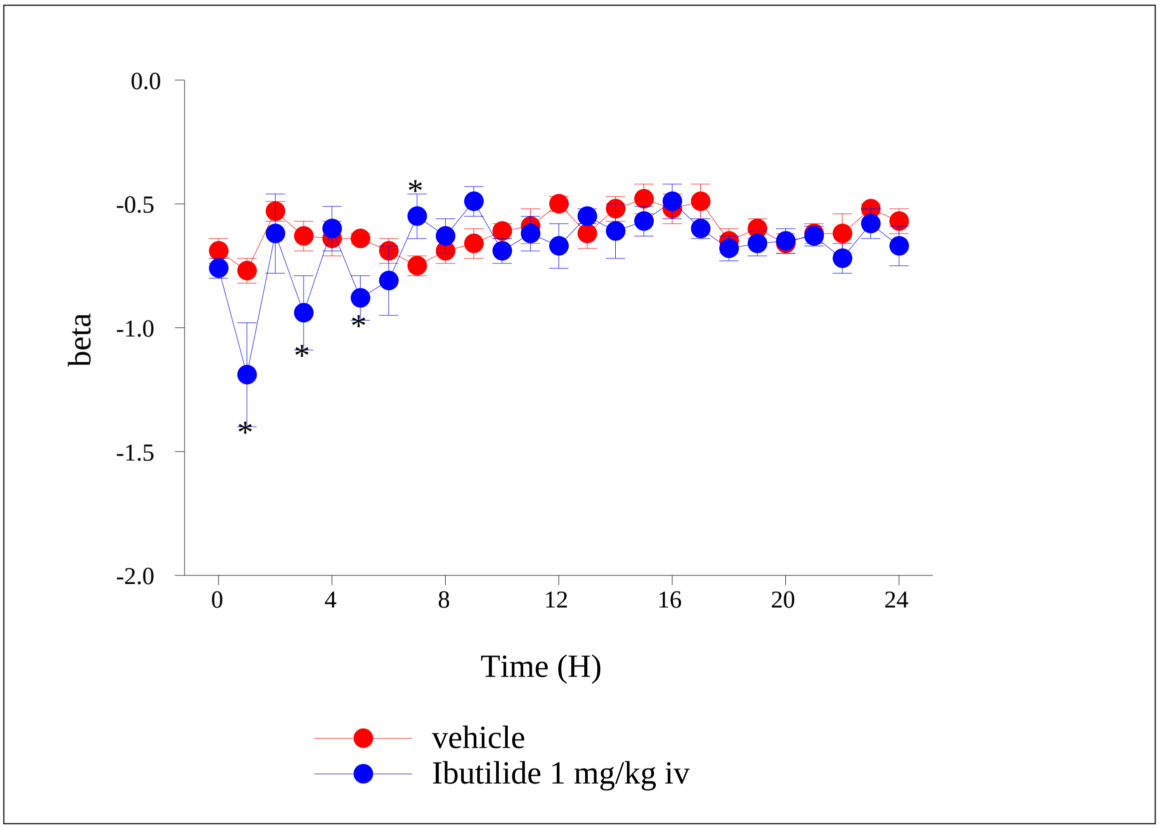

Results expressed in β
Repeated measures analysis of variance (RMANOVA)
Probability for Treatment factor: P=0.127
Probability for Time X Treatment interaction: P=0.017
$\star$: P$\leq$0.05 (LSD)
LSD=0.2 - Least significant difference for α type-1 error=5%
MDD=0.3 - Minimum detectable difference for α type-1 error=5% and β type-2 error=20% (*i.e.*power=80%)
Electronic authentication: created by Pascal Champéroux on 11-FEV-2025 at 14:47:40.293
Study QTOS

Isoprenaline 1 mg/kg po

Figure 31 Isoprenaline 1 mg/kg po

One-step QTc dQTc

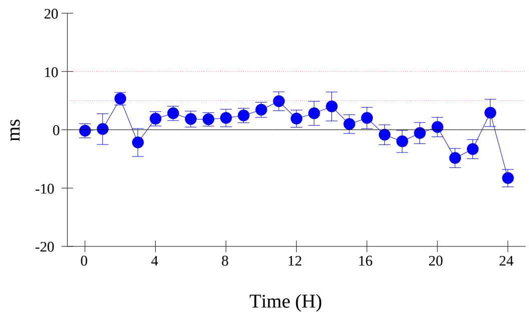

*: P<=0.05: one-sided 95% CI lower bound =>5ms
LSD= 1.6 ms

One-step QTc ddQTc

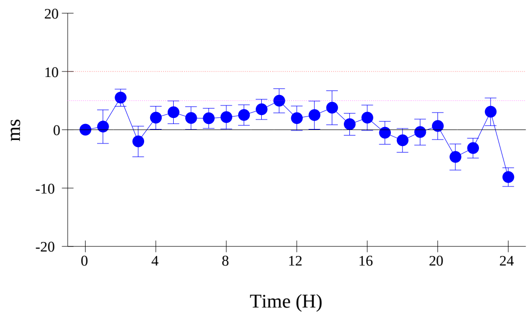

*: P<=0.05: one-sided 95% CI lower bound =>5ms
LSD= 2 ms

LogQTRR dQTc

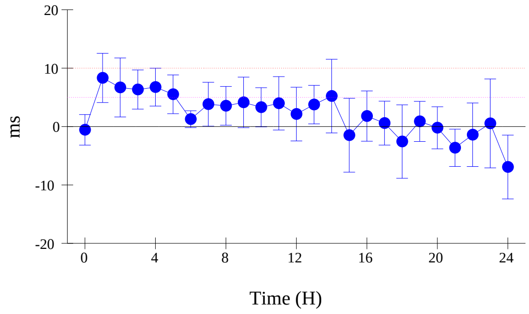

*: P<=0.05: one-sided 95% CI lower bound =>5ms
LSD= 3.8 ms

LogQTRR ddQTc

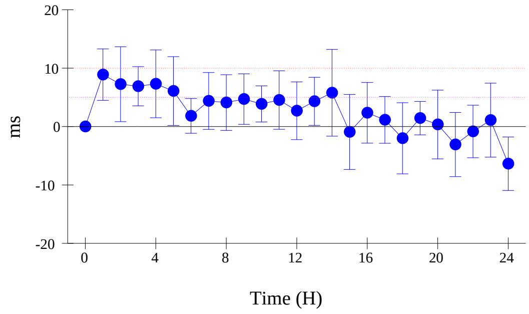

*: P<=0.05: one-sided 95% CI lower bound =>5ms
LSD= 4.9 ms

LinQTHR dQTc

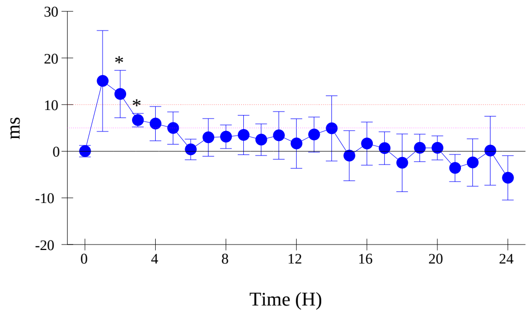

*: P<=0.05: one-sided 95% CI lower bound =>5ms
LSD= 4 ms

LinQTHR ddQTc

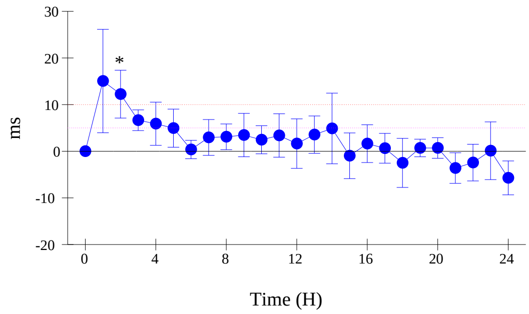

*: P<=0.05: one-sided 95% CI lower bound =>5ms
LSD= 4 ms

Figure 32 Isoprenaline 1 mg/kg po - Effect on β slope (one step QTc model)

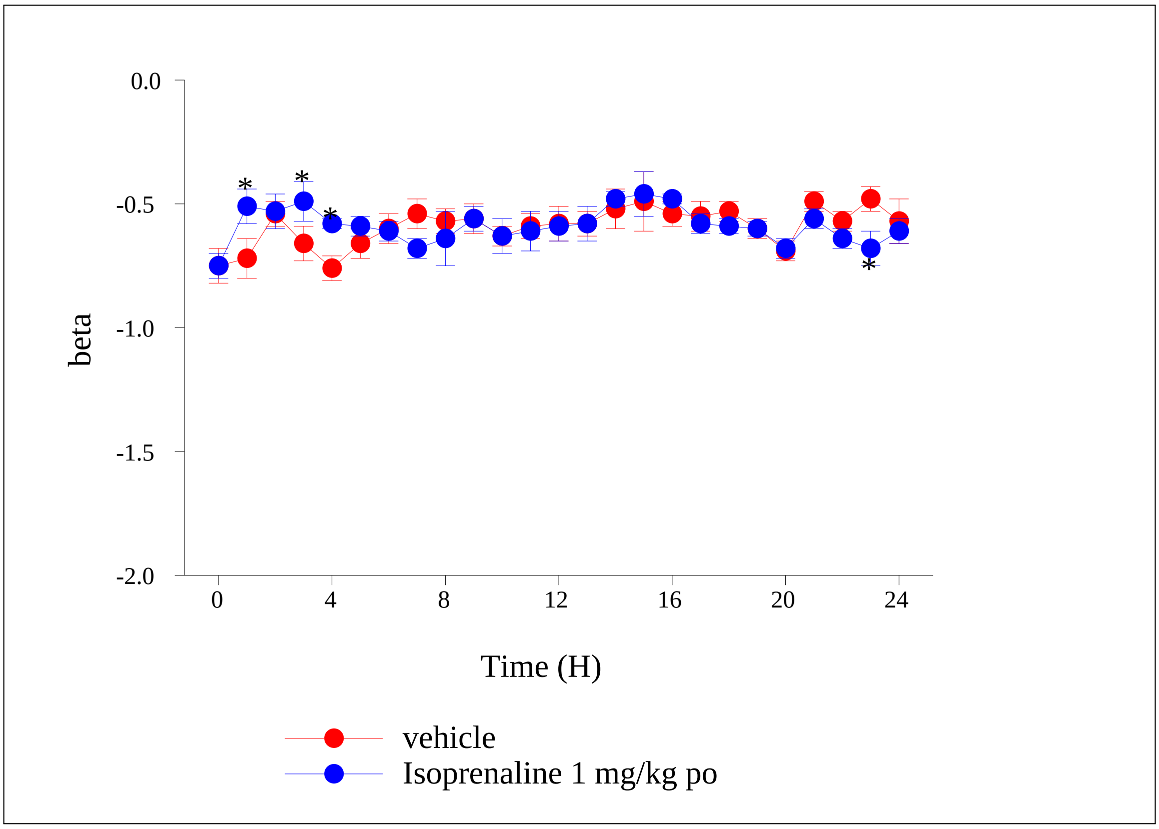

Results expressed in β
Repeated measures analysis of variance (RMANOVA)
Probability for Treatment factor: P=0.936
Probability for Time X Treatment interaction: P=0.119
$\star$: P$\leq$0.05 (LSD)
LSD=0.2 - Least significant difference for α type-1 error=5%
MDD=0.2 - Minimum detectable difference for α type-1 error=5% and β type-2 error=20% (*i.e.*power=80%)
Electronic authentication: created by Pascal Champéroux on 11-FEV-2025 at 14:47:40.770
Study QTOS

Milrinone 1 mg/kg iv

Figure 33 Milrinone 1 mg/kg iv

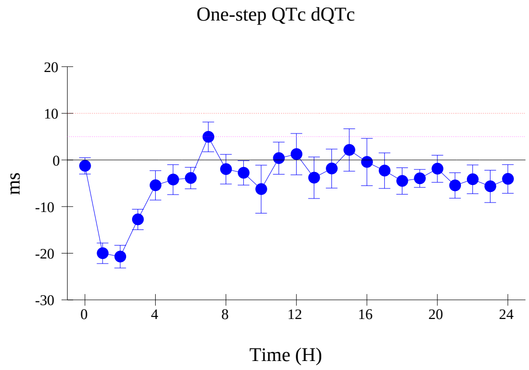

*: P<=0.05: one-sided 95% CI lower bound =>5ms
LSD= 3.2 ms

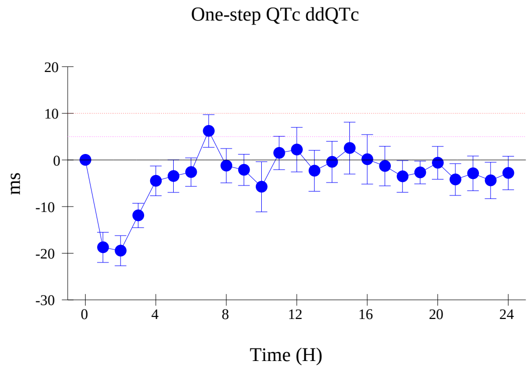

*: P<=0.05: one-sided 95% CI lower bound =>5ms
LSD= 3.5 ms

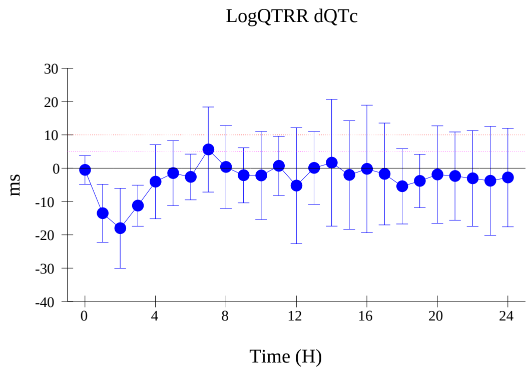

*: P<=0.05: one-sided 95% CI lower bound =>5ms
LSD= 12.5 ms

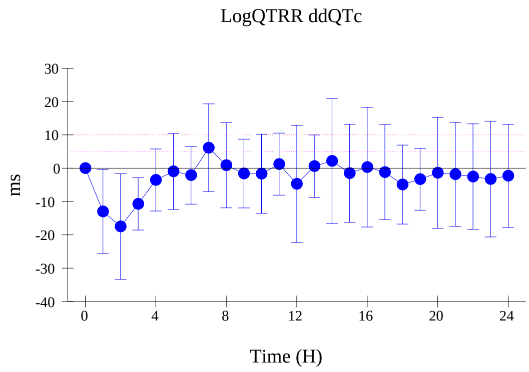

*: P<=0.05: one-sided 95% CI lower bound =>5ms
LSD= 12.8 ms

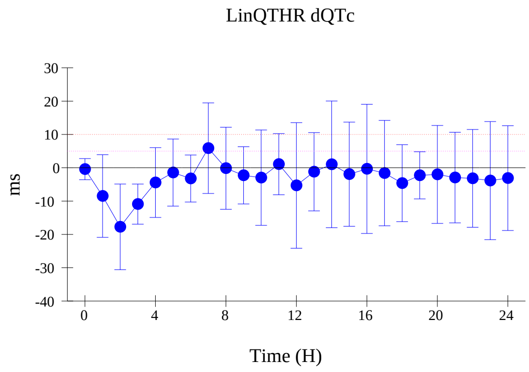

*: P<=0.05: one-sided 95% CI lower bound =>5ms
LSD= 12.9 ms

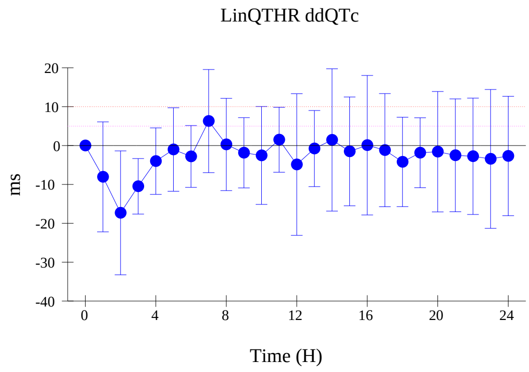

*: P<=0.05: one-sided 95% CI lower bound =>5ms
LSD= 13.3 ms

Figure 34 Milrinone 1 mg/kg iv - Effect on β slope (one step QTc model)

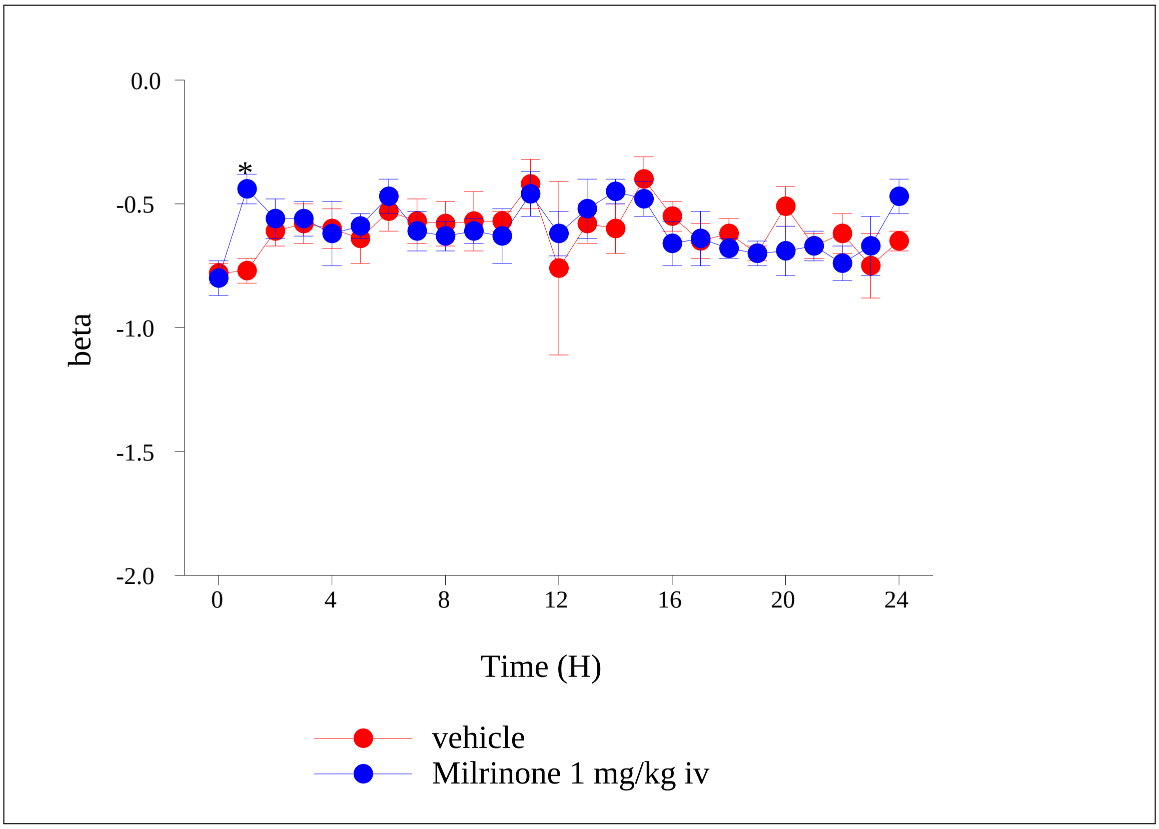

Results expressed in β
Repeated measures analysis of variance (RMANOVA)
Probability for Treatment factor: P=0.838
Probability for Time X Treatment interaction: P=0.726
$\star$: P$\leq$0.05 (LSD)
LSD=0.2 - Least significant difference for α type-1 error=5%
MDD=0.3 - Minimum detectable difference for α type-1 error=5% and β type-2 error=20% (*i.e.*power=80%)
Electronic authentication: created by Pascal Champéroux on 11-FEV-2025 at 14:47:41.249
Study QTOS

Milrinone 3 mg/kg iv

Figure 35 Milrinone 3 mg/kg iv

One-step QTc dQTc

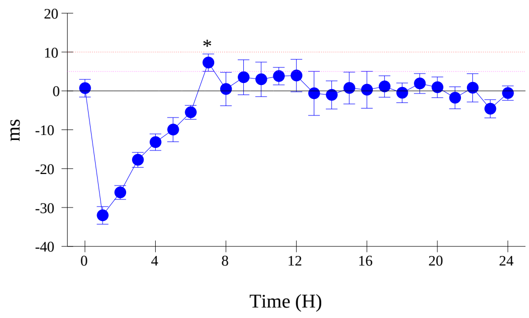

*: P<=0.05: one-sided 95% CI lower bound =>5ms
LSD= 2.7 ms

One-step QTc ddQTc

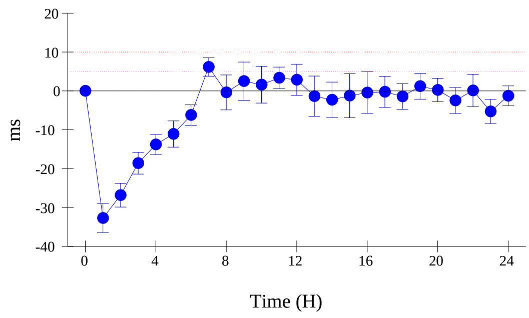

*: P<=0.05: one-sided 95% CI lower bound =>5ms
LSD= 3.3 ms

LogQTRR dQTc

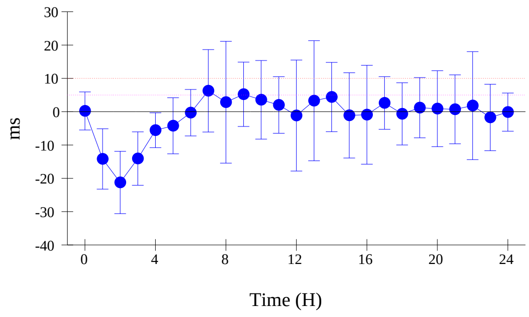

*: P<=0.05: one-sided 95% CI lower bound =>5ms
LSD= 9.7 ms

LogQTRR ddQTc

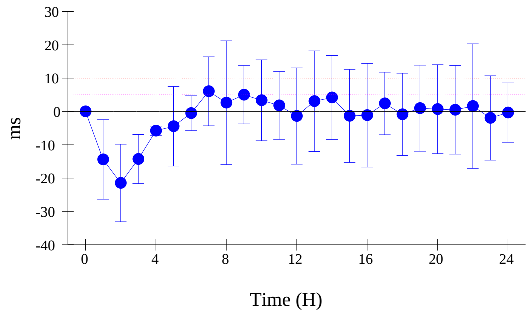

*: P<=0.05: one-sided 95% CI lower bound =>5ms
LSD= 12.1 ms

LinQTHR dQTc

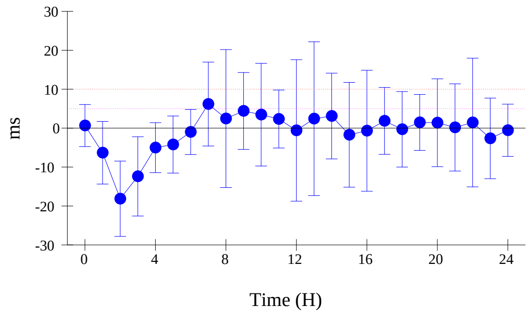

*: P<=0.05: one-sided 95% CI lower bound =>5ms
LSD= 10.2 ms

LinQTHR ddQTc

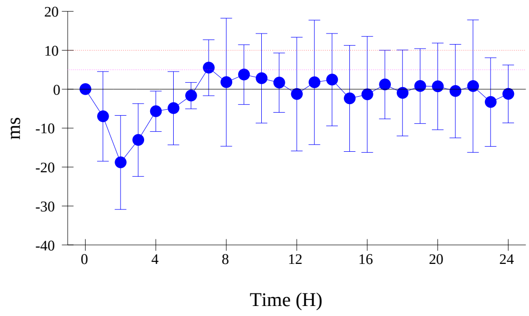

*: P<=0.05: one-sided 95% CI lower bound =>5ms
LSD= 11.1 ms

Figure 36 Milrinone 3 mg/kg iv - Effect on β slope (one step QTc model)

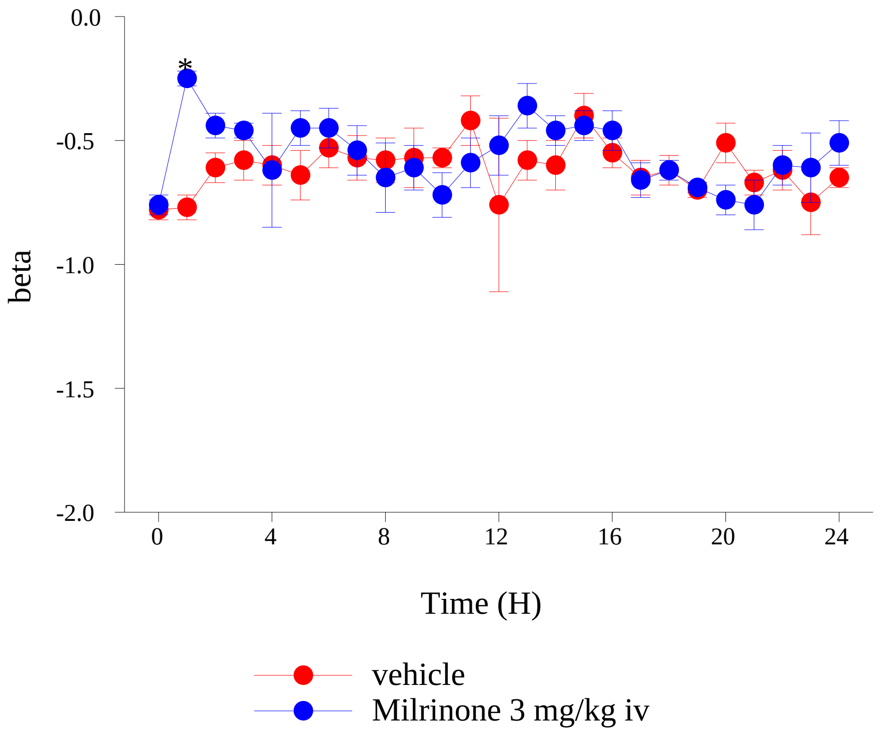

Results expressed in β
Repeated measures analysis of variance (RMANOVA)
Probability for Treatment factor: P=0.372
Probability for Time X Treatment interaction: P=0.096
$\star$: P$\leq$0.05 (LSD)
LSD=0.3 - Least significant difference for α type-1 error=5%
MDD=0.4 - Minimum detectable difference for α type-1 error=5% and β type-2 error=20% (*i.e.*power=80%)
Electronic authentication: created by Pascal Champéroux on 11-FEV-2025 at 14:47:41.743
Study QTOS

Morphine 2 mg/kg sc

Figure 37 Morphine 2 mg/kg sc

One-step QTc dQTc

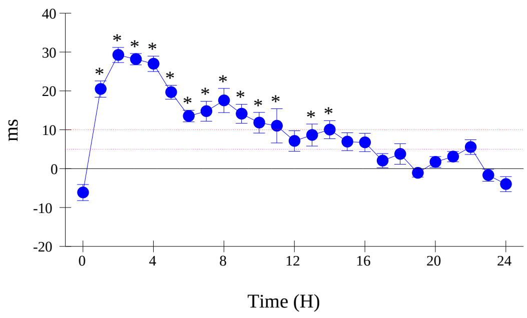

*: P<=0.05: one-sided 95% CI lower bound =>5ms
LSD= 2.1 ms

One-step QTc ddQTc

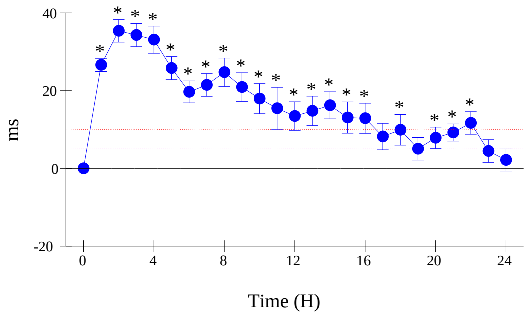

*: P<=0.05: one-sided 95% CI lower bound =>5ms
LSD= 3 ms

LogQTRR dQTc

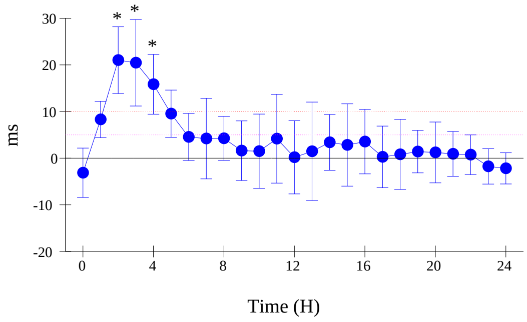

*: P<=0.05: one-sided 95% CI lower bound =>5ms
LSD= 6.4 ms

LogQTRR ddQTc

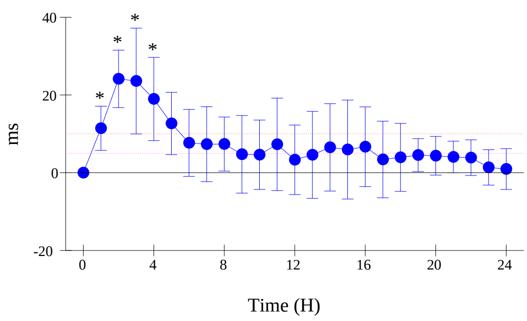

*: P<=0.05: one-sided 95% CI lower bound =>5ms
LSD= 8.7 ms

LinQTHR dQTc

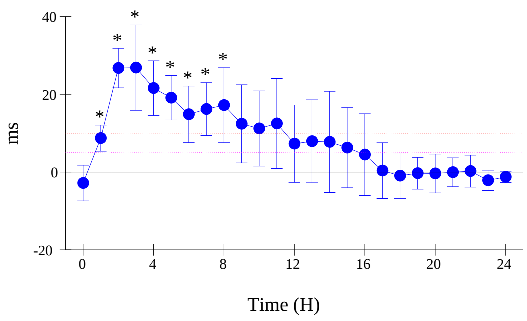

*: P<=0.05: one-sided 95% CI lower bound =>5ms
LSD= 7 ms

LinQTHR ddQTc

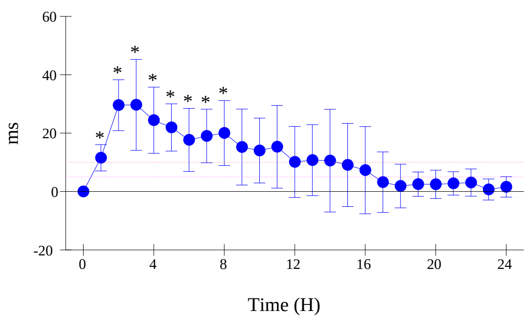

*: P<=0.05: one-sided 95% CI lower bound =>5ms
LSD= 10.4 ms

Figure 38 Morphine 2 mg/kg sc - Effect on β slope (one step QTc model)

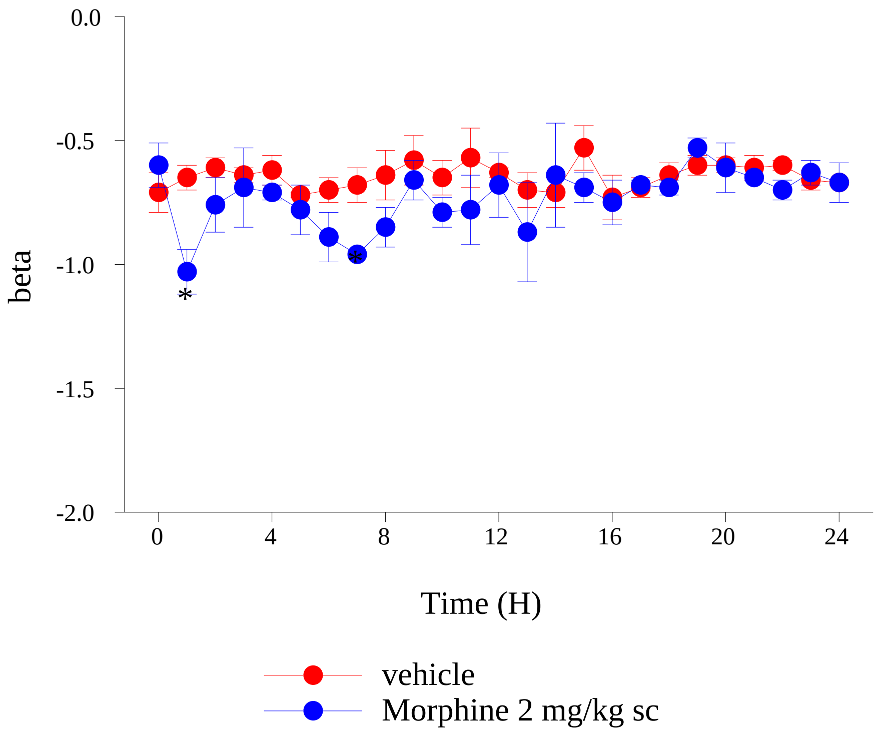

Results expressed in β
Repeated measures analysis of variance (RMANOVA)
Probability for Treatment factor: P=0.094
Probability for Time X Treatment interaction: P=0.394
$\star$: P$\leq$0.05 (LSD)
LSD=0.2 - Least significant difference for α type-1 error=5%
MDD=0.3 - Minimum detectable difference for α type-1 error=5% and β type-2 error=20% (*i.e.*power=80%)
Electronic authentication: created by Pascal Champéroux on 11-FEV-2025 at 14:47:42.019
Study QTOS

Moxifloxacin 10 mg/kg po

Figure 39 Moxifloxacin 10 mg/kg po

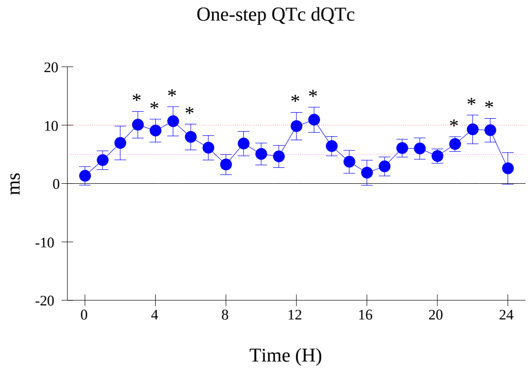

*: P<=0.05: one-sided 95% CI lower bound =>5ms
LSD= 2 ms

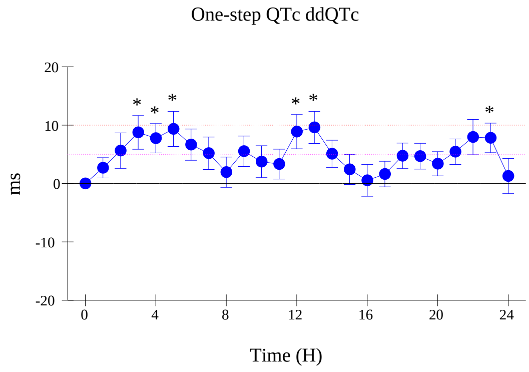

*: P<=0.05: one-sided 95% CI lower bound =>5ms
LSD= 2.6 ms

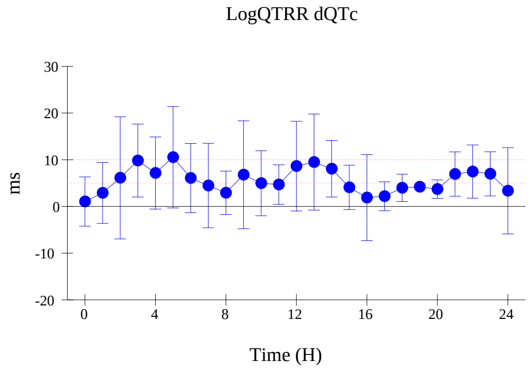

*: P<=0.05: one-sided 95% CI lower bound =>5ms
LSD= 6.5 ms

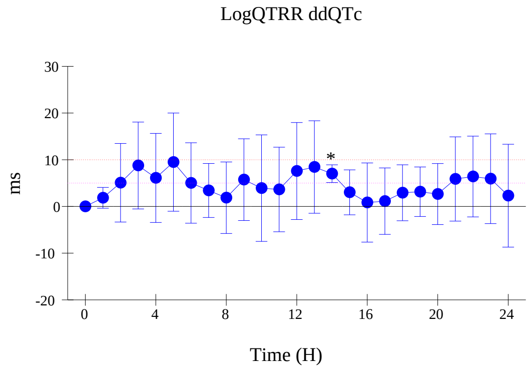

*: P<=0.05: one-sided 95% CI lower bound =>5ms
LSD= 8.6 ms

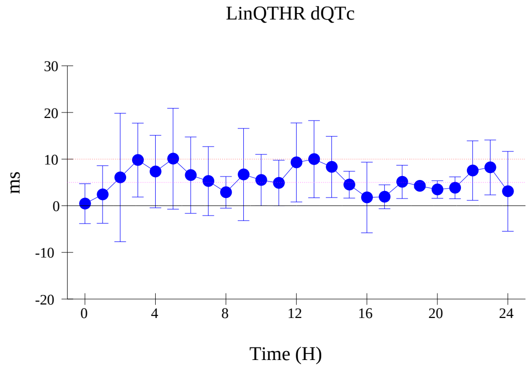

*: P<=0.05: one-sided 95% CI lower bound =>5ms
LSD= 6.4 ms

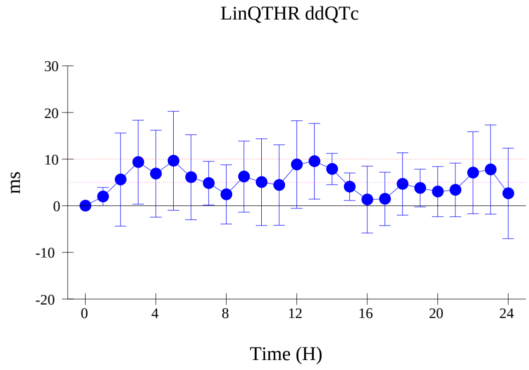

*: P<=0.05: one-sided 95% CI lower bound =>5ms
LSD= 7.6 ms

Figure 40 Moxifloxacin 10 mg/kg po - Effect on β slope (one step QTc model)

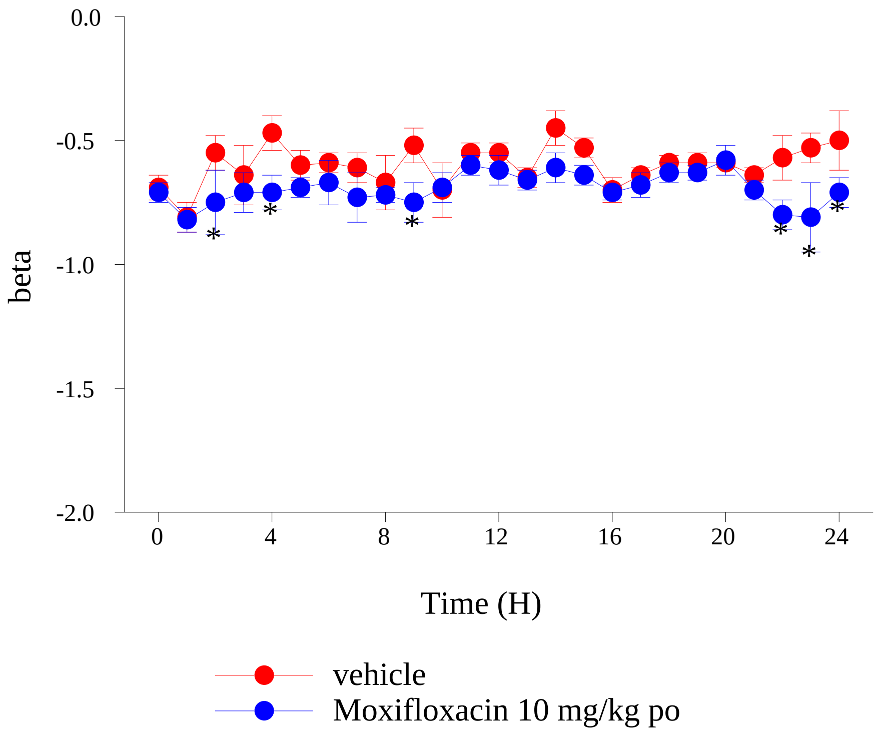

Results expressed in β
Repeated measures analysis of variance (RMANOVA)
Probability for Treatment factor: P=0.025
Probability for Time X Treatment interaction: P=0.526
$\star$: P$\leq$0.05 (LSD)
LSD=0.2 - Least significant difference for α type-1 error=5%
MDD=0.3 - Minimum detectable difference for α type-1 error=5% and β type-2 error=20% (*i.e.*power=80%)
Electronic authentication: created by Pascal Champéroux on 11-FEV-2025 at 14:47:42.210
Study QTOS

Moxifloxacin 30 mg/kg po

Figure 41 Moxifloxacin 30 mg/kg po

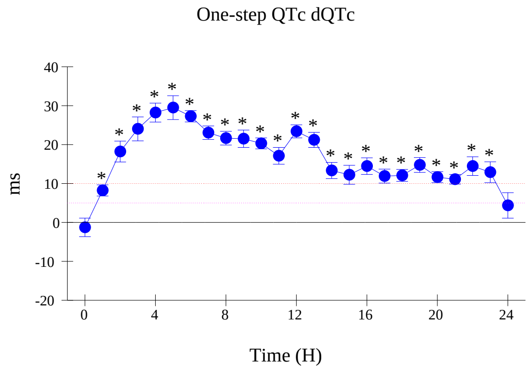

*: P<=0.05: one-sided 95% CI lower bound =>5ms
LSD= 2.1 ms

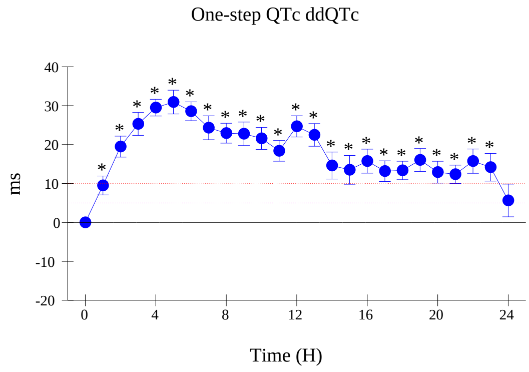

*: P<=0.05: one-sided 95% CI lower bound =>5ms
LSD= 2.8 ms

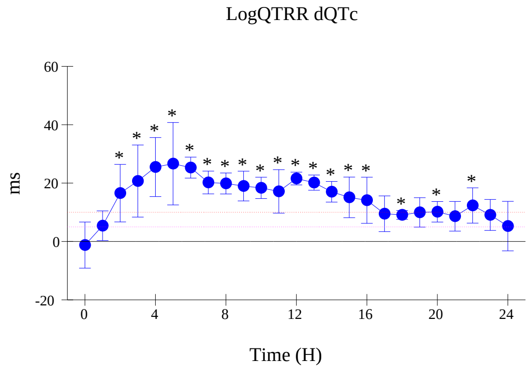

*: P<=0.05: one-sided 95% CI lower bound =>5ms
LSD= 5.1 ms

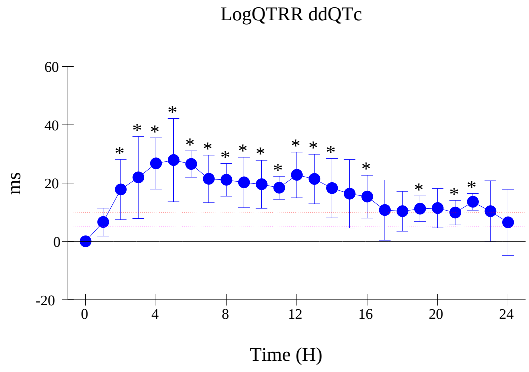

*: P<=0.05: one-sided 95% CI lower bound =>5ms
LSD= 8.2 ms

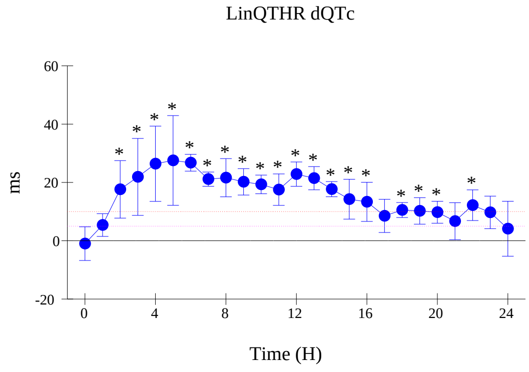

*: P<=0.05: one-sided 95% CI lower bound =>5ms
LSD= 5.4 ms

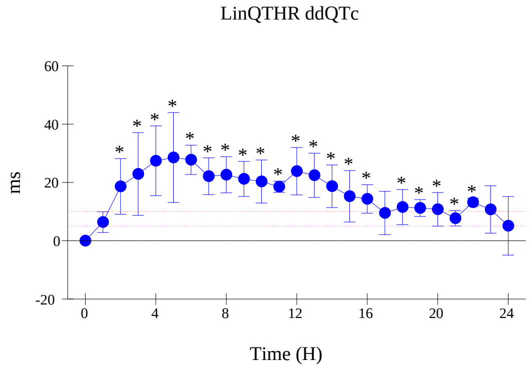

*: P<=0.05: one-sided 95% CI lower bound =>5ms
LSD= 6.3 ms

Figure 42 Moxifloxacin 30 mg/kg po - Effect on β slope (one step QTc model)

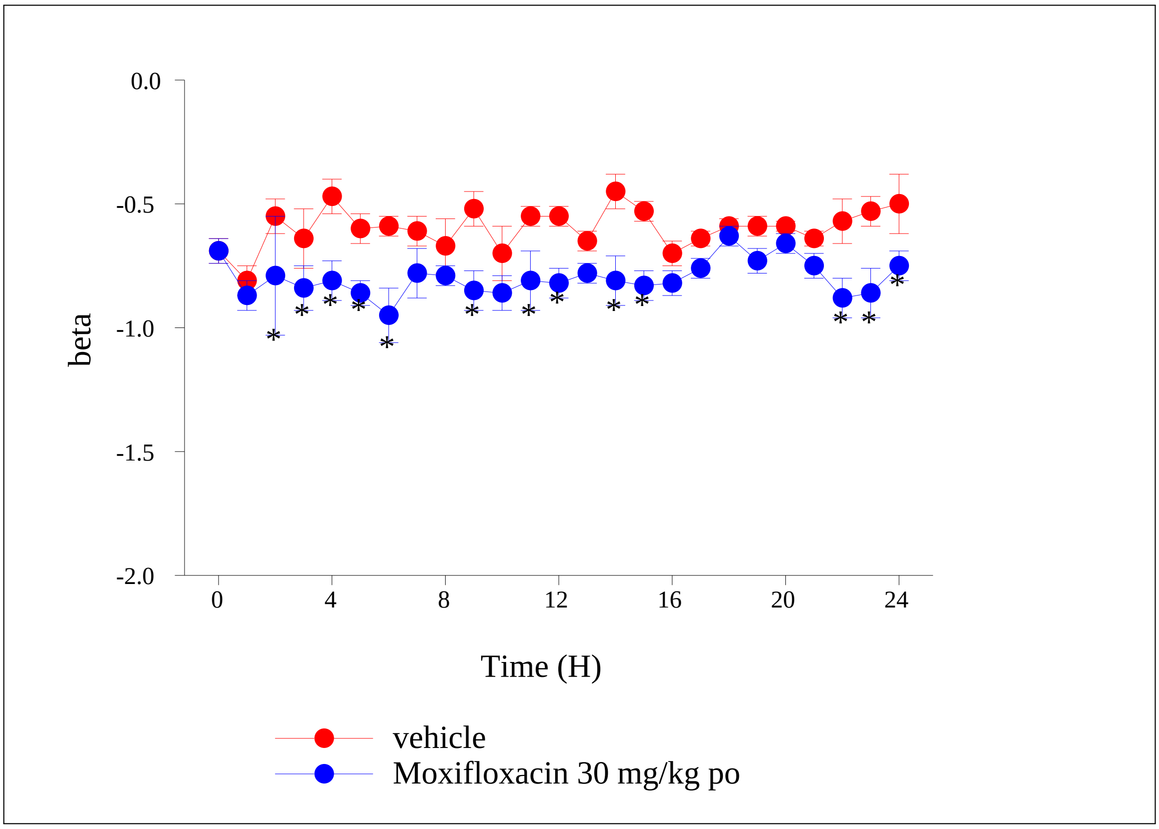

Results expressed in β
Repeated measures analysis of variance (RMANOVA)
Probability for Treatment factor: P=0.002
Probability for Time X Treatment interaction: P=0.236
$\star$: P$\leq$0.05 (LSD)
LSD=0.2 - Least significant difference for α type-1 error=5%
MDD=0.3 - Minimum detectable difference for α type-1 error=5% and β type-2 error=20% (*i.e.*power=80%)
Electronic authentication: created by Pascal Champéroux on 11-FEV-2025 at 14:47:42.386
Study QTOS

Moxifloxacin 90 mg/kg po

Figure 43 Moxifloxacin 90 mg/kg po

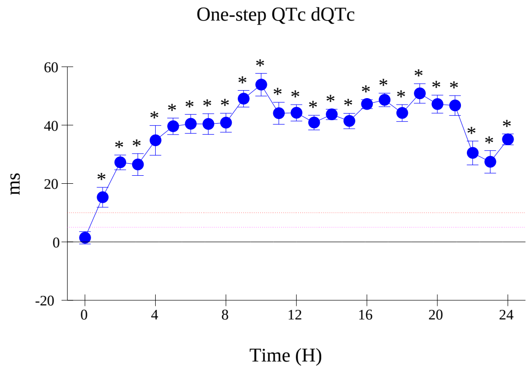

*: P<=0.05: one-sided 95% CI lower bound =>5ms
LSD= 3.1 ms

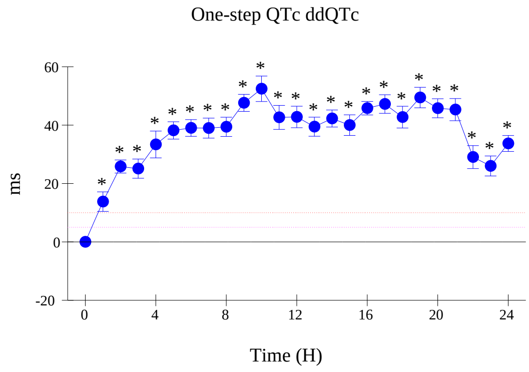

*: P<=0.05: one-sided 95% CI lower bound =>5ms
LSD= 3.3 ms

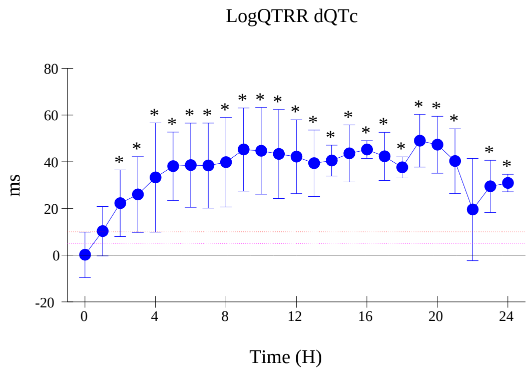

*: P<=0.05: one-sided 95% CI lower bound =>5ms
LSD= 14.2 ms

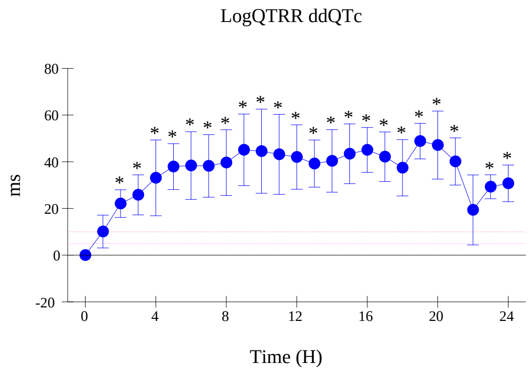

*: P<=0.05: one-sided 95% CI lower bound =>5ms
LSD= 12.1 ms

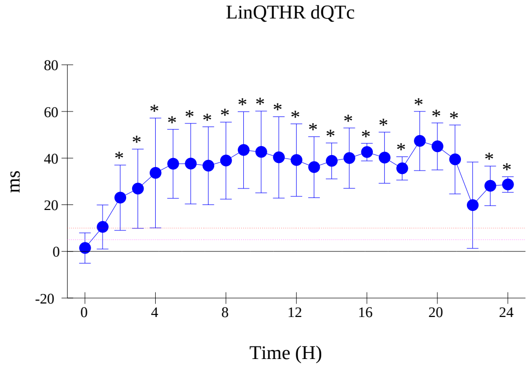

*: P<=0.05: one-sided 95% CI lower bound =>5ms
LSD= 14 ms

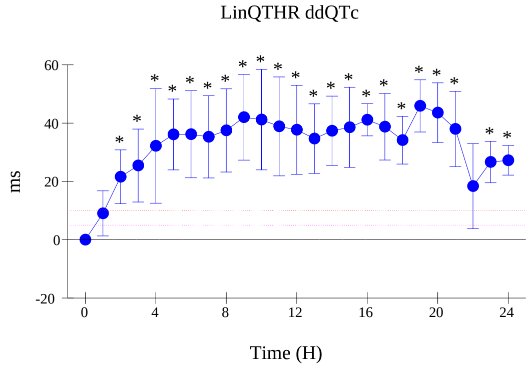

*: P<=0.05: one-sided 95% CI lower bound =>5ms
LSD= 12.2 ms

Figure 44 Moxifloxacin 90 mg/kg po - Effect on β slope (one step QTc model)

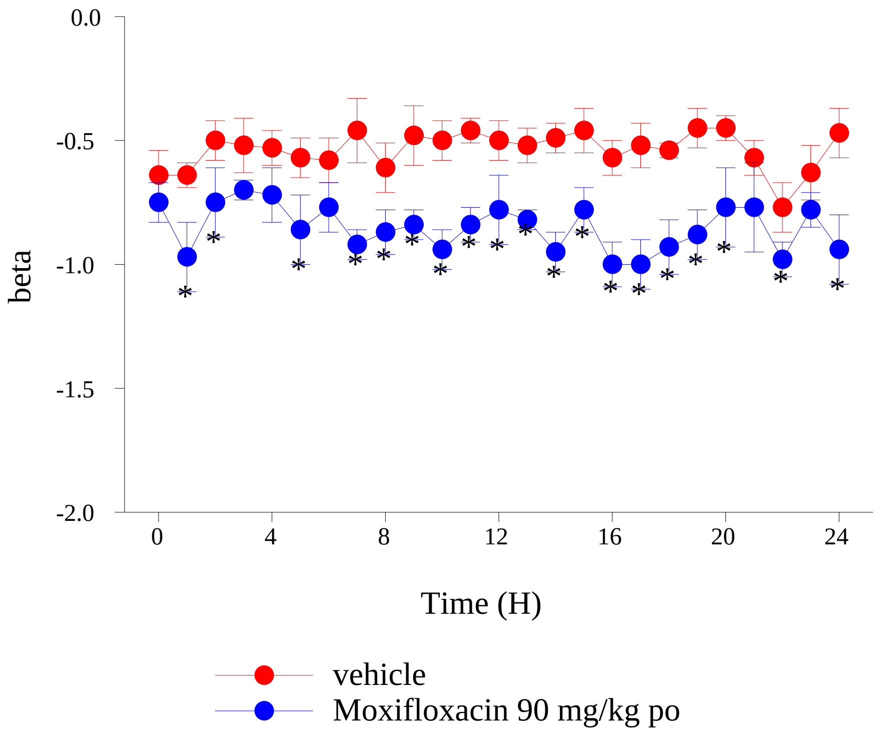

Results expressed in β
Repeated measures analysis of variance (RMANOVA)
Probability for Treatment factor: P=0.006
Probability for Time X Treatment interaction: P=0.26
$\star$: P$\leq$0.05 (LSD)
LSD=0.2 - Least significant difference for α type-1 error=5%
MDD=0.3 - Minimum detectable difference for α type-1 error=5% and β type-2 error=20% (*i.e.*power=80%)
Electronic authentication: created by Pascal Champéroux on 11-FEV-2025 at 14:47:42.591
Study QTOS

Nicardipine 3 mg/kg po

Figure 45 Nicardipine 3 mg/kg po

One-step QTc dQTc

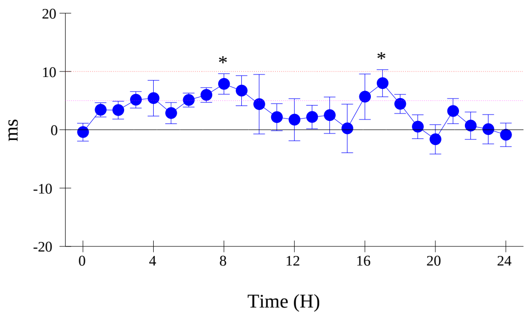

*: P<=0.05: one-sided 95% CI lower bound =>5ms
LSD= 2.2 ms

One-step QTc ddQTc

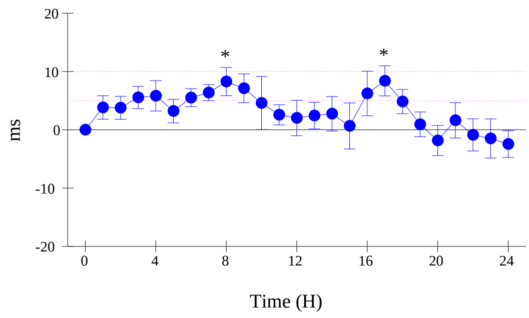

*: P<=0.05: one-sided 95% CI lower bound =>5ms
LSD= 2.4 ms

LogQTRR dQTc

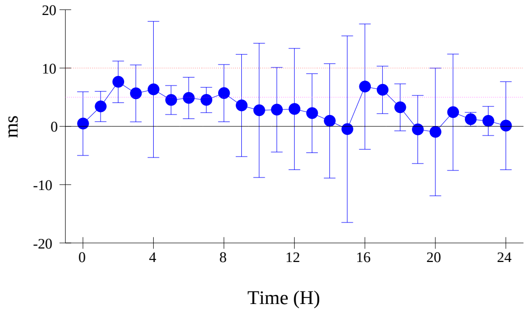

*: P<=0.05: one-sided 95% CI lower bound =>5ms
LSD= 5.8 ms

LogQTRR ddQTc

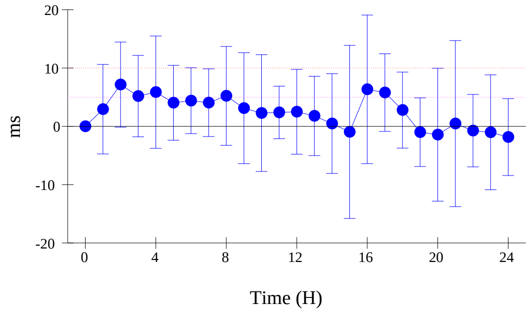

*: P<=0.05: one-sided 95% CI lower bound =>5ms
LSD= 7.3 ms

LinQTHR dQTc

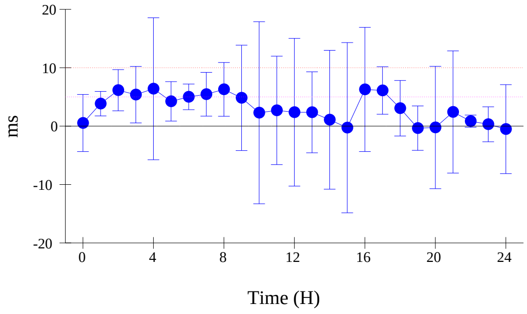

*: P<=0.05: one-sided 95% CI lower bound =>5ms
LSD= 4.9 ms

LinQTHR ddQTc

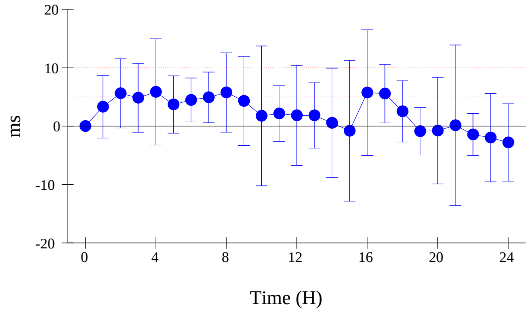

*: P<=0.05: one-sided 95% CI lower bound =>5ms
LSD= 5.9 ms

Figure 46 Nicardipine 3 mg/kg po - Effect on β slope (one step QTc model)

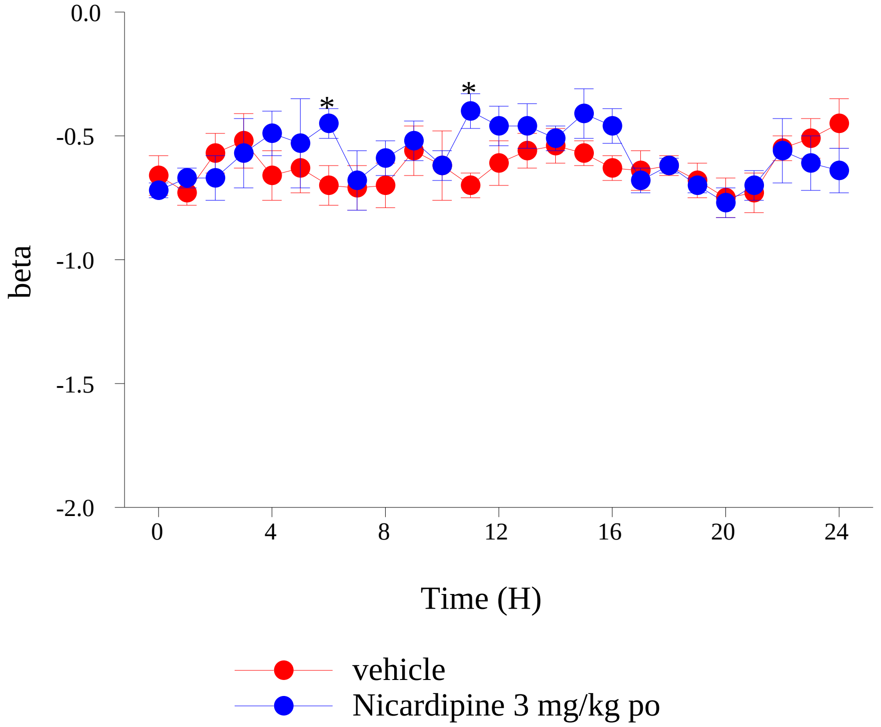

Results expressed in β
Repeated measures analysis of variance (RMANOVA)
Probability for Treatment factor: P=0.297
Probability for Time X Treatment interaction: P=0.426
$\star$: P$\leq$0.05 (LSD)
LSD=0.2 - Least significant difference for α type-1 error=5%
MDD=0.3 - Minimum detectable difference for α type-1 error=5% and β type-2 error=20% (*i.e.*power=80%)
Electronic authentication: created by Pascal Champéroux on 11-FEV-2025 at 14:47:42.787
Study QTOS

Nicardipine 30 mg/kg po

Figure 47 Nicardipine 30 mg/kg po

One-step QTc dQTc

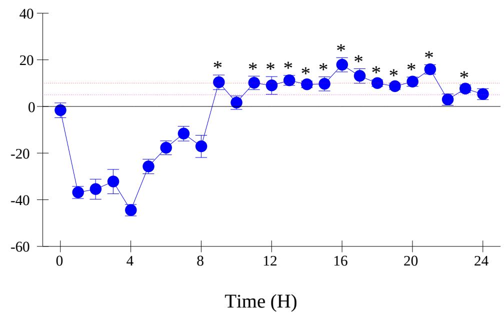

*: P<=0.05: one-sided 95% CI lower bound =>5ms
LSD= 2.9 ms

One-step QTc ddQTc

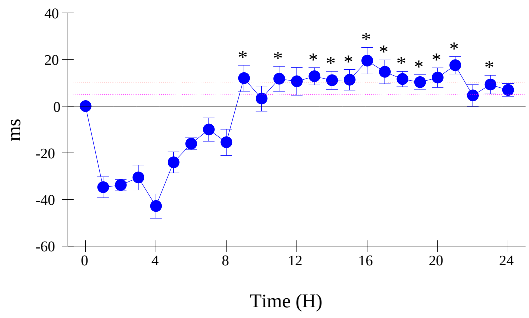

*: P<=0.05: one-sided 95% CI lower bound =>5ms
LSD= 4.5 ms

LogQTRR dQTc

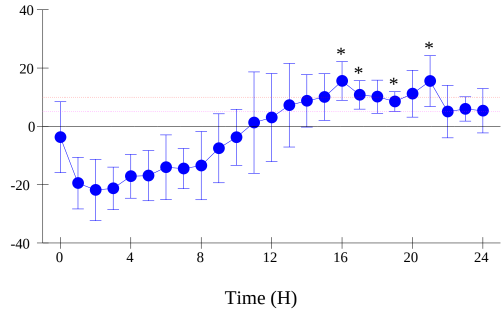

*: P<=0.05: one-sided 95% CI lower bound =>5ms
LSD= 8.7 ms

LogQTRR ddQTc

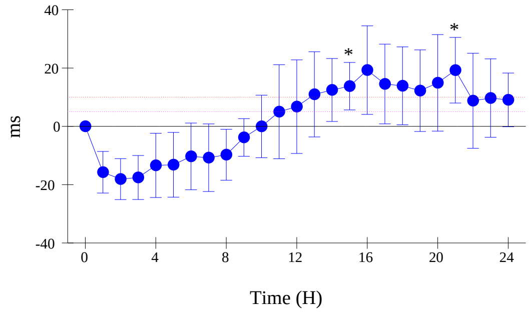

*: P<=0.05: one-sided 95% CI lower bound =>5ms
LSD= 11.3 ms

LinQTHR dQTc

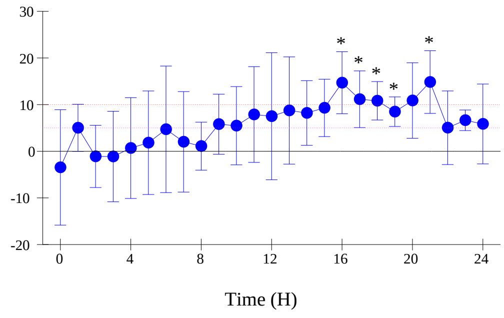

*: P<=0.05: one-sided 95% CI lower bound =>5ms
LSD= 7.9 ms

LinQTHR ddQTc

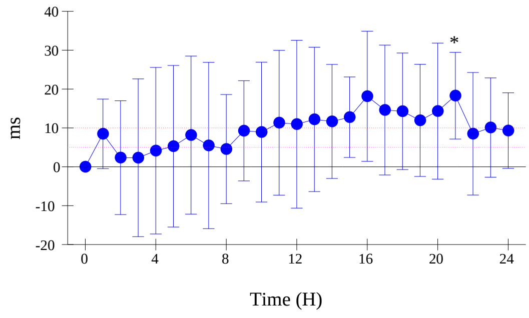

*: P<=0.05: one-sided 95% CI lower bound =>5ms
LSD= 15.8 ms

Figure 48 Nicardipine 30 mg/kg po - Effect on β slope (one step QTc model)

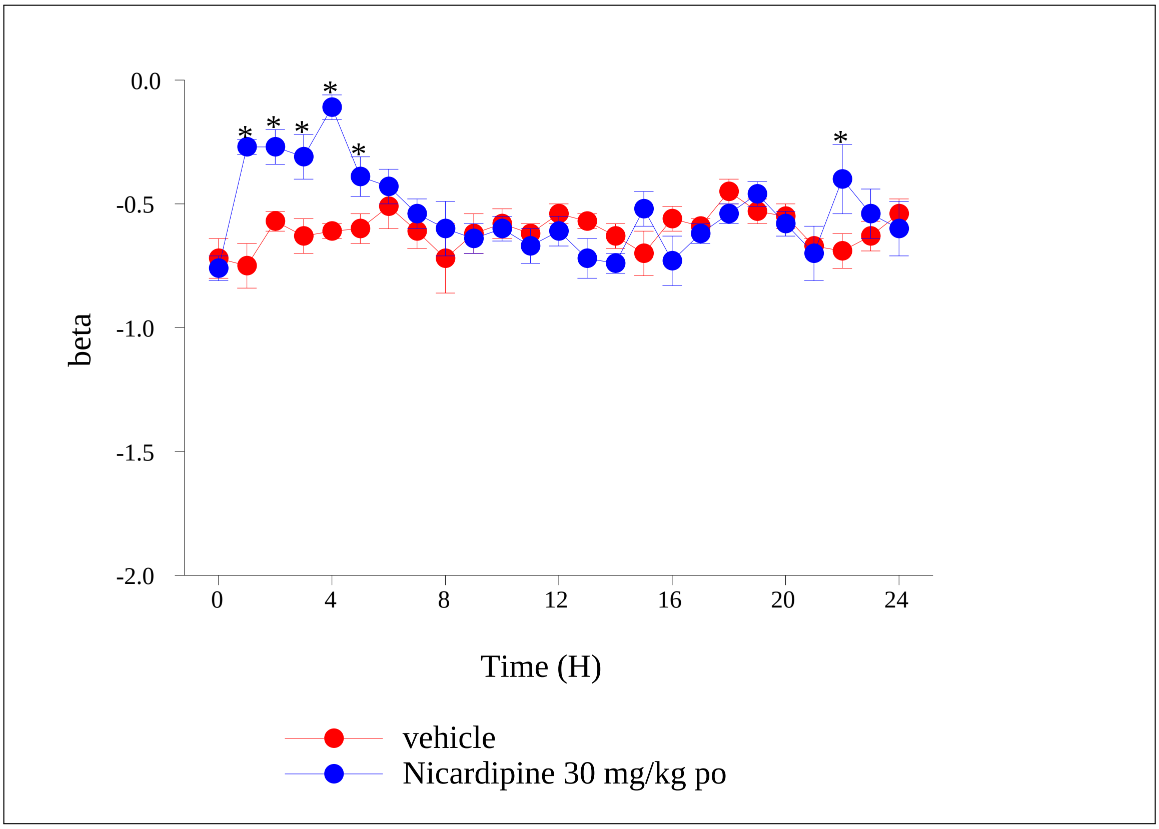

Results expressed in β
Repeated measures analysis of variance (RMANOVA)
Probability for Treatment factor: P=0.068
Probability for Time X Treatment interaction: P<0.001
$\star$: P$\leq$0.05 (LSD)
LSD=0.2 - Least significant difference for α type-1 error=5%
MDD=0.3 - Minimum detectable difference for α type-1 error=5% and β type-2 error=20% (*i.e.*power=80%)
Electronic authentication: created by Pascal Champéroux on 11-FEV-2025 at 14:47:42.982
Study QTOS

Phenylephrine 1 mg/kg po

Figure 49 Phenylephrine 1 mg/kg po

One-step QTc dQTc

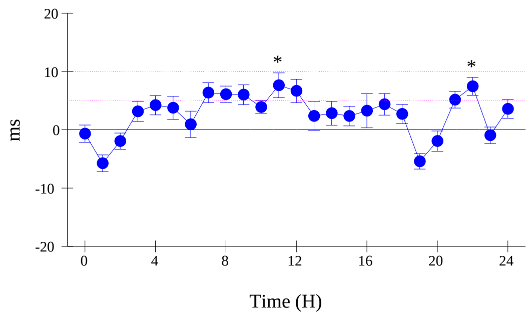

*: P<=0.05: one-sided 95% CI lower bound =>5ms
LSD= 1.7 ms

One-step QTc ddQTc

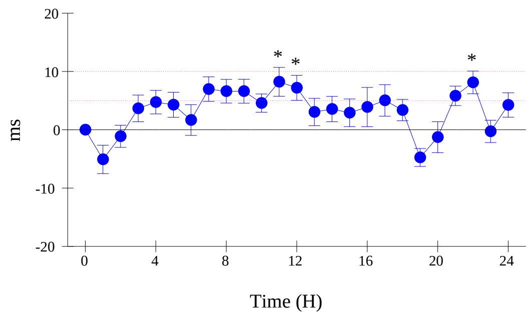

*: P<=0.05: one-sided 95% CI lower bound =>5ms
LSD= 2.1 ms

LogQTRR dQTc

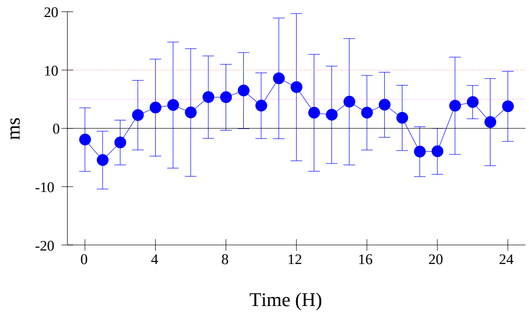

*: P<=0.05: one-sided 95% CI lower bound =>5ms
LSD= 6.4 ms

LogQTRR ddQTc

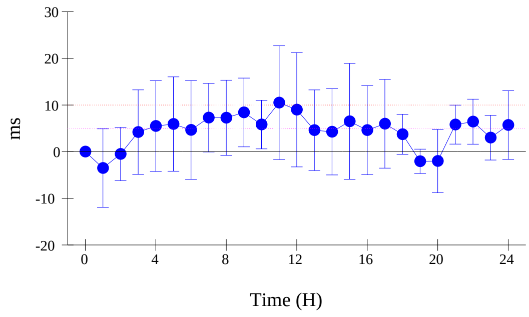

*: P<=0.05: one-sided 95% CI lower bound =>5ms
LSD= 8.1 ms

LinQTHR dQTc

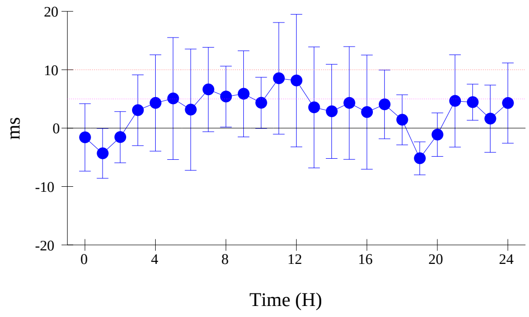

*: P<=0.05: one-sided 95% CI lower bound =>5ms
LSD= 6.9 ms

LinQTHR ddQTc

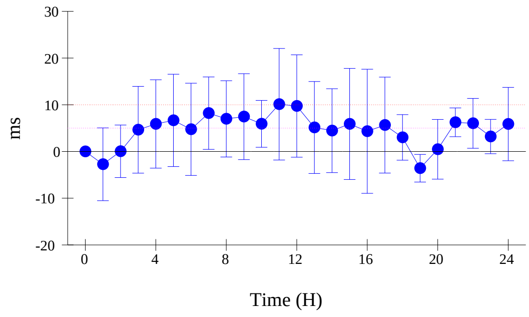

*: P<=0.05: one-sided 95% CI lower bound =>5ms
LSD= 8.2 ms

Figure 50 Phenylephrine 1 mg/kg po - Effect on β slope (one step QTc model)

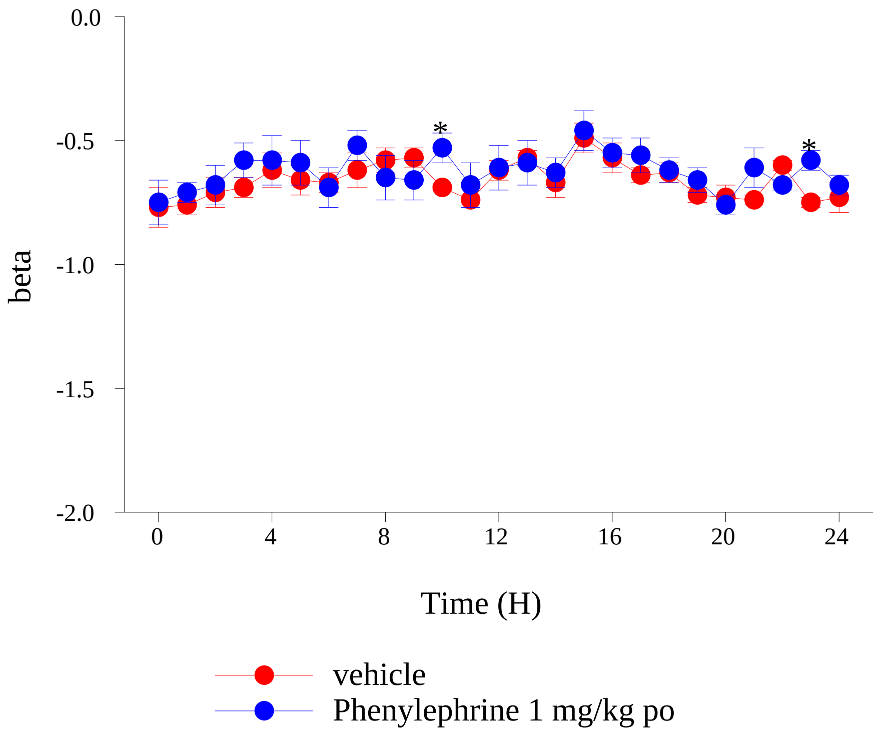

Results expressed in β
Repeated measures analysis of variance (RMANOVA)
Probability for Treatment factor: P=0.453
Probability for Time X Treatment interaction: P=0.59
$\star$: P$\leq$0.05 (LSD)
LSD=0.1 - Least significant difference for α type-1 error=5%
MDD=0.2 - Minimum detectable difference for α type-1 error=5% and β type-2 error=20% (*i.e.*power=80%)
Electronic authentication: created by Pascal Champéroux on 11-FEV-2025 at 14:47:43.158
Study QTOS

Phenytoin 100 mg/kg po

Figure 51 Phenytoin 100 mg/kg po

One-step QTc dQTc

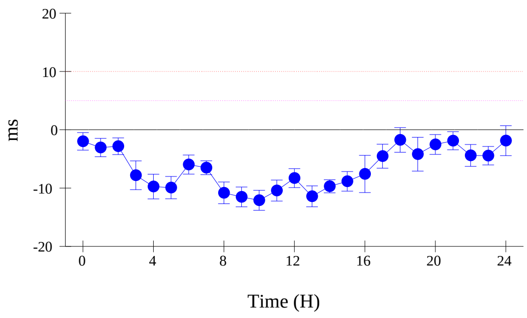

*: P<=0.05: one-sided 95% CI lower bound =>5ms
LSD= 1.7 ms

One-step QTc ddQTc

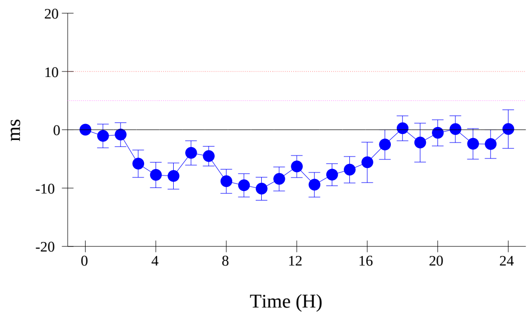

*: P<=0.05: one-sided 95% CI lower bound =>5ms
LSD= 2.1 ms

LogQTRR dQTc

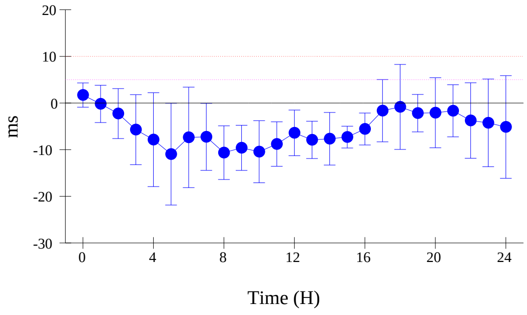

*: P<=0.05: one-sided 95% CI lower bound =>5ms
LSD= 5.8 ms

LogQTRR ddQTc

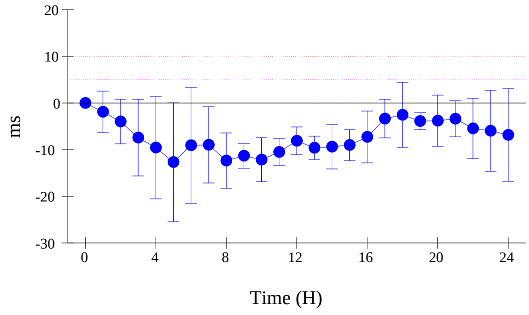

*: P<=0.05: one-sided 95% CI lower bound =>5ms
LSD= 4.8 ms

LinQTHR dQTc

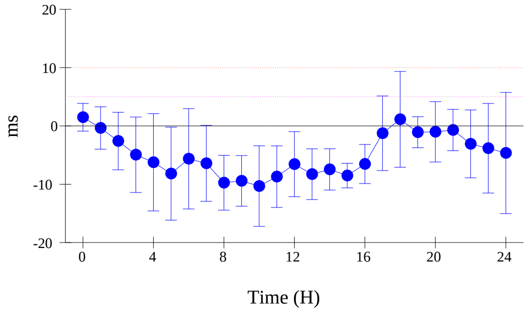

*: P<=0.05: one-sided 95% CI lower bound =>5ms
LSD= 5.3 ms

LinQTHR ddQTc

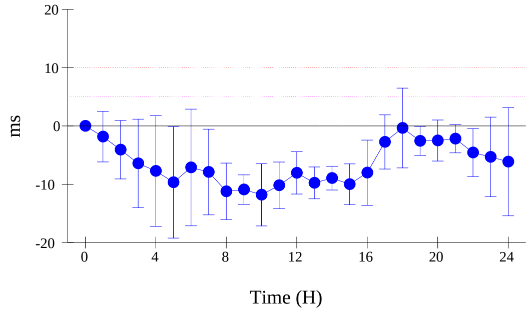

*: P<=0.05: one-sided 95% CI lower bound =>5ms
LSD= 4.6 ms

Figure 52 Phenytoin 100 mg/kg po - Effect on β slope (one step QTc model)

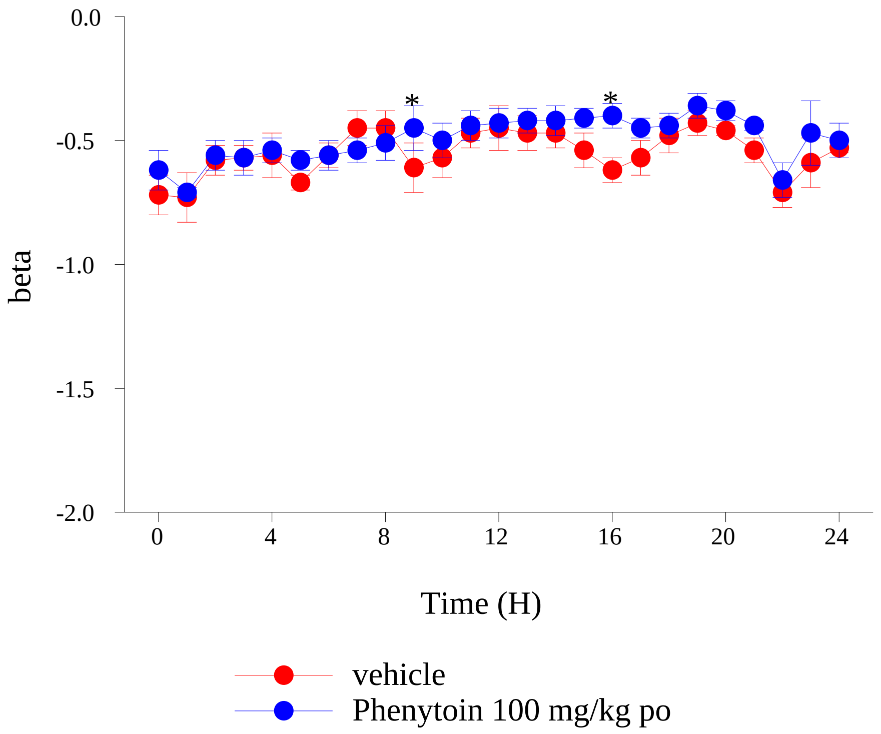

Results expressed in β
Repeated measures analysis of variance (RMANOVA)
Probability for Treatment factor: P=0.278
Probability for Time X Treatment interaction: P=0.79
$\star$: P$\leq$0.05 (LSD)
LSD=0.2 - Least significant difference for α type-1 error=5%
MDD=0.2 - Minimum detectable difference for α type-1 error=5% and β type-2 error=20% (*i.e.*power=80%)
Electronic authentication: created by Pascal Champéroux on 11-FEV-2025 at 14:47:43.318
Study QTOS

Pimozide 1 mg/kg iv

Figure 53 Pimozide 1 mg/kg iv

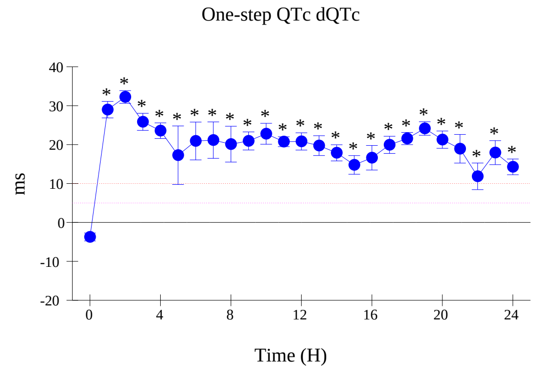

*: P<=0.05: one-sided 95% CI lower bound =>5ms
LSD= 2.2 ms

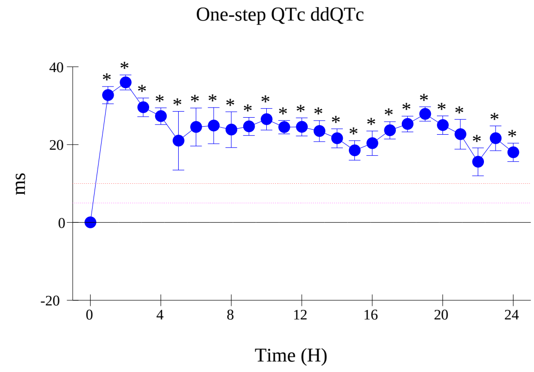

*: P<=0.05: one-sided 95% CI lower bound =>5ms
LSD= 2.4 ms

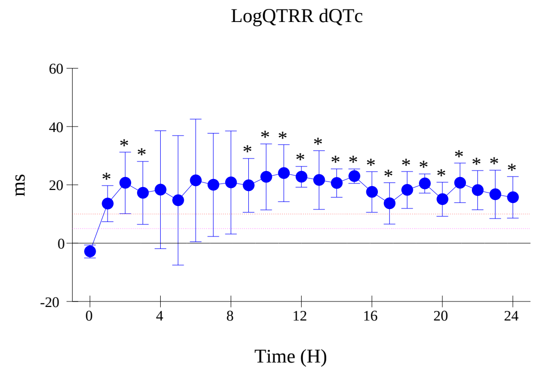

*: P<=0.05: one-sided 95% CI lower bound =>5ms
LSD= 7.1 ms

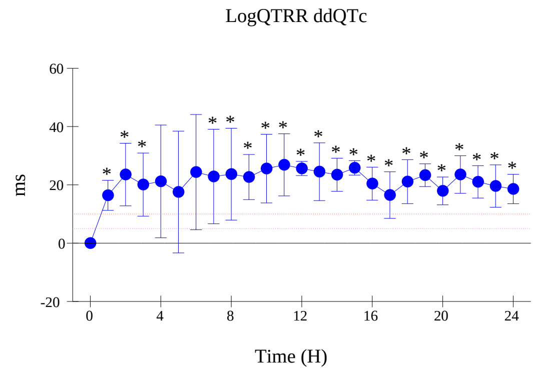

*: P<=0.05: one-sided 95% CI lower bound =>5ms
LSD= 7.5 ms

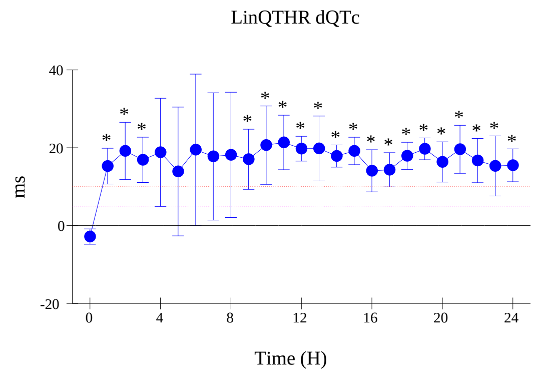

*: P<=0.05: one-sided 95% CI lower bound =>5ms
LSD= 5.8 ms

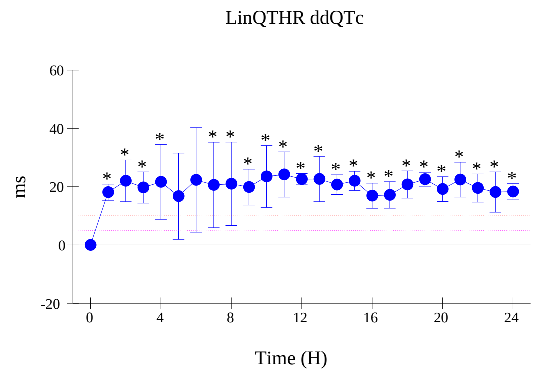

*: P<=0.05: one-sided 95% CI lower bound =>5ms
LSD= 5.3 ms

Figure 54 Pimozide 1 mg/kg iv - Effect on β slope (one step QTc model)

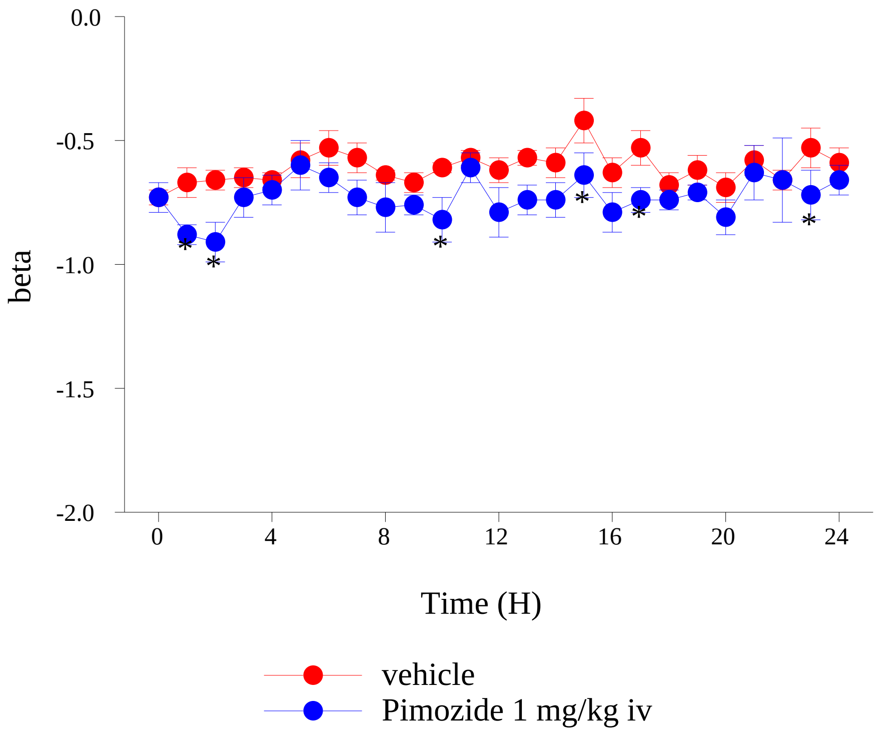

Results expressed in β
Repeated measures analysis of variance (RMANOVA)
Probability for Treatment factor: P=0.019
Probability for Time X Treatment interaction: P=0.806
$\star$: P$\leq$0.05 (LSD)
LSD=0.2 - Least significant difference for α type-1 error=5%
MDD=0.2 - Minimum detectable difference for α type-1 error=5% and β type-2 error=20% (*i.e.*power=80%)
Electronic authentication: created by Pascal Champéroux on 11-FEV-2025 at 14:47:43.497
Study QTOS

Prazosin 10 mg/kg po

Figure 55 Prazosin 10 mg/kg po

One-step QTc dQTc

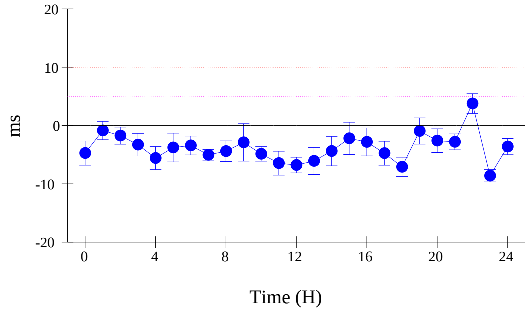

*: P<=0.05: one-sided 95% CI lower bound =>5ms
LSD= 1.9 ms

One-step QTc ddQTc

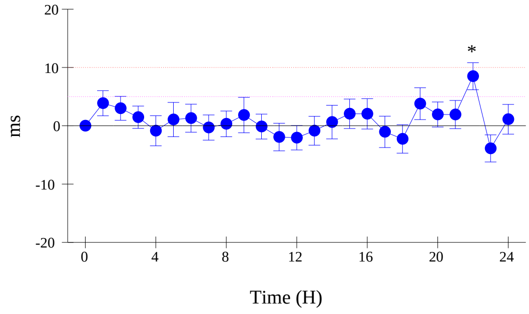

*: P<=0.05: one-sided 95% CI lower bound =>5ms
LSD= 2.4 ms

LogQTRR dQTc

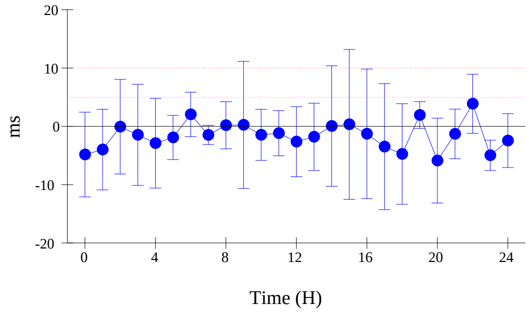

*: P<=0.05: one-sided 95% CI lower bound =>5ms
LSD= 6 ms

LogQTRR ddQTc

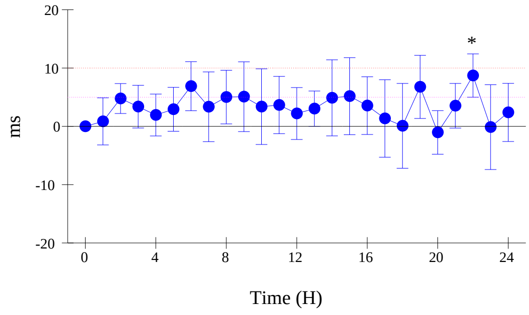

*: P<=0.05: one-sided 95% CI lower bound =>5ms
LSD= 4.6 ms

LinQTHR dQTc

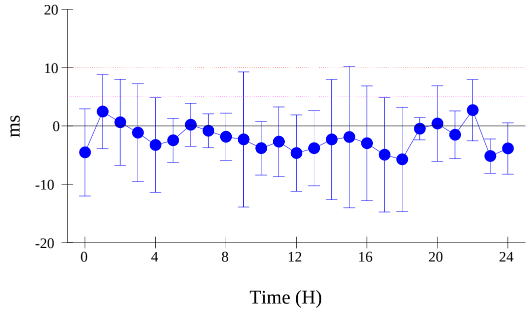

*: P<=0.05: one-sided 95% CI lower bound =>5ms
LSD= 6.4 ms

LinQTHR ddQTc

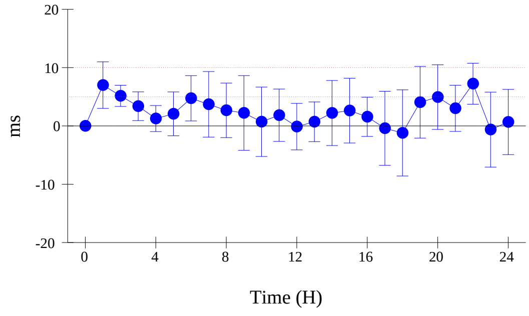

*: P<=0.05: one-sided 95% CI lower bound =>5ms
LSD= 4.5 ms

Figure 56 Prazosin 10 mg/kg po - Effect on β slope (one step QTc model)

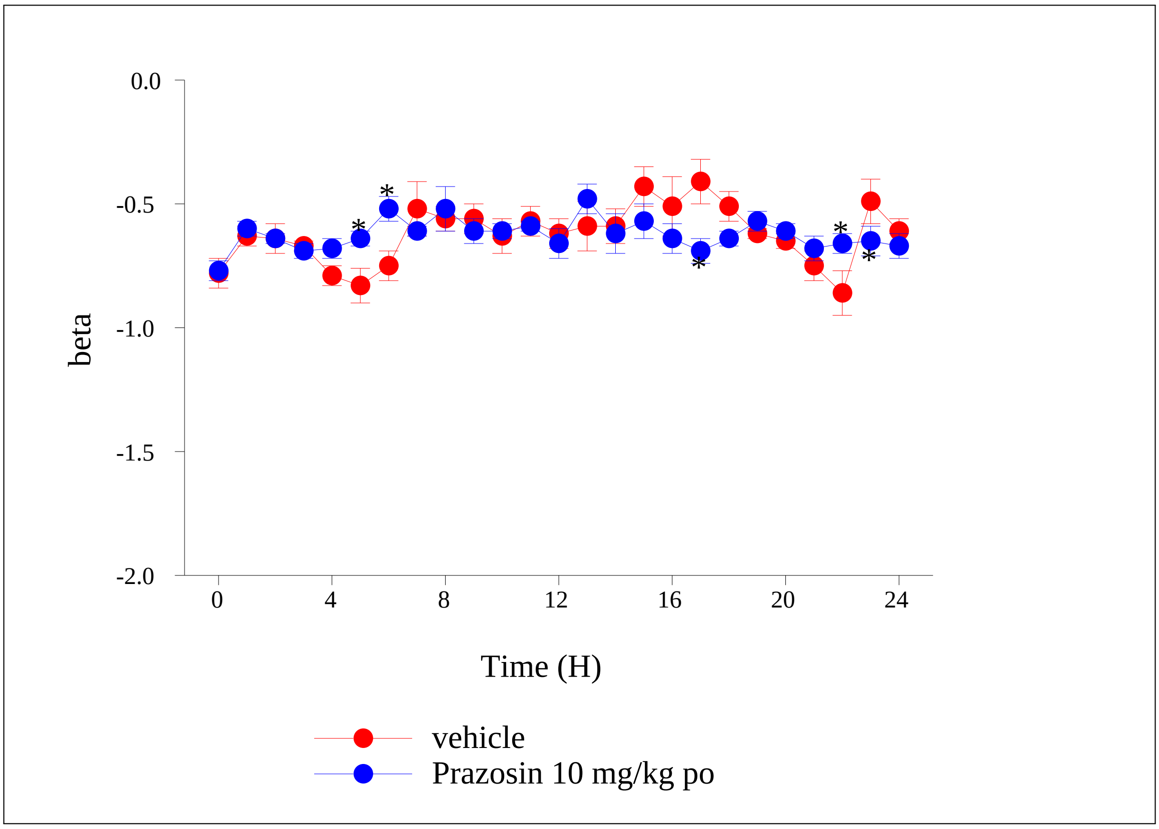

Results expressed in β
Repeated measures analysis of variance (RMANOVA)
Probability for Treatment factor: P=0.943
Probability for Time X Treatment interaction: P=0.001
$\star$: P$\leq$0.05 (LSD)
LSD=0.2 - Least significant difference for α type-1 error=5%
MDD=0.2 - Minimum detectable difference for α type-1 error=5% and β type-2 error=20% (*i.e.*power=80%)
Electronic authentication: created by Pascal Champéroux on 11-FEV-2025 at 14:47:43.689
Study QTOS

Procainamide 10 mg/kg iv

Figure 57 Procainamide 10 mg/kg iv

One-step QTc dQTc

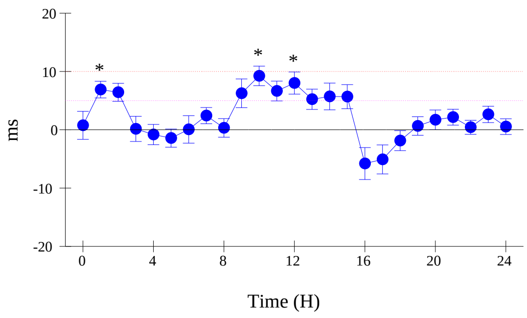

*: P<=0.05: one-sided 95% CI lower bound =>5ms
LSD= 1.7 ms

One-step QTc ddQTc

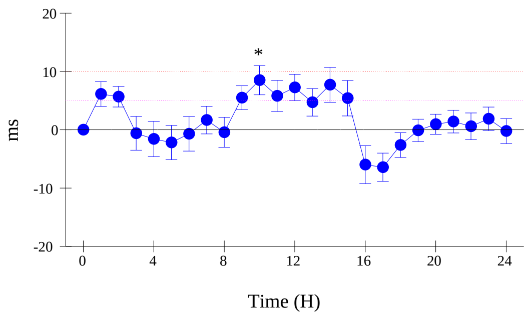

*: P<=0.05: one-sided 95% CI lower bound =>5ms
LSD= 2.4 ms

LogQTRR dQTc

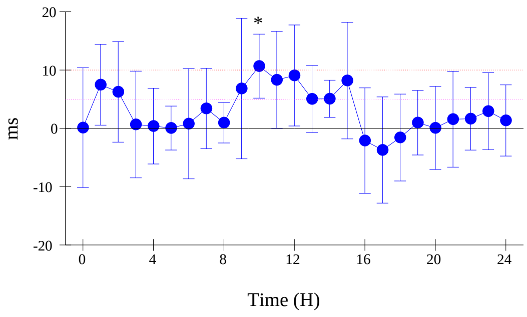

*: P<=0.05: one-sided 95% CI lower bound =>5ms
LSD= 7.1 ms

LogQTRR ddQTc

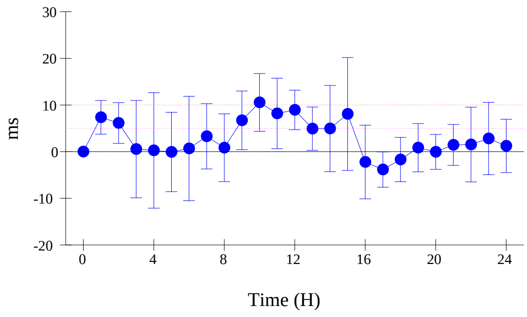

*: P<=0.05: one-sided 95% CI lower bound =>5ms
LSD= 6.3 ms

LinQTHR dQTc

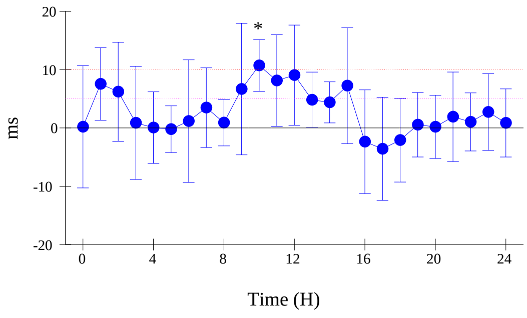

*: P<=0.05: one-sided 95% CI lower bound =>5ms
LSD= 6.8 ms

LinQTHR ddQTc

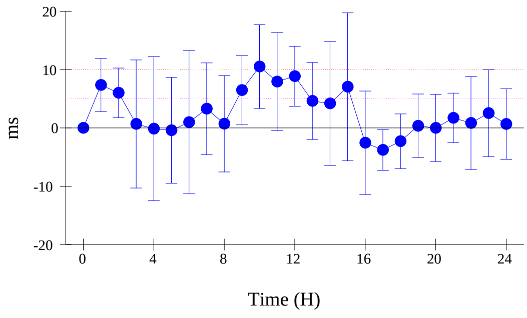

*: P<=0.05: one-sided 95% CI lower bound =>5ms
LSD= 7.2 ms

Figure 58 Procainamide 10 mg/kg iv - Effect on β slope (one step QTc model)

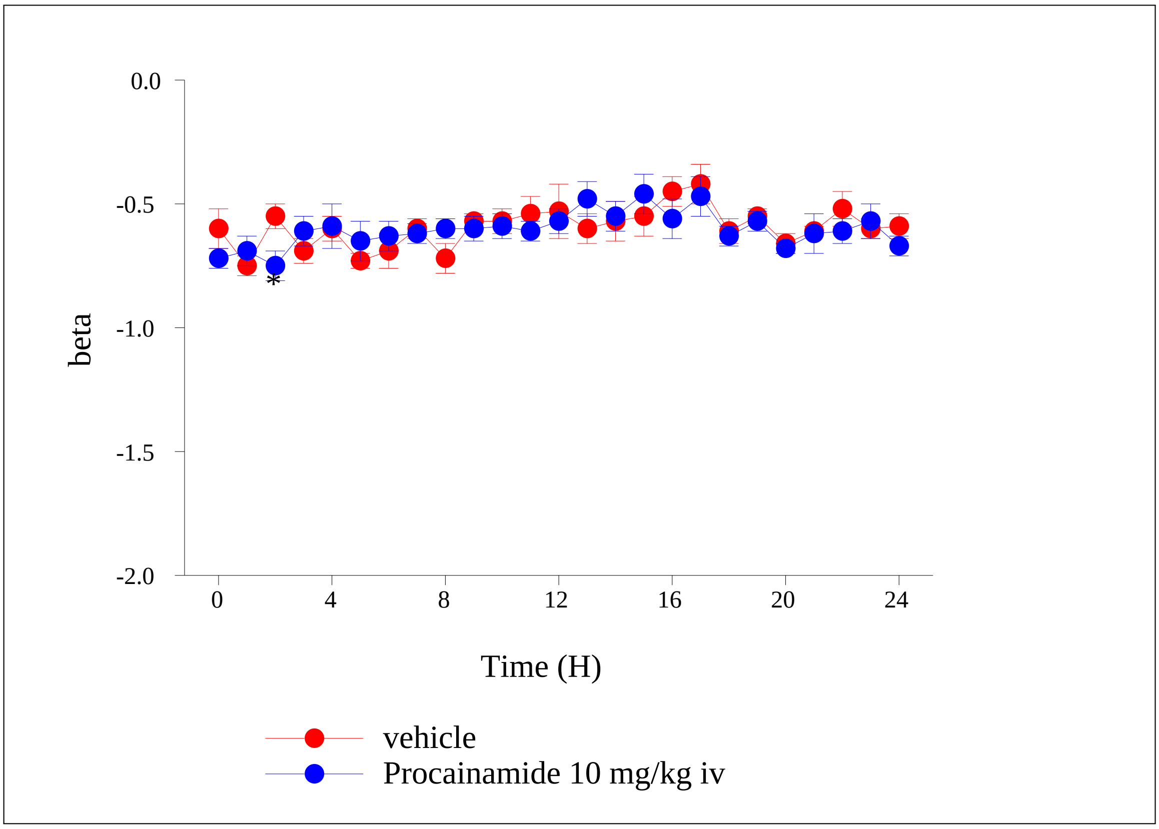

Results expressed in β
Repeated measures analysis of variance (RMANOVA)
Probability for Treatment factor: P=0.794
Probability for Time X Treatment interaction: P=0.446
$\star$: P$\leq$0.05 (LSD)
LSD=0.2 - Least significant difference for α type-1 error=5%
MDD=0.2 - Minimum detectable difference for α type-1 error=5% and β type-2 error=20% (*i.e.*power=80%)
Electronic authentication: created by Pascal Champéroux on 11-FEV-2025 at 14:47:43.972
Study QTOS

Procainamide 30 mg/kg iv

Figure 59 Procainamide 30 mg/kg iv

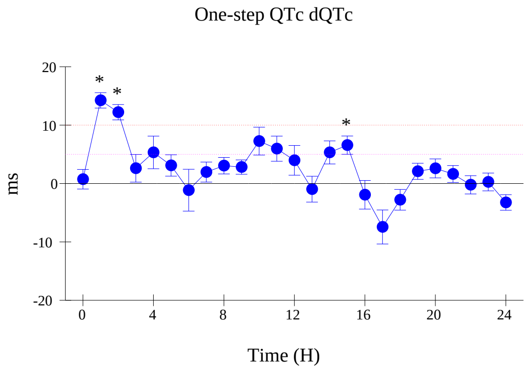

*: P<=0.05: one-sided 95% CI lower bound =>5ms
LSD= 1.7 ms

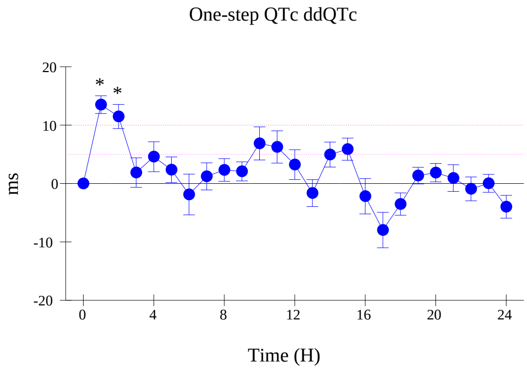

*: P<=0.05: one-sided 95% CI lower bound =>5ms
LSD= 2.1 ms

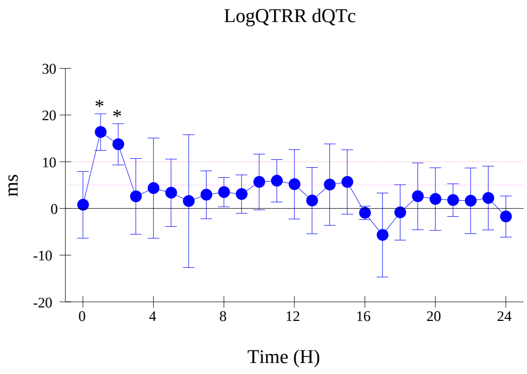

*: P<=0.05: one-sided 95% CI lower bound =>5ms
LSD= 6.8 ms

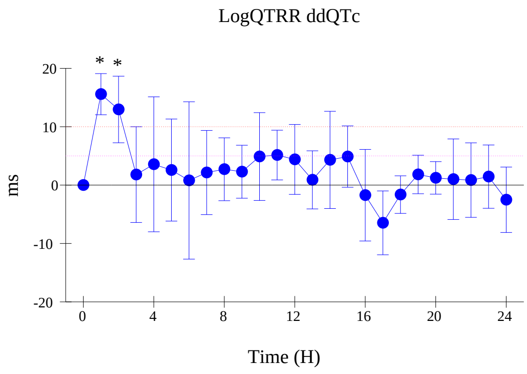

*: P<=0.05: one-sided 95% CI lower bound =>5ms
LSD= 5.6 ms

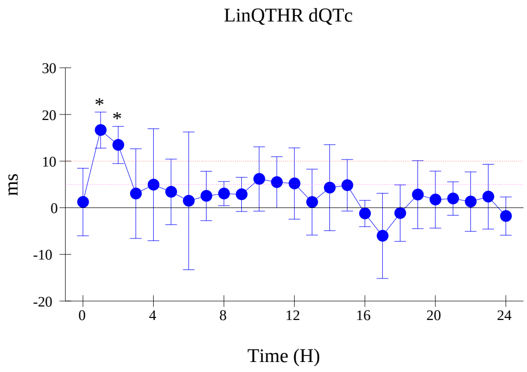

*: P<=0.05: one-sided 95% CI lower bound =>5ms
LSD= 6.4 ms

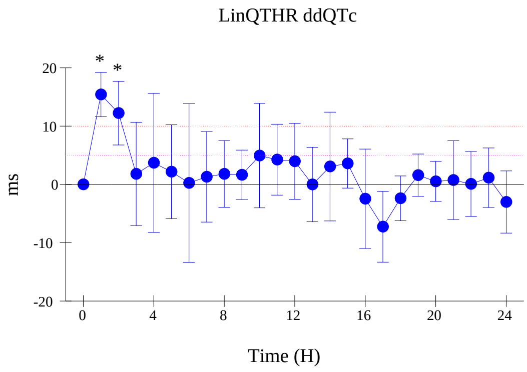

*: P<=0.05: one-sided 95% CI lower bound =>5ms
LSD= 6.1 ms

Figure 60 Procainamide 30 mg/kg iv - Effect on β slope (one step QTc model)

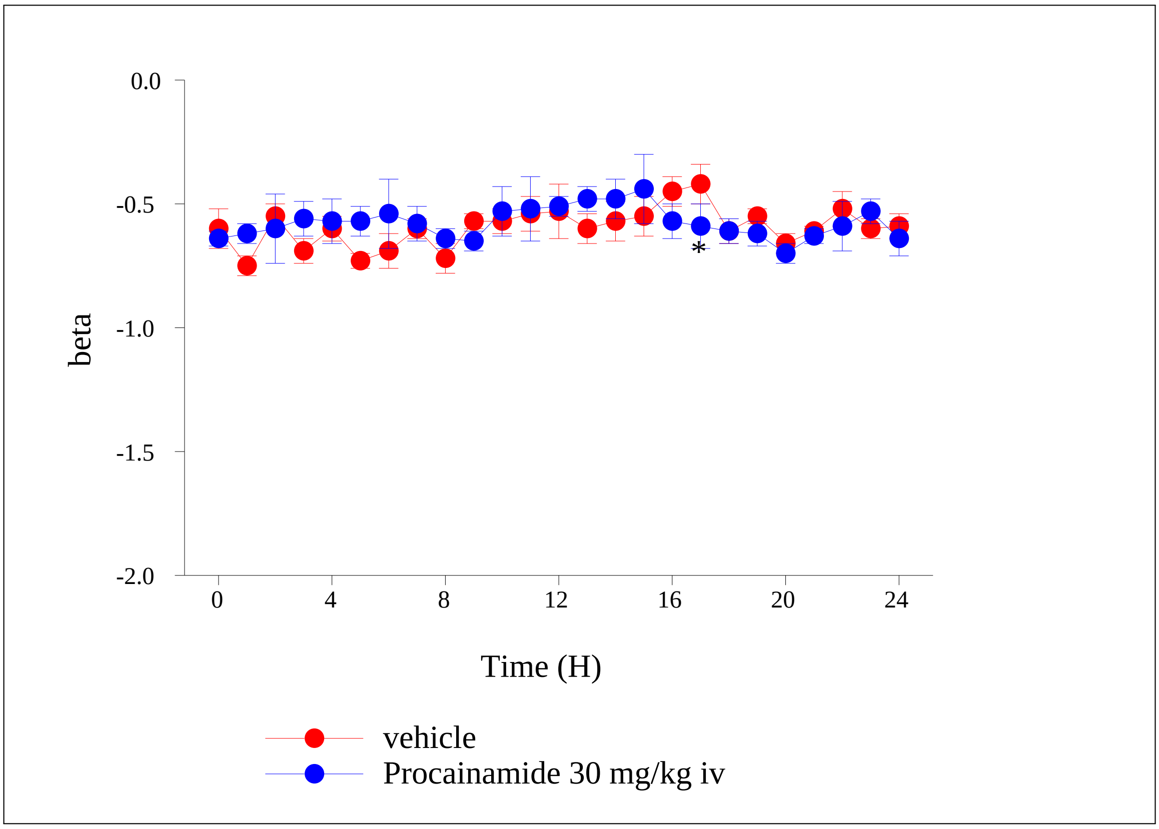

Results expressed in β
Repeated measures analysis of variance (RMANOVA)
Probability for Treatment factor: P=0.733
Probability for Time X Treatment interaction: P=0.381
$\star$: P$\leq$0.05 (LSD)
LSD=0.2 - Least significant difference for α type-1 error=5%
MDD=0.2 - Minimum detectable difference for α type-1 error=5% and β type-2 error=20% (*i.e.*power=80%)
Electronic authentication: created by Pascal Champéroux on 11-FEV-2025 at 14:47:44.194
Study QTOS

Quinidine 3 mg/kg po

Figure 61 Quinidine 3 mg/kg po

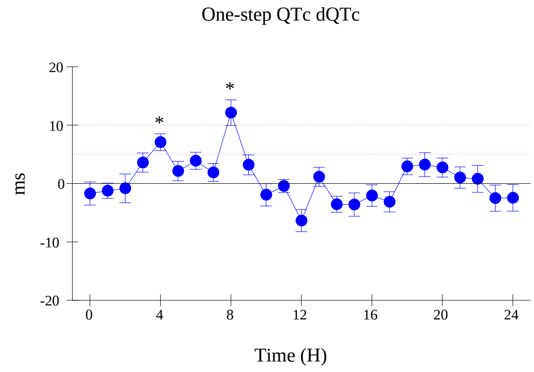

*: P<=0.05: one-sided 95% CI lower bound =>5ms
LSD= 1.7 ms

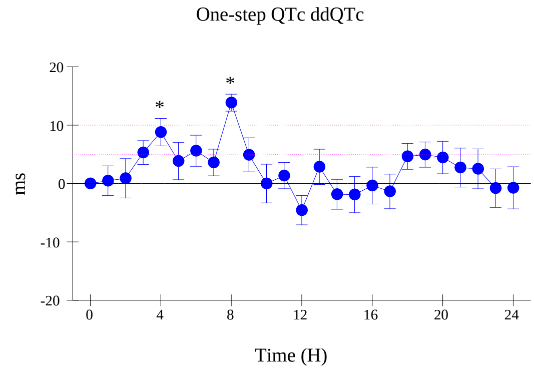

*: P<=0.05: one-sided 95% CI lower bound =>5ms
LSD= 2.8 ms

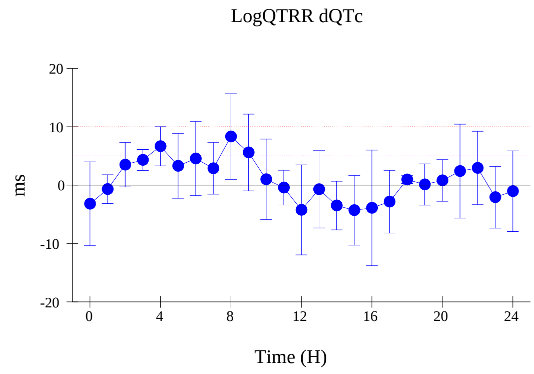

*: P<=0.05: one-sided 95% CI lower bound =>5ms
LSD= 5.5 ms

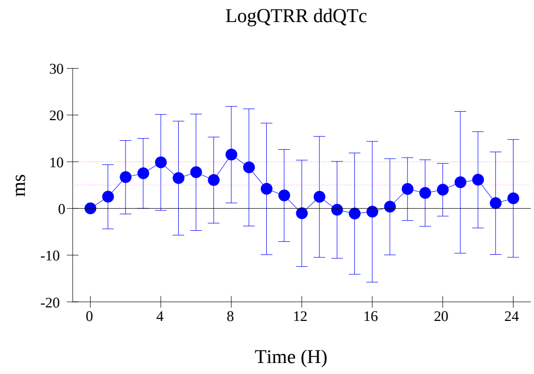

*: P<=0.05: one-sided 95% CI lower bound =>5ms
LSD= 10.3 ms

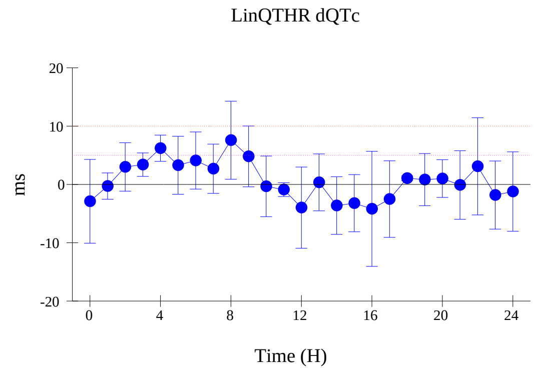

*: P<=0.05: one-sided 95% CI lower bound =>5ms
LSD= 4.9 ms

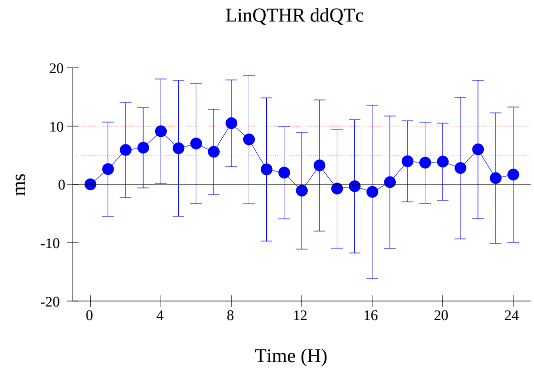

*: P<=0.05: one-sided 95% CI lower bound =>5ms
LSD= 10.2 ms

Figure 62 Quinidine 3 mg/kg po - Effect on β slope (one step QTc model)

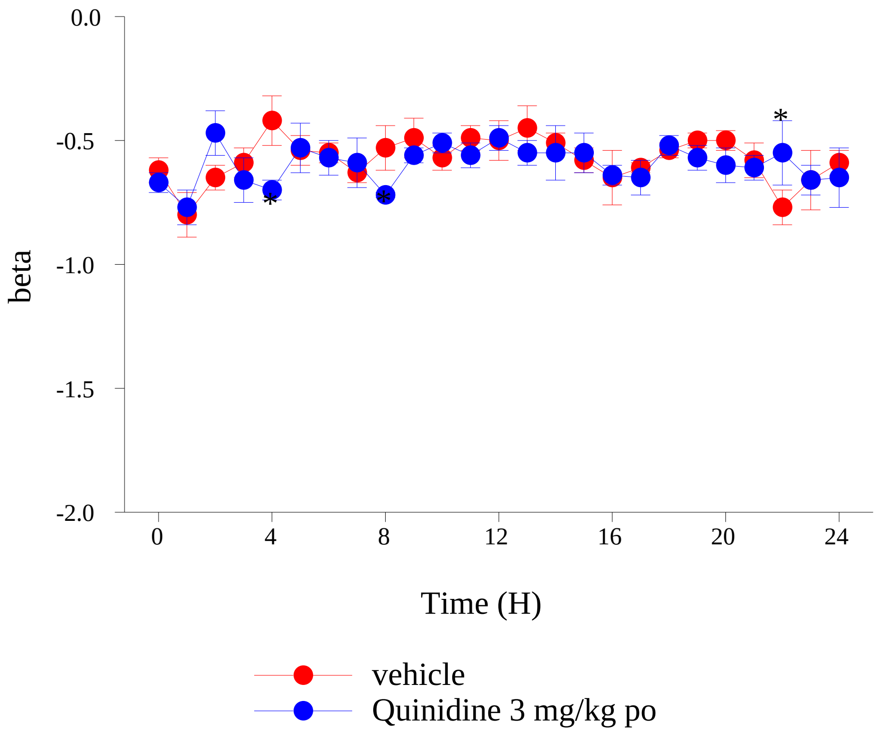

Results expressed in β
Repeated measures analysis of variance (RMANOVA)
Probability for Treatment factor: P=0.449
Probability for Time X Treatment interaction: P=0.362
$\star$: P$\leq$0.05 (LSD)
LSD=0.2 - Least significant difference for α type-1 error=5%
MDD=0.3 - Minimum detectable difference for α type-1 error=5% and β type-2 error=20% (*i.e.*power=80%)
Electronic authentication: created by Pascal Champéroux on 11-FEV-2025 at 14:47:44.367
Study QTOS

Quinidine 10 mg/kg po

Figure 63 Quinidine 10 mg/kg po

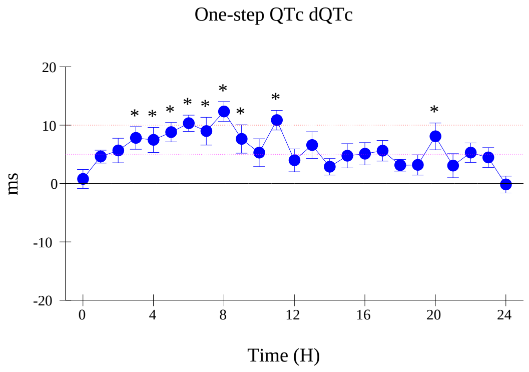

*: P<=0.05: one-sided 95% CI lower bound =>5ms
LSD= 1.8 ms

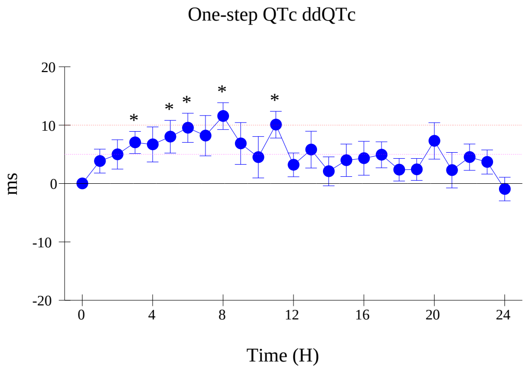

*: P<=0.05: one-sided 95% CI lower bound =>5ms
LSD= 2.5 ms

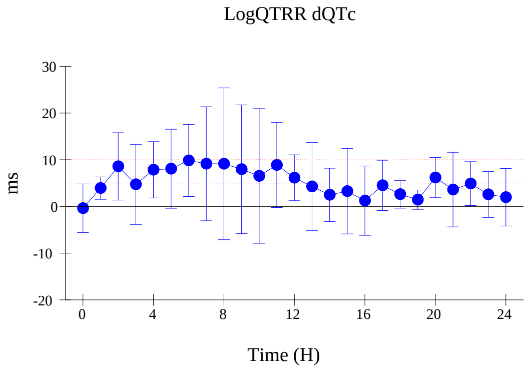

*: P<=0.05: one-sided 95% CI lower bound =>5ms
LSD= 7.2 ms

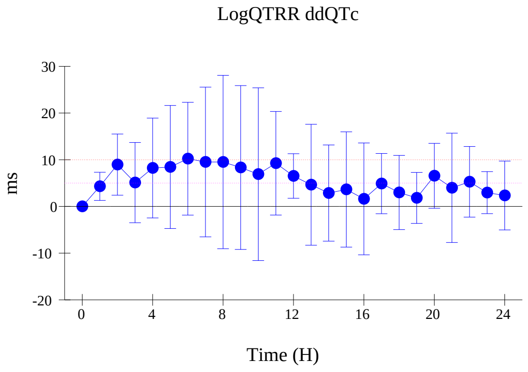

*: P<=0.05: one-sided 95% CI lower bound =>5ms
LSD= 10.3 ms

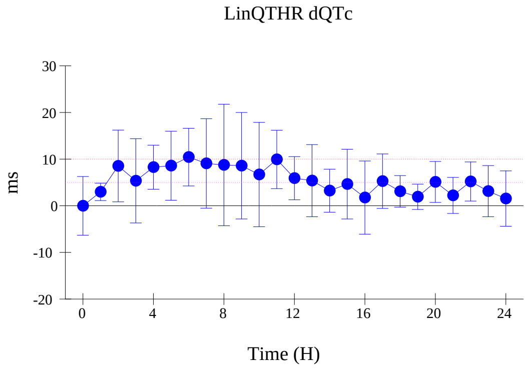

*: P<=0.05: one-sided 95% CI lower bound =>5ms
LSD= 6.2 ms

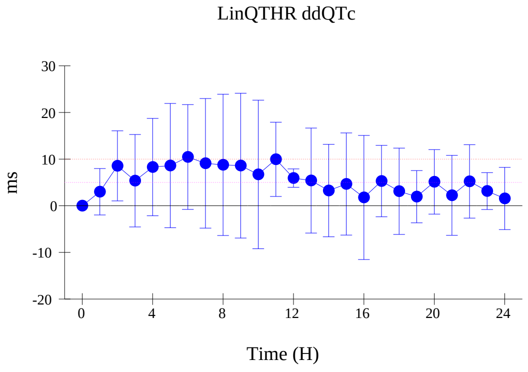

*: P<=0.05: one-sided 95% CI lower bound =>5ms
LSD= 9.3 ms

Figure 64 Quinidine 10 mg/kg po - Effect on β slope (one step QTc model)

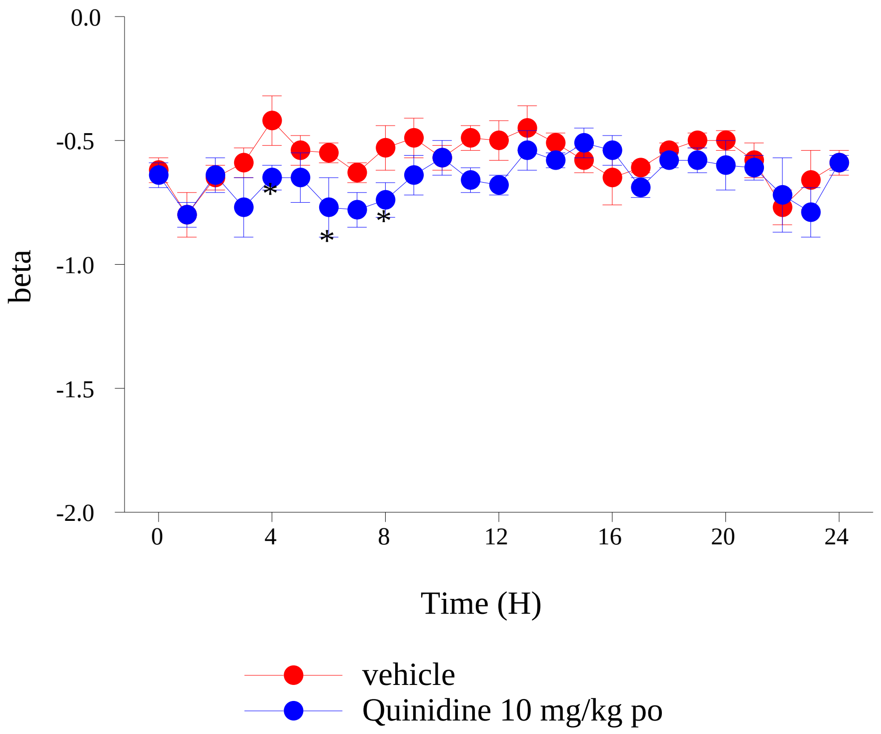

Results expressed in β
Repeated measures analysis of variance (RMANOVA)
Probability for Treatment factor: P=0.079
Probability for Time X Treatment interaction: P=0.412
$\star$: P$\leq$0.05 (LSD)
LSD=0.2 - Least significant difference for α type-1 error=5%
MDD=0.3 - Minimum detectable difference for α type-1 error=5% and β type-2 error=20% (*i.e.*power=80%)
Electronic authentication: created by Pascal Champéroux on 11-FEV-2025 at 14:47:44.544
Study QTOS

Quinidine 30 mg/kg po

Figure 65 Quinidine 30 mg/kg po

One-step QTc dQTc

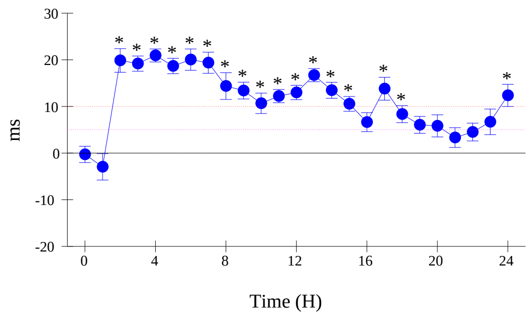

*: P<=0.05: one-sided 95% CI lower bound =>5ms
LSD= 1.9 ms

One-step QTc ddQTc

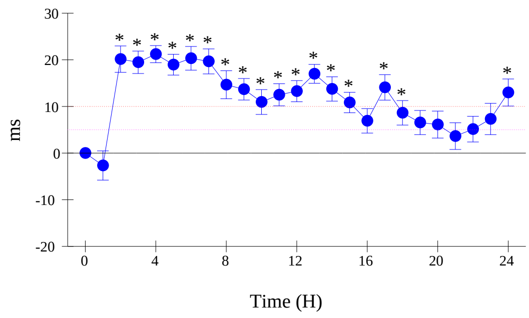

*: P<=0.05: one-sided 95% CI lower bound =>5ms
LSD= 2.6 ms

LogQTRR dQTc

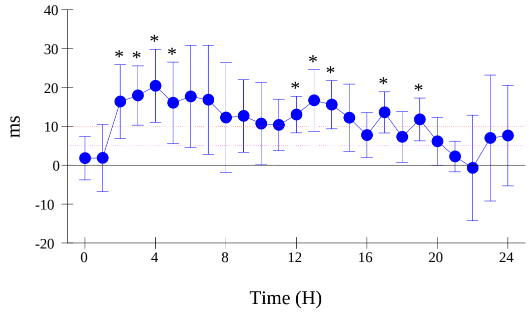

*: P<=0.05: one-sided 95% CI lower bound =>5ms
LSD= 8.6 ms

LogQTRR ddQTc

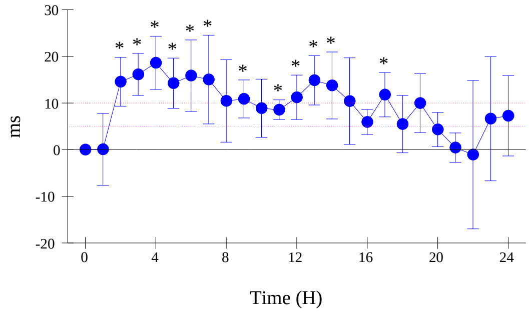

*: P<=0.05: one-sided 95% CI lower bound =>5ms
LSD= 5.7 ms

LinQTHR dQTc

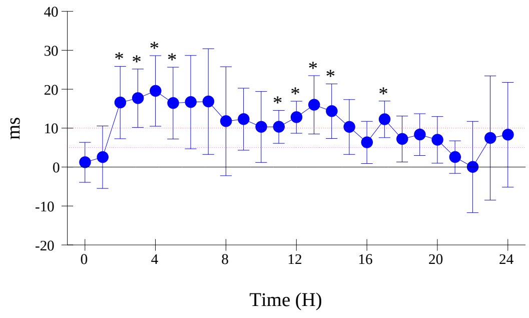

*: P<=0.05: one-sided 95% CI lower bound =>5ms
LSD= 7.5 ms

LinQTHR ddQTc

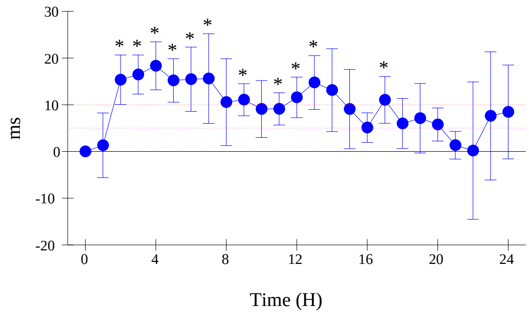

*: P<=0.05: one-sided 95% CI lower bound =>5ms
LSD= 5.4 ms

Figure 66 Quinidine 30 mg/kg po - Effect on β slope (one step QTc model)

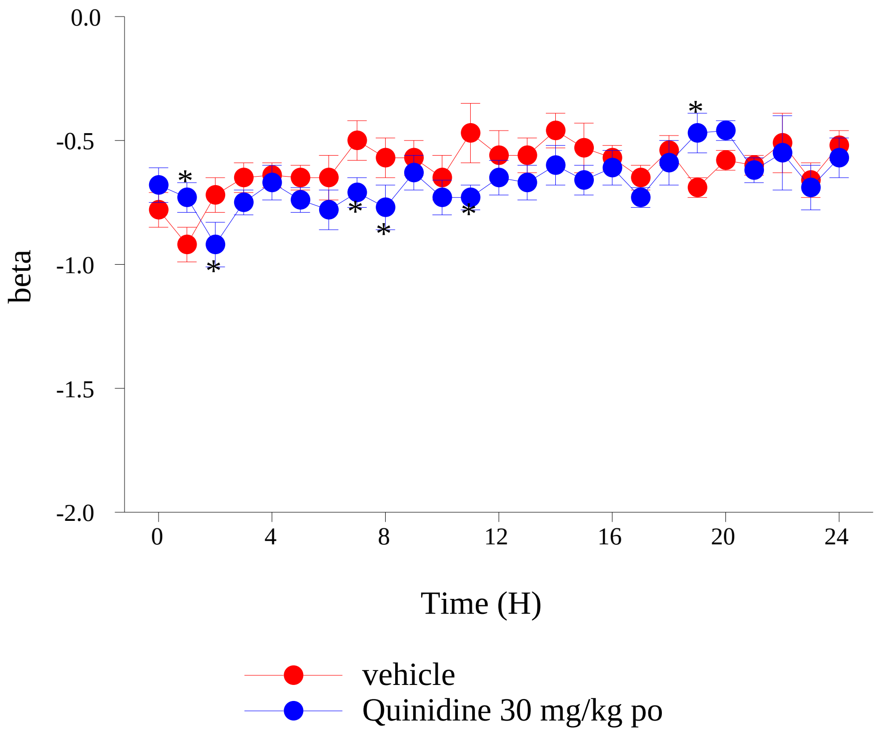

Results expressed in β
Repeated measures analysis of variance (RMANOVA)
Probability for Treatment factor: P=0.315
Probability for Time X Treatment interaction: P=0.019
$\star$: P$\leq$0.05 (LSD)
LSD=0.2 - Least significant difference for α type-1 error=5%
MDD=0.2 - Minimum detectable difference for α type-1 error=5% and β type-2 error=20% (*i.e.*power=80%)
Electronic authentication: created by Pascal Champéroux on 11-FEV-2025 at 14:47:44.791
Study QTOS

Ranolazine 50 mg/kg po

Figure 67 Ranolazine 50 mg/kg po

One-step QTc dQTc

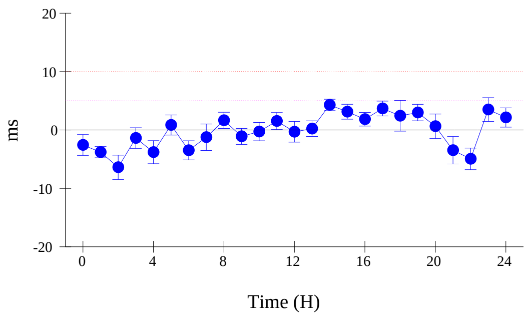

*: P<=0.05: one-sided 95% CI lower bound =>5ms
LSD= 1.6 ms

One-step QTc ddQTc

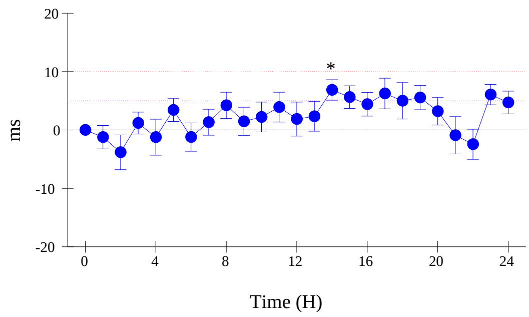

*: P<=0.05: one-sided 95% CI lower bound =>5ms
LSD= 2.4 ms

LogQTRR dQTc

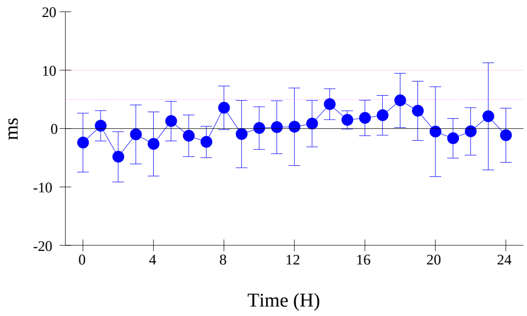

*: P<=0.05: one-sided 95% CI lower bound =>5ms
LSD= 4.1 ms

LogQTRR ddQTc

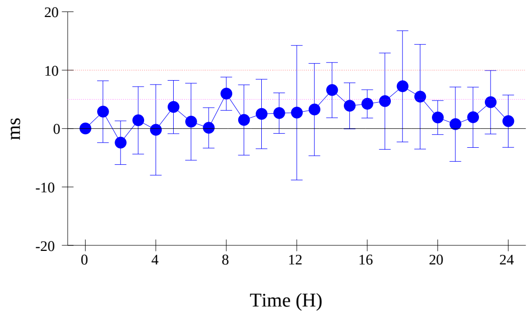

*: P<=0.05: one-sided 95% CI lower bound =>5ms
LSD= 5.3 ms

LinQTHR dQTc

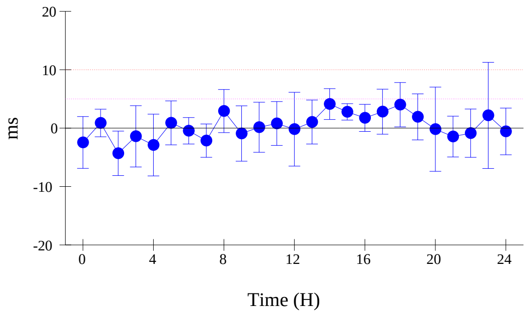

*: P<=0.05: one-sided 95% CI lower bound =>5ms
LSD= 3.8 ms

LinQTHR ddQTc

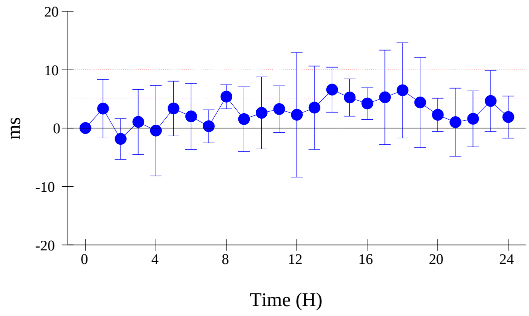

*: P<=0.05: one-sided 95% CI lower bound =>5ms
LSD= 5 ms

Figure 68 Ranolazine 50 mg/kg po - Effect on β slope (one step QTc model)

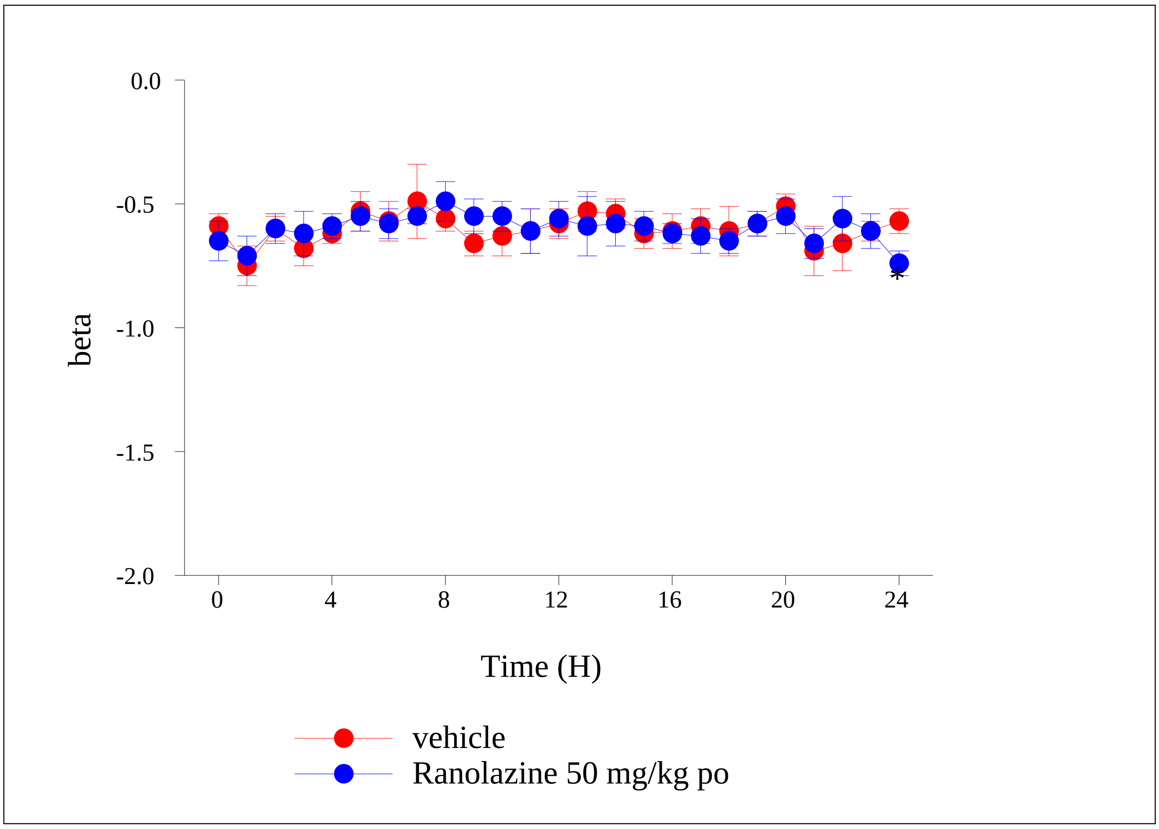

Results expressed in β
Repeated measures analysis of variance (RMANOVA)
Probability for Treatment factor: P=0.994
Probability for Time X Treatment interaction: P=0.964
$\star$: P$\leq$0.05 (LSD)
LSD=0.2 - Least significant difference for α type-1 error=5%
MDD=0.2 - Minimum detectable difference for α type-1 error=5% and β type-2 error=20% (*i.e.*power=80%)
Electronic authentication: created by Pascal Champéroux on 11-FEV-2025 at 14:47:44.975
Study QTOS

Risperidone 1 mg/kg iv

Figure 69 Risperidone 1 mg/kg iv

One-step QTc dQTc

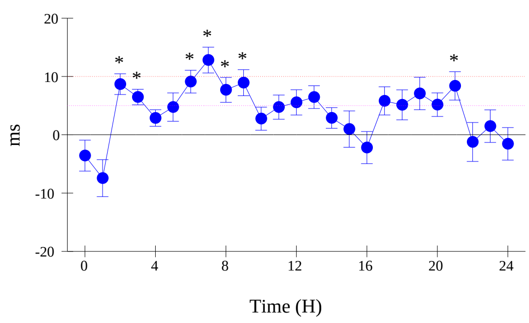

*: P<=0.05: one-sided 95% CI lower bound =>5ms
LSD= 2.2 ms

One-step QTc ddQTc

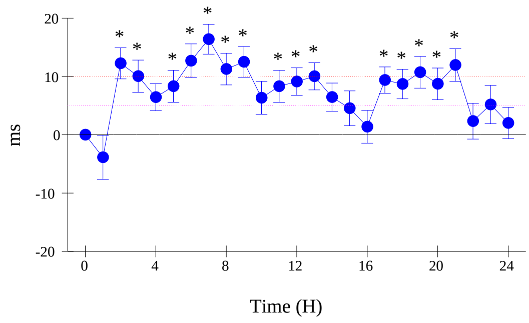

*: P<=0.05: one-sided 95% CI lower bound =>5ms
LSD= 2.7 ms

LogQTRR dQTc

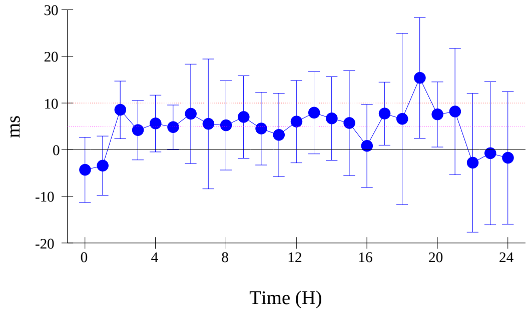

*: P<=0.05: one-sided 95% CI lower bound =>5ms
LSD= 8.9 ms

LogQTRR ddQTc

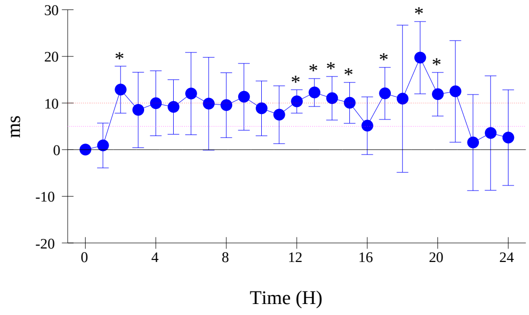

*: P<=0.05: one-sided 95% CI lower bound =>5ms
LSD= 6.2 ms

LinQTHR dQTc

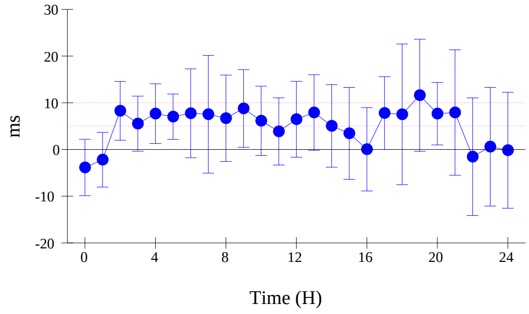

*: P<=0.05: one-sided 95% CI lower bound =>5ms
LSD= 8.3 ms

LinQTHR ddQTc

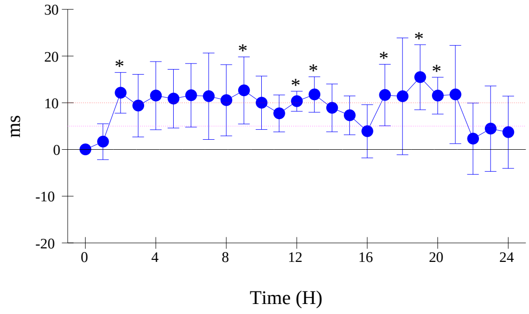

*: P<=0.05: one-sided 95% CI lower bound =>5ms
LSD= 6.6 ms

Figure 70 Risperidone 1 mg/kg iv - Effect on β slope (one step QTc model)

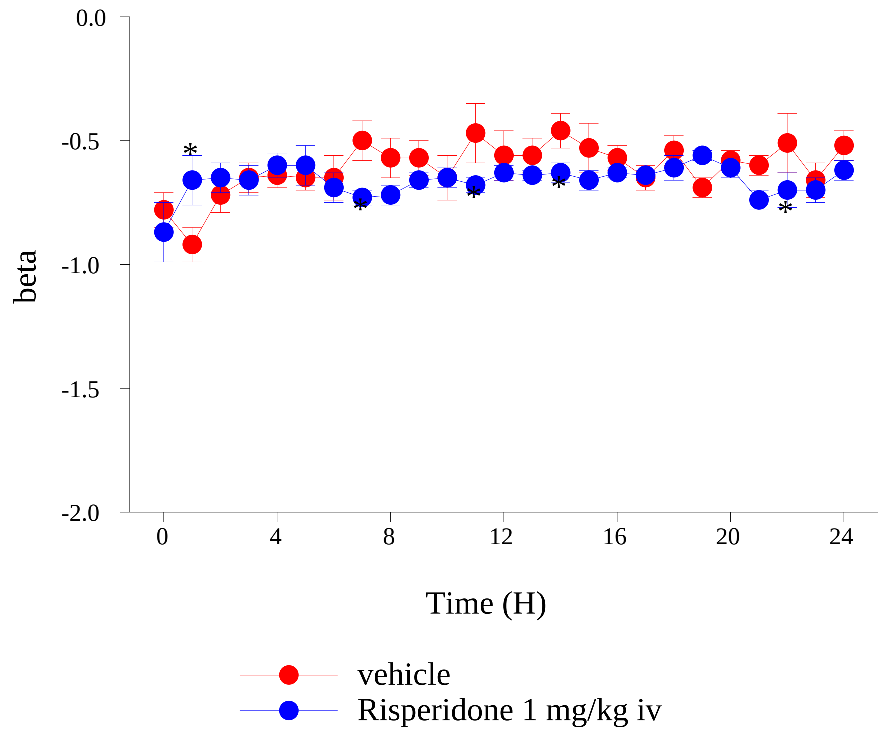

Results expressed in β
Repeated measures analysis of variance (RMANOVA)
Probability for Treatment factor: P=0.271
Probability for Time X Treatment interaction: P=0.01
$\star$: P$\leq$0.05 (LSD)
LSD=0.2 - Least significant difference for α type-1 error=5%
MDD=0.2 - Minimum detectable difference for α type-1 error=5% and β type-2 error=20% (*i.e.*power=80%)
Electronic authentication: created by Pascal Champéroux on 11-FEV-2025 at 14:47:45.165
Study QTOS

Risperidone 1 mg/kg iv + atenolol

Figure 71 Risperidone 1 mg/kg iv + atenolol

One-step QTc dQTc

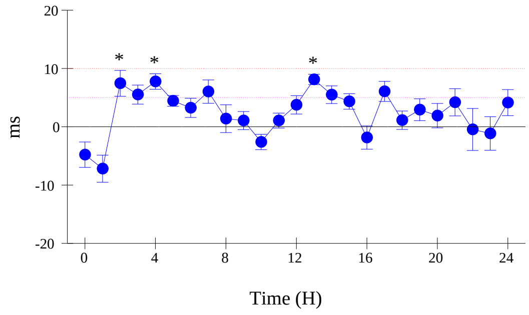

*: P<=0.05: one-sided 95% CI lower bound =>5ms
LSD= 1.7 ms

One-step QTc ddQTc

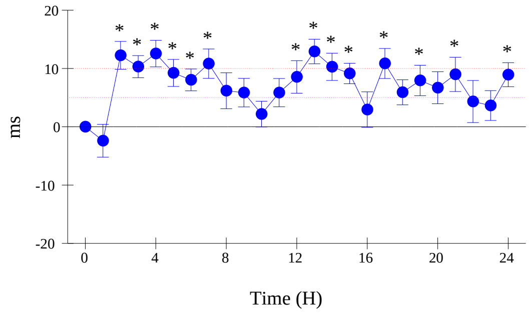

*: P<=0.05: one-sided 95% CI lower bound =>5ms
LSD= 2.4 ms

LogQTRR dQTc

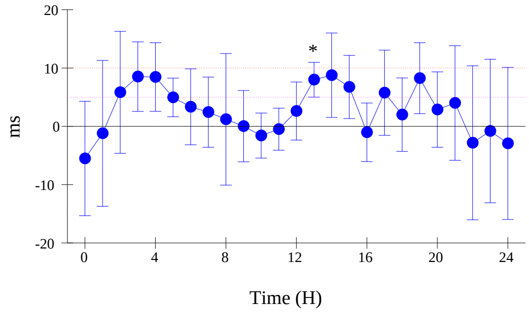

*: P<=0.05: one-sided 95% CI lower bound =>5ms
LSD= 6.3 ms

LogQTRR ddQTc

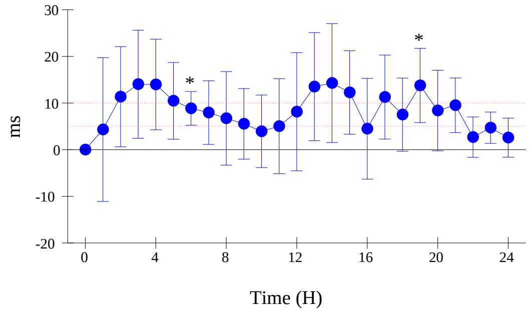

*: P<=0.05: one-sided 95% CI lower bound =>5ms
LSD= 8.6 ms

LinQTHR dQTc

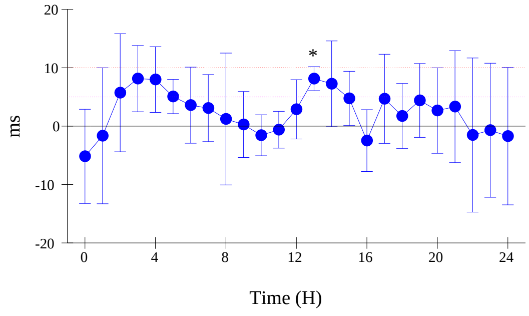

*: P<=0.05: one-sided 95% CI lower bound =>5ms
LSD= 6.3 ms

LinQTHR ddQTc

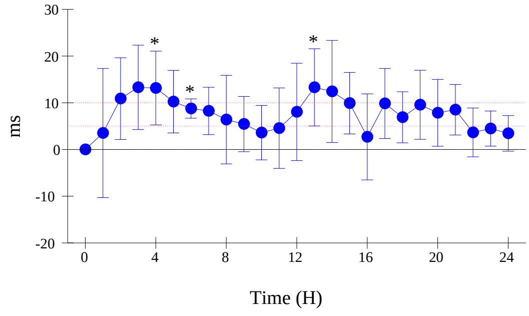

*: P<=0.05: one-sided 95% CI lower bound =>5ms
LSD= 7.2 ms

Figure 72 Risperidone 1 mg/kg iv + atenolol - Effect on β slope (one step QTc model)

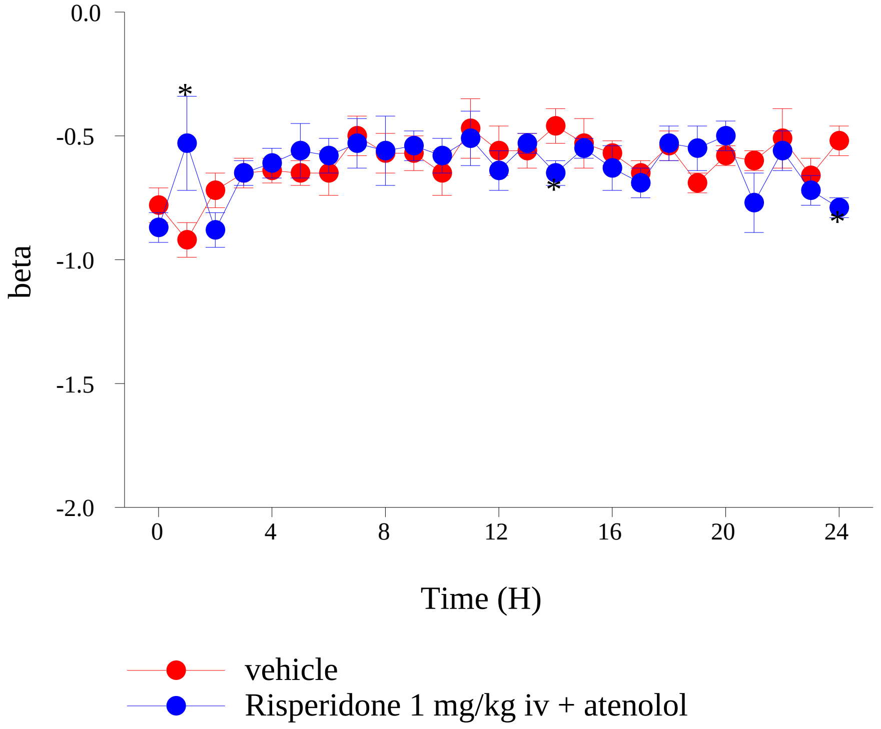

Results expressed in β
Repeated measures analysis of variance (RMANOVA)
Probability for Treatment factor: P=0.861
Probability for Time X Treatment interaction: P=0.021
$\star$: P$\leq$0.05 (LSD)
LSD=0.2 - Least significant difference for α type-1 error=5%
MDD=0.3 - Minimum detectable difference for α type-1 error=5% and β type-2 error=20% (*i.e.*power=80%)
Electronic authentication: created by Pascal Champéroux on 11-FEV-2025 at 14:47:45.325
Study QTOS

Sotalol 3 mg/kg po

Figure 73 Sotalol 3 mg/kg po

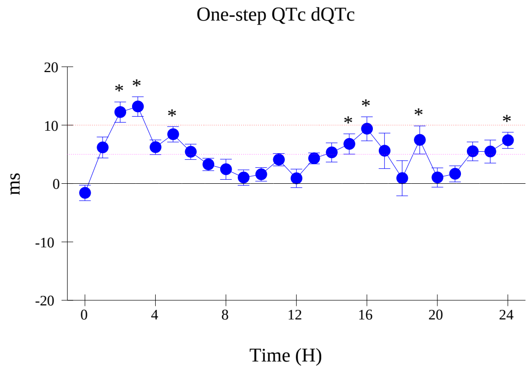

*: P<=0.05: one-sided 95% CI lower bound =>5ms
LSD= 1.6 ms

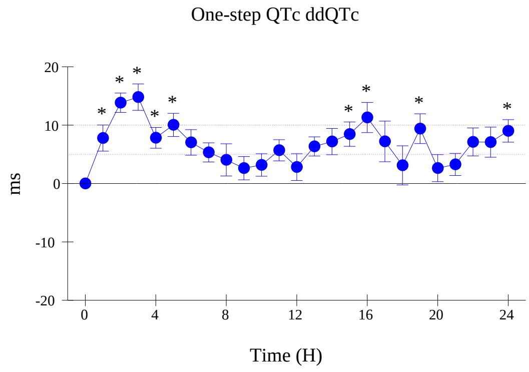

*: P<=0.05: one-sided 95% CI lower bound =>5ms
LSD= 2.2 ms

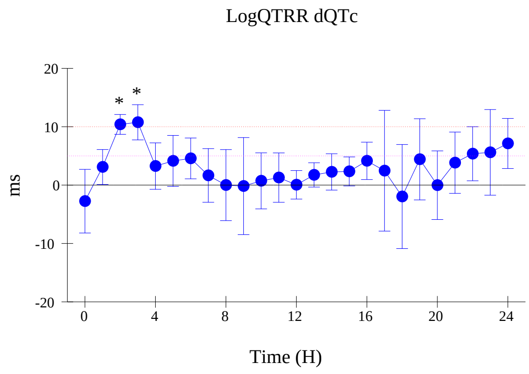

*: P<=0.05: one-sided 95% CI lower bound =>5ms
LSD= 4.4 ms

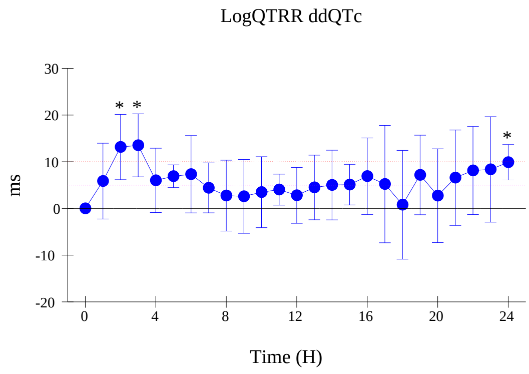

*: P<=0.05: one-sided 95% CI lower bound =>5ms
LSD= 7.6 ms

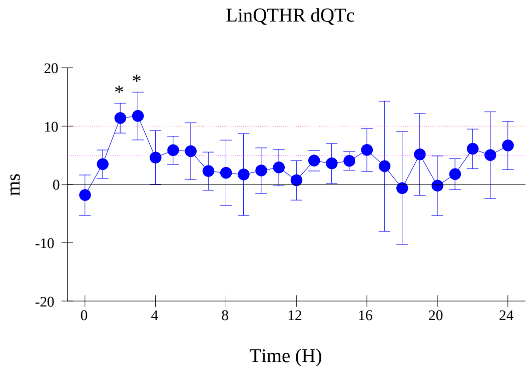

*: P<=0.05: one-sided 95% CI lower bound =>5ms
LSD= 3.7 ms

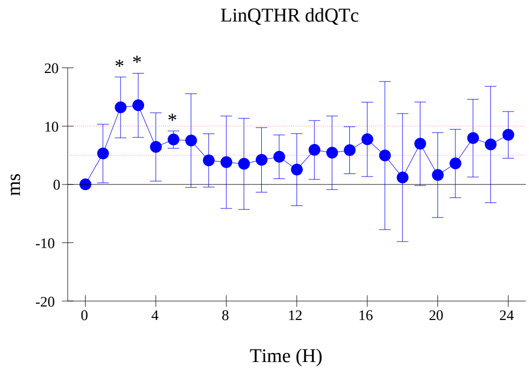

*: P<=0.05: one-sided 95% CI lower bound =>5ms
LSD= 5.9 ms

Figure 74 Sotalol 3 mg/kg po - Effect on β slope (one step QTc model)

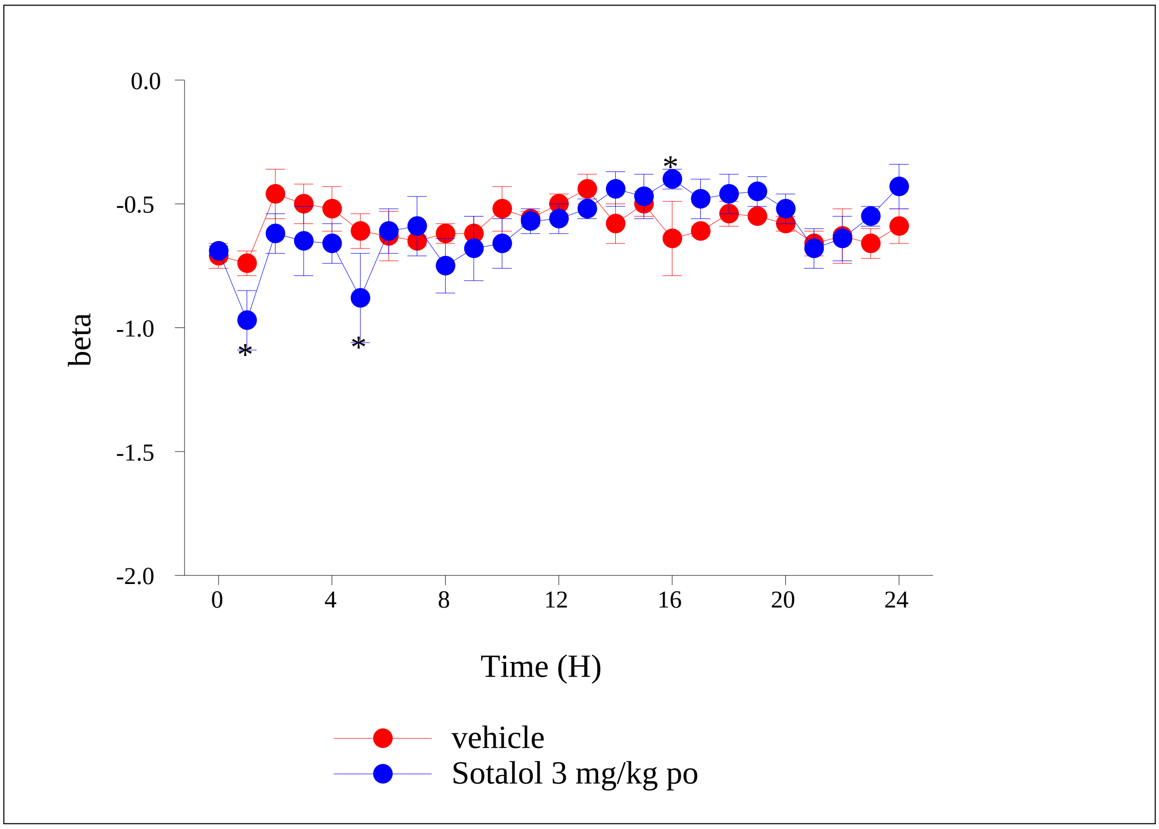

Results expressed in β
Repeated measures analysis of variance (RMANOVA)
Probability for Treatment factor: P=0.802
Probability for Time X Treatment interaction: P=0.096
$\star$: P$\leq$0.05 (LSD)
LSD=0.2 - Least significant difference for α type-1 error=5%
MDD=0.3 - Minimum detectable difference for α type-1 error=5% and β type-2 error=20% (*i.e.*power=80%)
Electronic authentication: created by Pascal Champéroux on 11-FEV-2025 at 14:47:45.533
Study QTOS

Sotalol 10 mg/kg po

Figure 75 Sotalol 10 mg/kg po

One-step QTc dQTc

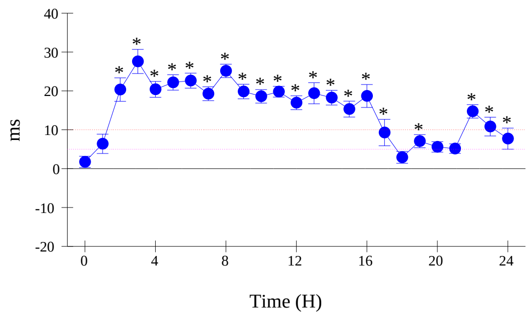

*: P<=0.05: one-sided 95% CI lower bound =>5ms
LSD= 1.9 ms

One-step QTc ddQTc

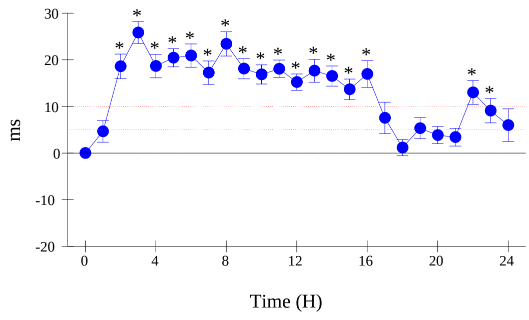

*: P<=0.05: one-sided 95% CI lower bound =>5ms
LSD= 2.3 ms

LogQTRR dQTc

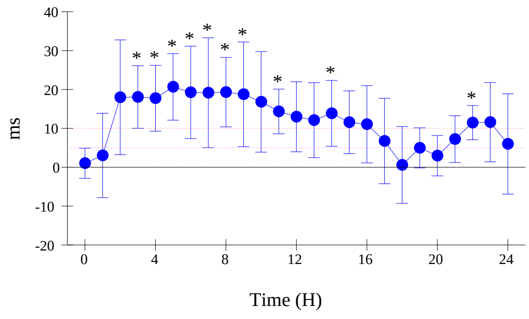

*: P<=0.05: one-sided 95% CI lower bound =>5ms
LSD= 9 ms

LogQTRR ddQTc

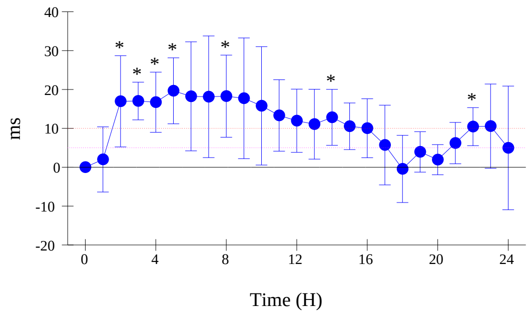

*: P<=0.05: one-sided 95% CI lower bound =>5ms
LSD= 8.5 ms

LinQTHR dQTc

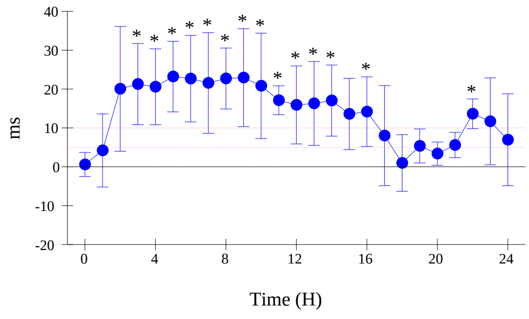

*: P<=0.05: one-sided 95% CI lower bound =>5ms
LSD= 9.4 ms

LinQTHR ddQTc

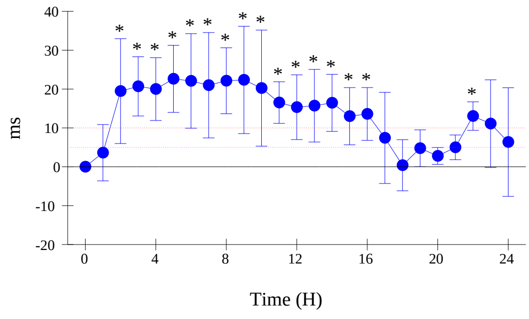

*: P<=0.05: one-sided 95% CI lower bound =>5ms
LSD= 8.1 ms

Figure 76 Sotalol 10 mg/kg po - Effect on β slope (one step QTc model)

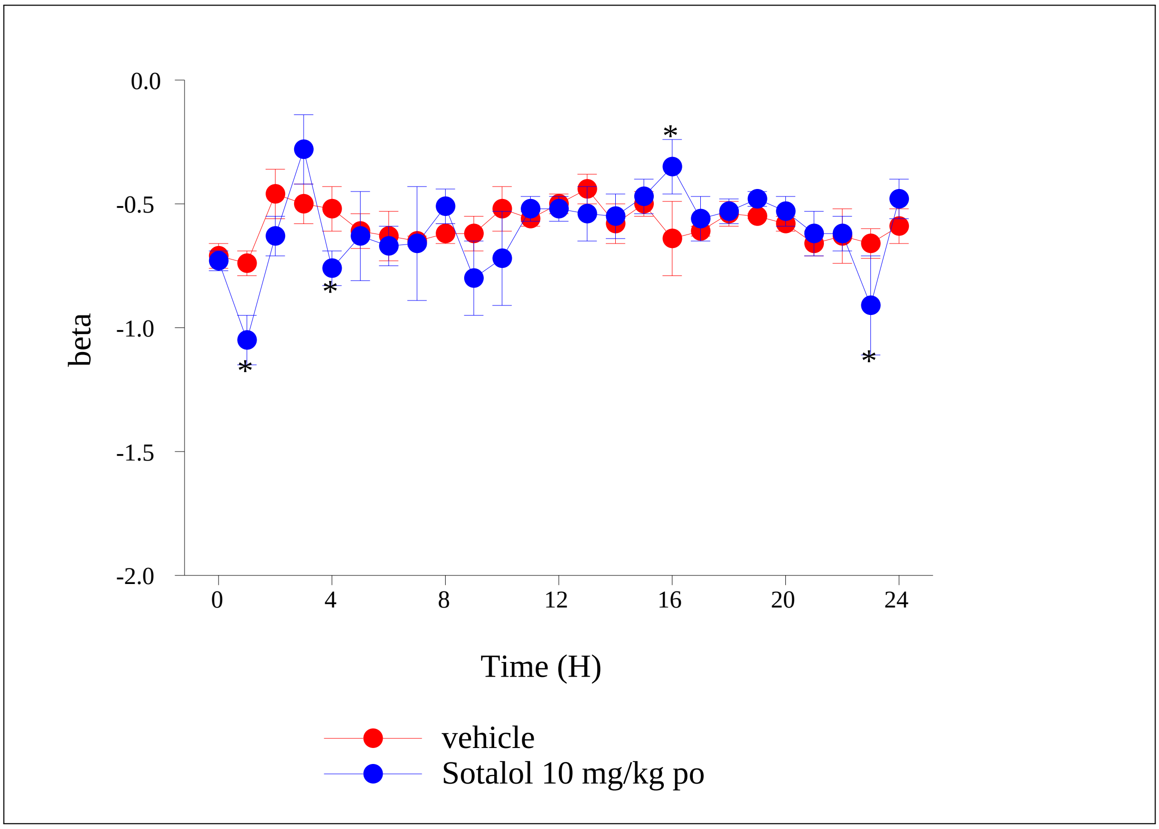

Results expressed in β
Repeated measures analysis of variance (RMANOVA)
Probability for Treatment factor: P=0.737
Probability for Time X Treatment interaction: P=0.125
$\star$: P$\leq$0.05 (LSD)
LSD=0.2 - Least significant difference for α type-1 error=5%
MDD=0.3 - Minimum detectable difference for α type-1 error=5% and β type-2 error=20% (*i.e.*power=80%)
Electronic authentication: created by Pascal Champéroux on 11-FEV-2025 at 14:47:45.692
Study QTOS

Sotalol 30 mg/kg po

Figure 77 Sotalol 30 mg/kg po

One-step QTc dQTc

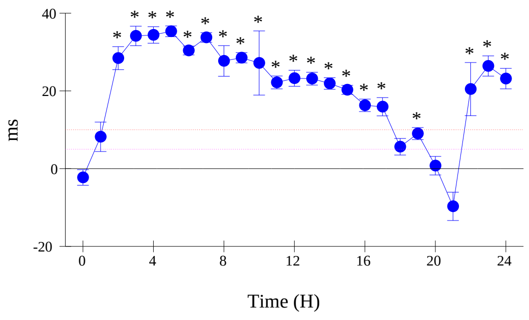

*: P<=0.05: one-sided 95% CI lower bound =>5ms
LSD= 2.1 ms

One-step QTc ddQTc

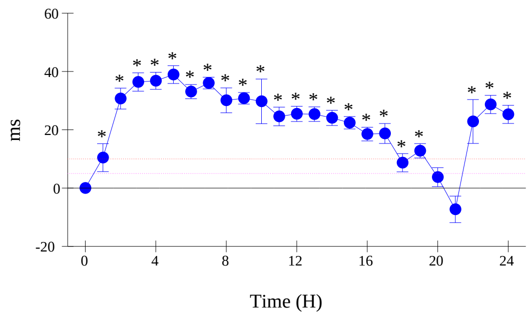

*: P<=0.05: one-sided 95% CI lower bound =>5ms
LSD= 3.1 ms

LogQTRR dQTc

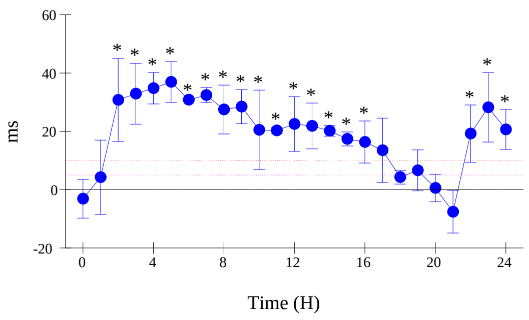

*: P<=0.05: one-sided 95% CI lower bound =>5ms
LSD= 7 ms

LogQTRR ddQTc

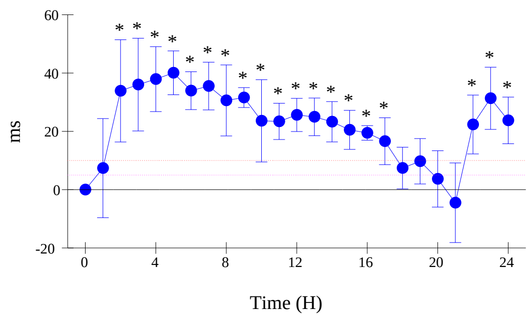

*: P<=0.05: one-sided 95% CI lower bound =>5ms
LSD= 8 ms

LinQTHR dQTc

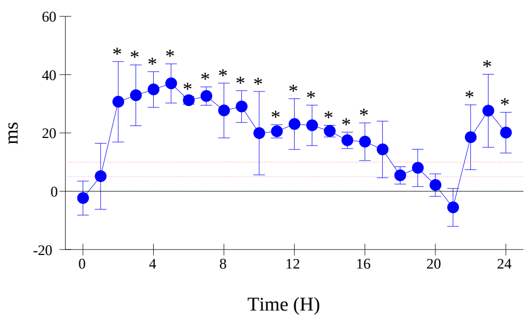

*: P<=0.05: one-sided 95% CI lower bound =>5ms
LSD= 6.5 ms

LinQTHR ddQTc

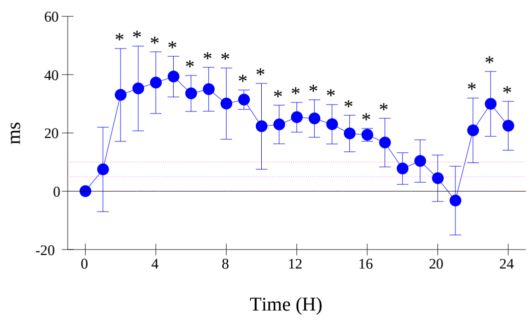

*: P<=0.05: one-sided 95% CI lower bound =>5ms
LSD= 7.5 ms

Figure 78 Sotalol 30 mg/kg po - Effect on β slope (one step QTc model)

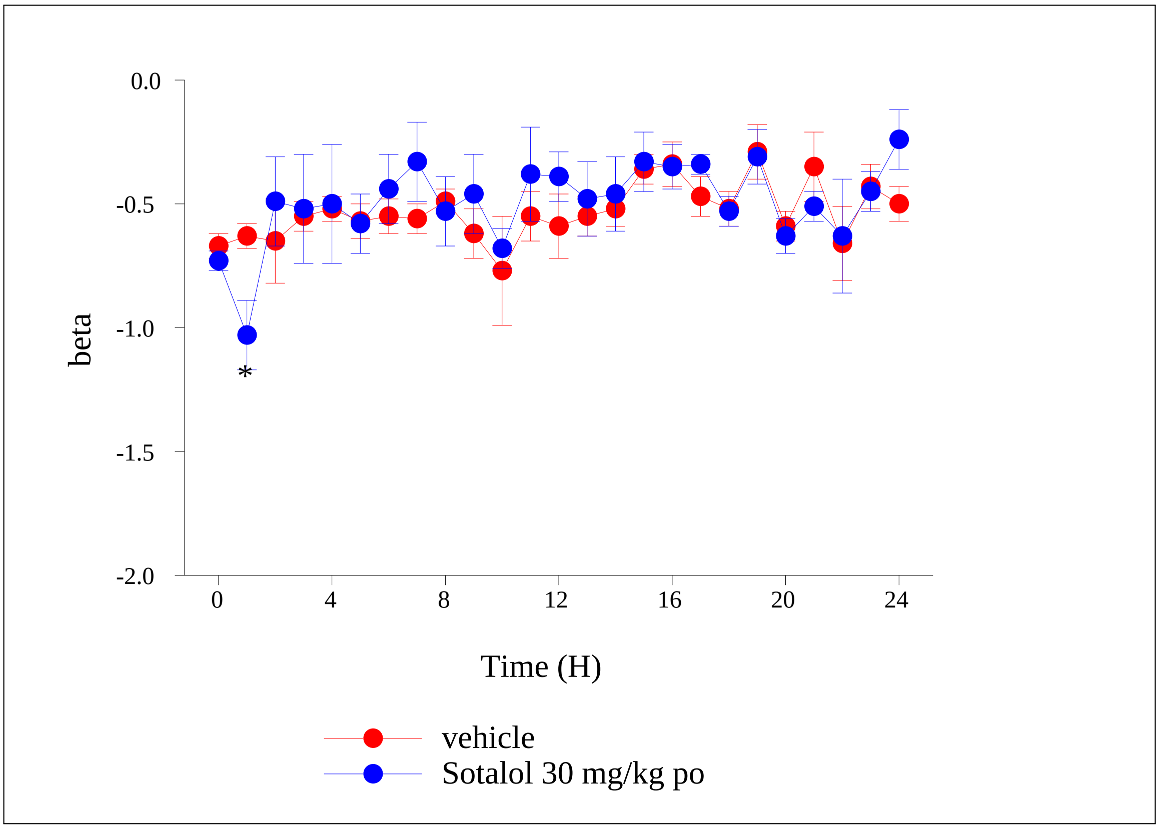

Results expressed in β
Repeated measures analysis of variance (RMANOVA)
Probability for Treatment factor: P=0.696
Probability for Time X Treatment interaction: P=0.628
$\star$: P$\leq$0.05 (LSD)
LSD=0.3 - Least significant difference for α type-1 error=5%
MDD=0.4 - Minimum detectable difference for α type-1 error=5% and β type-2 error=20% (*i.e.*power=80%)
Electronic authentication: created by Pascal Champéroux on 11-FEV-2025 at 14:47:45.804
Study QTOS

Sertindole 1 mg/kg iv

Figure 79 Sertindole 1 mg/kg iv

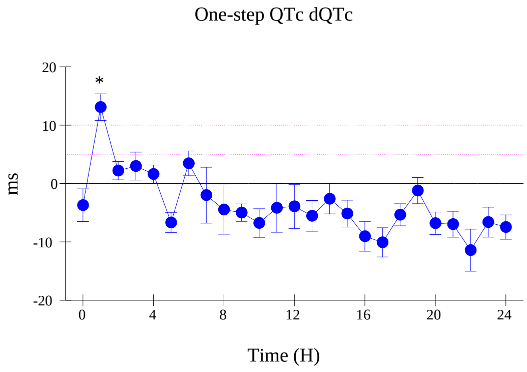

*: P<=0.05: one-sided 95% CI lower bound =>5ms
LSD= 2.4 ms

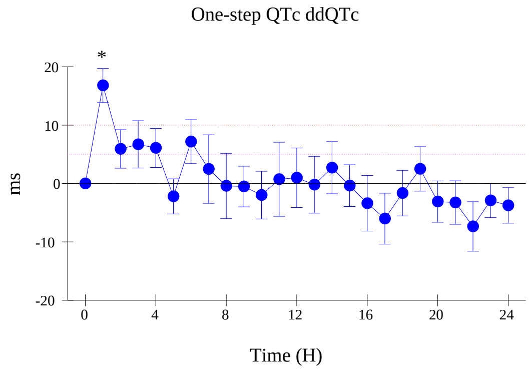

*: P<=0.05: one-sided 95% CI lower bound =>5ms
LSD= 3.8 ms

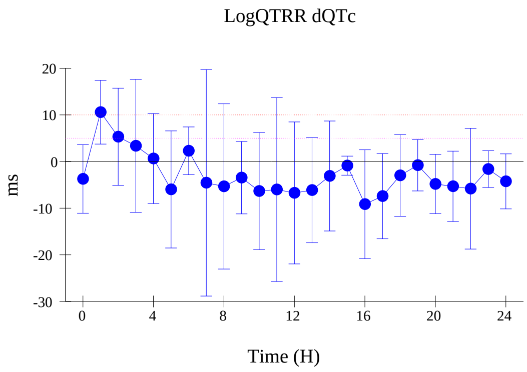

*: P<=0.05: one-sided 95% CI lower bound =>5ms
LSD= 9.7 ms

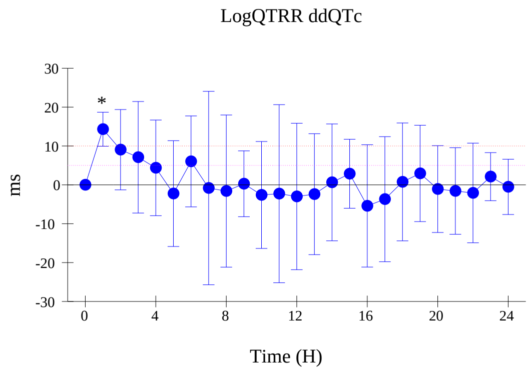

*: P<=0.05: one-sided 95% CI lower bound =>5ms
LSD= 12.8 ms

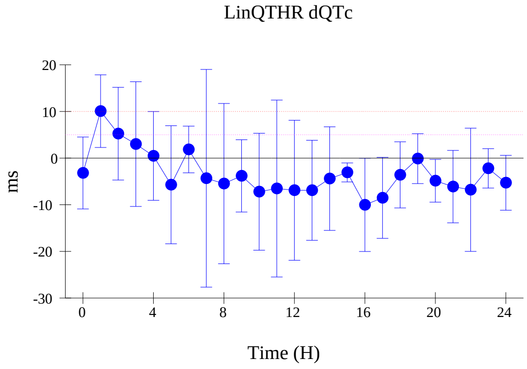

*: P<=0.05: one-sided 95% CI lower bound =>5ms
LSD= 9.5 ms

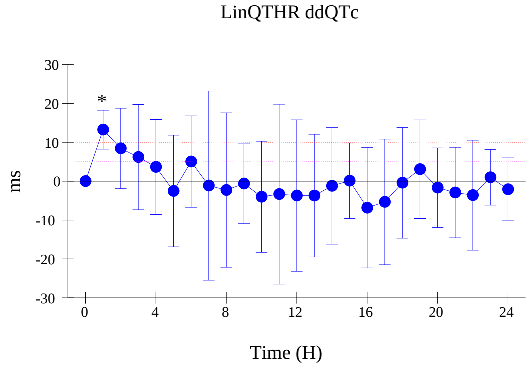

*: P<=0.05: one-sided 95% CI lower bound =>5ms
LSD= 13.5 ms

Figure 80 Sertindole 1 mg/kg iv - Effect on β slope (one step QTc model)

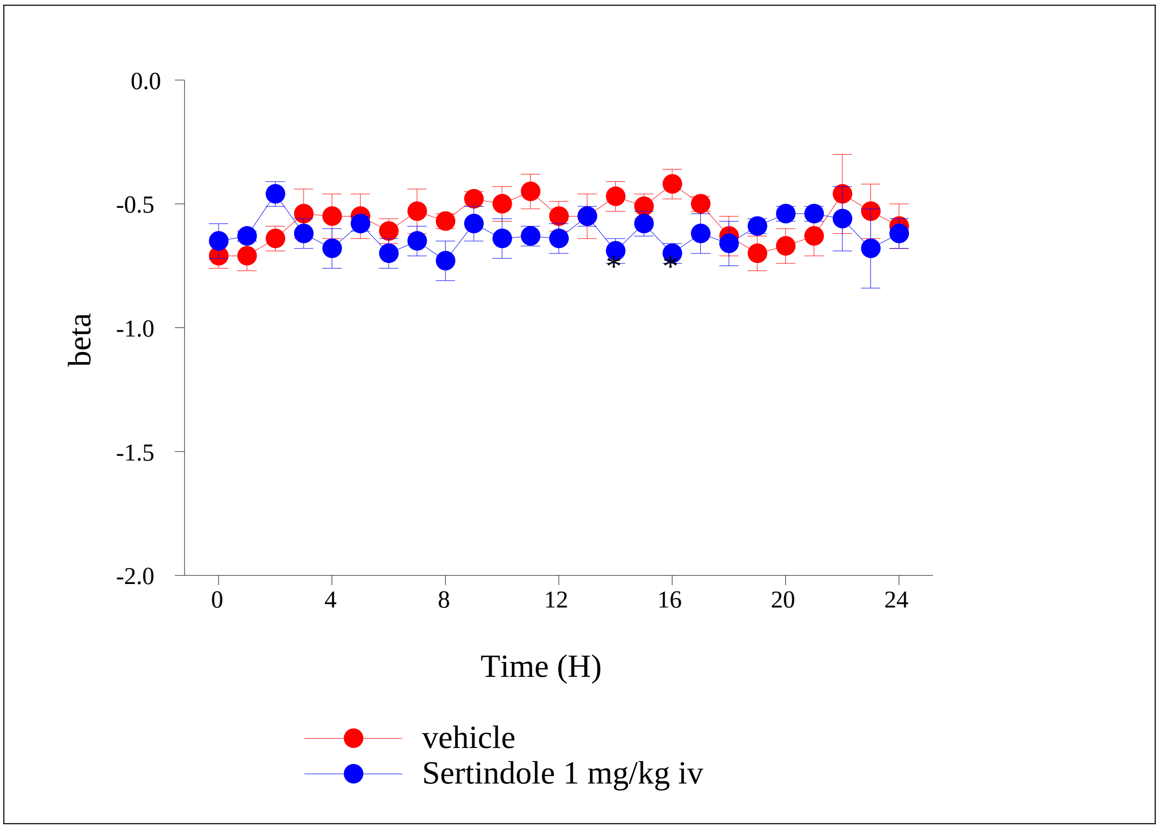

Results expressed in β
Repeated measures analysis of variance (RMANOVA)
Probability for Treatment factor: P=0.282
Probability for Time X Treatment interaction: P=0.06
$\star$: P$\leq$0.05 (LSD)
LSD=0.2 - Least significant difference for α type-1 error=5%
MDD=0.3 - Minimum detectable difference for α type-1 error=5% and β type-2 error=20% (*i.e.*power=80%)
Electronic authentication: created by Pascal Champéroux on 11-FEV-2025 at 14:47:45.980
Study QTOS

Sertindole 1 mg/kg iv + atenolol

Figure 81 Sertindole 1 mg/kg iv + atenolol

One-step QTc dQTc

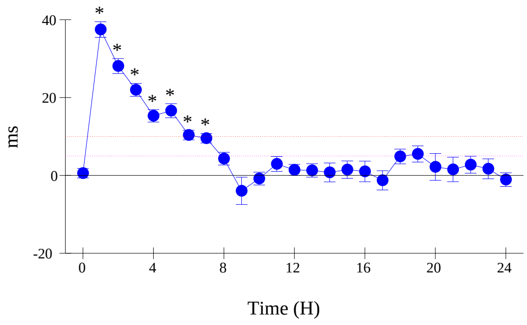

*: P<=0.05: one-sided 95% CI lower bound =>5ms
LSD= 1.9 ms

One-step QTc ddQTc

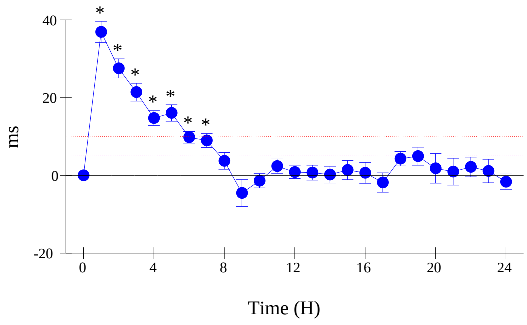

*: P<=0.05: one-sided 95% CI lower bound =>5ms
LSD= 2.2 ms

LogQTRR dQTc

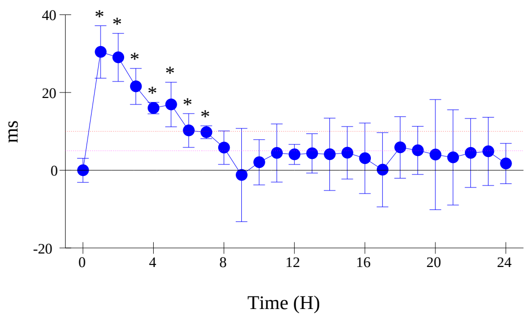

*: P<=0.05: one-sided 95% CI lower bound =>5ms
LSD= 6.2 ms

LogQTRR ddQTc

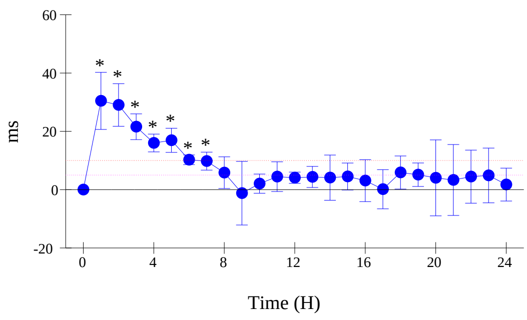

*: P<=0.05: one-sided 95% CI lower bound =>5ms
LSD= 5.4 ms

LinQTHR dQTc

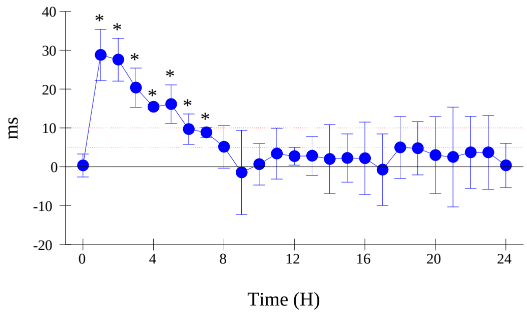

*: P<=0.05: one-sided 95% CI lower bound =>5ms
LSD= 6.2 ms

LinQTHR ddQTc

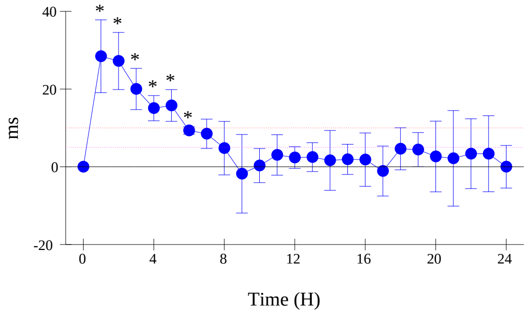

*: P<=0.05: one-sided 95% CI lower bound =>5ms
LSD= 5.4 ms

Figure 82 Sertindole 1 mg/kg iv + atenolol - Effect on β slope (one step QTc model)

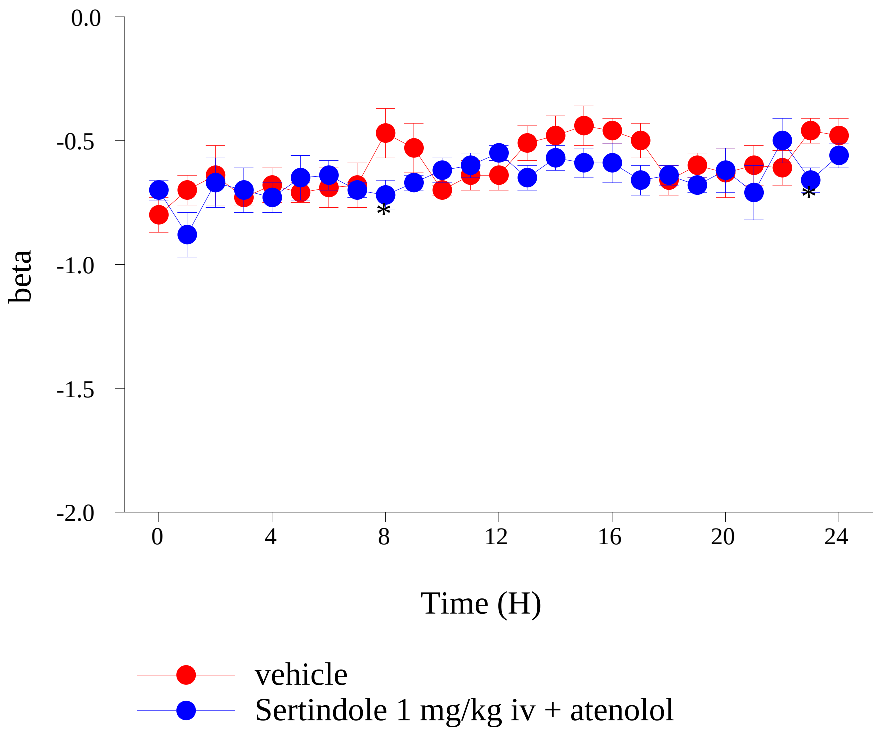

Results expressed in β
Repeated measures analysis of variance (RMANOVA)
Probability for Treatment factor: P=0.159
Probability for Time X Treatment interaction: P=0.225
$\star$: P$\leq$0.05 (LSD)
LSD=0.2 - Least significant difference for α type-1 error=5%
MDD=0.3 - Minimum detectable difference for α type-1 error=5% and β type-2 error=20% (*i.e.*power=80%)
Electronic authentication: created by Pascal Champéroux on 11-FEV-2025 at 14:47:46.152
Study QTOS

Terfenadine 30 mg/kg po

Figure 83 Terfenadine 30 mg/kg po

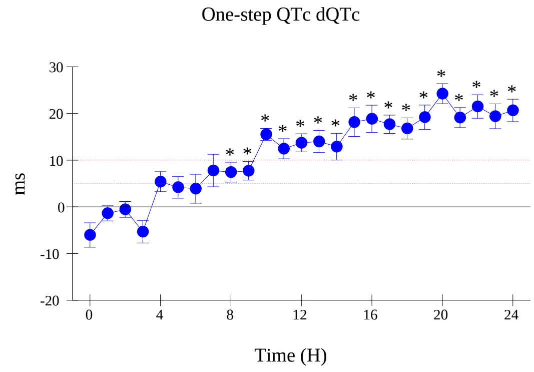

*: P<=0.05: one-sided 95% CI lower bound =>5ms
LSD= 2.3 ms

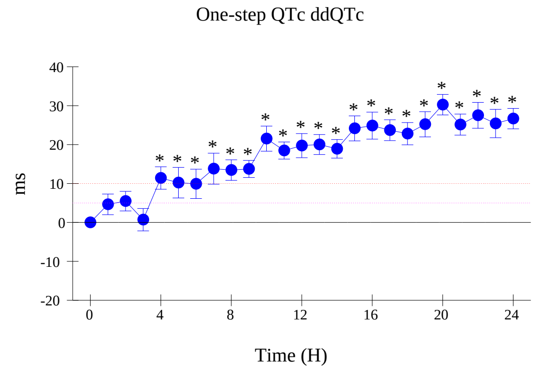

*: P<=0.05: one-sided 95% CI lower bound =>5ms
LSD= 2.9 ms

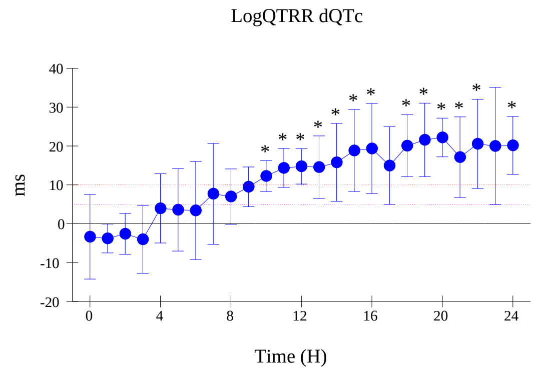

*: P<=0.05: one-sided 95% CI lower bound =>5ms
LSD= 8.9 ms

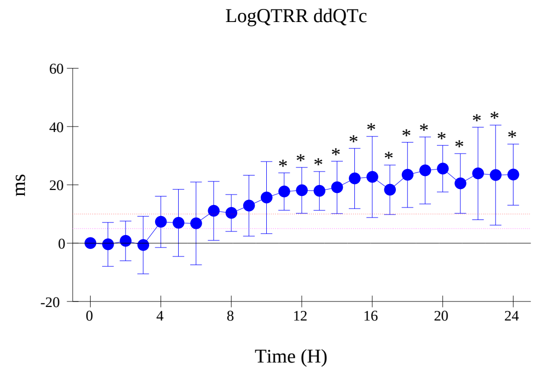

*: P<=0.05: one-sided 95% CI lower bound =>5ms
LSD= 10.1 ms

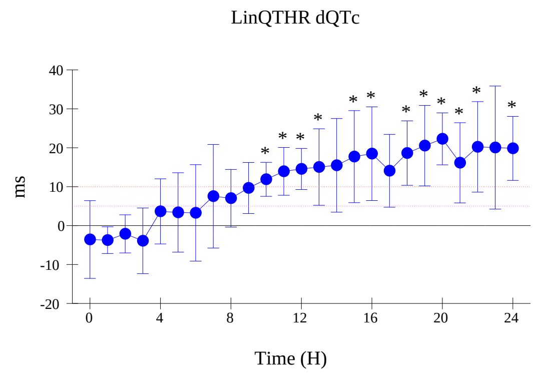

*: P<=0.05: one-sided 95% CI lower bound =>5ms
LSD= 9.4 ms

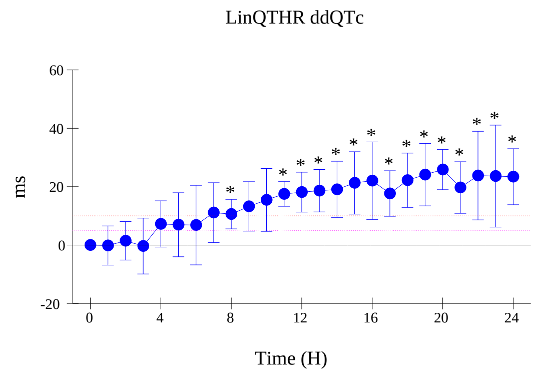

*: P<=0.05: one-sided 95% CI lower bound =>5ms
LSD= 9.3 ms

Figure 84 Terfenadine 30 mg/kg po - Effect on β slope (one step QTc model)

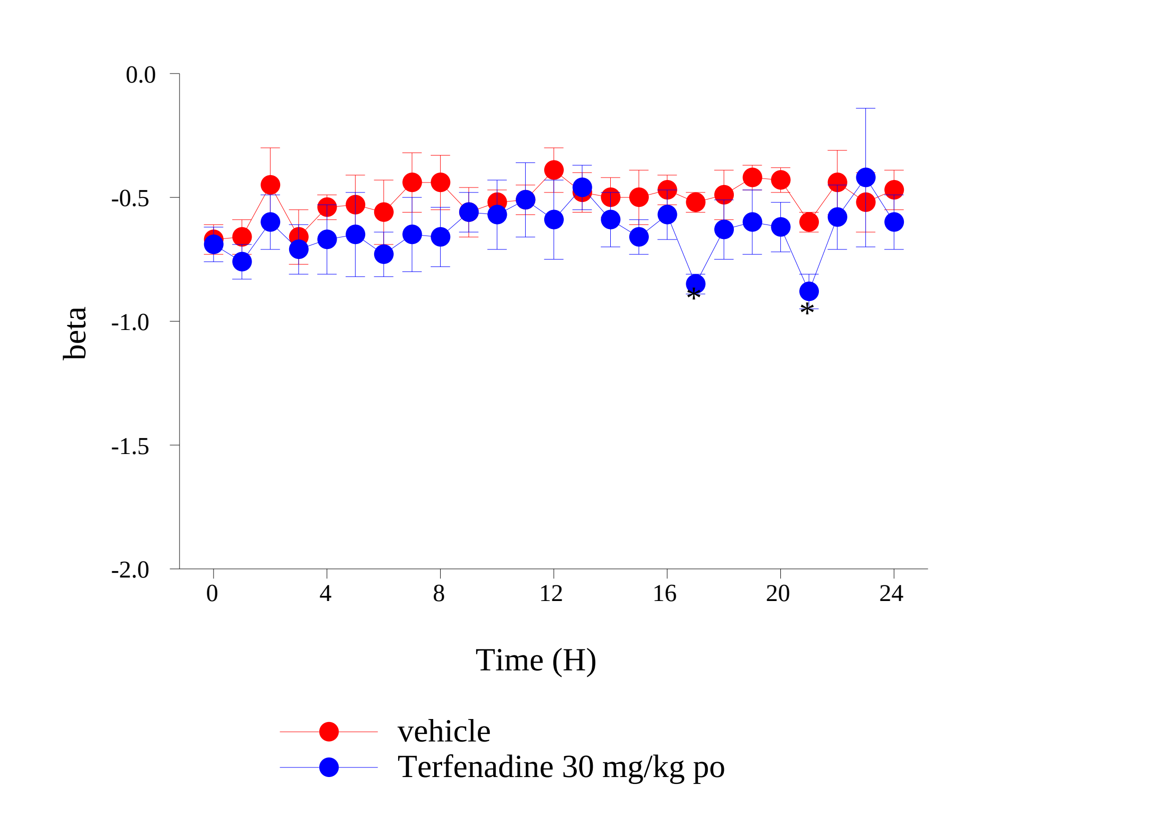

Results expressed in β
Repeated measures analysis of variance (RMANOVA)
Probability for Treatment factor: P=0.223
Probability for Time X Treatment interaction: P=0.947
$\star$: P$\leq$0.05 (LSD)
LSD=0.3 - Least significant difference for α type-1 error=5%
MDD=0.4 - Minimum detectable difference for α type-1 error=5% and β type-2 error=20% (*i.e.*power=80%)
Electronic authentication: created by Pascal Champéroux on 11-FEV-2025 at 14:47:46.331
Study QTOS

Terfenadine 100 mg/kg po

Figure 85 Terfenadine 100 mg/kg po

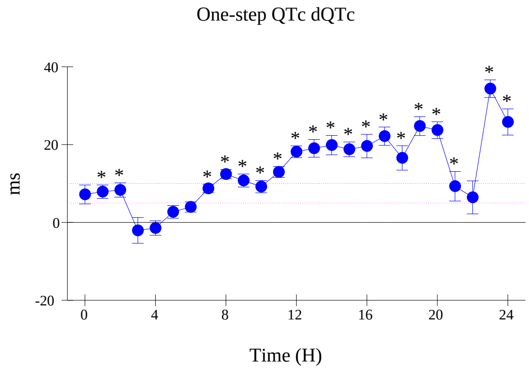

*: P<=0.05: one-sided 95% CI lower bound =>5ms
LSD= 2.2 ms

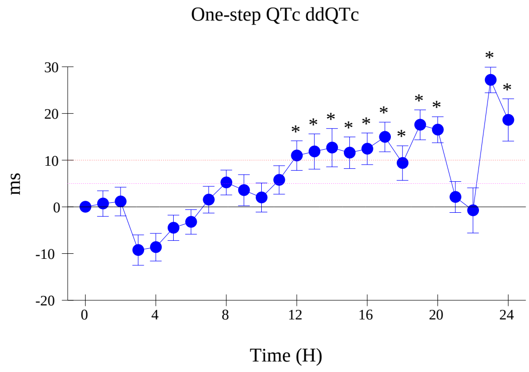

*: P<=0.05: one-sided 95% CI lower bound =>5ms
LSD= 3.2 ms

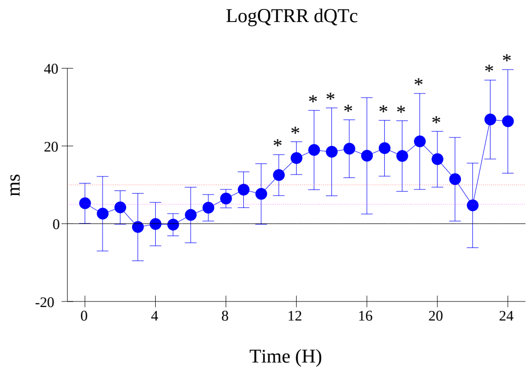

*: P<=0.05: one-sided 95% CI lower bound =>5ms
LSD= 7.5 ms

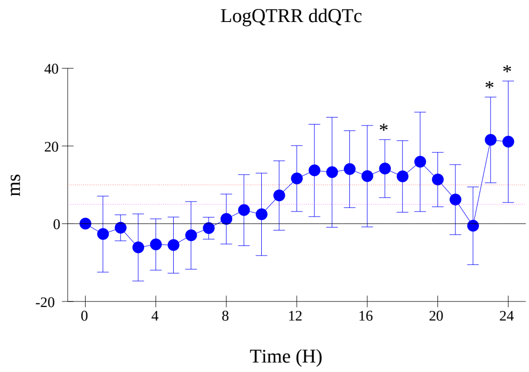

*: P<=0.05: one-sided 95% CI lower bound =>5ms
LSD= 9 ms

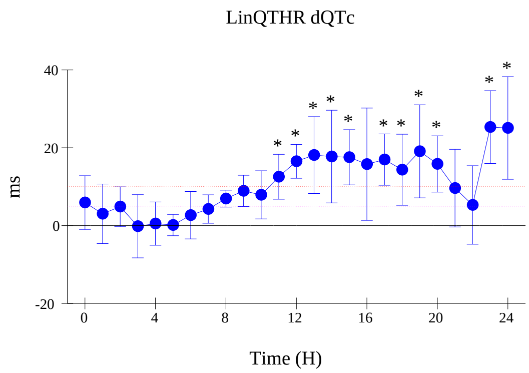

*: P<=0.05: one-sided 95% CI lower bound =>5ms
LSD= 7.1 ms

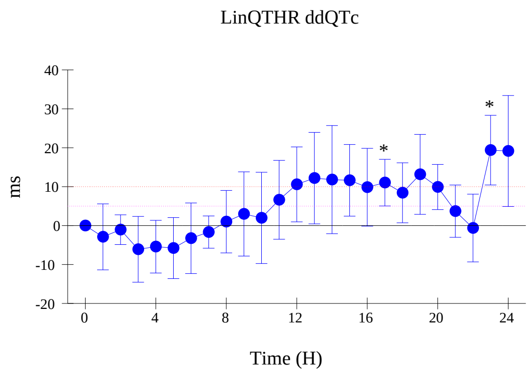

*: P<=0.05: one-sided 95% CI lower bound =>5ms
LSD= 8.7 ms

Figure 86 Terfenadine 100 mg/kg po - Effect on β slope (one step QTc model)

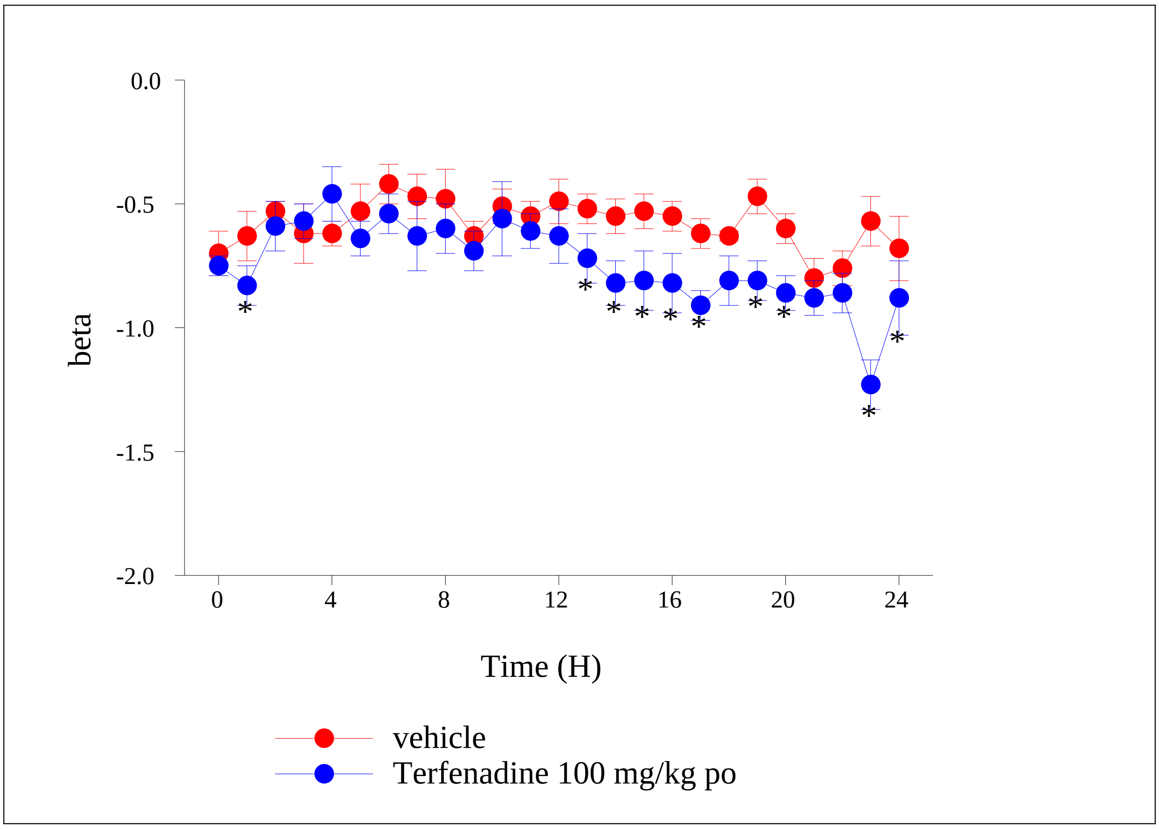

Results expressed in β
Repeated measures analysis of variance (RMANOVA)
Probability for Treatment factor: P=0.083
Probability for Time X Treatment interaction: P=0
$\star$: P$\leq$0.05 (LSD)
LSD=0.2 - Least significant difference for α type-1 error=5%
MDD=0.3 - Minimum detectable difference for α type-1 error=5% and β type-2 error=20% (*i.e.*power=80%)
Electronic authentication: created by Pascal Champéroux on 11-FEV-2025 at 14:47:46.507
Study QTOS

Thioridazine 1.5 mg/kg po

Figure 87 Thioridazine 1.5 mg/kg po

One-step QTc dQTc

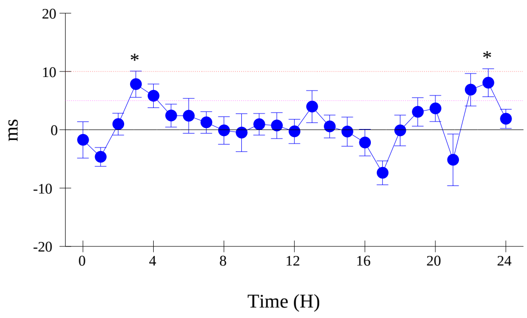

*: P<=0.05: one-sided 95% CI lower bound =>5ms
LSD= 2.3 ms

One-step QTc ddQTc

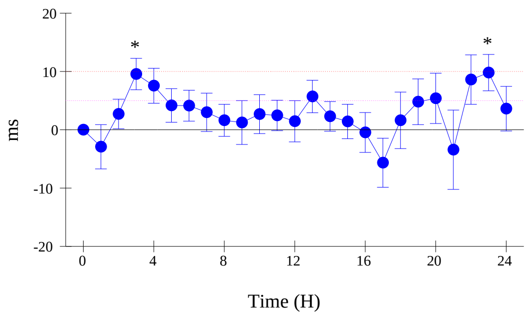

*: P<=0.05: one-sided 95% CI lower bound =>5ms
LSD= 3.3 ms

LogQTRR dQTc

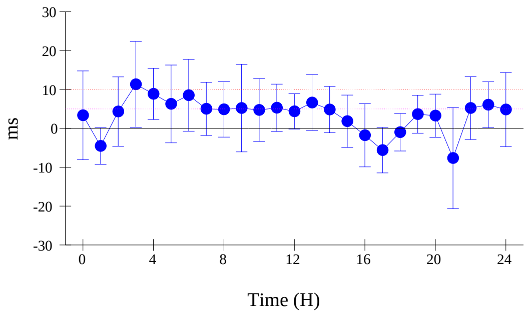

*: P<=0.05: one-sided 95% CI lower bound =>5ms
LSD= 7.1 ms

LogQTRR ddQTc

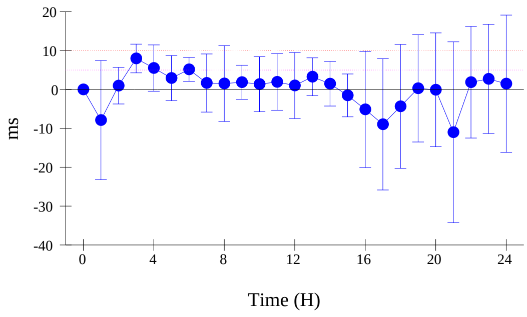

*: P<=0.05: one-sided 95% CI lower bound =>5ms
LSD= 7.5 ms

LinQTHR dQTc

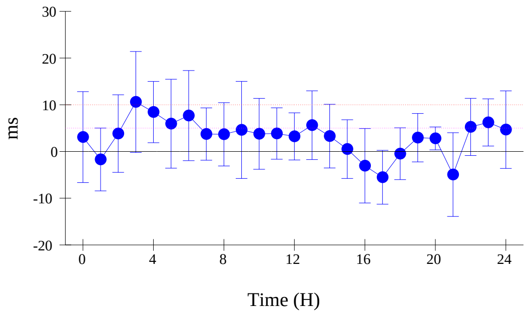

*: P<=0.05: one-sided 95% CI lower bound =>5ms
LSD= 6.8 ms

LinQTHR ddQTc

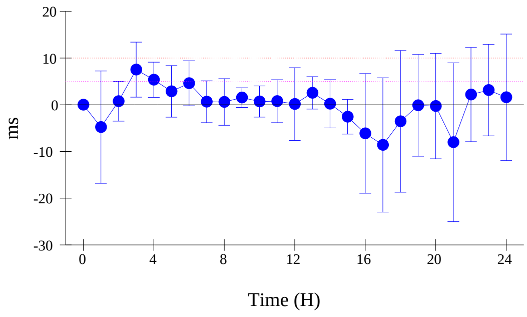

*: P<=0.05: one-sided 95% CI lower bound =>5ms
LSD= 5.5 ms

Figure 88 Thioridazine 1.5 mg/kg po - Effect on β slope (one step QTc model)

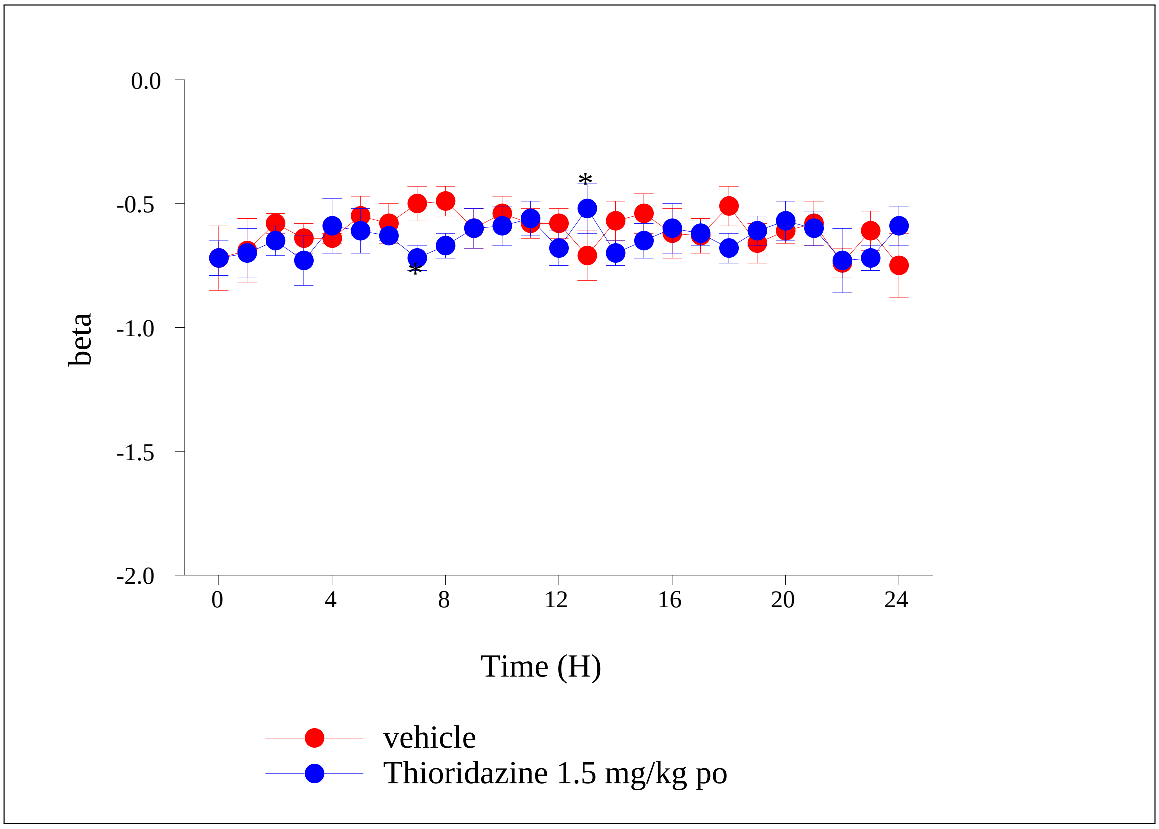

Results expressed in β
Repeated measures analysis of variance (RMANOVA)
Probability for Treatment factor: P=0.626
Probability for Time X Treatment interaction: P=0.375
$\star$: P$\leq$0.05 (LSD)
LSD=0.2 - Least significant difference for α type-1 error=5%
MDD=0.3 - Minimum detectable difference for α type-1 error=5% and β type-2 error=20% (*i.e.*power=80%)
Electronic authentication: created by Pascal Champéroux on 11-FEV-2025 at 14:47:46.683
Study QTOS

Thioridazine 1.5 mg/kg po + atenolol

Figure 89 Thioridazine 1.5 mg/kg po + atenolol

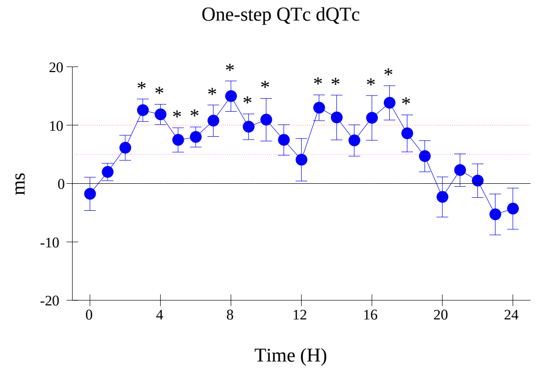

*: P<=0.05: one-sided 95% CI lower bound =>5ms
LSD= 2.7 ms

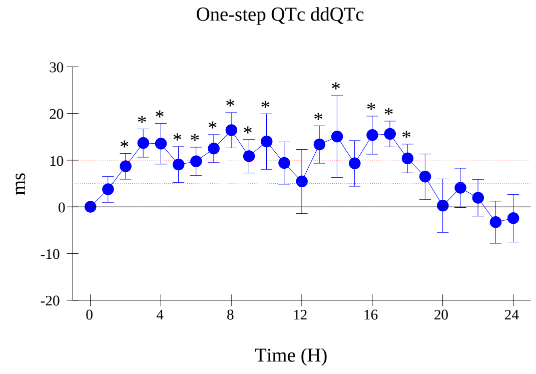

*: P<=0.05: one-sided 95% CI lower bound =>5ms
LSD= 4 ms

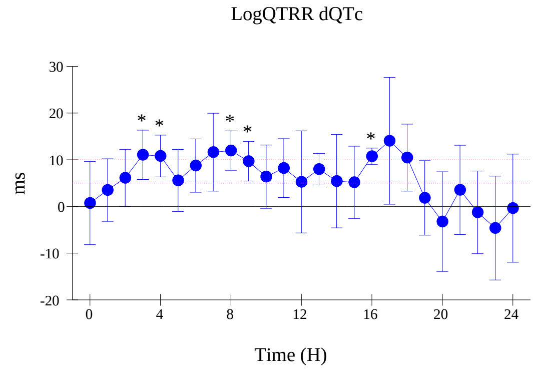

*: P<=0.05: one-sided 95% CI lower bound =>5ms
LSD= 7.2 ms

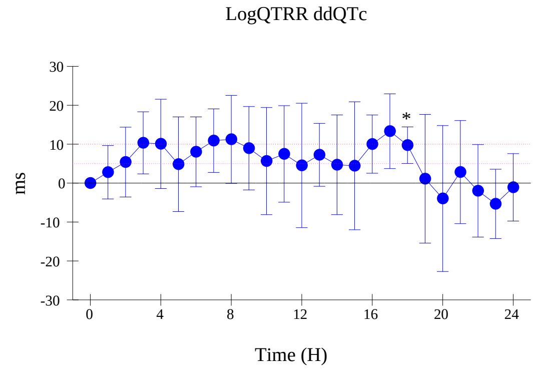

*: P<=0.05: one-sided 95% CI lower bound =>5ms
LSD= 10.7 ms

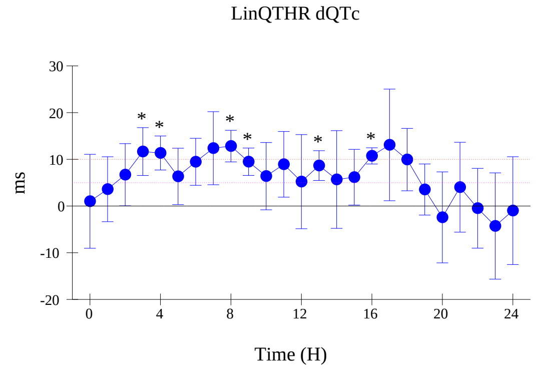

*: P<=0.05: one-sided 95% CI lower bound =>5ms
LSD= 7 ms

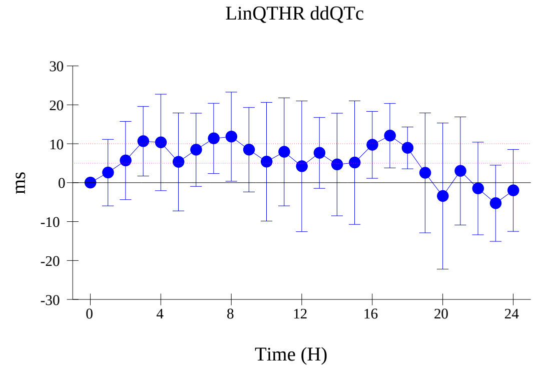

*: P<=0.05: one-sided 95% CI lower bound =>5ms
LSD= 10.8 ms

Figure 90 Thioridazine 1.5 mg/kg po + atenolol - Effect on β slope (one step QTc model)

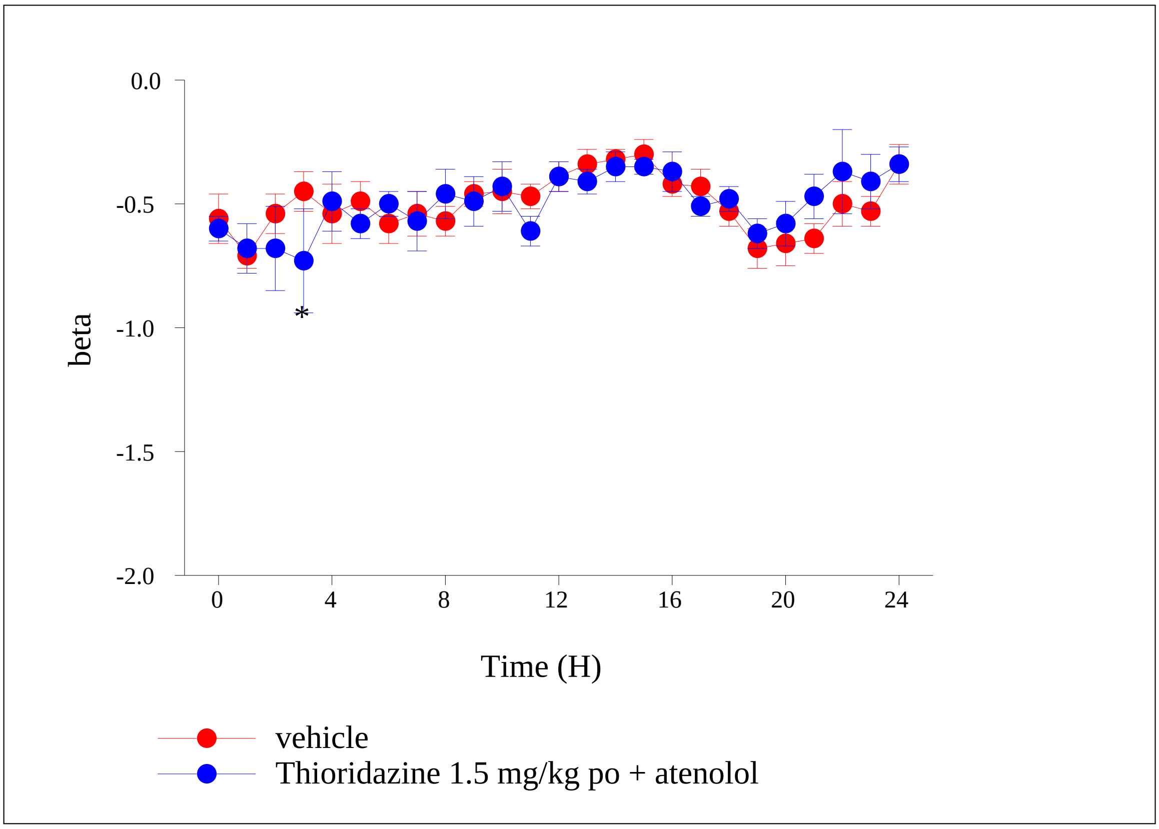

Results expressed in β
Repeated measures analysis of variance (RMANOVA)
Probability for Treatment factor: P=0.99
Probability for Time X Treatment interaction: P=0.591
$\star$: P$\leq$0.05 (LSD)
LSD=0.2 - Least significant difference for α type-1 error=5%
MDD=0.3 - Minimum detectable difference for α type-1 error=5% and β type-2 error=20% (*i.e.*power=80%)
Electronic authentication: created by Pascal Champéroux on 11-FEV-2025 at 14:47:46.859
Study QTOS

Thioridazine 5 mg/kg po

Figure 91 Thioridazine 5 mg/kg po

One-step QTc dQTc

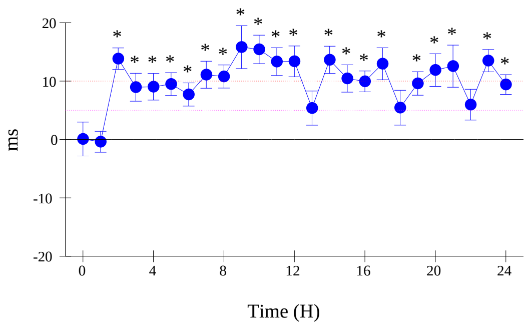

*: P<=0.05: one-sided 95% CI lower bound =>5ms
LSD= 2.3 ms

One-step QTc ddQTc

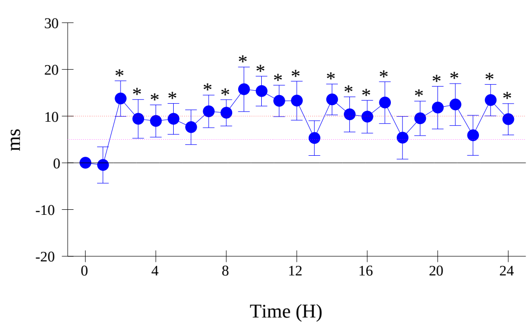

*: P<=0.05: one-sided 95% CI lower bound =>5ms
LSD= 3.7 ms

LogQTRR dQTc

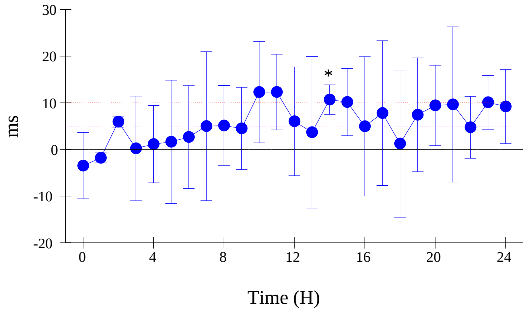

*: P<=0.05: one-sided 95% CI lower bound =>5ms
LSD= 8.8 ms

LogQTRR ddQTc

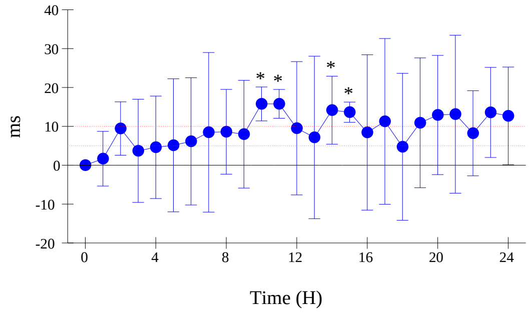

*: P<=0.05: one-sided 95% CI lower bound =>5ms
LSD= 13.3 ms

LinQTHR dQTc

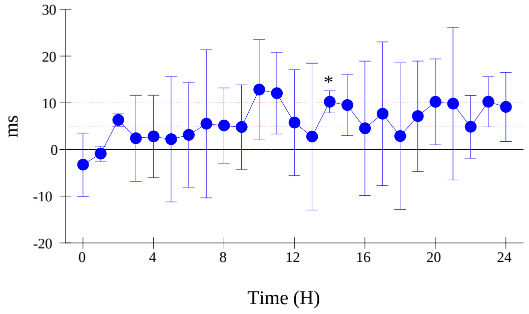

*: P<=0.05: one-sided 95% CI lower bound =>5ms
LSD= 9.2 ms

LinQTHR ddQTc

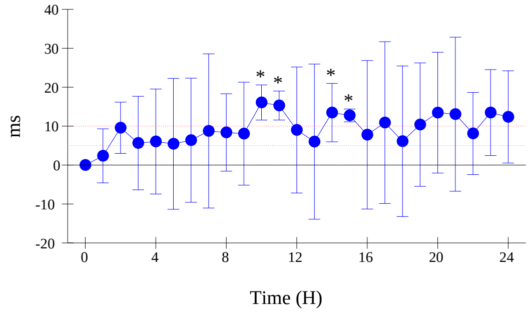

*: P<=0.05: one-sided 95% CI lower bound =>5ms
LSD= 13.2 ms

Figure 92 Thioridazine 5 mg/kg po - Effect on β slope (one step QTc model)

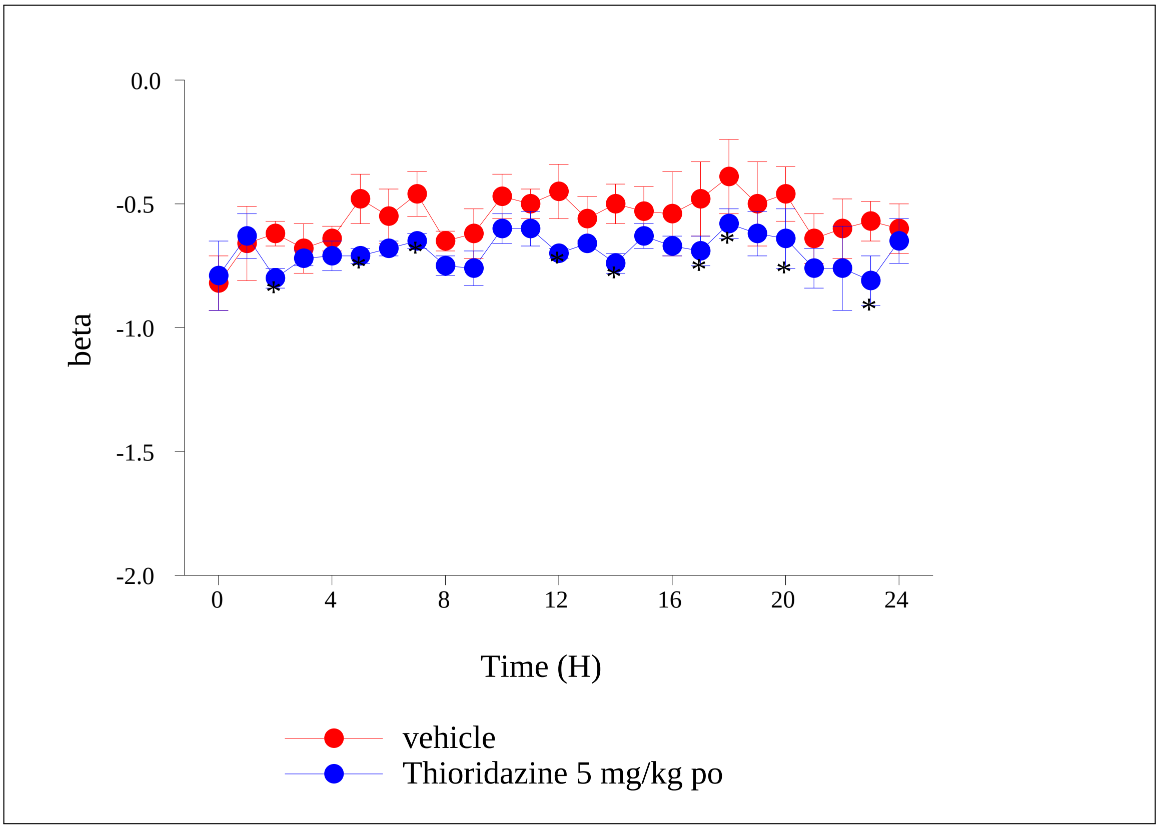

Results expressed in β
Repeated measures analysis of variance (RMANOVA)
Probability for Treatment factor: P=0.202
Probability for Time X Treatment interaction: P=0.867
$\star$: P$\leq$0.05 (LSD)
LSD=0.2 - Least significant difference for α type-1 error=5%
MDD=0.3 - Minimum detectable difference for α type-1 error=5% and β type-2 error=20% (*i.e.*power=80%)
Electronic authentication: created by Pascal Champéroux on 11-FEV-2025 at 14:47:47.179
Study QTOS

Thioridazine 20 mg/kg po

Figure 93 Thioridazine 20 mg/kg po

One-step QTc dQTc

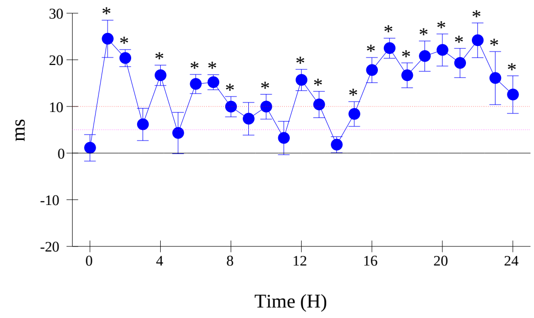

*: P<=0.05: one-sided 95% CI lower bound =>5ms
LSD= 2.8 ms

One-step QTc ddQTc

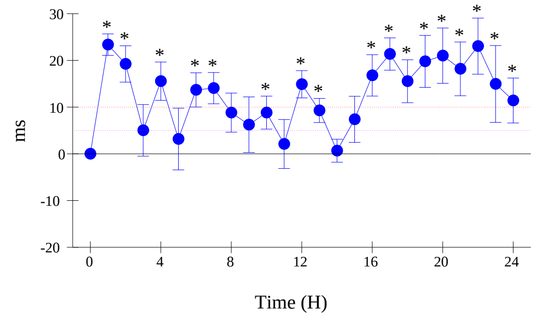

*: P<=0.05: one-sided 95% CI lower bound =>5ms
LSD= 4.4 ms

LogQTRR dQTc

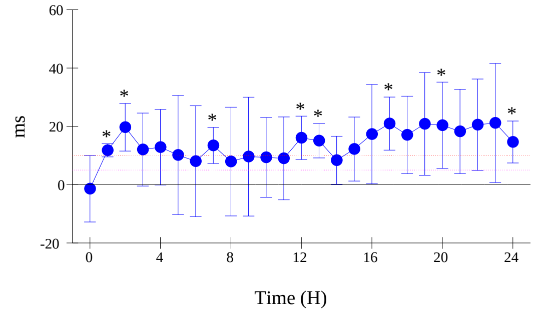

*: P<=0.05: one-sided 95% CI lower bound =>5ms
LSD= 13.3 ms

LogQTRR ddQTc

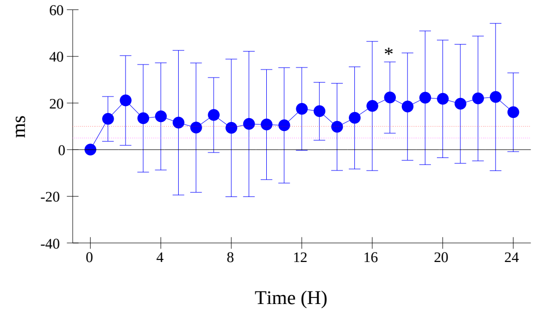

*: P<=0.05: one-sided 95% CI lower bound =>5ms
LSD= 23.1 ms

LinQTHR dQTc

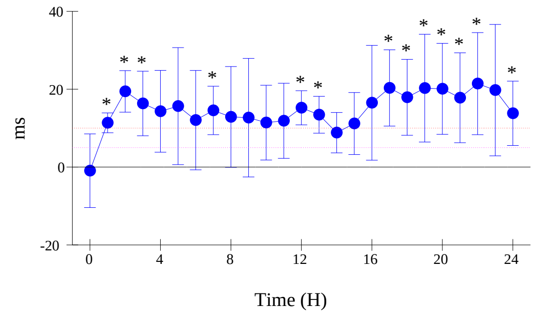

*: P<=0.05: one-sided 95% CI lower bound =>5ms
LSD= 9.8 ms

LinQTHR ddQTc

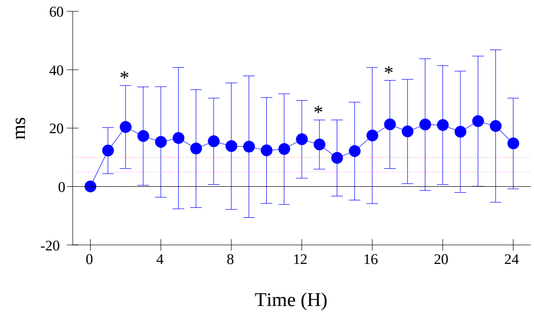

*: P<=0.05: one-sided 95% CI lower bound =>5ms
LSD= 18.1 ms

Figure 94 Thioridazine 20 mg/kg po - Effect on β slope (one step QTc model)

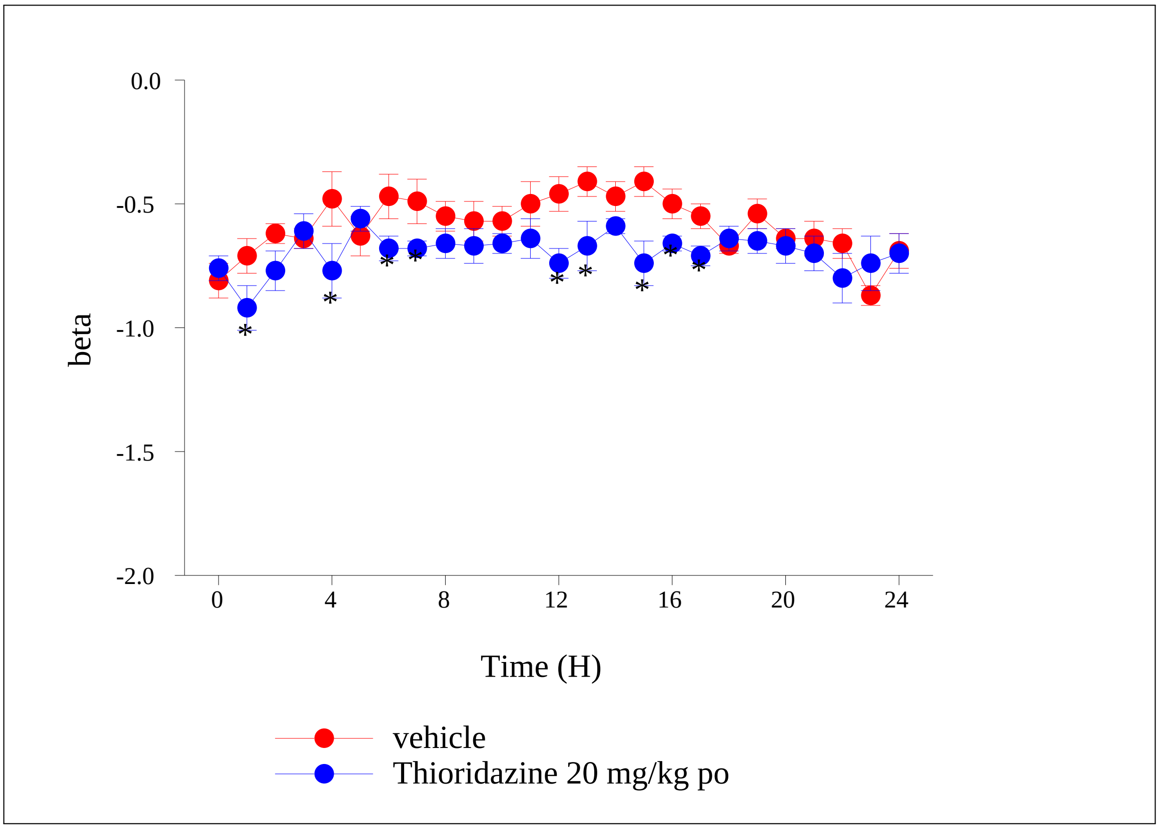

Results expressed in β
Repeated measures analysis of variance (RMANOVA)
Probability for Treatment factor: P=0.081
Probability for Time X Treatment interaction: P=0.001
$\star$: P$\leq$0.05 (LSD)
LSD=0.2 - Least significant difference for α type-1 error=5%
MDD=0.2 - Minimum detectable difference for α type-1 error=5% and β type-2 error=20% (*i.e.*power=80%)
Electronic authentication: created by Pascal Champéroux on 11-FEV-2025 at 14:47:47.339
Study QTOS

Thioridazine 20 mg/kg po + atenolol

Figure 95 Thioridazine 20 mg/kg po + atenolol

One-step QTc dQTc

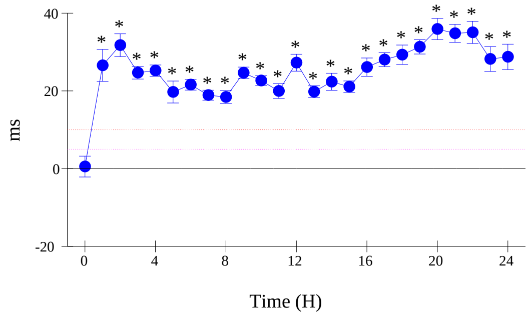

*: P<=0.05: one-sided 95% CI lower bound =>5ms
LSD= 2.2 ms

One-step QTc ddQTc

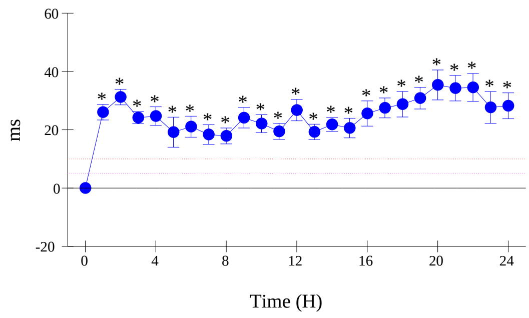

*: P<=0.05: one-sided 95% CI lower bound =>5ms
LSD= 3.4 ms

LogQTRR dQTc

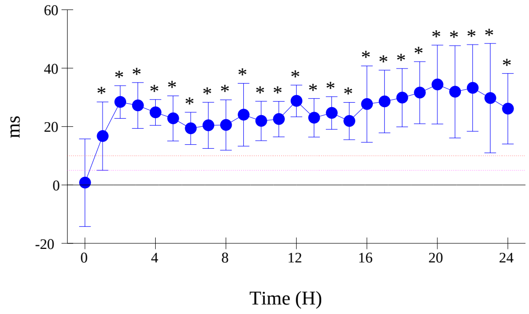

*: P<=0.05: one-sided 95% CI lower bound =>5ms
LSD= 8.6 ms

LogQTRR ddQTc

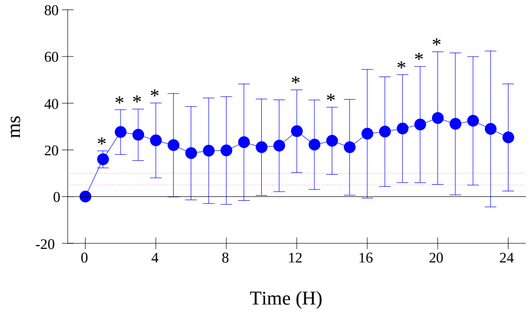

*: P<=0.05: one-sided 95% CI lower bound =>5ms
LSD= 22.1 ms

LinQTHR dQTc

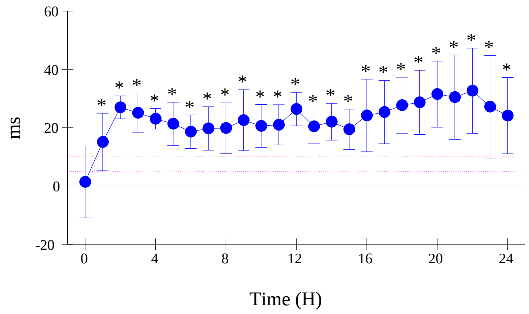

*: P<=0.05: one-sided 95% CI lower bound =>5ms
LSD= 8.6 ms

LinQTHR ddQTc

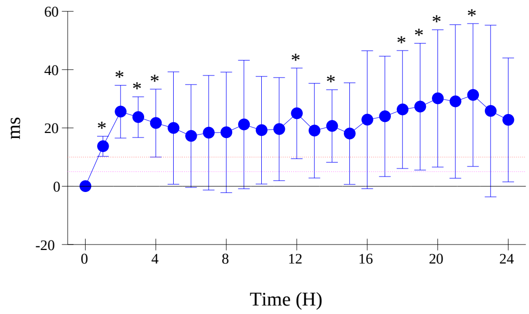

*: P<=0.05: one-sided 95% CI lower bound =>5ms
LSD= 19.3 ms

Figure 96 Thioridazine 20 mg/kg po + atenolol - Effect on β slope (one step QTc model)

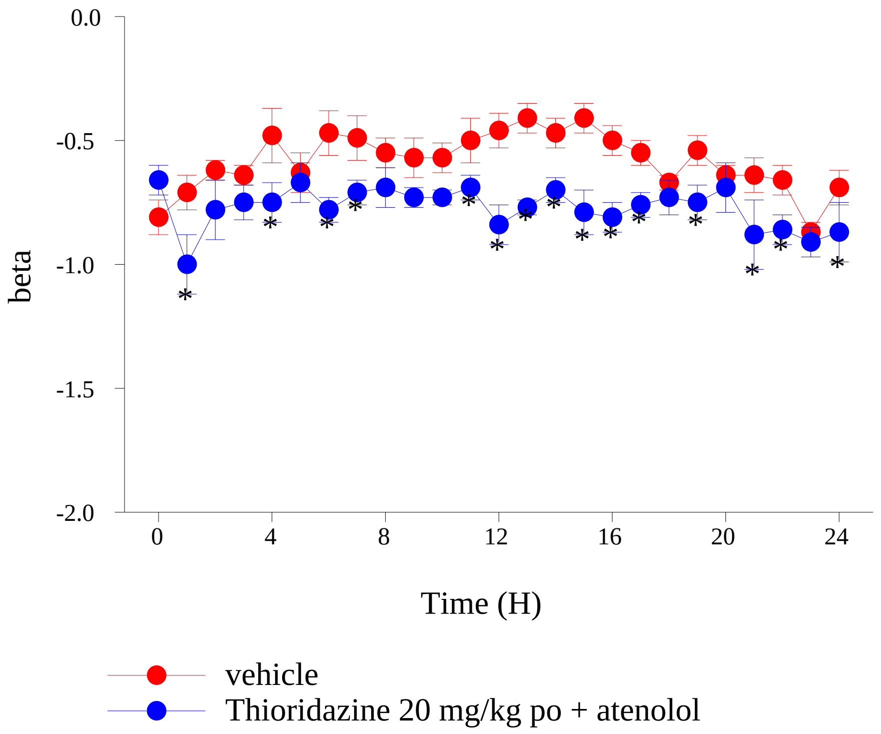

Results expressed in β
Repeated measures analysis of variance (RMANOVA)
Probability for Treatment factor: P=0.005
Probability for Time X Treatment interaction: P=0.012
$\star$: P$\leq$0.05 (LSD)
LSD=0.2 - Least significant difference for α type-1 error=5%
MDD=0.3 - Minimum detectable difference for α type-1 error=5% and β type-2 error=20% (*i.e.*power=80%)
Electronic authentication: created by Pascal Champéroux on 11-FEV-2025 at 14:47:47.546
Study QTOS

Verapamil 3 mg/kg po

Figure 97 Verapamil 3 mg/kg po

One-step QTc dQTc

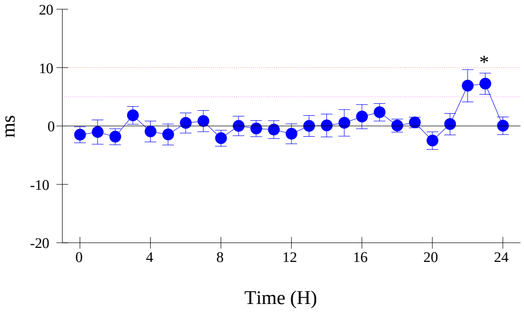

*: P<=0.05: one-sided 95% CI lower bound =>5ms
LSD= 1.7 ms

One-step QTc ddQTc

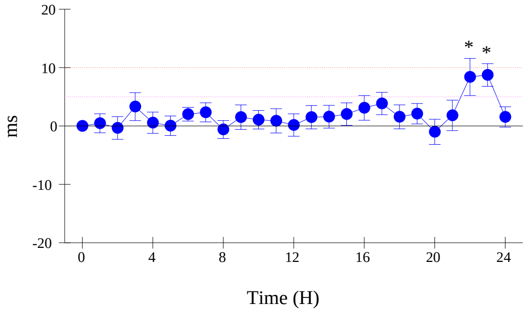

*: P<=0.05: one-sided 95% CI lower bound =>5ms
LSD= 1.9 ms

LogQTRR dQTc

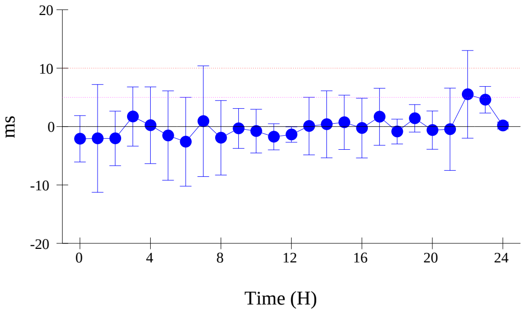

*: P<=0.05: one-sided 95% CI lower bound =>5ms
LSD= 4.9 ms

LogQTRR ddQTc

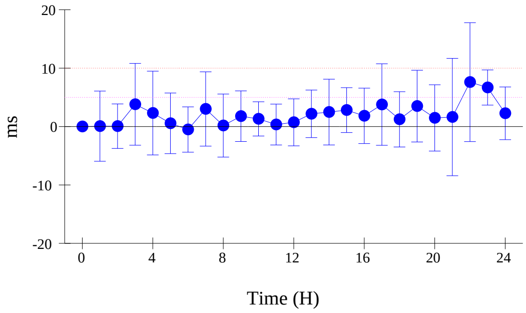

*: P<=0.05: one-sided 95% CI lower bound =>5ms
LSD= 4.7 ms

LinQTHR dQTc

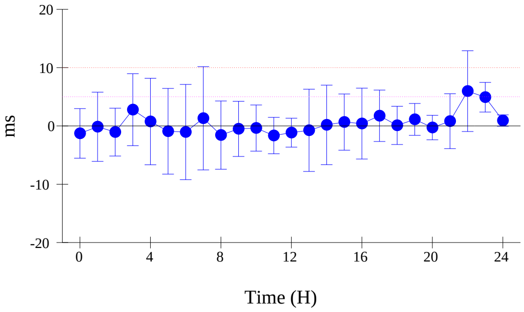

*: P<=0.05: one-sided 95% CI lower bound =>5ms
LSD= 4.7 ms

LinQTHR ddQTc

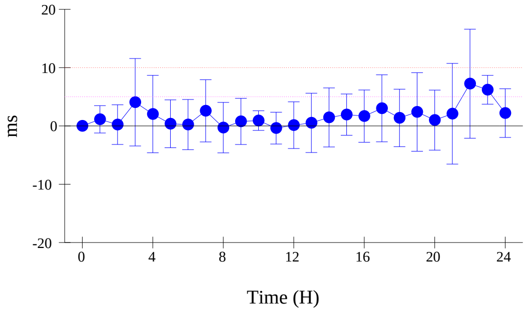

*: P<=0.05: one-sided 95% CI lower bound =>5ms
LSD= 4.3 ms

Figure 98 Verapamil 3 mg/kg po - Effect on β slope (one step QTc model)

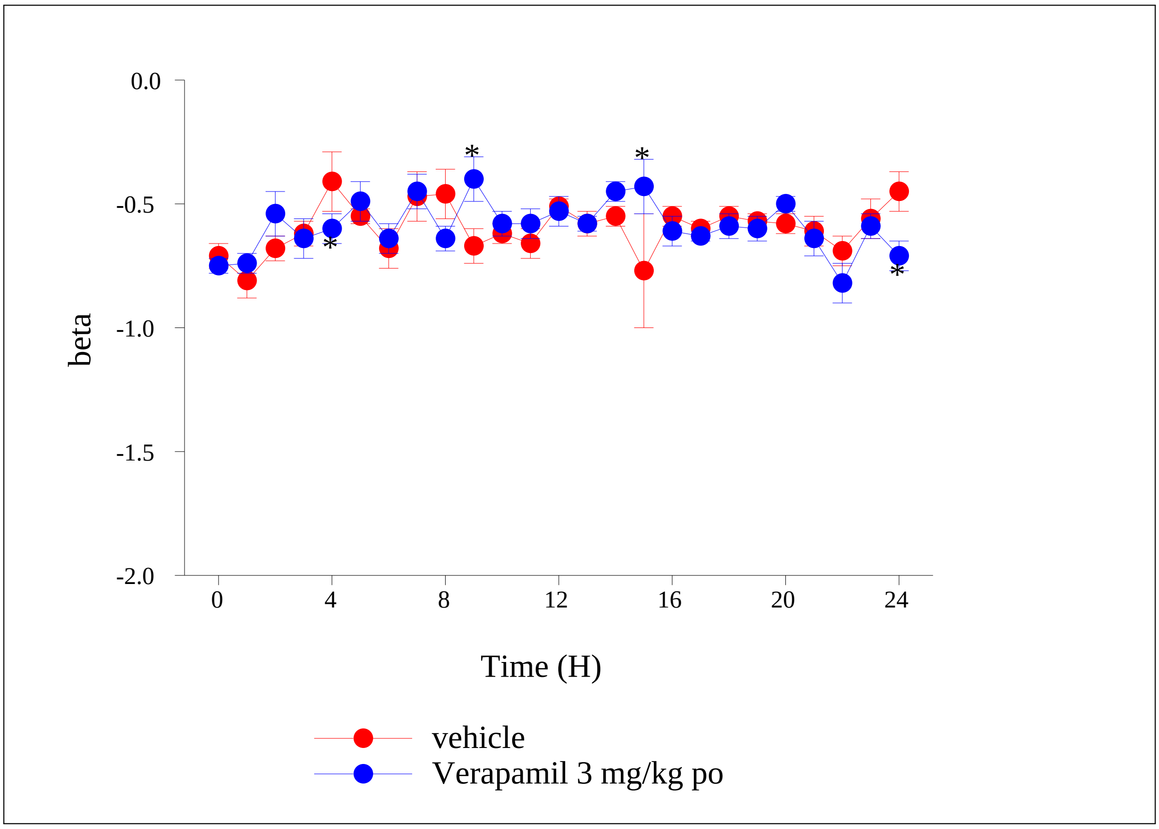

Results expressed in β
Repeated measures analysis of variance (RMANOVA)
Probability for Treatment factor: P=0.836
Probability for Time X Treatment interaction: P=0.012
$\star$: P$\leq$0.05 (LSD)
LSD=0.2 - Least significant difference for α type-1 error=5%
MDD=0.3 - Minimum detectable difference for α type-1 error=5% and β type-2 error=20% (*i.e.*power=80%)
Electronic authentication: created by Pascal Champéroux on 11-FEV-2025 at 14:47:47.722
Study QTOS

Verapamil 10 mg/kg po

Figure 99 Verapamil 10 mg/kg po

One-step QTc dQTc

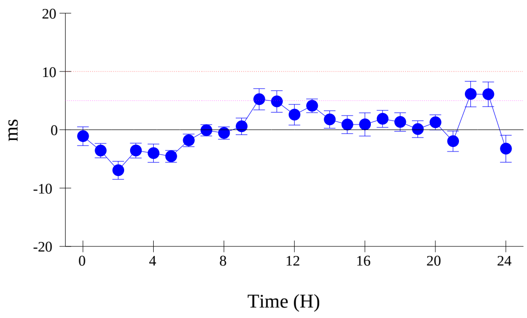

*: P<=0.05: one-sided 95% CI lower bound =>5ms
LSD= 1.5 ms

One-step QTc ddQTc

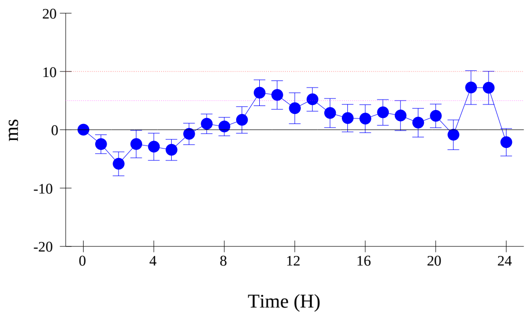

*: P<=0.05: one-sided 95% CI lower bound =>5ms
LSD= 2.3 ms

LogQTRR dQTc

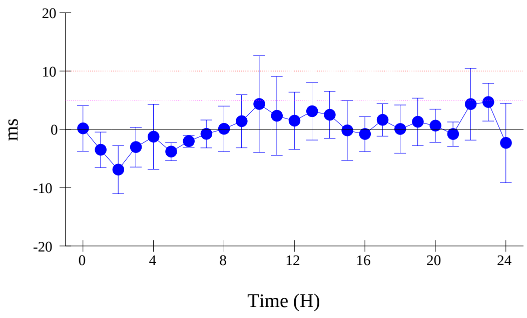

*: P<=0.05: one-sided 95% CI lower bound =>5ms
LSD= 4 ms

LogQTRR ddQTc

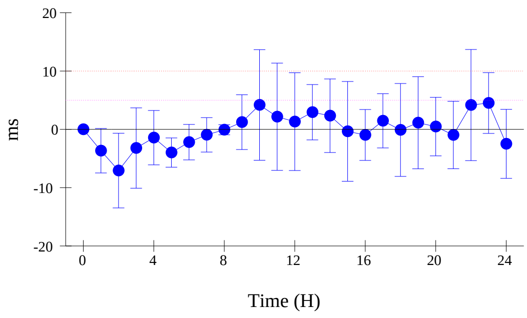

*: P<=0.05: one-sided 95% CI lower bound =>5ms
LSD= 5.2 ms

LinQTHR dQTc

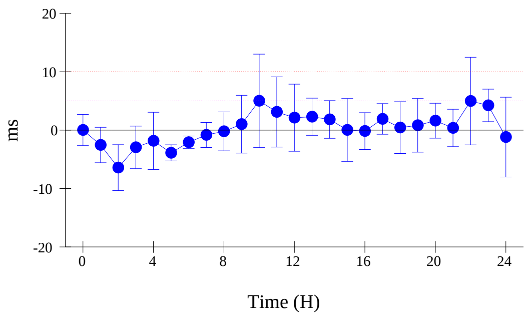

*: P<=0.05: one-sided 95% CI lower bound =>5ms
LSD= 3.3 ms

LinQTHR ddQTc

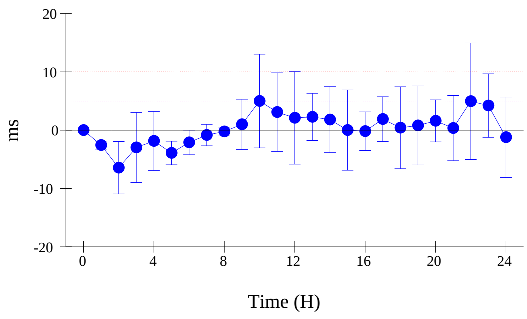

*: P<=0.05: one-sided 95% CI lower bound =>5ms
LSD= 5.1 ms

Figure 100 Verapamil 10 mg/kg po - Effect on β slope (one step QTc model)

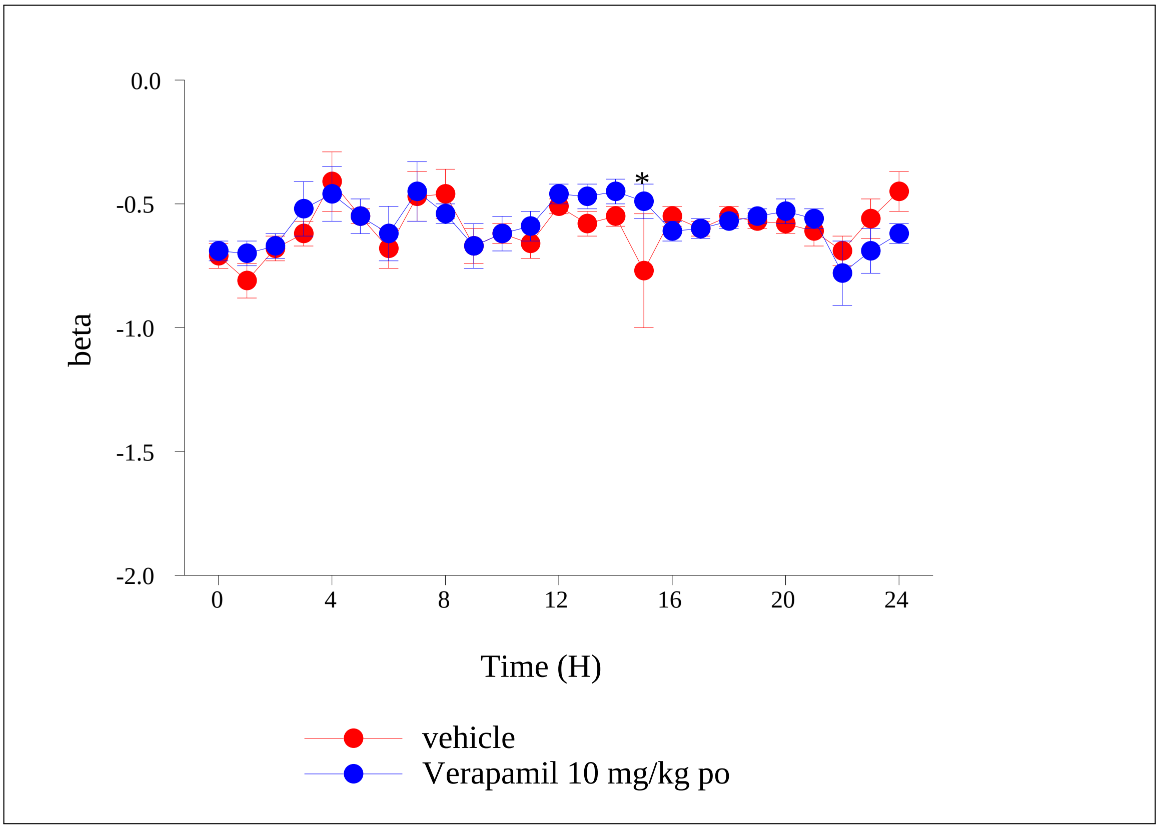

Results expressed in β
Repeated measures analysis of variance (RMANOVA)
Probability for Treatment factor: P=0.523
Probability for Time X Treatment interaction: P=0.782
$\star$: P$\leq$0.05 (LSD)
LSD=0.2 - Least significant difference for α type-1 error=5%
MDD=0.3 - Minimum detectable difference for α type-1 error=5% and β type-2 error=20% (*i.e.*power=80%)
Electronic authentication: created by Pascal Champéroux on 11-FEV-2025 at 14:47:47.899
Study QTOS

Verapamil 30 mg/kg po

Figure 101 Verapamil 30 mg/kg po

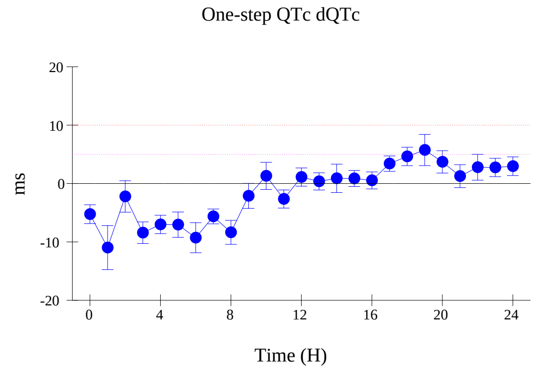

*: P<=0.05: one-sided 95% CI lower bound =>5ms
LSD= 1.9 ms

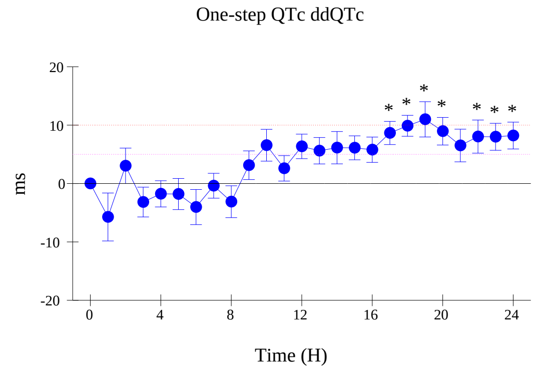

*: P<=0.05: one-sided 95% CI lower bound =>5ms
LSD= 2.4 ms

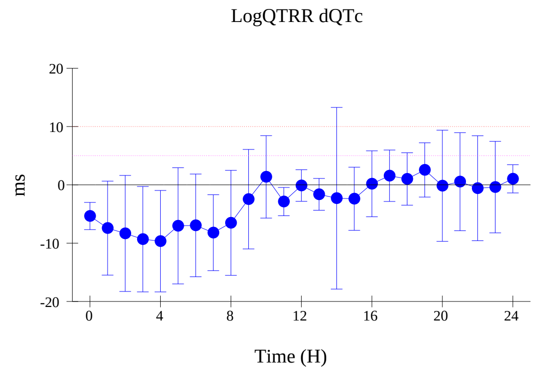

*: P<=0.05: one-sided 95% CI lower bound =>5ms
LSD= 7.8 ms

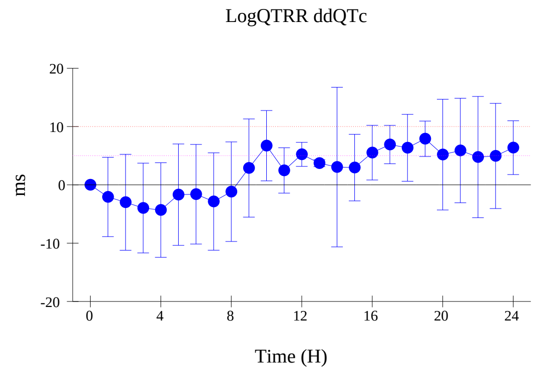

*: P<=0.05: one-sided 95% CI lower bound =>5ms
LSD= 7.7 ms

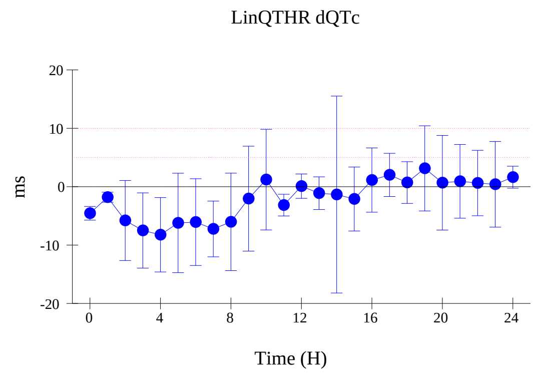

*: P<=0.05: one-sided 95% CI lower bound =>5ms
LSD= 6.3 ms

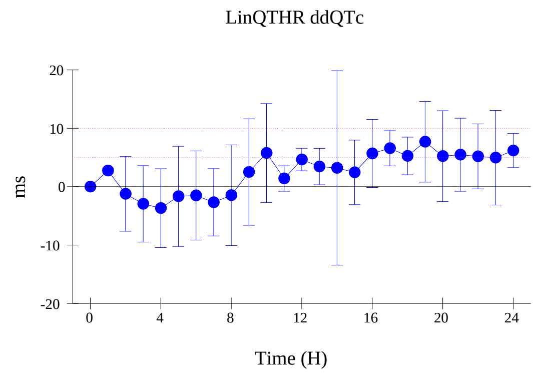

*: P<=0.05: one-sided 95% CI lower bound =>5ms
LSD= 6.3 ms

Figure 102 Verapamil 30 mg/kg po - Effect on β slope (one step QTc model)

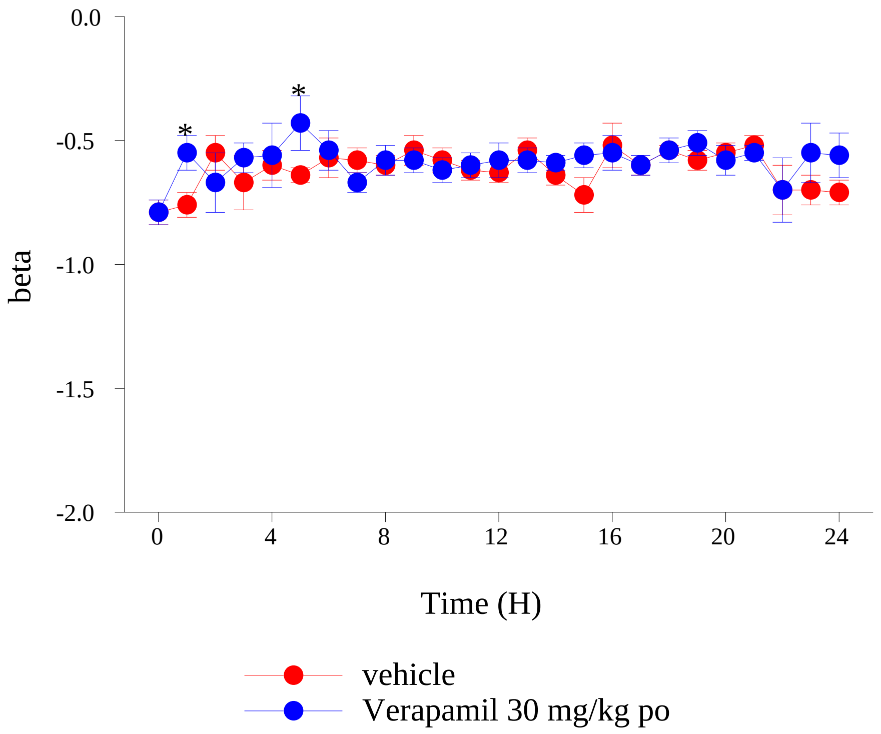

Results expressed in β
Repeated measures analysis of variance (RMANOVA)
Probability for Treatment factor: P=0.358
Probability for Time X Treatment interaction: P=0.562
$\star$: P$\leq$0.05 (LSD)
LSD=0.2 - Least significant difference for α type-1 error=5%
MDD=0.3 - Minimum detectable difference for α type-1 error=5% and β type-2 error=20% (*i.e.*power=80%)
Electronic authentication: created by Pascal Champéroux on 11-FEV-2025 at 14:47:48.091
Study QTOS
